# Concepts and Engineering Aspects of a Neutron Resonance Spin-Echo Spectrometer for the National Institute of Standards and Technology Center for Neutron Research

**DOI:** 10.6028/jres.119.005

**Published:** 2014-04-08

**Authors:** Jeremy C. Cook

**Affiliations:** National Institute of Standards and Technology, Gaithersburg, MD 20899

**Keywords:** neutron instrumentation simulations, neutron resonance spin-echo spectrometers, quasi-elastic neutron scattering

## Abstract

Following a brief introduction, the Neutron Resonance Spin-Echo (NRSE) principle is discussed classically in Sec. 2. In Sec. 3, two idealized 4-coil NRSE spectrometers are discussed (one using single *π*-flipper coil units and one using paired “bootstrap” coils); some idealized (exact *π*-flip) expressions are given for the spin-echo signal and some theoretical limitations are discussed. A more quantum mechanical discussion of NRSE is presented in Sec. 4 and additional theory related to the spin-echo signal, including wavelength-dependence, is given is Sec. 5. Factors affecting the instrumental resolution are discussed in Sec. 6. In Sec. 7, a variety of engineering issues are assessed in the context of challenging performance goals for a NIST Center for Neutron Research (NCNR) NRSE spectrometer. In Sec. 8, some Monte Carlo simulations are presented that examine the combined influences of spectrometer imperfections on the NRSE signal. These are compared with analytical predictions developed in previous sections. In Sec. 9, possible alternatives for a NCNR NRSE spectrometer configuration are discussed together with a preliminary assessment of the spectrometer neutron guide requirements. A summary of some of the useful formulas is given in Appendix A.

## 1. Introduction

Neutron Resonance Spin-Echo (NRSE) [1,2] is an alternative to the conventional Neutron Spin-Echo (NSE) technique [3], whereby the long solenoids of the latter are replaced by r.f. spin-flippers separated by regions in which there is ideally no magnetic field. For this reason, NRSE is occasionally referred to as “Zero-Field Spin-Echo”. Neutron Spin-Echo spectrometers have the distinguishing characteristic of being able to resolve neutron scattering energy exchanges that are much smaller than the energy bandwidth of the incident neutron beam. This contrasts with conventional time-of-flight spectrometers, where the minimum time uncertainty is limited by the incident pulse duration and the associated velocity spread of the incident beam. Some of the important issues for high-resolution NRSE spectrometer design are explored in the following sections.

## 2. Classical Description of Resonance Spin-Echo

### 2.1 Classical Principle of Operation

In a common NRSE configuration, four short resonant r.f. flipper coils replace the static field boundaries of the classical NSE spectrometer and the intervening space has zero magnetic field. The r.f. fields in the first and second coils must be phased-locked and in the third and fourth coils. The phase of the r.f. field at the times of neutron passage through the coils acts effectively as a neutron clock (as opposed to the number of Larmor precessions performed in the solenoids of a conventional NSE instrument). In the following illustration, we adopt the coordinate system used by Gähler and Golub in Ref. [2], which differs from the one used in Ref. [1].

**Fig. 1:**
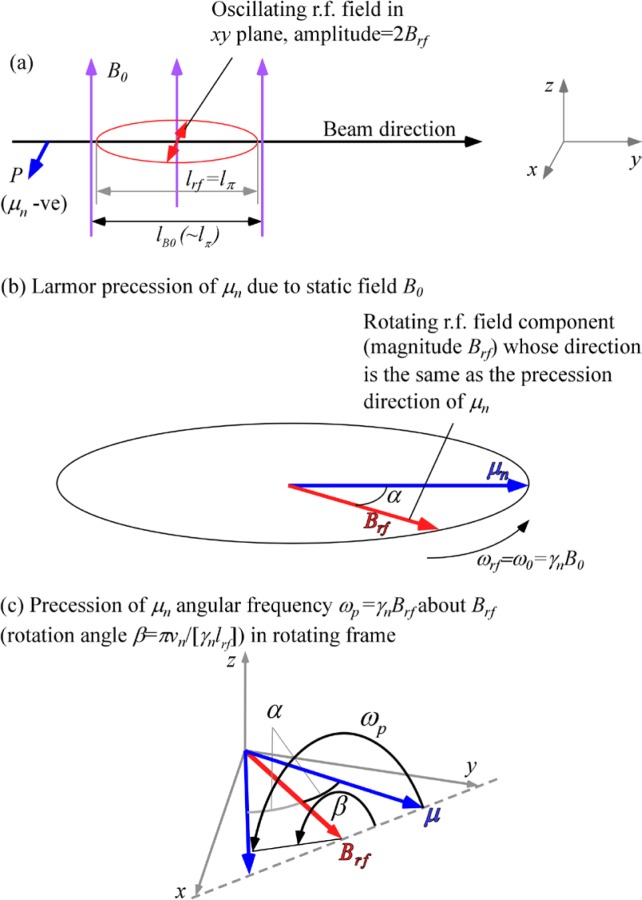
(a) Coordinate system showing initial neutron polarization direction. (b) Larmor precession (with angular velocity *ω_0_*) of the neutron magnetic moment, *µ_n_*, in the *xy* plane due to a static magnetic field *B_0_* applied along the *z*-axis; (c) Larmor precession (at angular velocity *ω_p_*) of *µ_n_* with respect to the resonant component of the r.f. field, *B_rf_*, as viewed in a frame which is rotating with *B_rf_*. At resonance *ω_rf_* = *ω_0_*.

With reference to Fig. 1(a), consider an incident monochromatic beam of neutrons of velocity *v_n_*, traveling along the *y*-axis, initially polarized parallel to the *x*-axis. In the flipper coil, a static magnetic field of magnitude *B_0_* is applied in the *z* direction and an oscillating r.f. field is applied in a plane perpendicular to *B_0_* (i.e., in the *xy* plane). We use the notation *l_π_* to define the length (in the beam direction) of the region of intersection of the static field region (length *l_B0_*) and the r.f. field region (length *l_rf_*), i.e.,

(1) $$l_{\pi }=l_{B_{0}}\cap l_{rf},$$

since *l_B0_* and *l_rf_* cannot be identical. Therefore, if the static field region completely encloses the r.f. region, *l_π_* = *l_rf_* and if the r.f. region completely encloses the static field region *l_π_* = *l_B0_*. Note that *l_π_* is distinct from the *length of the coil*, which is a useful parameter in some instances and is defined by

(2) $$l_{coil}=l_{B_{0}}\cup l_{rf}.$$

If one field region completely encloses the other, Eq. (2) may be restated as

(3) $$l_{coil}=\max(l_{B_{0}},l_{rf}).$$

In r.f. flipper coils, the static field usually completely encloses the r.f. field region. Therefore, when discussing flipper coils we assume, by default, that *l_π_* ≡ *l_rf_* and *l_coil_* ≡ *l_B0_*, as indicated in Fig. 1, and that the r.f. field is always in a region in which there is a perpendicular static field, with the possibility of a short static-only field region either side of the r.f. coil. The following provides a classical illustration of the NRSE principle. It agrees with the quantum mechanical result, provided that the following approximations are valid:

1. |*B_0_*| >> |*B_rf_* | (see Refs. [4,5]).
2. The interacting component of the oscillating field is considered as a purely rotating field.
3. The Zeeman splitting due to *B_0_* (2*µ_n_B_0_*) << the kinetic energy of the neutron, *m_n_v*^2^/2.

**Fig. 2:**
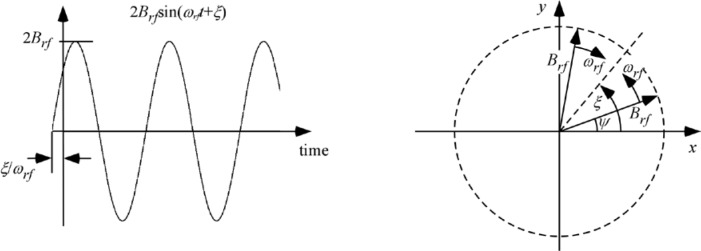
A sinusoidal oscillating field of angular frequency *ω_rf_* and amplitude 2*B_rf_* may be considered as being composed of two counter-rotating components at angular frequency *ω_rf_*, each of magnitude *B_rf_*.

Therefore, decomposing the oscillating field into two counter-rotating components, as shown in Fig. 2, the *resonant* component is the one that rotates in the same direction as the Larmor precession induced by the static field, *B_0_*; it is described by

(4) $${\vec{B}}_{rf}=B_{rf}(\hat{i}\cos\omega_{rf}t+\hat{j}\sin\omega_{rf}t),$$

where the sign of *ω_rf_* is chosen appropriately. The present approximation ignores the much weaker interaction of the counter-rotating (non-resonant) component of the r.f. field. We consider first the neutron spin with respect to the static field *B_0_*. If *B_0_* >> *B_rf_*, the neutron spin may be assumed to precess in the *xy* plane with Larmor angular frequency

(5) $$\omega_{0}=\gamma_{n}B_{0},$$

where *γ_n_* is the gyromagnetic ratio of the neutron, defined as the ratio of the magnitude of the neutron magnetic moment to the magnitude of its angular momentum, where

(6) $$\gamma_{n}=\frac{2\left|\mu_{n}\right|}{\hbar }=1.832472\times 10^{8}{\text{rad s}}^{-1}T^{-1}$$

and *µ_n_* is the maximum component of the neutron magnetic moment measurable along a single axis. For *B_0_* measured in Tesla, we have

(7) $$\omega_{0}[{\text{rads s}}^{-1}]=1.832472\times 10^{8}B_{0}[T],$$

or

(8) $$v_{0}[\text{MHz}]=29.1647B_{0}[T].$$

If the r.f. angular frequency, *ω_rf_*, is tuned exactly to the value *ω_0_*, one component of the r.f. field rotates about the *z*-axis synchronously with the precessing neutron spin (i.e., is “on resonance” – Fig. 1(b)). In this case, we can write

(9) $$\omega_{rf}\left[{\text{rad s}}^{-1}\right]=1.832472\times 10^{8}B_{0}[T]\;on\text{-}resonance$$

and

(10) $$v_{rf}\left[\text{MHz}\right]=29.1647B_{0}[T]\;on\text{-}resonance.$$

Because we are ignoring the effects of the counter-rotating (non-resonant) r.f. component, Fig. 1(b) shows only the resonant component of the r.f. field.

In the rest frame of the resonant component of *B_rf_* (i.e. the frame rotating in the *xy* plane at angular speed *ω_rf_* = *ω_0_*), the neutron spin precesses around the axis defined by the direction of *B_rf_* in this frame (Fig. 1(c)) at angular frequency *ω_p_*, where

(11) $$\omega_{p}=\gamma_{n}B_{rf}.$$

As a result, the precession angle, *β*, around this axis depends on the time spent in the r.f. (combined) field region, i.e.,

(12) $$\beta =\gamma_{n}B_{rf}t\approx \gamma_{n}B_{rf}\frac{l_{rf}}{v_{n}}=\frac{\gamma_{n}m_{n}}{h}B_{rf}l_{rf}\lambda_{n}\;(\text{approximation valid for small beam divergence}),$$

where *t* is the time of flight of the neutron across the region *l_rf_*. Note that the approximation in Eq. (12) implies that the beam divergence is sufficiently small that the substitution *t* ≈ *l_rf_/v_n_* can be made. In order to create a *π* flip of the neutron spin around *B_rf_* (such that the neutron spin returns to the *xy* plane), the magnitude of *B_rf_* must be tuned in order to satisfy

(13) $$\left|B_{rf}\right|\approx \pi \frac{v_{n}}{\gamma_{n}l_{rf}}=\pi \frac{h}{\gamma_{n}m_{n}l_{rf}\lambda_{n}}\;forsmallbeamdivergence$$

from which we have

(14) $$B_{rf}[T]≃\frac{6.782232\times 10^{-5}}{l_{rf}[m]\lambda_{n}\left[Å\right]}=\frac{6.782232\times 10^{-5}}{l_{\pi }[m]\lambda_{n}\left[Å\right]}.$$

Note that the peak amplitude of the applied r.f. field has *twice* the magnitude of the resonant component (i.e., $B_{rf}^{pk}=2B_{rf}$). In other words,

(15) $$B_{rf}^{pk}[T]=2B_{rf}[T]≃\frac{1.35645\times 10^{-4}}{l_{rf}[m]\lambda_{n}\left[Å\right]}=\frac{1.35645\times 10^{-4}}{l_{\pi }[m]\lambda_{n}\left[Å\right]}.$$

Typically, the time of flight through such coils (of several cm in length) is around 50*µ*s (within a factor of a few, depending on the neutron wavelength), therefore *B_rf_* is typically a few tenths of a mT. By contrast *B_0_* may range up to about 25 mT or more, so clearly for the larger values of *B_0_* the assumption *B_0_* >> *B_rf_* is valid.

At exact resonance, where *π*-flips return the spin initially in the *xy* plane back into the *xy* plane after passage through the combined field region, it is relatively straightforward to visualize the operation. Following Ref. [1], we denote the phase of the neutron spin in the *xy* plane relative to some fixed origin by *φ* and the phase of the resonant component of *B_rf_* with respect to the same origin by *ψ*. If the initial phase angle of the neutron spin lags the resonant component of *B_rf_* by *α* on entry into the r.f. field (see Fig. 1(c)), and Eq. (13) is satisfied, then the neutron spin will *lead B_rf_* by *α* in the rotating frame on exiting the field region. Transforming back into the laboratory frame at the coil exit, we add on the phase change of the r.f. field during the neutron flight time through the coil (≡*ω_0_l_π_*/*v_n_*) plus the (usually small) additional Larmor precession angle in the *xy* plane due to the *B_0_* field-only regions either side of the r.f. coil (≈*ω_0_*(*l_B0_*−*l_π_*)/*v_n_*). Consequently, we find that the neutron spin has changed its *xy*-plane phase angle in the coil by an amount ≈ 2*α* +*ω_0_l_B0_*/*v_n_*. Thus for an *ideal π*-flipper, the neutron spin phase change in the coil is governed by the operator

(16) $$\varphi^{\prime }=\psi^{\prime }+\psi -\varphi ,$$

where “unprimed” and “primed” refer to the “entrance” and “exit” of the coil respectively. When the coil is tuned for resonance

(17) $$\psi^{\prime }\approx \psi +\omega_{0}\frac{l_{B_{0}}}{v_{n}},$$

the operator in Eq. (16) can be rewritten as

(18) $$\varphi^{\prime }\approx 2\psi +\omega_{0}\frac{l_{B_{0}}}{v_{n}}-\varphi .$$

Again small beam divergence is implied by replacing the time of flight through the coil by *l_B0_*/*v_n_*. If a zero field region of length *L_AB_* exists between the first coil (*A*) and second coil (*B*), the phase of the neutron spin leaving coil *A* is preserved until its entry into coil *B* (a time *L_AB_*/*v_n_* later), whilst the r.f. field in coil *B* (which is phase-locked to the field in coil *A*) advances by an amount *ω_0_L_AB_*/*v_n_*. Thus, we have

(19) $$\varphi_{B}={\varphi^{\prime }}_{A}\approx 2\psi_{A}+\omega_{0}\frac{l_{B_{0}}}{v_{n}}-\varphi_{A}.$$

We have chosen the initial polarization direction (along the *x*-axis) to define *φ_A_*=0, therefore Eq. (19) becomes

(20) $$\varphi_{B}\approx 2\psi_{A}+\omega_{0}\frac{l_{B_{0}}}{v_{n}}$$

and

(21) $$\psi_{B}\approx {\psi^{\prime }}_{A}+\omega_{0}\frac{L_{AB}}{v_{n}}\approx \psi_{A}+\omega_{0}\frac{\left(L_{AB}+l_{B_{0}}\right)}{v_{n}}.$$

Reapplying Eq. (18) at the exit of a second identical coil (*B*) with the *same field directions*, one obtains

(22) $${\varphi^{\prime }}_{B}-\varphi_{A}={\varphi^{\prime }}_{B}\approx 2\omega_{0}\frac{\left(L_{AB}+l_{B_{0}}\right)}{v_{n}}.$$

On exiting coil *B*, the total spin phase change is now independent of the initial r.f. phase angle, *ψ_Α_*, at the entrance to coil *A* (which is random for a continuous neutron beam). Similar arguments hold for the third and fourth coils (*C* and *D*), other than the signs of the spin phase changes are reversed by applying the static fields in the opposite direction to those in coils *A* and *B*, i.e.,

(23) $${\varphi^{\prime }}_{D}-\varphi_{C}\approx -2\omega_{0}\frac{\left(L_{CD}+l_{B_{0}}\right)}{v_{n}}.$$

This means that the phase locking of the r.f. fields between coils *A* and *B* may be performed independently of that in coils *C* and *D*, provided that the frequencies are equal. The spin turn in each arm of the spectrometer is proportional to the neutron time of flight in each arm irrespective of the time of entry. The net spin phase change in the whole spectrometer is therefore

(24) $$\varphi ={\varphi^{\prime }}_{D}-\varphi_{A}\approx 2\omega_{0}\left[\frac{\left(L_{AB}+l_{B_{0}}\right)}{v_{i}}-\frac{\left(L_{CD}+l_{B_{0}}\right)}{v_{f}}\right]=2\gamma_{n}B_{0}\left[\frac{\left(L_{AB}+l_{B_{0}}\right)}{v_{i}}-\frac{\left(L_{CD}+l_{B_{0}}\right)}{v_{f}}\right],$$

where we have substituted *v_i_* for *v_n_* in Eq. (22) (incident beam) and *v_f_* for *v_n_* in Eq. (23) (scattered beam) to account for the possibility of neutron speed changes in scattering events.

This expression is entirely analogous with that for a conventional NSE spectrometer if the lengths of the precession solenoids, *L*, of the latter (operating with axial fields of magnitude *B_0_*) are associated with the quantities 2(*L_AB_+l_Β0_*) or 2(*L_CD_*+*l_Β0_*). In the NRSE configuration *L_AB_+l_Β0_* and *L_CD_*+*l_Β0_* correspond to the separation of the midpoints of the coils in each arm of the spectrometer, with *L_AB_* and *L_CD_* typically >> *l_Β0_*. The factor “2” implies a factor 2 advantage in NRSE resolution when comparing equivalent *B_0_L* in both techniques. However, large values of *B_0_* are usually more easily achieved in the long solenoids of a NSE spectrometer than in the compact r.f. flippers of the NRSE instrument (see for example Sec. 7.3.3.1). A generalization of Eq. (24) for different coil lengths is given in Sec. 3.3 and in Sec. 3.4, where it is shown how a “bootstrap” coil configuration [2] further increases the resolution factor from 2 to 2*N*, where *N* is commonly a small even integer (*N* ≥ 2). Henceforth, we concentrate on elastic or quasielastic applications of the NRSE technique.

### 2.2 Dispersion of the Flipper Coils

#### 2.2.1 Single Flipper

For *polychromatic* beams, the coil is tuned for *π* spin flips for the mean incident neutron velocity, 〈*v_i_*〉 (or mean incident wavelength, 〈*λ_i_*〉) such that, according to Eq. (12), we require

(25) $$\left|B_{rf}\right|=\frac{\pi \langle v_{i}\rangle }{\gamma_{n}l_{rf}}=\pi \frac{h}{\gamma_{n}m_{n}l_{rf}\langle \lambda_{i}\rangle }\;\text{coil}\;\pi \text{-flip tuning condition}.$$

It is clear that exact *π* turns about *B_rf_* occur for a unique neutron velocity or wavelength if |*B_rf_* | is kept constant^1^. The wavelength-dependence of the precession angle around *B_rf_* is referred to henceforth as *dispersion*. For a general wavelength, *λ_i_*, corresponding to a deviation from the mean Δ*λ_i_* = *λ_i_*−〈*λ_i_*〉, we have:

(26) $$\beta (\lambda_{i})≃\pi \left(1+\frac{\Delta \lambda_{i}}{\langle \lambda_{i}\rangle }\right)=\pi \frac{\lambda_{i}}{\langle \lambda_{i}\rangle }.$$

**Fig. 3:**
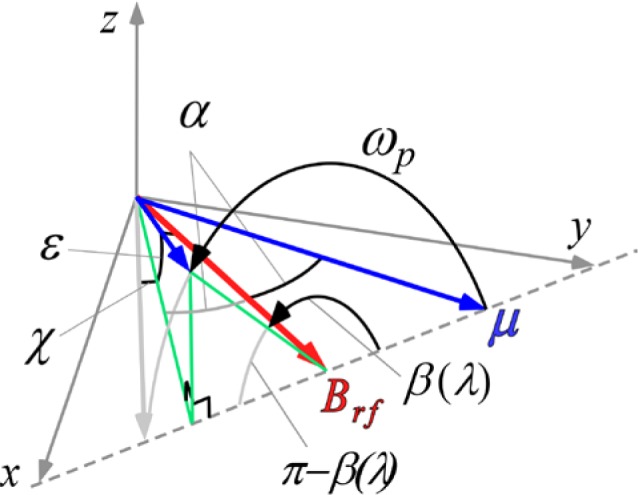
Classical precession of the neutron magnetic moment around the resonant component of the r.f. field in the rotating frame of the r.f. field component.

Equation (26) neglects the distribution in neutron flight times through the coil caused by beam divergence, which is typically narrow compared with that caused by the wavelength distribution. The particular case for *λ_i_* < 〈*λ_i_*〉 is illustrated in Fig. 3, resulting in a less-than-*π* precession of the neutron magnetic moment around *B_rf_* in the rotating frame. Similarly, an over-rotation occurs for *λ_i_* > 〈*λ_i_*〉. Thus, we see that some depolarization occurs due to the velocity spread and the maximum component in the *xy* plane no longer attains unity. For moderate *Δλ*, the depolarization is largely determined by the component of the magnetic moment out of the *xy* plane, (i.e., the angle *ε*), however, a (usually) smaller shift in the spin direction within the *xy* plane also occurs (i.e., the angle *χ*). For symmetric distributions of *λ* with respect to the mean, the angle *ε* is uniformly distributed above and below the *xy* plane and its magnitude depends on the angle *α*. It is zero for *α* = 0 and maximum for *α* = *π*/2. In fact,

(27) $$\cos\;\varepsilon =\sqrt{\cos^{2}\alpha +\sin^{2}\alpha \;\cos^{2}\beta }$$

and

(28) $$\cos\;\chi =\frac{\cos^{2}\alpha -\sin^{2}\alpha \cos\;\beta }{\cos\;\varepsilon }.$$

We see from Fig. 3 that for *Δλ* → 0, *β* → *π*, *ε* → 0, and therefore *χ* → 0, as expected.

For a continuous neutron beam with symmetrically-distributed wavelengths the ratio of the polarization “with dispersion” to that ignoring dispersion (or for a purely monochromatic beam, *I*(*λ_i_*) = *δ* (*λ_i_*)) after passage through the device is therefore

(29) $${\langle \frac{P_{disp}}{P_{ideal}}\rangle }_{I(\lambda_{i}),\psi }=\langle \cos\;\varepsilon \cos\;\chi \rangle =\langle \cos^{2}\alpha -\sin^{2}\alpha \cos\;\beta \rangle ={\langle 1-\sin^{2}\alpha \left[1+\cos\left(\pi \frac{\lambda_{i}}{\langle \lambda_{i}\rangle }\right)\right]\rangle }_{I(\lambda_{i}),\alpha }.$$

Qualitatively this is the component (or dot product) of the actual unit spin unit vector projected onto the “perfect” spin direction, averaged over all *α* and over the neutron wavelength spectrum, *I*(*λ_i_*). We note that *α* is random over 2*π* radians for a continuous beam and we may set 〈sin^2^*α*〉 = 1/2 in Eq. (29). Also using the identity cos *β* = 1–2 sin^2^(*β*/2), the average over *α* becomes

(30) $$\frac{P_{disp}}{P_{ideal}}=\sin^{2}\left(\frac{\pi }{2}\frac{\lambda_{i}}{\langle \lambda_{i}\rangle }\right)\;\text{averaged over}\;\alpha ,\;\text{continuous beam}.$$

*This is exactly the quantum-mechanically derived result for the spin flip probability for the π flipper (see Eq. (89) in Sec. 4.2.1.1) when the flipper is tuned for resonance and for exact π flips for the mean neutron wavelength 〈λ_i_〉.* Performing the average of Eq. (30) over the normalized incident wavelength spectrum *I*(*λ_i_*), we have finally

(31) $${\langle \frac{P_{disp}}{P_{ideal}}\rangle }_{I(\lambda_{i}),\psi }=\int I(\lambda_{i})\sin^{2}\left(\frac{\pi }{2}\frac{\lambda_{i}}{\langle \lambda_{i}\rangle }\right)d\lambda_{i}.$$

For normalized *rectangular intensity distributions* with 〈*λ_i_*〉−*Δλ_FW_*/2 ≤ *λ_i_* ≤ 〈*λ_i_*〉+*Δλ_FW_*/2 with *I_pk_* = 1/*Δλ_FW_*, we have

(32) $$\begin{array}{l} {\langle \frac{P_{disp}}{P_{ideal}}\rangle }_{1coil}(\text{rectangular})=\frac{2}{\Delta \lambda_{FW}}\int_{\langle \lambda_{i}\rangle }^{\langle \lambda_{i}\rangle +\Delta \lambda_{FW}/2}\sin^{2}\left(\frac{\pi }{2}\frac{\lambda_{i}}{\langle \lambda_{i}\rangle }\right)d\lambda_{i}\\ =\frac{1}{2}+\frac{\langle \lambda_{i}\rangle }{\Delta \lambda_{FW}\pi }\sin\left(\frac{\pi }{2}\frac{\Delta \lambda_{FW}}{\langle \lambda_{i}\rangle }\right). \end{array}$$

Defining the fractional full width of the spectrum by *Λ_FW_*, i.e.,

(33) $$\Lambda_{FW}=\Delta \lambda_{FW}/\langle \lambda_{i}\rangle ,$$

Eq. (32) is expressed more neatly as

(34) $${\langle \frac{P_{disp}}{P_{ideal}}\rangle }_{1coil}(\text{rectangular})=\frac{1}{2}+\frac{1}{\Lambda_{FW}\pi }\sin\left(\frac{\pi }{2}\Lambda_{FW}\right).$$

For *triangular intensity distributions* with FWHM = *Δλ_FWHM_*, we can use the normalized function

$$I(\lambda_{i})=\frac{1}{\Delta \lambda_{FWHM}}\left[1+\frac{\left(\lambda_{i}-\langle \lambda_{i}\rangle \right)}{\Delta \lambda_{FWHM}}\right]$$

in the interval 〈*λ_i_*〉−*Δλ_FWHM_* ≤ *λ_i_* ≤ 〈*λ_i_*〉, therefore

(35) $${\langle \frac{P_{disp}}{P_{ideal}}\rangle }_{1coil}(\text{triangular})=\frac{2}{\Delta \lambda_{FWHM}}\int_{\langle \lambda_{i}\rangle -\Delta \lambda_{FWHM}}^{\langle \lambda_{i}\rangle }\left(1+\frac{\lambda_{i}-\langle \lambda_{i}\rangle }{\Delta \lambda_{FWHM}}\right)\sin^{2}\left(\frac{\pi }{2}\frac{\lambda_{i}}{\langle \lambda_{i}\rangle }\right)d\lambda_{i}.$$

In analogy with Eq. (33) we set

(36) $$\Lambda_{FWHM}=\Delta \lambda_{FWHM}/\langle \lambda_{i}\rangle ,$$

then this integral becomes

(37) $${\langle \frac{P_{disp}}{P_{ideal}}\rangle }_{1coil}(\text{triangular})=\frac{1}{2}+\frac{1}{\pi^{2}\Lambda_{FWHM}^{2}}[1-\cos(\pi \Lambda_{FWHM})].$$

For *Gaussian intensity distributions* with FWHM = *Δλ_FWHM_*, we use the normalized function

(38) $$I(\lambda_{i})=\sqrt{\frac{4\ln2}{\pi \Delta \lambda_{FWHM}^{2}}}\exp\left[-\frac{4\ln2}{\Delta \lambda_{FWHM}^{2}}{\left(\lambda_{i}-\langle \lambda_{i}\rangle \right)}^{2}\right],$$

therefore,

(39) $${\langle \frac{P_{disp}}{P_{ideal}}\rangle }_{1coil}(\text{Gaussian})=4\sqrt{\frac{\ln2}{\pi \Delta \lambda_{FWHM}^{2}}}\int_{0}^{\infty }\exp\left[-\frac{4\ln2}{\Delta \lambda_{FWHM}^{2}}{\left(\lambda_{i}-\langle \lambda_{i}\rangle \right)}^{2}\right]\sin^{2}\left(\frac{\pi }{2}\frac{\lambda_{i}}{\langle \lambda_{i}\rangle }\right)d\lambda_{i}.$$

Using the definition of *Λ_FWHM_* from Eq. (36) for the Gaussian distribution this integral becomes

(40) $${\langle \frac{P_{disp}}{P_{ideal}}\rangle }_{1coil}(\text{Gaussian})=\frac{1}{2}\left(1+\exp\left[-{\left(\frac{\pi }{4}\right)}^{2}\frac{\Lambda_{FWHM}^{2}}{\ln2}\right]\right).$$

#### 2.2.2 Approximation for *M* Flippers

The above equations apply to a single flipper coil. If there are *M* identical flippers (e.g. *M* = 8 for a 4-coil *N* = 2 bootstrap instrument), and we assume that the neutron spectrum is unmodified through the spectrometer (elastic scattering, negligible absorption etc.), we can make the following approximation, provided that the cumulative spin rotation out of the *xy* plane remains small:

We rewrite Eq. (30) approximately as the product of the *M* flipper coil efficiencies, prior to averaging over the spectrum, *I*(*λ_i_*), i.e.,

(41) $$\frac{P_{disp}}{P_{ideal}}(M)\approx \sin^{2M}\left(\frac{\pi }{2}\frac{\lambda_{i}}{\langle \lambda_{i}\rangle }\right).$$

The overall average flipping efficiency for the spectrometer is therefore described for the rectangular, triangular, and Gaussian incident spectra by expressions similar to Eqs. (32), (35), and (39), but with the sin^2^ replaced by sin^2^*^M^*, i.e., for the rectangular spectrum:

(42) $$\begin{array}{l} {\langle \frac{P_{disp}}{P_{ideal}}\rangle }_{M\;coils}(\text{rectangular})\approx \frac{2}{\Delta \lambda_{FW}}\int_{\langle \lambda_{i}\rangle }^{\frac{2\langle \lambda_{i}\rangle +\Delta \lambda_{FW}}{2}}\sin^{2M}\left(\frac{\pi }{2}\frac{\lambda_{i}}{\langle \lambda_{i}\rangle }\right)d\lambda_{i}\\ \approx \frac{1}{2^{2M}}\left[\left(\begin{array}{c} 2M\\ M \end{array}\right)+\frac{4}{\pi \Lambda_{FW}}\sum_{k=0}^{M-1}\left(\begin{array}{c} 2M\\ k \end{array}\right)\frac{\sin\left(\left(M-k\right)\frac{\pi }{2}\Lambda_{FW}\right)}{\left(M-k\right)}\right], \end{array}$$

where $\left(\begin{array}{c} n\\ k \end{array}\right)$ is the binomial coefficient.

Likewise for triangular *I*(*λ_i_*):

(43) $$\begin{array}{l} {\langle \frac{P_{disp}}{P_{ideal}}\rangle }_{M\;coils}(\text{triangular})\approx \frac{2}{\Delta \lambda_{FWHM}}\int_{\langle \lambda_{i}\rangle -\Delta \lambda_{FWHM}}^{\langle \lambda_{i}\rangle }\left(1+\frac{\lambda_{i}-\langle \lambda_{i}\rangle }{\Delta \lambda_{FWHM}}\right)\sin^{2M}\left(\frac{\pi }{2}\frac{\lambda_{i}}{\langle \lambda_{i}\rangle }\right)d\lambda_{i}\\ \approx \frac{1}{2^{2M}}\left[\left(\begin{array}{c} 2M\\ M \end{array}\right)+\frac{4}{\pi^{2}\Lambda_{FWHM}^{2}}\sum_{k=0}^{M-1}\left(\begin{array}{c} 2M\\ k \end{array}\right)\left\{\frac{1-\cos\left[\left(M-k\right)\pi \Lambda_{FWHM}\right]}{{\left(M-k\right)}^{2}}\right\}\right]. \end{array}$$

Finally, for Gaussian *I*(*λ_i_*)

(44) $${\langle \frac{P_{disp}}{P_{ideal}}\rangle }_{M\;coils}(\text{Gaussian})\approx 4\sqrt{\frac{\ln2}{\pi \Delta \lambda_{FWHM}^{2}}}\int_{\langle \lambda_{i}\rangle }^{\infty }\exp\left[-\frac{4\ln2}{\Delta \lambda_{FWHM}^{2}}{\left(\lambda_{i}-\langle \lambda_{i}\rangle \right)}^{2}\right]\sin^{2M}\left(\frac{\pi }{2}\frac{\lambda_{i}}{\langle \lambda_{i}\rangle }\right)d\lambda_{i}.$$

The accuracy of the approximation for *M* > 1 is demonstrated by some special case Monte Carlo calculations in Sec. 8.2. Table 1 and Table 2 show predicted values of ${\langle P_{disp}/P_{ideal}\rangle }_{M\;coils}$ for uniform, triangular, and Gaussian wavelength distributions for *M* = 8 (i.e., a typical 4-coil, *N* = 2 bootstrap configuration) and for *M* = 6 (e.g., a MIEZE-II type, *N* = 2 bootstrap configuration – see Sec. 9) respectively. The Gaussian results, which are quite similar to the triangular case, were obtained by numerical integration of Eq. (44) between the limits 〈*λ_i_*〉 and 〈*λ_i_*〉+2*Δλ_FWHM_*. Using larger upper limits of integration showed no change in the fifth significant figure and no significant effects of spectral truncation are encountered for *Λ* up to 0.5 in this case. The values in the tables, which are apparently independent of the Fourier time, represent theoretically maximal signals with polychromatic incident beams in an otherwise perfectly-constructed spectrometer with perfect polarizers. When other instrumental imperfections are present, typically the tabulated values are approached in the short Fourier time limit.

### 2.3 Coil Resonance Width

The coil flipping efficiencies given in the previous section are for optimally-tuned coils (exact resonance *ω_rf_* = *ω_0_*, and exact *π*-flips for the mean wavelength 〈*λ_i_*〉). They account only for dispersion. For moderate *Δλ*/*λ*, dispersion leads mainly to excursions of the spin vector out of the intended *x–y* plane accompanied by a usually small rotation of the spin component within the *x–y* plane. An additional question concerns non-optimal tuning of the coils arising either from (i) systematic differences between the Larmor frequency (*ω_0_*) and r.f. frequency (*ω_rf_*) or (ii) that caused by static field inhomogeneity when *ω_rf_* = 〈*ω_0_*〉; i.e., to what tolerance must *ω_rf_* match *ω_0_*? Alternatively, what is the resonance width? Alvarez and Bloch [6] provided a quantum mechanical result for the flipping efficiency (valid for static field magnitudes that are much larger than the oscillating field magnitude), which (almost) in their original notation is

(45) $$P=\sin^{2}\left[\left(\frac{\mu H_{1}t}{2\hbar }\right)\sqrt{\left(\frac{H_{1}^{2}+{\left(2\Delta H\right)}^{2}}{H_{1}^{2}}\right)}\right]/\left[\frac{H_{1}^{2}+{\left(2\Delta H\right)}^{2}}{H_{1}^{2}}\right],$$

where *t* is the time spent in the oscillating field, *µH_1_t* equates to *β*/2 (Eq. (12)), *H_1_* is the amplitude of the oscillating field $\equiv 2B_{rf}=B_{rf}^{pk}$, and

(46) $$\Delta H=H_{0}-H_{0}^{{}^{*}}$$

is the difference between the actual value of the static field and the value required for exact resonance (i.e., when *ω_0_* =*ω_rf_*). Thus, Eq. (46) can be re-expressed as

(47) $$\Delta H=\frac{1}{\gamma_{n}}\left(\omega_{0}-\omega_{rf}\right)=\frac{2\pi }{\gamma_{n}}(v_{0}-v_{rf}).$$

In our notation Eq. (45) becomes

(48) $$P=\sin^{2}\left[\left(\frac{\gamma_{n}B_{rf}l_{\pi }m_{n}\lambda_{n}}{2h}\right)\sqrt{\left(1+{\left\{\frac{\omega_{0}-\omega_{rf}}{\gamma_{n}B_{rf}}\right\}}^{2}\right)}\right]/\left[1+{\left\{\frac{\omega_{0}-\omega_{rf}}{\gamma_{n}B_{rf}}\right\}}^{2}\right],$$

where it is understood that *l_π_* = *l_rf_* for the typical flipper coil. For the special case that *B_rf_* is tuned to produce exact *π* flips for the mean wavelength 〈*λ_i_*〉, Eq. (48) becomes (see also Eq. (11))

(49) $$P=\sin^{2}\left[\left(\frac{\pi \lambda_{n}}{2\langle \lambda_{n}\rangle }\right)\sqrt{\left(1+{\left\{\frac{\omega_{0}-\omega_{rf}}{\omega_{p}}\right\}}^{2}\right)}\right]/\left[1+{\left\{\frac{\omega_{0}-\omega_{rf}}{\omega_{p}}\right\}}^{2}\right]B_{rf}\text{tuned for exact}\;\pi \;\text{flips at}\;\lambda_{n}=\langle \lambda_{n}\rangle ,$$

which, for exact resonance (*ω_rf_* = *ω_0_*) reduces to Eq. (30).

**Fig. 4:**
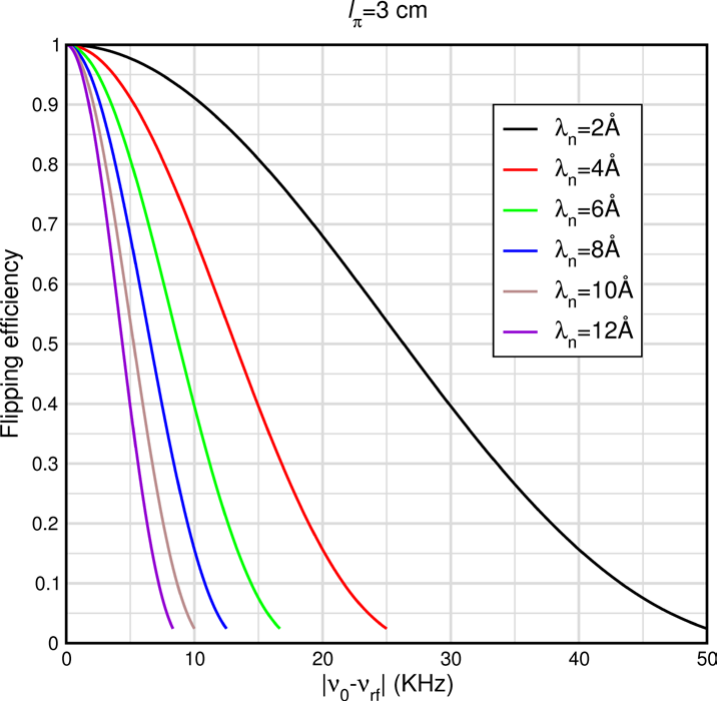
Coil flipping efficiencies calculated using Eq. (49) for a *π*-flipper with *l_π_* = 3 cm whose r.f. field magnitude *B_rf_* is tuned to produce exact *π*-flips for monochromatic, well collimated beams of wavelength *λ_n_* (zero dispersion approximation). These curves are plotted as a function of the difference in the Larmor frequency *ν_0_* and the r.f. frequency *ν_rf_* in kHz.

These equations quantify the effects of detuning the r.f. frequency from the nominal Larmor frequency or the effects of static field inhomogeneities (giving rise to a spread of *ω_0_* values). The latter is of concern for the spectrometer design tolerances. Equation (49) is plotted versus the frequency difference (in kHz) for *l_π_* = 3 cm (for a *single* wavelength *λ_n_* = 〈*λ_n_*〉) in Fig. 4. The Alvarez and Bloch formalism ignores certain rapidly oscillating terms when the static field magnitude is large compared to the oscillating field magnitude. When this is not the case, the oscillating field decreasingly approximates to a pure rotating field and the counter-rotating component increasingly plays a role. One manifestation of this is a shift in the resonance frequency as discussed in Ref. [5] (see Sec. 7.3.6.1). Eventually the r.f. flipper cannot function when *B_0_*/*B_rf_* falls below a certain threshold. The full width at half maximum of these resonance curves for a general value of *l_π_* is very well fitted by

(50) $$\Delta \nu_{FWHM}[\text{Hz}]=\frac{3.16\times 10^{3}}{l_{\pi }[m]\lambda_{n}\left[Å\right]}.$$

A useful quantity is the frequency shift for a 1 % drop in the flipping efficiency. This is well fitted by the following similar expression

(51) $$\Delta \nu_{99\%}[\text{Hz}]=\frac{198}{l_{\pi }[m]\lambda_{n}\left[Å\right]}.$$

Thus for longer wavelengths and longer coils the resonance sharpens, requiring increased tuning accuracy. Consequently, the tolerable field inhomogeneity also decreases with increasing *l_π_* and *λ_n_*.

### 2.4 Influence of *π*-Flipper Efficiency on Polarization

The term “flipper efficiency” usually excludes spin-independent effects such as scattering or absorption. Thus for a *π*-flipper of efficiency *f*, a fraction *f* of the spin-down component of a beam is converted to spin-up and vice-versa. Conversely, fractions (1−*f*) of the spin-down and spin-up components are transmitted with no change of their spin directions. For an incoming beam with spin-up and spin-down intensities $I_{0}^{+}$ and $I_{0}^{{}^{-}}$ respectively, the corresponding intensities in the outgoing beam are

(52) $$I_{1}^{+}=fI_{0}^{{}^{-}}+(1-f)I_{0}^{+}$$

and

(53) $$I_{1}^{{}^{-}}=fI_{0}^{+}+(1-f)I_{0}^{{}^{-}}.$$

The incident beam polarization is, by definition,

(54) $$P_{0}=\frac{I_{0}^{+}-I_{0}^{{}^{-}}}{I_{0}^{+}+I_{0}^{{}^{-}}}$$

therefore, by the same definition, the outgoing beam has polarization

(55) $$P_{1}=\frac{I_{0}^{+}-I_{0}^{{}^{-}}}{I_{0}^{+}+I_{0}^{{}^{-}}}=\frac{\left(1-2f)(I_{0}^{+}-I_{0}^{{}^{-}}\right)}{I_{0}^{+}+I_{0}^{{}^{-}}}=(1-2f)P_{0}.$$

Thus, the outgoing beam polarization is just the incoming beam polarization multiplied by the factor (1−2*f*). Note that for a perfect *π*-flipper (*f* = 1), *P_1_*= −*P_0_*, as expected.

## 3. Illustrations of Idealized 4-Coil NRSE Instruments

In the following examples, we illustrate the performance of a 4-coil unit NRSE spectrometer by assuming “perfect” *π*-flipper coils (Sec. 3.1). In Secs. 6 and 7, we discuss departures from the idealized performance due to the non-ideal nature of the components.

### 3.1 The Perfect *π*-Flipper Coil

We define the “perfect” *π*-flipper coil as having the following properties:

1. “Dispersionless” – the exact *π* flip operation is assumed to be independent of wavelength (i.e., all neutron spins start and finish in the *xy* plane - see Sec. 2.2).
2. Perfectly uniform and stable applied static field *B_0_* within the beam passage.
3. Perfectly stable (frequency and magnitude) and sinusoidal r.f. field *B_rf_*.
4. Perfect perpendicularity of the static, r.f. fields, and beam direction (⇒ zero divergence beam).
5. Perfectly-defined field boundaries along the beam direction.
6. Zero stray fields or leakage fields in the “zero-field” regions.
7. Perfectly transmitting for neutrons.

**Fig. 5:**
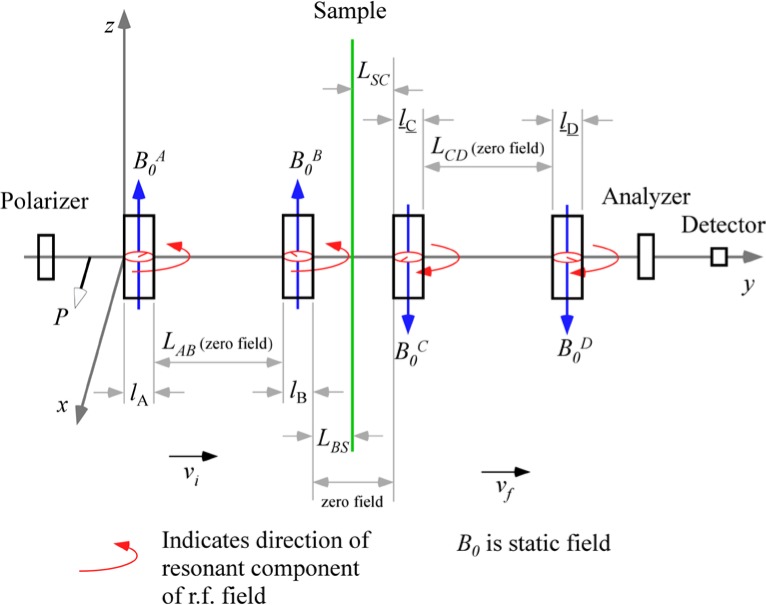
A 4-single *π* coil NRSE instrument.

Consider the coordinate system in Fig. 5. where the origin of the *y*-axis is chosen to coincide with the entrance to the first *π* flipper (*A*). We will assume that the static field magnitude, *B_0_*, in coils *A* and *B* is equal and that the static field magnitude, *B_1_*, in coils *C* and *D* is the same, i.e.,

(56) $$B_{0}^{A}=B_{0}^{B}=B_{0}$$

and

(57) $$B_{0}^{C}=B_{0}^{D}=B_{1}.$$

When the *π*-flipper r.f. frequency is on-resonance, we can write

(58) $$\omega_{rf}^{A}=\omega_{rf}^{B}=\omega_{0}=\gamma_{n}B_{0}$$

(59) $$\omega_{rf}^{C}=\omega_{rf}^{D}=\omega_{1}=\gamma_{n}B_{1}.$$

For elastic and small energy transfer quasielastic scattering (where the detailed balance factor is essentially 1 and the scattering function is symmetrical around zero energy transfer), we have 〈*v_i_*〉 = 〈*v_f_*〉. Therefore, the magnitude of the r.f. field is tuned to create *π*-flips for the mean incident velocity 〈*v_i_*〉 for all coils. This implies

(60) $$\left|B_{rf}^{k}\right|=\frac{\pi \langle v_{i}\rangle }{\gamma_{n}l_{k}}=\pi \frac{h}{\gamma_{n}m_{n}l_{k}\langle \lambda_{i}\rangle }\;\text{for}\;k=A,B,C,D.$$

For elastic and small energy transfer quasielastic scattering (where the detailed balance factor is essentially 1 and the scattering function is symmetrical around zero energy transfer), we have 〈*v_i_*〉 = 〈*v_f_*〉. Therefore, the magnitude of the r.f. field is tuned to create *π*-flips for the mean incident velocity 〈*v_i_*〉 for all coils. This implies

(60) $$\left|B_{rf}^{k}\right|=\frac{\pi \langle v_{i}\rangle }{\gamma_{n}l_{k}}=\pi \frac{h}{\gamma_{n}m_{n}l_{k}\langle \lambda_{i}\rangle }\;\text{for}\;k=A,B,C,D.$$

### 3.2 A Note About Signs

In Sec. 3.3 and especially in Sec. 3.5 we must account for reversals of the directions of the static fields *B_0_* from one coil to the next. This is important because the reversed direction of *B_0_* reverses the direction of the Larmor precession and consequently switches the resonant r.f. field component to the counter-rotating component (that has a different absolute phase angle). This latter situation is simplified mathematically (with no loss of generality) if we assume that the r.f. field oscillates along the *x*-axis, since the shift of r.f. phase angle that accompanies the change of sign of *B_0_* amounts only to a flip of the sign of *ψ*. In the following sections, expressions for the phase changes throughout the spectrometer are written in tabular form, initially with signs that account for general static field directions in the coils.

### 3.3 A 4-Single *π*-Coil, “Perfect” Dispersionless NRSE with Zero Stray Fields And Well-Collimated Beams

The 4-single *π* flipper arrangement is illustrated in Fig. 5. We use the operator (Eq. (16)) for the neutron spin phase in the coil regions and assume truly zero field in the gaps between the coils (allowing Eq. (19) to be used). Phase locking of the r.f. frequency between coils *A* and *B* and between *C* and *D* is assumed (allowing expressions of the type (21) to be used), but no phase locking of the r.f. between the two arms of the spectrometer is required (hence *ψ_C_* is unrelated to *ψ_A_*). By assuming well-collimated beams (cos*θ* ≈ 1), we have replaced neutron flight times with expressions of the type *l*/*v_n_* or *L*/*v_n_* where *l* or *L* is a dimension along the beam (*y*) axis. We now construct a table of phases through the spectrometer, applying the above assumptions and signs to account for general static field directions. For example, sgn(*B_0_*) = “+” if the static field lies along +*z* and sgn(*B_0_*) = “−” if the static field lies along −*z*. The result is shown in Table 3.

Applying the signs shown in Fig. 5 (i.e., sgn(*B_0_^A^*) = sgn(*B_0_^B^*) = “+” and sgn(*B_0_^C^*) = sgn(*B_0_^D^*) = “−”), we reconstruct Table 3 as shown in Table 4.

***Observations***

1. The lack of a relation between the r.f. phases in each arm of the spectrometer is immaterial. This is because the r.f. phases, *ψ_A_*, and *ψ_C_*, on entry to coils *A* and *C* cancel on leaving coils *B* and *D* respectively.
2. The final spin phase angle of the neutron exiting coil *D* is independent of the distances between the second coil *B* and the sample (*L_BS_*) and the sample and the third coil *C* (*L_SC_*).
3. The final neutron spin phase from Table 4 is:

(61) $$\begin{array}{l} {\varphi^{\prime }}_{D}=\left[\frac{\omega_{0}(2L_{AB}+l_{A}+l_{B})}{v_{i}}-\frac{\omega_{1}(2L_{CD}+l_{C}+l_{D})}{v_{f}}\right]\\ =\gamma_{n}\left[\frac{B_{0}(2L_{AB}+l_{A}+l_{B})}{v_{i}}-\frac{B_{1}(2L_{CD}+l_{C}+l_{D})}{v_{f}}\right]. \end{array}$$

If all coils are identical in length (i.e., *l_A_* = *l_B_* = *l_C_* = *l_D_* = *l_Β0_ = l_coil_ = l*), then the phase angle of the neutron spin at the exit of coil *D* reduces to

(62) $${\varphi^{\prime }}_{D}=2\left[\frac{\omega_{0}(L_{AB}+l)}{v_{i}}-\frac{\omega_{1}(L_{CD}+l)}{v_{f}}\right]=2\gamma_{n}\left[\frac{B_{0}(L_{AB}+l)}{v_{i}}-\frac{B_{1}(L_{CD}+l)}{v_{f}}\right].$$

Equation (62) is expressed more neatly by introducing *L_0_* and *L_1_*, the distances between the *mid-points* of the coils in the first arm and second arm respectively, where

(63) $$L_{0}=L_{AB}+l$$

and

(64) $$L_{1}=L_{CD}+l$$

so that Eq. (62) becomes

(65) $${\varphi^{\prime }}_{D}=2\left[\frac{\omega_{0}L_{0}}{v_{i}}-\frac{\omega_{1}L_{1}}{v_{f}}\right]=2\gamma_{n}\left[\frac{B_{0}L_{0}}{v_{i}}-\frac{B_{1}L_{1}}{v_{f}}\right].$$

### 3.4 The “Bootstrap” Coil Technique

#### 3.4.1 Bootstrap Configurations

**Fig. 6:**
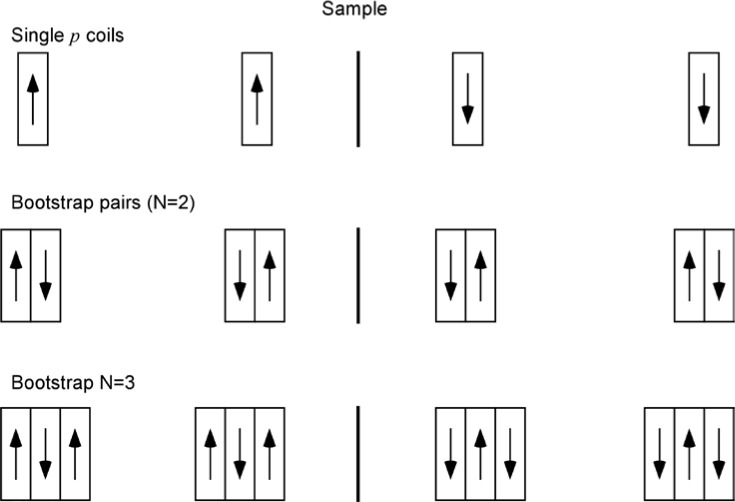
Some possible *π*-flipper spin-echo arrangements.

Gähler and Golub [2] appreciated that spin-echo configurations of resonant *π*-flippers are not limited to single *π*-flipper unit arrangements. Multiple flippers placed back-to-back with alternating static field directions can replace the single *π*-flippers at the zero-field region boundaries. Several 4-coil unit spin-echo arrangements are shown in Fig. 6. When more than one *π*-flipper (*N* > 1) comprises one unit, the combination is referred to as a “bootstrap coil”. The technique was first demonstrated experimentally in Ref. [7]. Note that the static field directions in the second arm mirror-image those in the first arm. Because closed magnetic field loops are produced within the coil unit for even-*N*, leakage fields outside the coil regions are strongly reduced with respect to odd-*N* combinations and it has been demonstrated [8] that the field homogeneity within the beam area is improved for *N* = 2 with respect to *N* = 1. Furthermore, the (small) leakage fields each side of the even-*N* bootstrap coil cancel to first order because the Larmor precession that they induce is approximately equal in magnitude but of opposite sign.

#### 3.4.2 Practical Limits to the Value of *N*

Bootstrap coils with *N* flippers effectively multiply the spin turn by a factor of *N*, thereby increasing the resolution of the spectrometer by the same factor *N*. This is illustrated for *N* = 2 in Sec. 3.5. However, instrumental non-ideality ultimately limits the maximum practical value of *N*.

1. The most obvious limitation is that *N* multiplies the number of coil windings traversed by the neutron beam, multiplying the absorption and scattering by the same factor.
2. The total power dissipation is proportional to *N*, negatively impacting the already challenging task of heat removal from the coils units.
3. The dispersion of the *π*-flippers means that increasing non-zero *z*-components of the spin vectors result as the neutron traverses additional coils. Gähler and Golub show [2] that the expectation values of 〈*σ_x_*〉 and 〈*σ _z_*〉 each contain 2*^N^* terms in sin*^m^*(*µB_rf_ l*/*ħv_n_*) and/or cos*^m^*(*µB_rf_ l*/*ħv_n_*) where *m* runs up to *N* and *v_n_* is the neutron velocity. Because these rapidly-varying functions of velocity lead to depolarization of the beam, Gähler and Golub also show that *Δv_n_/v_n_* must become increasingly small as *N* increases to compensate.

In view of the compromises imposed by 1, 2, and 3, and the advantages of even-*N* for stray field suppression, *N* = 2 is almost universally used in existing NRSE spectrometers.

### 3.5 A 4 “Perfect” Dispersionless *N* = 2 Bootstrap Coil NRSE with Zero Stray Fields and Well-Collimated Beams

In the bootstrap pair, the main consequence of the field direction reversal mid-way across the coil unit is that the resonant component of the r.f. field switches to the counter-rotating component in the second *π*-flipper. This reverses the sign of *ψ* and of *ω_0_*. In order to illustrate features that are likely present in a real bootstrap coil, it is assumed that the transition from one *π*-flipper of the pair to the other takes place across a small gap *l_g_*, which is equal for all coils. This gap is also assumed to be “zero field” (or a region where the stray fields of the adjacent coils exactly cancel). By adopting the procedure outlined in Sec. 3.3 and applying the specific field direction signs indicated in Fig. 6 for *N* = 2, we obtain the values given in Table 5.

***Observations***

1. The lack of a relation between the r.f. phases in each arm of the spectrometer is immaterial.
2. The final spin phase angle of the neutron exiting coil *D_2_* is independent of the distances between the coil *B_2_* and the sample (*L_BS_*) and between the sample and the coil *C_1_* (*L_SC_*).
3. The final neutron spin phase from Table 5 is:

(66) $${\varphi^{\prime }}_{D2}=\gamma_{n}\left[\begin{array}{c} \frac{B_{0}[l_{A1}+l_{B2}+3(l_{A2}+l_{B1})+4(L_{AB}+l_{g})]}{v_{i}}\\ -\frac{B_{1}[l_{C1}+l_{D2}+3(l_{C2}+l_{D1})+4(L_{CD}+l_{g})]}{v_{f}} \end{array}\right].$$

If the individual *π*-flippers are identical in length (i.e., *l_A1_* = *l_A2_* = *l_B1_* = *l_B2_* = *l_C1_* = *l_C2_* = *l_D1_* = *l_D2_* = *l_B0_*) and we consistently use the symbol *l* to define the total length of the bootstrap unit, i.e., *l* = 2*l_B0_* + *l_g_* (including the gap in the middle), then the phase angle of the neutron spin at the exit of coil *D_2_* (Eq. (66)) reduces to

(67) $${\varphi^{\prime }}_{D2}=\varphi_{NRSE}=4\gamma_{n}\left[\frac{B_{0}[L_{AB}+l]}{v_{i}}-\frac{B_{1}[L_{CD}+l]}{v_{f}}\right].$$

By comparing Eq. (67), with the equivalent equation for the single (*N* = 1) *π*-flipper case (Eq. (62)), we see that there is an additional *doubling* of the spin phase angle change by using bootstrap pairs. It can be shown [2] that this additional factor of 2 actually corresponds to *N*, the number of coils in the bootstrap coil unit, therefore we can rewrite Eq. (67) quite generally as

(68) $$\varphi_{NRSE}=2N\gamma_{n}\left[\frac{B_{0}[L_{AB}+l]}{v_{i}}-\frac{B_{1}[L_{CD}+l]}{v_{f}}\right].$$

Finally, Eq. (68) is represented more neatly by introducing *L_0_* and *L_1_*, the distances between the *mid-points* of the bootstrap coil units for the first arm and second arm respectively, so that Eq. (68) becomes

(69) $$\begin{array}{l} \varphi_{NRSE}=2N\gamma_{n}\left[\frac{{Β}_{0}L_{0}}{v_{i}}-\frac{B_{1}L_{1}}{v_{f}}\right]=\frac{2Nm_{n}\gamma_{n}}{h}[B_{0}L_{0}\lambda_{i}-B_{1}L_{1}\lambda_{f}]\\ =92641.8N\left[B_{0}[T]L_{0}[m]\lambda_{i}\left[Å\right]-B_{1}[T]L_{1}[m]\lambda_{f}\left[Å\right]\right]. \end{array}$$

With regard to differences that may exist in the flippers of a real spectrometer, it is interesting to note from Table 5 that the inner *π*-flippers in each arm contribute three times the spin turn of the outer coils whereas the zero-field gaps between the coils of a pair (designated by *l_g_*) contribute at the same rate per unit length as the inter-coil zero-field gaps (see also Eq. (66)).

### 3.6 Coils with Dimensional Uncertainties

In order to analyze the effect of dimensional uncertainties, we assume that the center lines of each *π* coil are fixed and that the coil length (winding flatness on each side of the coil) fluctuates by *Δl_B0_*, according to equal, but independent, Gaussian distributions of width *Δf^FWHM^* = *Δl_B0_^FWHM^*/√2. The zero-field flight paths between the coils are assumed truly zero field so that the neutron spin direction does not change in them.

We define the coil length deviation on the left and right hand sides of the coil as *Δf_L_* and *Δf_R_* respectively, where *Δf* is negative if the coil surface is on the -*y* side of the nominal position (neutron arrives earlier) and positive if on the +*y* side of the nominal position (neutron arrives later). The r.f. phase at the entrance to the first coil varies with respect to the nominal value due to fluctuations in the coil length where

$$\psi_{A}=\psi_{in}+\mathrm{sgn}\;\left(B_{0}^{A}\right)\frac{\omega_{rf}}{v_{n}}\Delta f_{L}(A),$$

where *ψ_in_* is the instantaneous phase of the resonant component of the r.f. field with respect to the neutron spin at the coil entrance in the *perfect* situation. At the coil exit the r.f. phase is

$$\begin{array}{l} {\psi^{\prime }}_{A}=\psi_{A}+\mathrm{sgn}\;\left(B_{0}^{A}\right)\frac{\omega_{rf}}{v_{n}}(l_{B_{0}}-\Delta f_{L}(A)+\Delta f_{R}(A))\\ =\psi_{in}+\mathrm{sgn}\;\left(B_{0}^{A}\right)\frac{\omega_{rf}}{v_{n}}(l_{B_{0}}+\Delta f_{R}(A)) \end{array}$$

therefore, from Eq. (16), the neutron phase at the exit of *π* coil *A* is

$${\varphi^{\prime }}_{A}=2\psi_{in}+\mathrm{sgn}\;(B_{0}^{A})\frac{\omega_{rf}}{v_{n}}\left(l_{B_{0}}+\Delta f_{L}(A)+\Delta f_{R}(A)\right)-\varphi_{in}.$$

On entry to the second coil *B*, we have $\varphi_{B}={\varphi^{\prime }}_{A}$ and

$$\begin{array}{c} \psi_{B}=\mathrm{sgn}\;(B_{0}^{B})\mathrm{sgn}\;(B_{0}^{A})\left[{\psi^{\prime }}_{A}+\mathrm{sgn}\;(B_{0}^{A})\frac{\omega_{rf}}{v_{n}}\left(L_{AB}-\Delta f_{R}(A)+\Delta f_{L}(B)\right)\right]\\ =\mathrm{sgn}\;(B_{0}^{B})\mathrm{sgn}\;(B_{0}^{A})\left[\psi_{in}+\mathrm{sgn}\;(B_{0}^{A})\frac{\omega_{rf}}{v_{n}}\left(L_{AB}+l_{B_{0}}+\Delta f_{L}(B)\right)\right]. \end{array}$$

At the exit of coil *B*, we have

$$\begin{array}{l} {\psi^{\prime }}_{B}=\psi_{B}+\mathrm{sgn}\;(B_{0}^{B})\frac{\omega_{rf}}{v_{n}}\left(l_{B_{0}}-\Delta f_{L}(B)+\Delta f_{R}(B)\right)\\ =\mathrm{sgn}\;(B_{0}^{B})\mathrm{sgn}\;(B_{0}^{A})\left[\psi_{in}+\mathrm{sgn}\;(B_{0}^{A})\frac{\omega_{rf}}{v_{n}}\left(L_{AB}+l_{B_{0}}+\Delta f_{L}(B)\right)\right]\\ +\mathrm{sgn}\;(B_{0}^{B})\frac{\omega_{rf}}{v_{n}}(l_{B_{0}}-\Delta f_{L}(B)+\Delta f_{R}(B)). \end{array}$$

Therefore the neutron phase at the exit of *π* coil *B* is

(70) $$\begin{array}{l} {\varphi^{\prime }}_{B}=2\mathrm{sgn}\;(B_{0}^{B})\mathrm{sgn}\;(B_{0}^{A})\left[\psi_{in}+\mathrm{sgn}\;(B_{0}^{A})\frac{\omega_{rf}}{v_{n}}(L_{AB}+l_{B_{0}}+\Delta f_{L}(B))\right]\\ +\mathrm{sgn}\;(B_{0}^{B})\frac{\omega_{rf}}{v_{n}}(l_{B_{0}}-\Delta f_{L}(B)+\Delta f_{R}(B))-\mathrm{sgn}\;(B_{0}^{A})\frac{\omega_{rf}}{v_{n}}\left(l_{B_{0}}+\Delta f_{L}(A)+\Delta f_{R}(A)\right)\\ -2\psi_{in}+\varphi_{in}. \end{array}$$

At the entrance to a third coil *C*, we have $\varphi_{C}={\varphi^{\prime }}_{B}$. Assuming that the r.f. in coil *C* is phase locked to coil *B* (we do this so that this 4 *π*-flipper coil argument can be extended to an *N* = 2, 4 *π*-flipper *per spectrometer arm* arrangement in Sec. 3.6.2)

$$\begin{array}{l} \psi_{C}=\mathrm{sgn}\;(B_{0}^{C})\mathrm{sgn}\;(B_{0}^{B})\left[{\psi^{\prime }}_{B}+\mathrm{sgn}\;(B_{0}^{B})\frac{\omega_{rf}}{v_{n}}(L_{BC}-\Delta f_{R}(B)+\Delta f_{L}(C))\right]\\ =\mathrm{sgn}\;(B_{0}^{C})\mathrm{sgn}\;(B_{0}^{B})\left[\begin{array}{l} \mathrm{sgn}\;(B_{0}^{B})\mathrm{sgn}\;(B_{0}^{A})\left[\psi_{in}+\mathrm{sgn}\;(B_{0}^{A})\frac{\omega_{rf}}{v_{n}}\left(L_{AB}+l_{B_{0}}+\Delta f_{L}(B)\right)\right]\\ +\mathrm{sgn}\;(B_{0}^{B})\frac{\omega_{rf}}{v_{n}}\left(L_{BC}+l_{B_{0}}+\Delta f_{L}(C)-\Delta f_{L}(B)\right) \end{array}\right]. \end{array}$$

At the exit of coil *C*, we have

$$\begin{array}{l} {\psi^{\prime }}_{C}=\psi_{C}+\mathrm{sgn}\;(B_{0}^{C})\frac{\omega_{rf}}{v_{n}}(l_{B_{0}}-\Delta f_{L}(C)+\Delta f_{R}(C))\\ =\mathrm{sgn}\;(B_{0}^{C})\mathrm{sgn}\;(B_{0}^{B})\left[\begin{array}{l} \mathrm{sgn}\;(B_{0}^{B})\mathrm{sgn}\;(B_{0}^{A})\left[\psi_{in}+\mathrm{sgn}\;(B_{0}^{A})\frac{\omega_{rf}}{v_{n}}\left(L_{AB}+l_{B_{0}}+\Delta f_{L}(B)\right)\right]\\ +\mathrm{sgn}\;(B_{0}^{B})\frac{\omega_{rf}}{v_{n}}\left(L_{BC}+l_{B_{0}}+\Delta f_{L}(C)-\Delta f_{L}(B)\right) \end{array}\right]\\ +\mathrm{sgn}\;(B_{0}^{C})\frac{\omega_{rf}}{v_{n}}\left(l_{B_{0}}-\Delta f_{L}(C)+\Delta f_{R}(C)\right) \end{array}$$

so that

$$\begin{array}{l} {\varphi^{\prime }}_{C}=2\mathrm{sgn}\;(B_{0}^{C})\mathrm{sgn}\;(B_{0}^{B})\left[\begin{array}{l} \mathrm{sgn}\;(B_{0}^{B})\mathrm{sgn}\;(B_{0}^{A})\left[\psi_{in}+\mathrm{sgn}\;(B_{0}^{A})\frac{\omega_{rf}}{v_{n}}\left(L_{AB}+l_{B_{0}}+\Delta f_{L}(B)\right)\right]\\ +\mathrm{sgn}\;(B_{0}^{B})\frac{\omega_{rf}}{v_{n}}\left(L_{BC}+l_{B_{0}}+\Delta f_{L}(C)-\Delta f_{L}(B)\right) \end{array}\right]\\ -2\mathrm{sgn}\;(B_{0}^{B})\mathrm{sgn}\;(B_{0}^{A})\left[\psi_{in}+\mathrm{sgn}\;(B_{0}^{A})\frac{\omega_{rf}}{v_{n}}\left(L_{AB}+l_{B_{0}}+\Delta f_{L}(B)\right)\right]\\ +\mathrm{sgn}\;(B_{0}^{C})\frac{\omega_{rf}}{v_{n}}\left(l_{B_{0}}-\Delta f_{L}(C)+\Delta f_{R}(C)\right)-\mathrm{sgn}\;(B_{0}^{B})\frac{\omega_{rf}}{v_{n}}\left(l_{B_{0}}-\Delta f_{L}(B)+\Delta f_{R}(B)\right)\\ +\mathrm{sgn}\;(B_{0}^{A})\frac{\omega_{rf}}{v_{n}}\left(l_{B_{0}}+\Delta f_{L}(A)+\Delta f_{R}(A)\right)+2\psi_{in}-\varphi_{in}. \end{array}$$

At the entrance to the 4^th^ coil *D* $\varphi_{D}={\varphi^{\prime }}_{C}$ and

$$\begin{array}{l} \psi_{D}=\mathrm{sgn}\;(B_{0}^{D})\mathrm{sgn}\;(B_{0}^{C})\left[{\psi^{\prime }}_{C}+\mathrm{sgn}\;(B_{0}^{C})\frac{\omega_{rf}}{v_{n}}\left(L_{CD}-\Delta f_{R}(C)+\Delta f_{L}(D)\right)\right]\\ =\mathrm{sgn}\;\left(B_{0}^{D}\right)\mathrm{sgn}\;\left(B_{0}^{C}\right)\times \\ \left[\begin{array}{l} \mathrm{sgn}\;\left(B_{0}^{C}\right)\mathrm{sgn}\;(B_{0}^{B})\left[\begin{array}{l} \mathrm{sgn}\;\left(B_{0}^{B}\right)\mathrm{sgn}\;\left(B_{0}^{A}\right)\left[\psi_{in}+\mathrm{sgn}\;\left(B_{0}^{A}\right)\frac{\omega_{rf}}{v_{n}}\left(L_{AB}+l_{B_{0}}+\Delta f_{L}(B)\right)\right]\\ +\mathrm{sgn}\;\left(B_{0}^{B}\right)\frac{\omega_{rf}}{v_{n}}\left(L_{BC}+l_{B_{0}}+\Delta f_{L}(C)-\Delta f_{L}(B)\right) \end{array}\right]\\ +\mathrm{sgn}\;\left(B_{0}^{C}\right)\frac{\omega_{rf}}{v_{n}}\left(L_{CD}+l_{B_{0}}+\Delta f_{L}(D)-\Delta f_{L}(C)\right) \end{array}\right] \end{array}$$

At the exit of coil *D*, we have

$$\begin{array}{l} {\psi^{\prime }}_{D}=\psi_{D}+\mathrm{sgn}\;(B_{0}^{D})\frac{\omega_{rf}}{v_{n}}\left(l_{B_{0}}-\Delta f_{L}(D)+\Delta f_{R}(D)\right)\\ =\mathrm{sgn}\;\left(B_{0}^{D}\right)\mathrm{sgn}\;\left(B_{0}^{C}\right)\times \\ \left[\begin{array}{l} \mathrm{sgn}\;\left(B_{0}^{C}\right)\mathrm{sgn}\;(B_{0}^{B})\left[\begin{array}{l} \mathrm{sgn}\;\left(B_{0}^{B}\right)\mathrm{sgn}\;\left(B_{0}^{A}\right)\left[\psi_{in}+\mathrm{sgn}\;\left(B_{0}^{A}\right)\frac{\omega_{rf}}{v_{n}}\left(L_{AB}+l_{B_{0}}+\Delta f_{L}(B)\right)\right]\\ +\mathrm{sgn}\;\left(B_{0}^{B}\right)\frac{\omega_{rf}}{v_{n}}\left(L_{BC}+l_{B_{0}}+\Delta f_{L}(C)-\Delta f_{L}(B)\right) \end{array}\right]\\ +\mathrm{sgn}\;\left(B_{0}^{C}\right)\frac{\omega_{rf}}{v_{n}}\left(L_{CD}+l_{B_{0}}+\Delta f_{L}(D)-\Delta f_{L}(C)\right) \end{array}\right]\\ +\mathrm{sgn}\;\left(B_{0}^{D}\right)\frac{\omega_{rf}}{v_{n}}\left(l_{B_{0}}-\Delta f_{L}(D)+\Delta f_{R}\left(D\right)\right) \end{array}$$

so that the neutron phase at the exit of the 4th coil is

(71) $$\begin{array}{l} {\varphi^{\prime }}_{D}=2\mathrm{sgn}\;\left(B_{0}^{D}\right)\mathrm{sgn}\;\left(B_{0}^{C}\right)\left[\begin{array}{l} \mathrm{sgn}\;\left(B_{0}^{C}\right)\mathrm{sgn}\;\left(B_{0}^{B}\right)\times \\ \left[\begin{array}{l} \mathrm{sgn}\;\left(B_{0}^{B}\right)\mathrm{sgn}\;\left(B_{0}^{A}\right)\left[\psi_{in}+\mathrm{sgn}\;\left(B_{0}^{A}\right)\frac{\omega_{rf}}{v_{n}}\left(L_{AB}+l_{B_{0}}+\Delta f_{L}(B)\right)\right]\\ +\mathrm{sgn}\;\left(B_{0}^{B}\right)\frac{\omega_{rf}}{v_{n}}\left(L_{BC}+l_{B_{0}}+\Delta f_{L}(C)-\Delta f_{L}(B)\right) \end{array}\right]\\ +\mathrm{sgn}\;\left(B_{0}^{C}\right)\frac{\omega_{rf}}{v_{n}}\left(L_{CD}+l_{B_{0}}+\Delta f_{L}(D)-\Delta f_{L}(C)\right) \end{array}\right]\\ -2\mathrm{sgn}\;\left(B_{0}^{C}\right)\mathrm{sgn}\;\left(B_{0}^{B}\right)\left[\begin{array}{l} \mathrm{sgn}\;\left(B_{0}^{B}\right)\mathrm{sgn}\;\left(B_{0}^{A}\right)\left[\psi_{in}+\mathrm{sgn}\;\left(B_{0}^{A}\right)\frac{\omega_{rf}}{v_{n}}\left(L_{AB}+l_{B_{0}}+\Delta f_{L}(B)\right)\right]\\ +\mathrm{sgn}\;\left(B_{0}^{B}\right)\frac{\omega_{rf}}{v_{n}}\left(L_{BC}+l_{B_{0}}+\Delta f_{L}(C)-\Delta f_{L}(B)\right) \end{array}\right]\\ +2\mathrm{sgn}\;\left(B_{0}^{B}\right)\mathrm{sgn}\;\left(B_{0}^{A}\right)\left[\psi_{in}+\mathrm{sgn}\;\left(B_{0}^{A}\right)\frac{\omega_{rf}}{v_{n}}\left(L_{AB}+l_{B_{0}}+\Delta f_{L}(B)\right)\right]\\ +\mathrm{sgn}\;\left(B_{0}^{D}\right)\frac{\omega_{rf}}{v_{n}}\left(l_{B_{0}}-\Delta f_{L}(D)+\Delta f_{R}\left(D\right)\right)-\mathrm{sgn}\;\left(B_{0}^{C}\right)\frac{\omega_{rf}}{v_{n}}\left(l_{B_{0}}-\Delta f_{L}(C)+\Delta f_{R}(C)\right)\\ +\mathrm{sgn}\;\left(B_{0}^{B}\right)\frac{\omega_{rf}}{v_{n}}\left(l_{B_{0}}-\Delta f_{L}(B)+\Delta f_{R}(B)\right)-\mathrm{sgn}\;\left(B_{0}^{A}\right)\frac{\omega_{rf}}{v_{n}}\left(l_{B_{0}}+\Delta f_{L}(A)+\Delta f_{R}(A)\right)-2\psi_{in}+\varphi_{in}. \end{array}$$

We now consider two cases.

#### 3.6.1 First Arm of a 4-*N*=1 *π*-Coil NRSE

In this case, the net spin turn in the first arm of the spectrometer in the absence of stray fields is given by Eq. (70) with sgn(*B_0_^A^*) = sgn(*B_0_^B^*) = “+” so that

(72) $$\begin{array}{l} {\varphi^{\prime }}_{B}=2\frac{\omega_{rf}}{v_{n}}\left(L_{AB}+l_{B_{0}}\right)+\frac{\omega_{rf}}{v_{n}}[\Delta f_{L}(B)+\Delta f_{R}(B)-\Delta f_{L}(A)-\Delta f_{R}(A)]+\varphi_{in}\\ =\frac{\omega_{rf}}{v_{n}}[2L_{0}+\Delta f_{L}(B)+\Delta f_{R}(B)-\Delta f_{L}(A)-\Delta f_{R}(A)]+\varphi_{in}. \end{array}$$

#### 3.6.2 First arm of a 4-*N*=2 *π*-Coil NRSE

In this case, the net spin turn in the first arm of the spectrometer in the absence of stray fields is given by Eq. (71) where, for consistency with previous notation used in Sec. 3.5, we interpret “*L_AB_*” as the nominal gap *l_g_* between the bootstrap coil pair, “*L_BC_*” as “*L_AB_*” and “*L_CD_*” as *l_g_* of the second coil pair. We also change the notation *A*→*A_1_*, *B*→*A_2_* etc. for consistency with notation in the previous discussion of bootstrap coils (Sec. 3.5). In this case we have sgn(*B_0_^A1^*) = sgn(*B_0_^B2^*) = “+” and sgn(*B_0_^A2^*) = sgn(*B_0_^B1^*) = “−” and Eq. (71) becomes

(73) $$\begin{array}{l} {\varphi^{\prime }}_{B_{2}}=\frac{\omega_{rf}}{v_{n}}\left(\begin{array}{l} 4L_{AB}+4l_{g}+8l_{B_{0}}-\Delta f_{L}(A_{1})-\Delta f_{R}(A_{1})-\Delta f_{L}(A_{2})-\Delta f_{R}(A_{2})\\ +\Delta f_{L}(B_{1})+\Delta f_{R}(B_{1})+\Delta f_{L}(B_{2})+\Delta f_{R}(B_{2}) \end{array}\right)+\varphi_{in}\\ =\frac{\omega_{rf}}{v_{n}}\left(\begin{array}{l} 4L_{0}-\Delta f_{L}(A_{1})-\Delta f_{R}(A_{1})-\Delta f_{L}(A_{2})-\Delta f_{R}(A_{2})\\ +\Delta f_{L}(B_{1})+\Delta f_{R}(B_{1})+\Delta f_{L}(B_{2})+\Delta f_{R}(B_{2}) \end{array}\right)+\varphi_{in}. \end{array}$$

## 4. Quantum Mechanical Description of NRSE

Gähler, Golub, and Keller [8] have discussed particle beam magnetic resonance in quantum mechanical terms and derived formulas for spin ½ particles passing along the *y*-axis. They show that for neutron magnetic interaction energies that are very much smaller than the neutron kinetic energy entering the field region (*µ B_0_* ≪ ½*m_n_v_i_*^2^ - where reflected matter waves at field boundaries may be neglected), the quantum mechanical treatment reproduces the classical results with wave interpretations of physical processes such as Larmor precession.

### 4.1 Polarized Beam Traversing a Static Field

A spin *s* = ½ particle such as the neutron has spin angular momentum of magnitude $\sqrt{s(s+1)}\hbar =\sqrt{3}\hbar /2$ with a component measured along any given axis of magnitude *m_s_ħ*, where *m_s_* = ± ½. Consider a beam initially polarized along the *x* direction traveling along *y*. The wave function is written as a plane wave (which may be considered as the superposition of equally probable spin-up and spin-down states with respect to the *z*-axis)

(74) $$\psi_{i}=\frac{1}{\sqrt{2}}\left(\left[\begin{array}{l} 1\\ 0 \end{array}\right]+\left[\begin{array}{l} 0\\ 1 \end{array}\right]\right)e^{i(k_{i}y-\omega_{i}t)}=\frac{1}{\sqrt{2}}\left[\begin{array}{l} 1\\ 1 \end{array}\right]e^{i(k_{i}y-\omega_{i}t)}.$$

When the neutron enters a static magnetic field applied along the *z*-direction, the field gradient at the boundary and the associated magnetic force causes the kinetic energy of the ±*σ_z_* spin states to split by an amount ±*µ*_n_*B_0_* with the opposite splitting of the orientational potential energy. Inside the field, where there is no field gradient, the kinetic energies of the ±*σ_z_* spin states differ. If the total splitting is expressed as

(75) $$\Delta E_{B_{0}}=2\mu_{n}B_{0}=\hbar \omega_{0},$$

the wave function inside the static field region is

(76) $$\psi_{B_{0}}=\frac{1}{\sqrt{2}}\left[\begin{array}{l} e^{-i\mu_{n}B_{0}y/\hbar v_{i}}\\ e^{i\mu_{n}B_{0}y/\hbar v_{i}} \end{array}\right]e^{i(k_{i}y-\omega_{i}t)}=\frac{1}{\sqrt{2}}\left[\begin{array}{l} e^{-i\omega_{0}y/2v_{i}}\\ e^{i\omega_{0}y/2v_{i}} \end{array}\right]e^{i(k_{i}y-\omega_{i}t)},$$

where *ω_0_* is interpreted as the classical Larmor precession frequency. The expectation value of the polarization along *x* inside the field region is

(77) $$\begin{array}{l} \langle \sigma_{x}\rangle =\psi_{B_{0}}^{{}^{*}}[\sigma_{x}]\psi_{B_{0}}=\frac{1}{\sqrt{2}}\left[\begin{array}{cc} e^{i\omega_{0}y/2v_{i}} & e^{-i\omega_{0}y/2v_{i}} \end{array}\right]e^{-i(k_{i}y-\omega_{i}t)}\left[\begin{array}{ll} 0 & 1\\ 1 & 0 \end{array}\right]\frac{1}{\sqrt{2}}\left[\begin{array}{l} e^{-i\omega_{0}y/2v_{i}}\\ e^{i\omega_{0}y/2v_{i}} \end{array}\right]e^{i(k_{i}y-\omega_{i}t)}\\ =\frac{1}{2}\left[e^{i\omega_{0}y/v_{i}}+e^{-i\omega_{0}y/v_{i}}\right]=\cos(\omega_{0}y/v_{i}). \end{array}$$

Here, the relative phase of the spin-up and down waves is a cosine function of the distance traveled through the field. This is exactly equivalent to Larmor precession in the classical case.

**Fig. 7:**
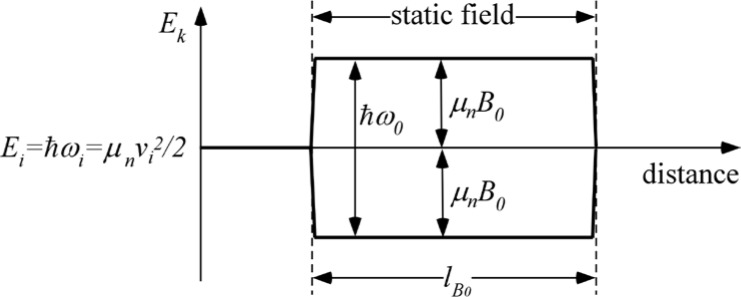
When a neutron, of initial energy *E_i_* = *ħω_i_*, enters a constant static magnetic field region, the field gradient at the boundary causes the spin states that are parallel and antiparallel to the field direction to be split symmetrically by ±*µ_n_B_0_* with respect to *E_i_*. Inside the field, the total (kinetic + potential) energy remains fixed at *ħω_i_*. The total splitting is *ħω_0_*, where *ω_0_* is the classical Larmor angular frequency. Usually *ħω_0_* « *ħω_i_*, such that reflections at the field boundaries can be ignored. On exiting the coil, the degeneracy of the two states is re-established, but the relative phase of the matter waves associated with the + and − states is shifted by an amount *ω_0_ l_B0_*/*v_i_*, corresponding to classical Larmor precession of the spin around the field direction during its passage through the field.

On exiting the field, the *±*σ_z_ spin states once again become degenerate and the neutron spin states retain the accumulated relative phase angle *ω_0_l_B0_/v_i_* with which they exited the field region. This phase angle does not change in the subsequent zero-field region, which is classically equivalent to the termination of the Larmor precession in the zero field region. This situation is illustrated in Fig. 7. Further accounts of these energy changes are found in Refs. [9–13].

Equation (75) is sometimes expressed in terms of a wavevector magnitude splitting of the two states. For NRSE applications it is always true that *ΔE_B0_* ≪ *ħω_i_*, the incident neutron energy, therefore we can write

(78) $$\Delta k\approx \frac{2m_{n}\mu_{n}}{\hbar^{2}}\frac{B_{0}}{k_{i}}$$

and the accumulated phase difference is *l_B0_Δk*.

### 4.2 Passage Through a Static Field with Superimposed Perpendicular Oscillatory Field

With a superimposed perpendicular oscillating field (see Krüger [14]), tuned such that *ω_0_* ≈ *ω_rf_*, transitions between the Zeeman split states (separated by *ΔE = ħω_0_*) are induced via exchange of quanta with the r.f. field. Golub, Gähler, and Keller [13] treat a general case consisting of three regions; two static field regions (I and III) sandwiching an intermediate region (II) were the static field coexists with a perpendicular oscillating field of length *d* along the beam direction. These authors use the properties of Eq. (74) to simplify the problem by treating the +*z* and −*z* components of the eigenvector separately. Further, they assume *ω_rf_* ≪ *ω_i_* (equivalent to the *µB_0_* ≪ ½*m_n_v_i_*^2^ assumption above) where ½*m_n_v_i_*^2^ = *ħω_i_* (whereby reflected matter waves at the potential boundary can be ignored and various simplifying approximations e.g. *δk_B0_* ≈ *ω_0_*/*v_i_* etc. can be made outside of the exponentials). Using the symbols $T_{0,1}^{\pm }$ for the transmission amplitude with subscript “0” for elastic (no exchange of quanta – i.e., no spin flip) and “1” for inelastic (exchange of quanta with spin flip) and “+” and “−” for spin-up and spin-down *final* spin states respectively with respect to *z*, it can be shown:

(79) $$\begin{array}{l} T_{0}^{+}≃\alpha^{+}e^{-i\frac{\varepsilon d}{v}}\left[\cos\left(\frac{\omega_{A}d}{v}\right)+i\frac{\varepsilon }{\omega_{A}}\sin\left(\frac{\omega_{A}d}{v}\right)\right]\equiv T_{↑↑}\\ T_{1}^{+}≃-i\alpha^{-}\frac{\omega_{p}}{2\omega_{A}}e^{-i\frac{\varepsilon d}{v}}\sin\left(\frac{\omega_{A}d}{v}\right)\equiv T_{↓↑}\\ T_{0}^{{}^{-}}≃\alpha^{-}e^{i\frac{\varepsilon d}{v}}\left[\cos\left(\frac{\omega_{A}d}{v}\right)-i\frac{\varepsilon }{\omega_{A}}\sin\left(\frac{\omega_{A}d}{v}\right)\right]\equiv T_{↓↓}\\ T_{1}^{{}^{-}}≃-i\alpha^{+}\frac{\omega_{p}}{2\omega_{A}}e^{i\frac{\varepsilon d}{v}}\sin\left(\frac{\omega_{A}d}{v}\right)\equiv T_{↑↓} \end{array}$$

where *α*_+_ and *α*_−_ are the amplitudes of the *incoming* + and − spin states respectively and

(80) $$\omega_{A}=\sqrt{\frac{\omega_{p}^{2}}{4}+\varepsilon^{2}}=\frac{1}{2}\sqrt{\omega_{p}^{2}+{\left(\omega_{rf}-\omega_{0}\right)}^{2}}$$

with the parameter *ε* proportional to the resonance detuning, defined by

(81) $$\varepsilon =\frac{1}{2}(\omega_{rf}-\omega_{0}),$$

where *ω_p_* =*γ_n_B_rf_* is the classical Larmor precession frequency of the neutron spin around *B_rf_* (Eq. (11)). Substituting the values from Eq. (79) into Eq. (318) of Ref. [13], the wave function in region III becomes

(82) $$\begin{array}{l} \psi_{III}=\left[\begin{array}{l} \psi_{III}^{+}\\ \psi_{III}^{{}^{-}} \end{array}\right]\\ =\left[\begin{array}{l} \left[\cos\left(\frac{\omega_{A}d}{v}\right)+i\frac{\varepsilon }{\omega_{A}}\sin\left(\frac{\omega_{A}d}{v}\right)\right]e^{-\left(\frac{\varepsilon d+\omega_{0}y/2}{v}\right)}\;-i\frac{\omega_{p}}{2\omega_{A}}\sin\left(\frac{\omega_{A}d}{v}\right)e^{-i\left(\frac{\varepsilon d+\left(\omega_{0}/2-\omega_{rf}\right)y}{v}\right)}e^{i\omega_{rf}t}\\ -i\frac{\omega_{p}}{2\omega_{A}}\sin\left(\frac{\omega_{A}d}{v}\right)e^{i\left(\frac{\varepsilon d+(\omega_{0}/2-\omega_{rf})y}{v}\right)}e^{i\omega_{rf}t}\;\left[\cos\left(\frac{\omega_{A}d}{v}\right)-i\frac{\varepsilon }{\omega_{A}}\sin\left(\frac{\omega_{A}d}{v}\right)\right]e^{i\left(\frac{\varepsilon d+\omega_{0}y/2}{v}\right)} \end{array}\right]\left[\begin{array}{l} \alpha_{+}\\ \alpha_{-} \end{array}\right]e^{i(k_{i}y-\omega_{i}t)}. \end{array}$$

The probability of a spin-flip involving a photon exchange with the r.f. field is

(83) $$\begin{array}{l} {\left|T_{1}^{{}^{-}}\right|}^{2}(1)={\left|T_{1}^{+}\right|}^{2}(2)={\left|-i\frac{\omega_{p}}{2\omega_{A}}e^{i\varepsilon \frac{d}{v_{i}}}\sin\left(\omega_{A}\frac{d}{v_{i}}\right)\right|}^{2}\\ =\frac{\omega_{p}^{2}}{\omega_{p}^{2}+{\left(\omega_{rf}-\omega_{0}\right)}^{2}}\sin^{2}\left(\frac{\sqrt{\omega_{p}^{2}+{\left(\omega_{rf}-\omega_{0}\right)}^{2}d}}{2v_{i}}\right), \end{array}$$

which is equivalent to the expression given by Rabi, Ramsey, and Schwinger for the spin flip probability (Eq. (17) of Ref. [15]). Correspondingly, the non-spin flip probability (where the energy does not change) is

(84) $$\begin{array}{l} {\left|T_{0}^{+}\right|}^{2}(1)={\left|T_{0}^{{}^{-}}\right|}^{2}(2)={\left|e^{-i\varepsilon \frac{d}{v_{i}}}\left(\cos\left(\omega_{A}\frac{d}{v_{i}}\right)+i\frac{\varepsilon }{\omega_{A}}\sin\left(\omega_{A}\frac{d}{v_{i}}\right)\right)\right|}^{2}\\ =1-\frac{\omega_{p}^{2}}{\omega_{p}^{2}+{\left(\omega_{rf}-\omega_{0}\right)}^{2}}\sin^{2}\left(\frac{\sqrt{\omega_{p}^{2}+{\left(\omega_{rf}-\omega_{0}\right)}^{2}d}}{2v_{i}}\right), \end{array}$$

which is equal to (1-spin flip probability), as expected.

#### 4.2.1 Special Cases

##### 4.2.1.1 Exact resonance (*ω_0_ = ω_rf_*) − general case

This is the condition for which *ω_rf_* = *ω_0_*, therefore *ε* = 0, *ω_A_* = *ω_p_*/2, consequently

(85) $$\psi_{III}=\frac{1}{\sqrt{2}}\left[\begin{array}{l} \cos\left(\frac{\omega_{p}d}{2v_{i}}\right)e^{-i\omega_{0}\frac{y}{2v_{i}}}-i\sin\left(\frac{\omega_{p}d}{2v_{i}}\right)e^{i\omega_{0}\left(\frac{y}{2v_{i}}-t\right)}\\ -i\sin\left(\frac{\omega_{p}d}{2v_{i}}\right)e^{-i\omega_{0}\left(\frac{y}{2v_{i}}-t\right)}+\cos\left(\frac{\omega_{p}d}{2v_{i}}\right)e^{i\omega_{0}\frac{y}{2v_{i}}} \end{array}\right]e^{i(k_{i}y-\omega_{i}t)}$$

and the probability of a spin-flip involving a photon exchange with the r.f. field is

(86) $${\left|T_{1}^{{}^{-}}\right|}^{2}(1)={\left|T_{1}^{+}\right|}^{2}(2)=\sin^{2}\left(\frac{\omega_{p}d}{2v_{i}}\right)$$

and the non-spin flip probability is

(87) $${\left|T_{0}^{+}\right|}^{2}(1)={\left|T_{0}^{{}^{-}}\right|}^{2}(2)=\cos^{2}\left(\frac{\omega_{p}d}{2v_{i}}\right).$$

We see that unless *ω_p_d*/*v_i_* = (2*N*+1)*π*, where *N* is an integer, an incomplete spin inversion occurs (corresponding to a flipper efficiency < 1); i.e., r.f. *π*-flippers only produce exact *π*-flips for a unique velocity (as was shown classically in Sec. 2.2).

##### 4.2.1.2 Exact resonance (*ω_0_ = ω_rf_*), with dispersion, flipper tuned optimally for *v_i_* = 〈*v_i_*〉

If, additionally, the flipper is tuned optimally for the average velocity 〈*v_i_*〉, we have

(88) $$\omega_{p}\frac{d}{\langle v_{i}\rangle }=\pi .$$

Thus, Eqs. (86) and (87) may be re-expressed as

(89) $${\left|T_{1}^{{}^{-}}\right|}^{2}(1)={\left|T_{1}^{+}\right|}^{2}(2)=\sin^{2}\left(\frac{\pi }{2}\frac{\langle v_{i}\rangle }{v_{i}}\right)=\sin^{2}\left(\frac{\pi }{2}\frac{\lambda_{i}}{\langle \lambda_{i}\rangle }\right)\;\;P(\text{sf})\;\text{tuned for exact}\;\pi \text{-flips for}\;\lambda_{i}=\langle \lambda_{i}\rangle$$

and

(90) $${\left|T_{0}^{+}\right|}^{2}(1)={\left|T_{0}^{{}^{-}}\right|}^{2}(2)=\cos^{2}\left(\frac{\pi }{2}\frac{\langle v_{i}\rangle }{v_{i}}\right)=\cos^{2}\left(\frac{\pi }{2}\frac{\lambda_{i}}{\langle \lambda_{i}\rangle }\right)\;\;P(\text{nsf})\;\text{tuned for exact}\;\pi \text{-flips for}\;\lambda_{i}=\langle \lambda_{i}\rangle ,$$

respectively. *Note that the quantum mechanical spin-flip probability for a neutron of wavelength λ_i_* (*Eq.* (*89*)) *is exactly equivalent to the quantity* $\frac{P_{disp}}{P_{ideal}}=\langle \cos\;\varepsilon \cos\;\chi \rangle$ *derived from the classical treatment* (*see Sec. 2.2 and* Eq. (30)).

##### 4.2.1.3 Exact resonance (*ω_0_ = ω_rf_*), exact *π*-flips for all velocities (i.e., no dispersion or monochromatic)

“No-dispersion” implies that the classical condition for exact *π*-flips around *B_rf_* is satisfied for *all* velocities, i.e., we replace *ω_p_d*/*v_i_* by *π* for any *v_i_* in Eq. (85), which becomes

(91) $$\psi_{III}=\frac{-i}{\sqrt{2}}\left[\begin{array}{l} e^{i\omega_{0}\left(\frac{y}{2v_{i}}-t\right)}\\ e^{-i\omega_{0}\left(\frac{y}{2v_{i}}-t\right)} \end{array}\right]e^{i(k_{i}y-\omega_{i}t)}$$

and the probability of a spin-flip involving a photon exchange with the r.f. field is

(92) $${\left|T_{1}^{{}^{-}}\right|}^{2}(1)={\left|T_{1}^{+}\right|}^{2}(2)=1$$

and the non-spin flip probability (where the energy stays the same) is

(93) $${\left|T_{0}^{+}\right|}^{2}(1)={\left|T_{0}^{{}^{-}}\right|}^{2}(2)=0.$$

Note that under these conditions exact spin inversion occurred in region II.

Region II is defined from 0 ≤ *y* ≤ *d* so that shifting the coordinate system to the exit of region II, we have

(94) $$\psi_{III}=\frac{-i}{\sqrt{2}}\left[\begin{array}{l} e^{i\omega_{0}\left(\frac{y-d}{2v_{i}}-t\right)}\\ e^{-i\omega_{0}\left(\frac{y-d}{2v_{i}}-t\right)} \end{array}\right]e^{i(k_{i}y-\omega_{i}t)}.$$

The expectation value of *σ_x_* at the exit of the coil is

(95) $$\begin{array}{l} \langle \sigma_{x}\rangle =\psi_{III}^{{}^{*}}\left[\begin{array}{ll} 0 & 1\\ 1 & 0 \end{array}\right]\psi_{III}=\frac{1}{2}\left[\begin{array}{cc} e^{-i\omega_{0}\left(\frac{y-d}{2v_{i}}-t\right)} & e^{i\omega_{0}\left(\frac{y-d}{2v_{i}}-t\right)} \end{array}\right]e^{-i(k_{i}y-\omega_{i}t)}\left[\begin{array}{ll} 0 & 1\\ 1 & 0 \end{array}\right]\left[\begin{array}{l} e^{i\omega_{0}\left(\frac{y-d}{2v_{i}}-t\right)}\\ e^{-i\omega_{0}\left(\frac{y-d}{2v_{i}}-t\right)} \end{array}\right]e^{i(k_{i}y-\omega_{i}t)}\\ =\frac{1}{2}\left[e^{-i2\omega_{0}\left(\frac{y-d}{2v_{i}}-t\right)}+e^{i2\omega_{0}\left(\frac{y-d}{2v_{i}}-t\right)}\right]=\cos\left(2\omega_{0}\left(\frac{y-d}{2v_{i}}-t\right)\right)\equiv \cos\left(2\omega_{rf}\left(\frac{y-d}{2v_{i}}-t\right)\right). \end{array}$$

Thus, *at a fixed position y*, the spin precesses at angular frequency *2ω_0_.* Because the superimposed r.f. field induces a spin state inversion in the resonant coil, the kinetic energy splitting of ±*ħω_0_*/2 induced by the static field gradient at the entrance to the coil *adds* to the splitting produced by the opposite field gradient at the coil exit (rather than canceling as it would with a static field alone). Thus the two spin states emerge from the coil with frequencies split by ±*ω_0_* = 2*ω_0_* (≡ 2*ω_rf_* at resonance) with correspondingly different momenta. The beating due to this difference in *ω* and *k* of the two spin states after exiting the coil corresponds to a Larmor precession seen at a fixed position *y*. This precession in a zero field has been observed directly in a MIEZE (“Modulation of Intensity Emerging from Zero Effort!”) setup [16].

The argument of Eq. (95) equates with the spin phase angle of a neutron exiting coil *A* in Table 4, where the neutron spin was initially polarized along *x*, and where exact resonance (*ω_0_* = *ω_rf_*) and zero dispersion were also assumed, i.e.,

(96) $$\varphi_{A}^{\prime }=2\psi_{A}+\frac{1}{v_{i}}\gamma_{n}\left|B_{0}\right|l_{A}=2\psi_{A}+\frac{1}{v_{i}}\omega_{0}l_{A}=2\psi_{A}+\frac{1}{v_{i}}\omega_{rf}l_{A}\;\text{from Table}\;4$$

with the argument in Eq. (95) evaluated at *y* = 0 (the coil exit)

(97) $$\varphi_{A}^{\prime }=2\omega_{rf}\left(\frac{d}{2v_{i}}-t\right).$$

By identifying *ψ_A_* with *ω_rf_t* and *d* with *l_A_*, we see that the two equations are equivalent.

**Fig. 8:**
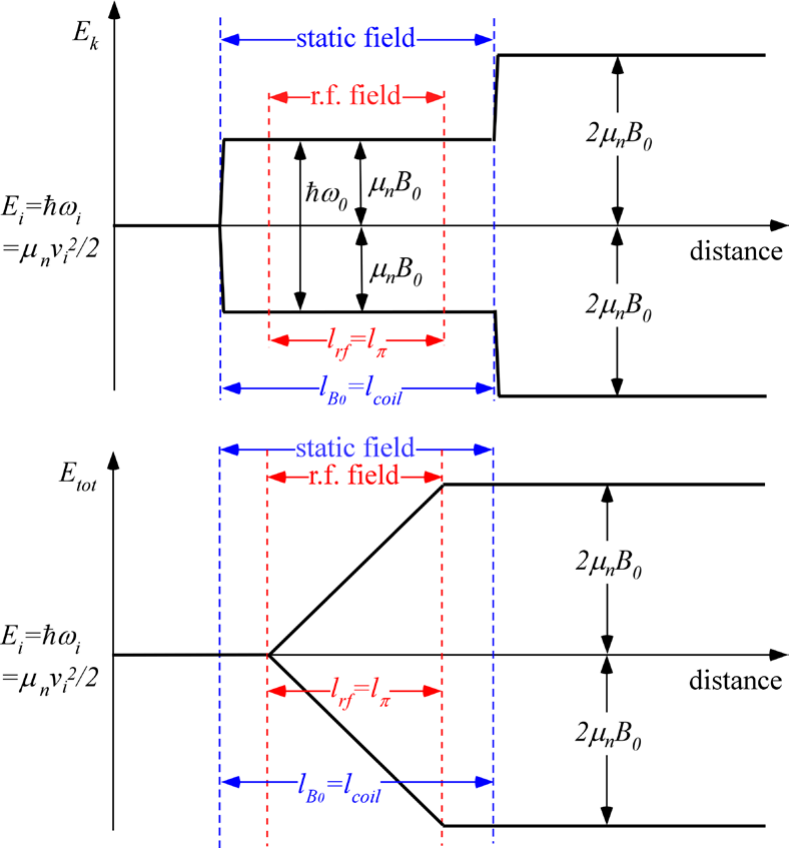
An r.f. flipper tuned for perfect spin flips for a non-dispersive flipper (or a monochromatic beam). The r.f. region is deliberately shown shorter than the static field region (as is the case for an r.f. coil placed inside a static field coil). The flipper is tuned for exact *π*-flips within the r.f. field region so that *γ_n_B_rf_ l_rf_*/*v_i_* =*π*. In this case, by the time the neutrons exit the r.f. field region a complete inversion of the spin states has occurred via exchange of photons with the r.f. field (of angular frequency *ω_rf_* = *ω_0_*). The absorption and emission of r.f. photons means that, in contrast to the case shown in Fig. 7, the total energy of individual spin states is not conserved, as shown. The splitting of the total energy reaches a maximum of 2*ħω_0_* at the exit boundary of the r.f. field, whilst the additional splitting in kinetic energy experienced due to the spin inversion does not manifest itself until the neutron crosses the static field boundary on the exit side of the coil. Because the kinetic energies of the two states differ by 2*ħω_0_* at the exit of the coil, the relative phase of the spin-up and down states go in and out of phase corresponding to a Larmor precession in the subsequent *zero* field region of angular frequency 2*ω_0_*. This precession in zero field has been referred to as anomalous or “wrong” Larmor precession by Mezei [10].

Qualitatively, a kinetic energy splitting of the two spin states occurs on entry to the coil (*ΔE_k_* = *ħω_0_*) due to the static field gradient, whilst the total energy (= kinetic + potential) remains constant initially. The total spin inversion that occurs in the flipper involving exchange of photons with the r.f. field (see Eq. (92)) causes the *total* energy of the two states to become split by 2*ħω_0_*, but leaves their kinetic energy unchanged (still split by *ΔE_k_* = *ħω_0_*). At the coil exit, when crossing the static field boundary, the kinetic energy splitting does not disappear (as it did in the static-only field case (Sec. 4.1)). If 100 % spin inversion occurs due to the r.f. field, an *additional* splitting in kinetic energy (by *ħω_0_*) due to the static field gradient at the exit boundary takes place (i.e., the kinetic energy splitting doubles at the exit of the first coil (*ΔE_k_* = 2*ħω_0_*)). This explains the 2*ω_0_* precession frequency in region III (see Eq. (95)). This situation is shown (for perfect spin state inversion) in Fig. 8. Further accounts of these energy changes are found in Refs. [9–13].

##### 4.2.1.4 Off-resonance, exact *π*-flips for all velocities (i.e., no dispersion or monochromatic)

“No-dispersion” implies that the classical condition for exact *π*-flips around *B_rf_* is satisfied for *all* velocities, i.e., *ω_r_d*/*v_i_* = *π*, therefore from Eq. (80)

(98) $$\omega_{A}=\sqrt{\frac{\pi^{2}v_{i}^{2}}{4d^{2}}+\varepsilon^{2}}$$

so there is no convenient simplification of the wave function for *ε* ≠ 0.

##### 4.2.1.5 Passage through a second similar coil a distance *L* downstream (on-resonance, no dispersion or monochromatic)

Golub, Gähler, and Keller [13] extrapolate the wave function exiting the first coil through a zero-field path length, *L*, to the entrance of a second similar coil downstream (equivalent to the first arm of a *N* = 1 NRSE) and show that the wave function exiting the second coil is given by

(99) $$\psi_{exit\;B}=\frac{1}{\sqrt{2}}\left[\begin{array}{l} e^{-i\omega_{0}\left(\frac{L+d}{v_{i}}\right)}\\ e^{i\omega_{0}\left(\frac{L+d}{v_{i}}\right)} \end{array}\right]e^{i(k_{i}y-\omega_{i}t)}$$

so that the expectation value of the polarization with respect to *x* at the exit of the second coil is

(100) $$\begin{array}{l} \langle \sigma_{x}\rangle =\psi_{exit\;B}^{{}^{*}}\left[\begin{array}{ll} 0 & 1\\ 1 & 0 \end{array}\right]\psi_{exit\;B}=\frac{1}{2}\left[e^{i2\omega_{0}\left(\frac{L+d}{v_{i}}\right)}+e^{-i2\omega_{0}\left(\frac{L+d}{v_{i}}\right)}\right]\\ =\cos\left(2\omega_{0}\left(\frac{L+d}{v_{i}}\right)\right). \end{array}$$

The presence of the second similar flipper coil with the same static field orientation “mirror images” the neutron energy history shown in Fig. 8 so that the kinetic and total energies of the two spin states revert to their starting value *E_i_*. Therefore, the precession occurring downstream of the first coil is not observed downstream of the second coil. The final spin phase angle, 2*ω_0_*(*L*+*d*)/*v_i_*, agrees exactly with the classical result for the phase change in the first arm of the *N* = 1 spectrometer, as expected (see Eq. (62)).

### 4.3 What is the Effect of *l_rf_* ≠ *l_B0_*? – Coil Tuning

For *any* spin inversion to take place, the r.f. field must be applied in a region where the static field is also present (i.e., there is a Zeeman splitting to induce transitions between states). Secondly, the r.f. photons must have a frequency close to that of the splitting (resonance) so that transitions occur with high probability (Sec. 2.3). Finally, for optimum flipping probability, Eq. (13) must be satisfied in the overlap region between the static and r.f. fields, of length *l_π_*, applying the definition in Eq. (1).

#### 4.3.1 Monochromatic Beam with Static Field Region Enclosing the r.f. Region (*l_coil_ = l_B0_*, *l_π_ = l_rf_*)

A monochromatic beam eliminates the complication of dispersion and for a tuned coil, therefore the spins remain in the *x-y* plane after passage through the coil. This is exactly the situation depicted in Fig. 8. The static-field-only regions each side of the r.f. region behave as Larmor precession regions as described in Sec. 4.1 where the spins precess at rate *ω_0_* around the *z*-axis with the corresponding spin phase angle shift in the *x–y* plane. However, because the effect of the spin inversion in the “r.f. + *B_0_*” region (length *l_π_* = *l_rf_*) is not manifested until the neutron reaches the exit of the static field region, the effective Larmor precession continues at a rate *ω_0_* over the entire coil length (*l_coil_* = *l_B0_*) and at a rate 2*ω_0_* in the zero field region after the coil. Thus for an initial spin direction along *x*, the phase of the spin a distance *L* downstream of the static field region is given by

(101) $$\begin{array}{cc} \sigma_{x}(L)=\omega_{0}(l_{B_{0}}+2L) & l_{B0}\;\text{encloses} \end{array}\;l_{rf}.$$

Using the definition of coil length in Eq. (3), we have $l_{coil}=l_{B_{0}}$, therefore we can equally write

(102) $$\begin{array}{cc} \sigma_{x}(L)=\omega_{0}(l_{coil}+2L) & l_{B0}\;\text{encloses} \end{array}\;l_{rf}$$

with *l_π_* = *l_rf_* so that the specific *π*-flip condition is

(103) $$\begin{array}{cc} B_{rf}=\pi \frac{h}{\gamma_{n}m_{n}l_{rf}\lambda_{n}} & l_{B0}\;\text{encloses} \end{array}\;l_{rf}.$$

#### 4.3.2 Monochromatic Beam with r.f. Region Enclosing the Static Field Region (*l_coil_ = l_rf_, l_π_ = l_B0_*)

**Fig. 9:**
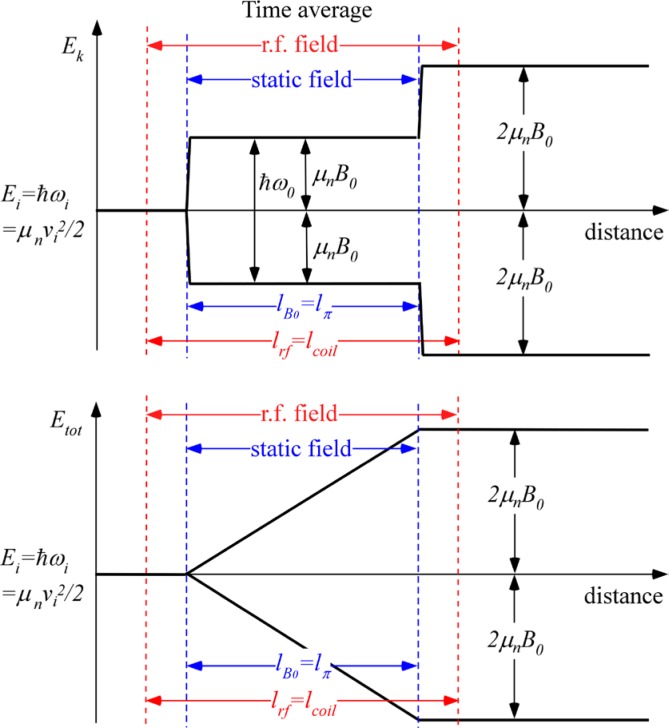
The oscillating r.f. field by itself does not change the average energy of the neutrons. In the field overlap region (length *l_π_*=*l_B0_*), the spin inversion occurs where all the change in the total energy of the two spin states occurs and where the effective Larmor precession angular frequency is *ω_0_*. The additional splitting of the kinetic energy brought about by the spin inversion occurs at the exit of the static field region, after which the effective Larmor precession frequency is 2*ω_0_*.

This case time-averaged is depicted in Fig. 9. Note that in the “r.f.-field-only” regions each side of the static field, the time-averaged field direction is random with respect to the neutron spin and so the average kinetic energy and total energy of the neutron spin states remain unchanged. Spin inversion and photon exchange occur only in the region where the static and r.f. fields are coincident, where all the change in total energy takes place. As before, the effect of the spin inversion on the kinetic energy is only felt at the static field boundary after which the time-averaged r.f. field does not change the total or kinetic energy. Thus for an initial spin aligned along *x*, the phase of the spin a distance *L* downstream of the *static field region* is given by

(104) $$\begin{array}{cc} \sigma_{x}(L)=\omega_{0}(l_{B_{0}}+2L) & l_{rf}\;\text{encloses} \end{array}\;l_{B0}.$$

However, using the definition of coil length in Eq. (3)*l_coil_* = *l_rf_*, no longer allows us to write an expression for *σ_x_* in terms of the coil length *l_coil_* as in Eq. (102). Therefore, if we always define *L* as the distance downstream of the *static field region*, Eq. (101) may be used for both geometrical cases. For this case, we have *l_π_* = *l_B0_* so that the specific *π*-flip condition is

(105) $$\begin{array}{cc} B_{rf}=\pi \frac{h}{\gamma_{n}m_{n}l_{B0}\lambda_{n}} & l_{rf}\;\text{encloses} \end{array}\;l_{B0}.$$

#### 4.3.3 With Dispersion or When Detuned from Resonance (*B_0_* Region Encloses *B_rf_*)

**Fig. 10:**
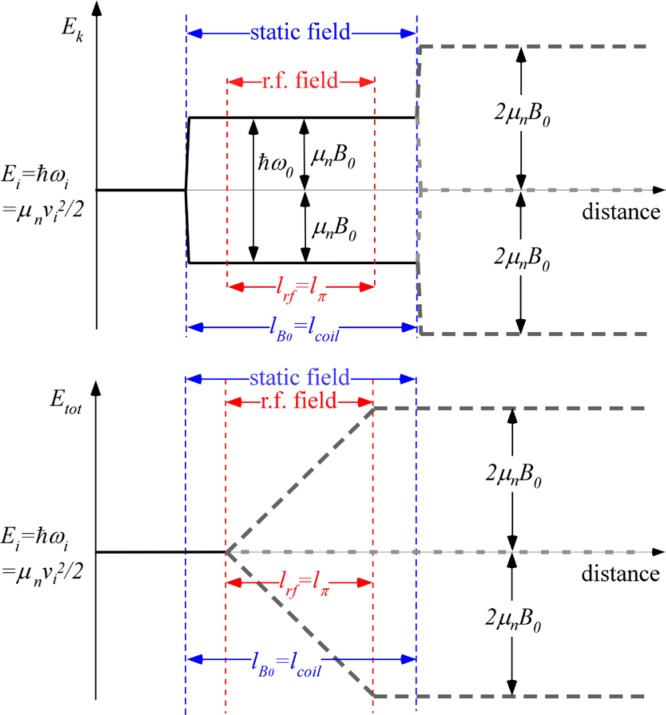
This is similar to the situation shown in Fig. 8, but with a reduction in flipping efficiency due to dispersion (Sec. 2.2) or a departure from the exact resonance condition (Sec. 2.3). A fraction of the neutrons that are not flipped return to their original kinetic energy state on leaving the static field region (light gray dashed lines). These neutrons have no splitting of their total energy. The flipped neutron fraction is represented by the dark gray dashed lines.

When dispersion is present or when the coil is not resonant for all neutrons (e.g., due to static field inhomogeneities), a fraction of the spins do not flip in the superimposed “static field + r.f. field” region. The unflipped neutrons behave similarly to neutrons in a pure static field (see Sec. 4.1 above), whilst the flipped component is subject to the behavior described in Sec. 4.2 and subsequent sections. This situation is illustrated (for the case where the *B_0_* field region encloses the r.f. field region) in Fig. 10.

## 5. Analysis of the Spin-Echo Signal

The following applies to an idealized spectrometer (no uncertainty on *B*, *L* etc.). The effects of instrumental uncertainties on the spin-echo signal are discussed in the Sec. 6. The emphasis here is on quasielastic applications of the NRSE. The terms in Eq. (68) corresponding to the neutron spin phase gain in the first arm and loss in the second arm of the spectrometer, expressed vectorially, are respectively:

(106) $$\varphi_{1}=2N\gamma_{n}\frac{B_{0}L_{0}}{v_{i}.{\hat{L}}_{0}}$$

and

(107) $$\varphi_{2}=-2N\gamma_{n}\frac{B_{1}L_{1}}{v_{f}.{\hat{L}}_{1}},$$

where *γ_n_* is the neutron gyromagnetic ratio, **v_i,f_** is the initial/final neutron velocity vector and ${\hat{L}}_{0,1}$ is a unit vector parallel to the axes of the first and second arms of the spectrometer (and perpendicular to the coil axis). The “−” sign in Eq. (107) implies that the field directions in the second arm are such that they reverse the spin phase angle change with respect to the first arm.

### 5.1 Small Divergence Approximation

For *small beam divergences* and coil axes that are perpendicular to **v_i_** and **v_f_**, we can approximate Eqs. (106) and (107) by

(108) $$\varphi_{0}=2N\gamma_{n}\frac{B_{0}L_{0}}{v_{i}}$$

and

(109) $$\varphi_{1}=-2N\gamma_{n}\frac{B_{1}L_{1}}{v_{f}}$$

respectively, where *v_i_* and *v_f_* are scalars, so that the net spin turn at the analyzer is given by Eq. (69). For *quasielastic* non-spin flip scattering that is sufficiently low energy transfer (δ*v* ≪ *v_i_*), we can write

(110) $$v_{f}\approx v_{i}+\delta v$$

so that Eq. (69) is approximately

(111) $$\varphi_{NRSE}\approx 2N\gamma_{n}\left[\frac{(B_{0}L_{0}-B_{1}L_{1})v_{i}+B_{0}L_{0}\delta v}{v_{i}(v_{i}+\delta v)}\right]\approx 2N\gamma_{n}\left[\frac{\delta (BL)v_{i}+B_{0}L_{0}\delta v}{v_{i}^{2}}\right],$$

where

(112) $$\delta (BL)=B_{0}L_{0}-B_{1}L_{1}$$

is often called the spectrometer *asymmetry*. It is conventional to perform NRSE asymmetric scans by fixing the static field and scanning *δL* = *L_0_* −*L_1_*. Also we will assume that *B_0_* = *B_1_* and replace *δ* (*BL*) by the slightly less general expression *δ* (*BL*) = *B_0_*(*L_0_* −*L_1_*) = *B_0_δL* in the following.

The measured quantity in neutron spin-echo is related to the polarization of the scattered beam. If the polarization is analyzed in the same direction as the polarization direction of the incident beam (assumed here to be the *x* axis), the polarization of the scattered beam is related (classically) to the cosine of *φ_NRSE_*, averaged over all the scattered neutron trajectories i.e.,

(113) $$P_{x}=\langle \cos\varphi_{NRSE}\rangle ,$$

where 〈〉 implies a statistical average over a *large* sample of scattered neutrons. From Eq. (111) we have

(114) $$\begin{array}{l} P_{x}=\langle \cos\varphi_{NRSE}\rangle =\langle \cos\left(2N\gamma_{n}B_{0}\frac{\delta Lv_{i}+L_{0}\delta v}{v_{i}^{2}}\right)\rangle \\ =\langle \cos\left(2N\gamma_{n}B_{0}\frac{\delta L}{v_{i}}\right)\cos\left(2N\gamma_{n}B_{0}L_{0}\frac{\delta v}{v_{i}^{2}}\right)-\sin\left(2N\gamma_{n}B_{0}\frac{\delta L}{v_{i}}\right)\sin\left(2N\gamma_{n}B_{0}L_{0}\frac{\delta v}{v_{i}^{2}}\right)\rangle . \end{array}$$

This expression must be averaged over all possible values of *v_i_* (the incident spectrum) and all possible values of *δv* (= *v_i_* − *v_f_*) determined by the scattering. Noting that *Q* is approximately independent of *ω*, i.e.,

(115) $$Q\approx \frac{2m_{n}v_{i}}{\hbar }\sin\theta ,$$

where 2*θ* is the scattering angle, we can write

(116) $$P(v_{i},\delta v)d(\delta v)≃S(Q,\omega )d\omega .$$

For *small* energy transfers (small *ω* and *v_i_* ≈ *v_f_*) we have from the definition of kinetic energy:

(117) $$\delta v=(v_{f}-v_{i})=\frac{2\hbar \omega }{m_{n}(v_{f}+v_{i})}\approx \frac{\hbar \omega }{m_{n}v_{i}}\approx \frac{\hbar \omega }{m_{n}v_{f}}.$$

If *S*(*Q,ω*) is *symmetric* in *ω* (usually a good approximation for quasielastic scattering) and substituting *δv* ≈ *ħω*/*m_n_v_i_* from Eq. (117), the average over the *δv* distribution characteristic of the scattering sample for a given *v_i_* becomes

(118) $$P_{x}(v_{i})=\cos\left(\frac{2N\gamma_{n}B_{0}}{v_{i}}\delta L\right)\int_{-\infty }^{\infty }S(Q,\omega )\cos\left(\frac{2N\hbar \gamma_{n}B_{0}L_{0}}{m_{n}v_{i}^{3}}\omega \right)\;d\omega \;\text{given}\;v_{i},$$

where the “sine” part of the expansion in Eq. (114) disappears in the integral for symmetric *S*(*Q,ω*) and the denominator $\int_{-\infty }^{\infty }S(Q,\omega )d\omega =1$ for a normalized scattering function is implicit. Note that the quantity preceding *ω* in the second cosine argument in Eq. (118) when *δ* (*BL*) = 0 (i.e., *B_0_L_0_* = *B_1_L_1_*) is often referred to as the spin-echo time, *τ_NRSE_*, i.e.,

(119) $$\tau_{NRSE}=\frac{2\hbar \gamma_{n}NB_{0}L_{0}}{m_{n}v_{i}^{3}}=\frac{\gamma_{n}}{\pi }{\left(\frac{m_{n}}{h}\right)}^{2}NB_{0}L_{0}\lambda_{i}^{3},$$

where

(120) $$\begin{array}{l} \tau_{NRSE}[\text{ns}]=0.37271N\;B_{0}[T]L_{0}[m]{\left(\lambda_{i}\left[\overset{\circ }{A}\right]\right)}^{3}\\ \equiv 1.27794\times 10^{-2}N\;v_{0}[\text{MHz}]L_{0}[m]{\left(\lambda_{i}\left[\overset{\circ }{A}\right]\right)}^{3}. \end{array}$$

Equation (118) must be averaged over the normalized incident velocity distribution, *F*(*v_i_*), so the final polarization is

(121) $$P_{x}=\int_{0}^{\infty }\int_{-\infty }^{\infty }F(v_{i})\cos\left(\frac{2N\gamma_{n}B_{0}}{v_{i}}\delta L\right)S(Q,\omega )\cos\left(\frac{2N\hbar \gamma_{n}B_{0}L_{0}}{m_{n}v_{i}^{3}}\omega \right)d\omega dv_{i}\;\text{symmetric}\;S(Q,\omega ),$$

where the denominator $\int_{0}^{\infty }F(v_{i})dv_{i}=1$ is implied. Expressed in terms of the normalized incident *wavelength* distribution, *I*(*λ_i_*), Eq. (121) becomes

(122) $$P_{x}=\int_{0}^{\infty }\int_{-\infty }^{\infty }I(\lambda_{i})\cos\left(\frac{2N\gamma_{n}m_{n}B_{0}}{h}\lambda_{i}\delta L\right)S(Q,\omega )\cos\left(\frac{N\gamma_{n}}{\pi }\left(\frac{m_{n}}{h}\right)B_{0}L_{0}\lambda_{i}^{3}\omega \right)d\omega d\lambda_{i}\;\text{symmetric}\;S(Q,\omega ).$$

The extent to which the spectrometer asymmetry cosine term contributes to the wavelength-dependence of the integrand depends on the situation. Since *Q* is also a function of *λ*, *Q*-dependent scattering also contributes to the wavelength-dependence of the integrand. However, for now we assume this part of the wavelength-dependence is weak or else the scattering is *Q*-independent. It should be remembered that Eq. (122) is valid for an essentially “perfect” spectrometer, i.e., negligible beam divergence and uncertainty in the value of *B_0_* and *L_0_*, and negligible flipper coil dispersion. These effects must be included separately. The effect of flipper coil dispersion has already been dealt with in Sec. 2.2 and the other instrumental effects are considered in Sec. 6. Examples are compared with simulation results in Sec. 8.

### 5.2 Special Cases for No Sample, Isotope Incoherent Elastic Scattering, or Small *ω* (“Resolution Function”)

Isotope incoherence implies scattering that is both *Q*-independent (so the *λ*-dependence of the scattering function can be ignored). Elastic scattering, or small *ω*, implies that there is negligible neutron wavelength change through the spectrometer and therefore the second cosine term in Eq. (122) is either unity or very close to unity. For the cosine term to exceed 0.99 requires *ωτ_NRSE_* ≤ 0.045*π*. Under these conditions Eq. (122) becomes

(123) $$P_{x}(\omega \to 0)\approx \int_{0}^{\infty }\int_{-\infty }^{\infty }I(\lambda_{i})\cos(A\lambda_{i})S(\omega )d\omega d\lambda_{i},$$

where for brevity we define

(124) $$A=\frac{2N\gamma_{n}m_{n}B_{0}}{h}\delta L$$

and the integral over *ω* evaluates to *S*(*Q*) since there is no other *ω*-dependence. The integral over *λ_i_* is readily performed for simple wavelength spectral functions, allowing analytical approximations for the “resolution” echo signal to be obtained in the absence of depolarizations resulting from instrumental imperfections. Expressions for purely monochromatic, rectangular, and triangular incident wavelength distributions are given in the following. The less trivial results for rectangular and triangular distributions are compared with simulations in Sec. 8.6.

#### 5.2.1 Purely Monochromatic Beam

For a purely monochromatic incident beam, *I*(*λ_i_*) = *δ*(*λ_0_*), and we have simply:

(125) $$P_{x}(\omega \to 0)\approx \cos(A\lambda_{0})\int_{-\infty }^{\infty }S(\omega )d\omega =S(Q)\cos(A\lambda_{0}),\;I(\lambda_{i})=\delta (\lambda_{0}).$$

Therefore, in the absence of instrumental imperfections (see Sec. 6), the resolution function has a pure cosinusoidal form of constant amplitude for any *δ* (*BL*) with a periodicity given by

(126) $$\delta {\left(BL\right)}_{2\pi }=\frac{h}{m_{n}}\frac{\pi }{\gamma_{n}N}\frac{1}{\lambda_{0}}=\frac{\pi v_{0}}{\gamma_{n}N}.$$

#### 5.2.2 Rectangular Incident Wavelength Spectrum

For a rectangular incident wavelength spectrum of full width *Δλ_FW_*, centered about *λ_i_* = 〈*λ_i_*〉, the wavelength-dependent integral in Eq. (123) becomes:

$$\frac{1}{\Delta \lambda_{FW}}\int_{\langle \lambda_{i}\rangle -\Delta \lambda_{FW}/2}^{\langle \lambda_{i}\rangle +\Delta \lambda_{FW}/2}\cos(A\lambda_{i})d\lambda_{i}=\frac{2}{A\Delta \lambda_{FW}}\sin(A\Delta \lambda_{FW}/2)\cos(A\langle \lambda_{i}\rangle ),$$

provided that the lower wavelength limit of integration is greater than zero, so that

(127) $$P_{x}(\omega \to 0)\approx \frac{2S(Q)}{A\Delta \lambda_{FW}}\sin\left(A\frac{\Delta \lambda_{FW}}{2}\right)\cos(A\langle \lambda_{i}\rangle ),\;\text{Rectangular incident spectrum},$$

apart from depolarizations resulting from instrumental imperfections and flipper coil dispersion.

#### 5.2.3 Triangular Incident Wavelength Spectrum

The triangular incident spectrum is useful in many practical situations as it is approximately the shape delivered by neutron velocity selectors when the source spectrum varies slowly within the selected wavelength range. For a triangular incident spectrum of FWHM = *Δλ_FWHM_* and mean wavelength 〈*λ_i_*〉, the wavelength-dependent integral in Eq. (123) becomes

$$\frac{1}{\Delta \lambda_{FWHM}^{2}}\left[\begin{array}{l} \int_{\langle \lambda_{i}\rangle -\Delta \lambda_{FWHM}}^{\langle \lambda_{i}\rangle }(\lambda_{i}-\langle \lambda_{i}\rangle +\Delta \lambda_{FWHM})\cos(A\lambda_{i})d\lambda_{i}+\\ \int_{\langle \lambda_{i}\rangle }^{\langle \lambda_{i}\rangle +\Delta \lambda_{FWHM}}(\langle \lambda_{i}\rangle +\Delta \lambda_{FWHM}-\lambda_{i})\cos(A\lambda_{i})d\lambda_{i} \end{array}\right]=\frac{1}{\Delta \lambda_{FWHM}^{2}A^{2}}[2\cos(A\langle \lambda_{i}\rangle )(1-\cos(A\Delta \lambda_{FWHM}))]$$

provided that the lower wavelength limit of integration is greater than zero, so that

(128) $$P_{x}(\omega \to 0)\approx \frac{S(Q)}{\Delta \lambda_{FWHM}^{2}A^{2}}\left\{2\cos(A\langle \lambda_{i}\rangle )[1-\cos(A\Delta \lambda_{FWHM})]\right\}\;\text{Triangular incident spectrum},$$

apart from depolarizations resulting from instrumental imperfections and flipper coil dispersion.

### 5.3 Special Cases for Quasielastic Neutron Scattering (QENS) Symmetric Scans

In this case, *δL* → 0 in Eq. (122) and we have:

(129) $$P_{x}(\delta L\to 0)\approx \int_{0}^{\infty }\int_{-\infty }^{\infty }I(\lambda_{i})S(Q,\omega )\cos\left(\frac{\gamma_{n}}{\pi }{\left(\frac{m_{n}}{h}\right)}^{2}NB_{0}L_{0}\lambda_{i}^{3}\omega \right)d\omega d\lambda_{i}.$$

For quasielastic scattering, the scattering function is represented by a Lorentzian

(130) $$S(Q,\omega )=\frac{1}{\pi }\frac{\gamma (Q)}{\gamma {\left(Q\right)}^{2}+\omega^{2}},$$

where *γ* (*Q*) = *Γ* (*Q*)/*ħ*, where *Γ*(*Q*) is the energy half-width at half maximum. Performing the integral over *ω*, we have

(131) $$\begin{array}{l} P_{x}(\delta L\to 0)\approx \int_{0}^{\infty }I(\lambda_{i})\exp\left(-\frac{\gamma_{n}}{\pi }{\left(\frac{m_{n}}{h}\right)}^{2}NB_{0}L_{0}\frac{\Gamma (Q)}{\hbar }\lambda_{i}^{3}\right)d\lambda_{i}\\ =\int_{0}^{\infty }I(\lambda_{i})\exp\left(-\frac{\Gamma (Q)}{\hbar }\tau_{NRSE}\right)d\lambda_{i}. \end{array}$$

We now consider the possible *λ*-dependence of *Γ*(*Q*). Following Hayter and Penfold [17], we consider a common case of self-diffusion at low *Q* at fixed scattering angle, *θ*. For simplicity, we ignore the very small change in energy of the neutrons on scattering, so we may make the approximation

(132) $$\Gamma (Q)=\hbar DQ^{2}\approx \frac{16\hbar \pi^{2}D\sin^{2}\theta }{\lambda_{i}^{2}}\;\text{QENS},\;\text{self-diffusion low}\;Q(Q\approx Q_{el}),$$

where *θ* is the scattering angle, so that Eq. (131) becomes

(133) $$\begin{array}{l} P_{x}(\delta L\to 0)\approx \int_{0}^{\infty }I(\lambda_{i})\exp\left(-16\pi \gamma_{n}{\left(\frac{m_{n}}{h}\right)}^{2}NB_{0}L_{0}D\sin^{2}\theta \;\lambda_{i}\right)d\lambda_{i}\\ =\int_{0}^{\infty }I(\lambda_{i})\exp(-K\lambda_{i})d\lambda_{i}\;\text{QENS},\;\text{self-diff low}\;Q(Q\approx Q_{el}), \end{array}$$

where we have set

(134) $$K=16\pi \gamma_{n}{\left(\frac{m_{n}}{h}\right)}^{2}NB_{0}L_{0}D\sin^{2}\theta =\frac{16\pi^{2}D\sin^{2}\theta }{\lambda_{i}^{3}}\tau_{NRSE}.$$

#### 5.3.1 Purely Monochromatic Beam

For a purely monochromatic incident beam, *I*(*λ_i_*) = *δ* (*λ_0_*), and Eq. (133) becomes simply:

(135) $$P_{x}(\delta L\to 0)\approx \exp(-K\lambda_{0})\equiv \exp\left(-\frac{\Gamma (Q)\tau_{NRSE}}{\hbar }\right)\;I(\lambda_{i})=\delta (\lambda_{0}).$$

#### 5.3.2 Rectangular Incident Wavelength Spectrum

For a rectangular incident wavelength spectrum of full width *Δλ_FW_*, centered about *λ_i_* = 〈*λ_i_*〉, Eq. (133) becomes

(136) $$\begin{array}{l} P_{x}(\delta L\to 0)\approx \frac{1}{\Delta \lambda_{FW}}\int_{\langle \lambda_{i}\rangle -\Delta \lambda_{FW}/2}^{\langle \lambda_{i}\rangle +\Delta \lambda_{FW}/2}\exp(-K\lambda_{i})d\lambda_{i}\\ =\frac{\sinh(K\;\Delta \lambda_{FW}/2)}{\left(K\;\Delta \lambda_{FW}/2\right)}\exp(-K\langle \lambda_{i}\rangle ). \end{array}$$

#### 5.3.3 Triangular Incident Wavelength Spectrum

For a triangular incident spectrum of FWHM = *Δλ_FWHM_* and mean wavelength 〈*λ_i_*〉, Eq. (133) becomes

(137) $$\begin{array}{l} P_{x}(\delta L\to 0)\approx \frac{1}{\Delta \lambda_{FWHM}^{2}}\left[\begin{array}{l} \int_{\langle \lambda_{i}\rangle -\Delta \lambda_{FWHM}}^{\langle \lambda_{i}\rangle }(\lambda_{i}-\langle \lambda_{i}\rangle +\Delta \lambda_{FWHM})\exp(-K\lambda_{i})d\lambda_{i}+\\ \int_{\langle \lambda_{i}\rangle }^{\langle \lambda_{i}\rangle +\Delta \lambda_{FWHM}}(\langle \lambda_{i}\rangle +\Delta \lambda_{FWHM}-\lambda_{i})\exp(-K\lambda_{i})d\lambda_{i} \end{array}\right]\\ =\frac{2(\cosh(K\Delta \lambda_{FWHM})-1)}{K^{2}\Delta \lambda_{FWHM}^{2}}\exp(-K\langle \lambda_{i}\rangle ). \end{array}$$

#### 5.3.4 Gaussian Incident Wavelength Spectrum

For a Gaussian incident spectrum of FWHM = *Δλ_FWHM_* (standard deviation *σ*) and mean wavelength 〈*λ_i_*〉, Eq. (133) becomes

(138) $$\begin{array}{l} P_{x}(\delta L\to 0)\approx \frac{1}{2}\exp(-K\langle \lambda_{i}\rangle )\exp\left(\frac{\sigma^{2}K^{2}}{2}\right)\left(\text{erf}\left(\frac{\langle \lambda_{i}\rangle -\sigma^{2}K}{\sqrt{2}\sigma }\right)+1\right)\\ \sigma =\frac{\Delta \lambda_{FWHM}}{\sqrt{8\ln2}}. \end{array}$$

### 5.4 Detected Signal

#### 5.4.1 Perfect Polarizer, Analyzer and Non-Spin Flip Scattering

In an NRSE instrument, for a perfect polarizer and analyzer, the non-spin flip quasielastic signal in the detector is

(139) $$I_{+}=\frac{1}{2}\left(1+\langle \cos\varphi_{{}_{\mathrm{NRSE}}}\rangle \right)=\frac{1}{2}\left(1+\langle \cos\;\omega \tau_{{}_{\mathrm{NRSE}}}\rangle \right)=\frac{1}{2}\left(1+P_{x}\right),$$

where *P_x_* has been derived for some specific cases in the preceding section and is proportional to the intermediate scattering function. Measuring scattering in the time domain rather than in energy has the significant advantage that the scattering function is obtained from the measured data by simple division by the instrumental resolution function, rather than by deconvolution. This feature allows for very sensitive line shape analysis.

**Fig. 11:**
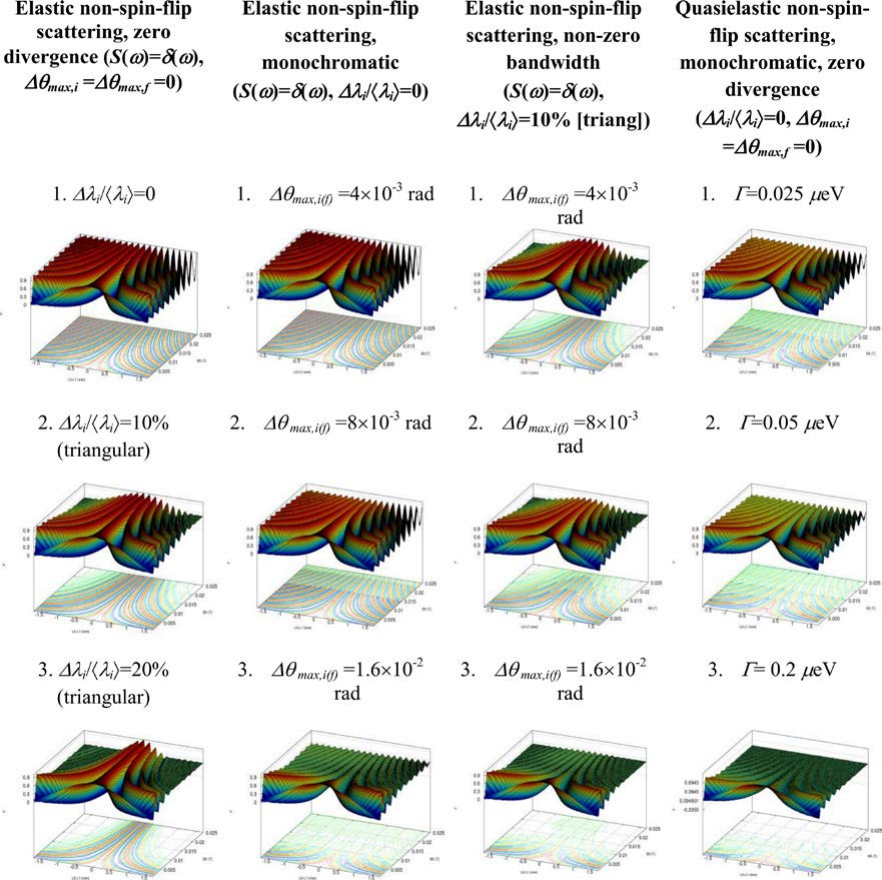
Some examples of Monte Carlo-simulated NRSE echo signals in the vicinity of the echo point demonstrating the effects of varying different parameters in isolation. These particular simulations were performed for a 4 *N*=2 bootstrap coil NRSE with *L_1_* = 2.0 m, *l_B0_* = 3.0 cm, *l_g_* = 0.0 cm, and *λ* = 8 Å. The axis oriented into the paper represents the static field *B_0_* from 10^−3^ T at the front to 0.025 T (*τ_NRSE_* ≈ 19 ns for these spectrometer parameters) at the rear for each plot. The horizontal axis more parallel to the plane of the paper is the spectrometer asymmetry (*L_0_* − *L_1_*), in units of mm, between about ± 1.7 mm for each plot. Columns 1 to 3 are for elastic non spin-flip scattering (or no neutron energy change through the spectrometer). For the pure monochromatic elastic scattering examples there is no coil dispersion. Column 4 is for quasielastic non spin-flip scattering. For each of these simulations there are no sample size effects and *ΔB_0_* = *Δl_B0_* = 0 (zero field inhomogeneity and perfect dimensions of the flipper coils). Additionally for columns 1 and 4 zero beam divergence is assumed.

Monte Carlo simulations illustrating the behavior of Eq. (139) in various situations are shown in Fig. 11. In these examples there are no sample size effects and *ΔB_0_* = *Δl_B0_* = 0 (zero field inhomogeneity and perfect dimensions of the flipper coils), but the effects of beam divergence, incident neutron bandwidth, and non-elastic scattering are illustrated. The effects of spectrometer imperfections are analyzed further in Sec. 6 and additional simulation examples are given in Sec. 8. Columns 1 to 3 of Fig. 11 are for elastic, non-spin-flip (isotope incoherent elastic) scattering (or no sample). Column 4 is for quasielastic, isotope incoherent (non-spin-flip) scattering. Additionally for columns 1 and 4 zero beam divergence is assumed. These particular simulations were performed for a 4-*N*=2 bootstrap coil NRSE with *L_1_* = 2.0 m, *l_B0_* = 3.0 cm, *l_g_* = 0.0 cm, and *λ_0_* = 8 Å, for 10^−3^ ≤ *B_0_*(T) ≤ 0.025. The asymmetric scan is performed with |*B_0_*| =|*B_1_*|, with (*L_0_* − *L_1_*) varied ten minimum periods each side of the symmetric position (i.e., between $\pm 10\pi v_{0}/\gamma_{n}NB_{0}^{\max}$, where *B_0_^max^* is the maximum applied static field [0.025 T], corresponding to *τ_NRSE_* = 19.1 ns). *δ* (*BL*) was varied by changing *L_1_* with respect to *L_0_*. (i.e., *δ* (*BL*) = *BδL*). The incoming and outgoing beam divergence, if any, is equal and uniform up to a maximum *Δθ_i,max_* = *Δθ_f,max_* = *Δθ_max_*, and is symmetrical with respect to the nominal axes for both spectrometer arms (see also Sec. 6.4). Under these conditions, the echo maximum is found at *L_0_* = *L_1_*. The left hand column of Fig. 11 illustrates the effect of broadening *Δλ_i_* for elastic, non-spin flip scattering (or no sample). In the extreme, purely monochromatic case of *Δλ_i_* = 0, the signal is cosinusoidal with respect to *δL* (as predicted by Eq. (125) with *δ* (*BL*) = *BδL* for one signal period given by Eq. (126). For *Δλ_i_* > 0, the maximum signal is achieved at the symmetrical spectrometer setting and, as *Δλ_i_* increases, the primary envelope of the echo signal tightens around this point. Note that the period (in *L*) also decreases inversely proportional to *B_0_* (= *B_1_*) (and hence *τ_NRSE_*), as predicted by Eq. (126). The second and third columns show the effect of increasing the neutron flight path differences via increasing beam divergence for (i) a purely monochromatic incident beam (column 2), and (ii) a triangular wavelength distribution with *Δλ_i_*/〈*λ_i_*〉 = 10 % (column 3). The fourth column demonstrates the increasingly rapid decay of the echo point signal with respect to *τ_NRSE_* as the quasielastic width is increased (as predicted by Eq. (135) for a purely monochromatic incident beam (*Δλ_i_* = 0)).

#### 5.4.2 Imperfect Polarizers with Non-Spin Flip Scattering or No Sample

Real polarizing devices transmit a fraction of the wrong spin state, which results in a reduction of the NRSE signal. It is important to correct data in such a way as to isolate depolarization due to sample dynamics from instrumental depolarization as far as it is possible. Considering the quantum mechanical description of the polarization in terms of spin-up and spin-down neutrons, the polarizing efficiency of a “+” polarizer is numerically equal to the polarization of an initially unpolarized beam obtained after action of the polarizer. Using the definition in Eq. (54), the polarization after the action of the initial polarizer is

(140) $$P_{P}=\frac{I_{P}^{+}-I_{P}^{{}^{-}}}{I_{P}^{+}+I_{P}^{{}^{-}}}$$

where $I_{P}^{+}$ and $I_{P}^{{}^{-}}$ are the intensities of + and − neutrons in the beam after the polarizer *P*. Note that *P_P_* can vary between +1 and −1. The incoming unpolarized beam of total intensity *I_0_* is described by equal + and − components, i.e.,

(141) $$I_{0}^{+}=I_{0}^{{}^{-}}=\frac{I_{0}}{2}.$$

The total intensity after the polarizer

(142) $$I_{P}^{tot}=I_{P}^{+}+I_{P}^{{}^{-}}=T_{P}\frac{I_{0}}{2}=T_{P}I_{0}^{+}$$

where we have used the boundary condition that for perfect + polarization efficiency (*P_P_* =1), only the + state neutrons of the originally unpolarized beam are transmitted (i.e., one half of the neutrons of the incoming beam) and we assume that this total number is conserved for inefficient polarizers. *T_P_* is the spin-independent transmission factor of the device with 0 < *T_P_* < 1 due to effects such as absorption or scattering. From Eqs. (140) and (142) it is easy to show that after the polarizer:

(143) $$I_{P}^{+}=T_{P}\frac{I_{0}}{4}(1+P_{P})=T_{P}\frac{I_{0}^{+}}{2}(1+P_{P})$$

and

(144) $$I_{P}^{{}^{-}}=T_{P}\frac{I_{0}}{4}(1-P_{P})=T_{P}\frac{I_{0}^{{}^{-}}}{2}(1-P_{P}).$$

Therefore, the combined action of the polarizer (*P*) with the analyzer (*A*), both oriented to transmit + spin neutrons, for non-spin flip scattering is expected to give transmitted intensities

(145) $$I_{PA}^{+}=T_{A}\frac{I_{P}^{+}}{2}(1+P_{A})=T_{P}T_{A}\frac{I_{0}^{+}}{4}(1+P_{P})(1+P_{A})=T_{P}T_{A}\frac{I_{0}}{8}(1+P_{P})(1+P_{A}).$$

Likewise

(146) $$I_{PA}^{{}^{-}}=T_{A}\frac{I_{P}^{{}^{-}}}{2}(1-P_{A})=T_{P}T_{A}\frac{I_{0}^{{}^{-}}}{4}(1-P_{P})(1-P_{A})=T_{P}T_{A}\frac{I_{0}}{8}(1-P_{P})(1-P_{A})$$

with the total beam intensity after the analyzer

(147) $$I_{PA}^{tot}=I_{PA}^{+}+I_{PA}^{{}^{-}}=T_{P}T_{A}\frac{I_{0}}{4}(1+P_{P}P_{A}).$$

Therefore, the final polarization for non-spin flip scattering is

(148) $$P_{PA}=\frac{I_{PA}^{+}-I_{PA}^{{}^{-}}}{I_{PA}^{+}+I_{PA}^{{}^{-}}}=\frac{P_{P}+P_{A}}{1+P_{P}P_{A}}.$$

If two *π*-flippers of efficiency *f_1_* and *f_2_* and spin-independent transmission factor *T_f1_* and *T_f2_* are placed between the polarizer and the analyzer, and remembering that the effect of a *π*-flipper of efficiency *f* is to multiply the incoming polarization by the factor (1–2*f*) (see Sec. 2.4), we infer by analogy with Eqs. (145) and (146) that the + and – intensities downstream of the analyzer (i.e., at the detector) are

(149) $$\begin{array}{l} I_{PA}^{+}=T_{f_{1}}T_{f_{2}}T_{P}T_{A}\frac{I_{0}}{8}(1+P_{P})(1+P_{A})\\ I_{PA}^{{}^{-}}=T_{f_{1}}T_{f_{2}}T_{P}T_{A}\frac{I_{0}}{8}(1-P_{P})(1-P_{A})\\ I_{PA}^{tot}=T_{f_{1}}T_{f_{2}}T_{P}T_{A}\frac{I_{0}}{4}(1+P_{P}P_{A}) \end{array}\}\;\text{both flippers off},$$

(150) $$\begin{array}{l} I_{PA}^{+}=T_{f_{1}}T_{f_{2}}T_{P}T_{A}\frac{I_{0}}{8}(1+[1-2f_{1}]P_{P})(1+P_{A})\\ I_{PA}^{{}^{-}}=T_{f_{1}}T_{f_{2}}T_{P}T_{A}\frac{I_{0}}{8}(1-[1-2f_{1}]P_{P})(1-P_{A})\\ I_{PA}^{tot}=T_{f_{1}}T_{f_{2}}T_{P}T_{A}\frac{I_{0}}{4}(1+[1-2f_{1}]P_{P}P_{A}) \end{array}\}\;\text{only flipper}\;1\;\text{on},$$

(151) $$\begin{array}{l} I_{PA}^{+}=T_{f_{1}}T_{f_{2}}T_{P}T_{A}\frac{I_{0}}{8}(1+[1-2f_{2}]P_{P})(1+P_{A})\\ I_{PA}^{{}^{-}}=T_{f_{1}}T_{f_{2}}T_{P}T_{A}\frac{I_{0}}{8}(1-[1-2f_{2}]P_{P})(1-P_{A})\\ I_{PA}^{tot}=T_{f_{1}}T_{f_{2}}T_{P}T_{A}\frac{I_{0}}{4}(1+[1-2f_{2}]P_{P}P_{A}) \end{array}\}\;\text{only flipper}\;2\;\text{on},$$

and

(152) $$\begin{array}{l} I_{PA}^{+}=T_{f_{1}}T_{f_{2}}T_{P}T_{A}\frac{I_{0}}{8}(1+[1-2f_{1}][1-2f_{2}]P_{P})(1+P_{A})\\ I_{PA}^{{}^{-}}=T_{f_{1}}T_{f_{2}}T_{P}T_{A}\frac{I_{0}}{8}(1-[1-2f_{1}][1-2f_{2}]P_{P})(1-P_{A})\\ I_{PA}^{tot}=T_{f_{1}}T_{f_{2}}T_{P}T_{A}\frac{I_{0}}{4}(1+[1-2f_{1}][1-2f_{2}]P_{P}P_{A}) \end{array}\}\;\text{both flippers}\;1\;\text{and}\;2\;\text{on}.$$

As pointed out by Hayter [18], the ratio of the detector count rates, *I_PA_^tot^*, measured with both *π*-flippers switched off to the count rates with the two flippers switched “on-off”, “off-on” and “on-on” provides three “flipping ratios”, *R_1_*, *R_2_*, and *R_12_* respectively, which no longer have the spin-independent pre-factors common to each measurement. We thus have three equations for the three unknowns: *f_1_*, *f_2_*, and the product of the polarizer and analyzer efficiencies, *P_P_P_A_*, which can be solved to obtain

(153) $$P_{P}P_{A}=\frac{R_{12}(R_{1}-1)(R_{2}-1)}{\left(R_{1}R_{2}-R_{12}\right)},$$

(154) $$f_{i}=\frac{\left(R_{i}-1\right)}{2R_{i}}\frac{\left(1+P_{P}P_{A}\right)}{P_{P}P_{A}}.$$

The flipping ratios are determined for multi-angle instruments by using a diffuse, non-spin flip scattering sample such as quartz.

In an *M*-coil NRSE instrument with non-spin flipping samples (e.g. pure nuclear coherently-scattering samples), the polarization (NRSE signal) is reduced from the ideal value by the product of these instrumental inefficiencies. Therefore, the corrected signal, *P_corr_*, is related to the measured signal, *P_meas_*, by

(155) $$P_{corr}=\frac{P_{meas}}{P_{P}P_{A}\prod_{i=1}^{M}\left(1-2f_{i}\right)}.$$

#### 5.4.3 Imperfect Polarizers with Spin Flip Scattering

When there is a sample that modifies the spin state of the incoming neutrons, the spin transfer function of the sample has to be taken into account just like the function (1 − 2*f*) for the flipper. Table 6 shows relative spin-flip probabilities for various types of nuclear scattering for non-magnetic samples.

Consider a non-magnetic sample that flips a fraction *q* of the neutron spins so that, in exact analogy with the *π*-flipper (Sec. 2.4) and Eq. (55), the polarization after the sample, *P_S_*, is related to the polarization before the sample, *P_i_*, by

(156) $$P_{S}=(1-2q)P_{i}.$$

For the simple example of a single isotope, pure incoherent scatterer, 1/3 of the neutrons have their spins unchanged whilst 2/3 of the neutrons have their spins flipped by *π*. Thus the sample flipping efficiency is given by *q* = 2/3, consequently

(157) $$\frac{P_{S}}{P_{i}}=-\frac{1}{3}\;\text{non-magnetic},\;\text{pure isotope incoherent scatterer}.$$

This means that the spin-echo signal amplitude is reduced to 1/3 and the minus sign means that the echo signal is inverted. For this case, Eq. (155) becomes

(158) $$P_{corr}=-\frac{3P_{meas}}{P_{P}P_{A}\prod_{i=1}^{N}\left(1-2f_{i}\right)}.$$

For a more general non-magnetic case where both spin-incoherent and coherent scattering are present, we might have

(159) $$q\approx \frac{2S_{inc}(Q)}{3(S_{coh}(Q)+S_{inc}(Q))},$$

where we have assumed that the relative probabilities of coherent and spin-incoherent scattering are given by *S_coh_*(*Q*) and *S_inc_*(*Q*) respectively, therefore

(160) $$\frac{P_{s}}{P_{i}}=\frac{3S_{coh}(Q)-S_{inc}(Q)}{3\left(S_{coh}(Q)+S_{inc}(Q)\right)}.$$

This represents an upper limit on the size of the spin-echo signal. For this case Eq. (155) becomes

(161) $$P_{corr}=\frac{3P_{meas}\left(S_{coh}(Q)+S_{inc}(Q)\right)}{P_{A}P_{A}\prod_{i=1}^{M}\left(1-2f_{i}\right)\left[3S_{coh}(Q)-S_{inc}(Q)\right]}.$$

Other scattering cases including paramagnetic, ferromagnetic, and antiferromagnetic samples have been discussed by Mezei [3]. In order to determine the exact spin-flip/non-spin flip behavior of the sample, a conventional polarization analysis arrangement may be used with a polarizer and analyzer and only one flipper switched on or off.

## 6. Analysis of Contributions to the Elastic Instrumental Resolution Function: Allowable Flight Path Differences and Static Magnetic Field Inhomogeneity

The spin-echo phase is given by Eq. (69), i.e.,

$$\varphi_{NRSE}=\varphi_{0}-\varphi_{1}=\frac{2Nm_{n}\gamma_{n}}{h}\left[B_{0}L_{0}\lambda_{i}-B_{1}L_{1}\lambda_{f}\right].$$

At the echo point, 〈*φ_NRSE_*〉=0, however, even for *λ_i_ = λ_f_* (elastic scattering or no sample), *φ_NRSE_* has a distribution of values about the mean, 〈*φ_NRSE_*〉, of characteristic width *δφ_NRSE_.* This is because the terms *B_0_L_0_* and *B_1_L_1_* have non-zero spread, *Δ*(*B_0_L_0_*) and *Δ*(*B_1_L_1_*) respectively^2^, arising from instrumental imperfections. Consequently, *Δφ_0_* and *Δφ_1_* are non-zero and the valued information, which is the depolarization due to *the scattering energy transfer distribution*, is modified by the instrumental depolarization. The instrumental uncertainty, *Δ*(*BL*), determines the *elastic instrumental resolution function.* In order to obtain a broad dynamic range, *Δφ_NRSE_* must be dominated by the distribution of *λ_i_ − λ_f_* from sample energy exchanges (rather than the uncertainties in the *BL* terms) to the largest field magnitudes possible.

If we assume Gaussian uncertainties on the values of *B* and *L* and that *B* and *L* are independent variables, we expect *φ_NRSE_* also to have a Gaussian distribution, *g*(*φ_NRSE_*). At the echo point (〈*B_0_L_0_*〉 = 〈*B_1_L_1_*〉), *g*(*φ_NRSE_*) is symmetrically distributed about zero (polarization realigned along the original direction – the *x* axis in these examples). For the Gaussian distribution *g*(*φ_NRSE_*), the elastic scattering polarization along *x*, *P_x_^0^*, is

(162) $$\begin{array}{l} P_{x}{}^{0}\left(\Delta \varphi_{NRSE}^{FWHM}\right)=\frac{\int_{0}^{\infty }\cos(\varphi_{NRSE})g(\varphi_{NRSE})d\varphi_{NRSE}}{\int_{0}^{\infty }g(\varphi_{NRSE})d\varphi_{NRSE}}\\ =\exp\left[-\frac{{\left(\Delta \varphi_{NRSE}^{FWHM}\right)}^{2}}{16\ln2}\right]\approx \exp\left[-0.09{\left(\Delta \varphi_{NRSE}^{FWHM}\right)}^{2}\right], \end{array}$$

or the inverse relation

(163) $$\Delta \varphi_{NRSE}^{FWHM}=4\sqrt{\;\ln\;2\;\ln\left[\frac{1}{P_{x}^{0}}\right]}\approx \sqrt{11.1\;\ln\left[\frac{1}{P_{x}^{0}}\right]}.$$

We use this convenient form when estimating spectrometer tolerances in the following sections. One notes that if the distribution *g*(*φ_NRSE_*) was *uniform* between the limits ± *Δφ_NRSE_^max^*, rather than Gaussian, the analogue of Eq. (162) is a sinc function of ± *Δφ_NRSE_^max^*:

(164) $$P_{x}^{0}\left(\Delta \varphi_{NRSE}^{\max}\right)=\frac{\sin\Delta \varphi_{NRSE}^{\max}}{\Delta \varphi_{NRSE}^{\max}}.$$

The elastic and quasi-elastic signal count rate cannot exceed a maximum proportional to *P_x_^0^* (for pure coherent scatterers) and sometimes considerably less for incoherent scatterers (see Sec. 5.4.3), therefore *P_x_^0^*(*τ_NRSE_*) must remain comfortably greater than zero. In order to avoid excessive counting times or poor signal-to-noise ratio we suggest a practical minimum *P_x_^0^ >* 0.2 at the maximum required *τ_NRSE_* in a quasielastic measurement. Purely coherent, elastic scatterers, such as Grafoil®, Carbopack™, and carbon black are all used for measuring the resolution function in spin-echo spectrometers.

In order to estimate the depolarization produced by static field inhomogeneities, dimensional uncertainties, and beam divergence, we use the convenience of Eq. (163). We further assume *similar* distributions of *φ_0_* and *φ_1_*, which imposes *Δφ_0_ = Δφ_1_*, and that the spectrometer is operated at the echo point (i.e., 〈*φ_0_*〉= 〈*φ_1_*〉) If *φ_0_* and *φ_1_* are distributed normally, we can write

(165) $$\Delta \varphi_{NRSE}=\sqrt{\Delta \varphi_{0}^{2}+\Delta \varphi_{1}^{2}}\approx \sqrt{2}\Delta \varphi_{0}.$$

In order to isolate individual contributions, we analyze first the effect of static magnetic field inhomogeneities in the absence of flight path uncertainties, and secondly, the flight path uncertainties in the absence of field inhomogeneities. We also separate the flight path uncertainties due to spectrometer dimensional fluctuations from those due to beam divergence. For the beam divergence, we cannot assume Gaussian distributions, as explained in Sec. 6.4.

### 6.1 Static Magnetic Field Inhomogeneities

We may consider the effect of static field inhomogeneity as creating a distribution of values of *ω_0_ − ω_rf_.* In order to simplify the argument we consider *ω_rf._ as* being precisely fixed (a reasonable assumption for a high quality frequency generator). The effect of field inhomogeneity is isolated by attributing all the fluctuation in *ω_0_ − ω_rf_ to* the distribution of *ω_0_* caused by the field inhomogeneity, *ΔB_0_*, and comparing the polarization with the equivalent system in which *ω_0_* = *ω_rf_* for all trajectories (*ΔB_0_* = 0). Further, we assume that the spectrometer is optimally tuned such that *〈ω_0_〉 = ω_rf_* and that the field inhomogeneity gives rise to a normal distribution of *ω_0_* with respect to *〈ω_0_〉.* In this approximation, the effect of static field inhomogeneity is analogous to the effect of dispersion discussed in Sec. 2.2. The effect of *ω_0_* ≠ *ω_rf_* is conveniently visualized in the rotating coordinate system, as proposed by Rabi, Ramsey, and Schwinger in Ref. [15], whereby the rotating field magnitude transforms to an effective field of magnitude

(166) $$\left|B_{rf}^{eff}\right|=\frac{\sqrt{{\left(\omega_{0}-\omega_{rf}\right)}^{2}+\omega_{p}^{2}}}{\gamma_{n}}.$$

The effective field lies at an angle *α_eff_* to the *x-y* plane given by

(167) $$\alpha_{eff}=\tan^{-1}\frac{\omega_{0}-\omega_{rf}}{\gamma_{n}\left|B_{rf}\right|}=\tan^{-1}\frac{\omega_{0}-\omega_{rf}}{\omega_{p}}.$$

This is implicit in the quantum mechanical treatment of Ref. [13] discussed in Sec. 4.2. We now find an approximate relation between the static field inhomogeneity and the consequent depolarization that works well within certain limits.

The spin-flip probability for exact resonance (*ω_0_* = *ω_rf_*) is given by Eq. (86) and in the general off-resonance case by Eq. (83). If *Δφ_π_* represents the difference in the *x-y* spin turn in the off-resonance case with respect to exact resonance case, then, in analogy with Sec. 2.2, we equate the ratio of the spin-flip probabilities with the quantity 〈cos*ε* cos*Δφ_π_*〉, where *ε* here refers to the angle of the spin vector out of the *x-y* plane, i.e.,

(168) $$\frac{\frac{\omega_{p}^{2}}{\omega_{p}^{2}+{\left(\gamma_{n}\Delta B_{0}\right)}^{2}}\sin^{2}\left(\frac{\sqrt{\omega_{p}^{2}+{\left(\gamma_{n}\Delta B_{0}\right)}^{2}}m_{n}l_{B_{0}}\lambda_{i}}{2h}\right)}{\sin^{2}\left(\frac{\omega_{p}m_{n}l_{B_{0}}\lambda_{i}}{2h}\right)}\equiv \frac{P_{\Delta B_{0}\neq 0}}{P_{\Delta B_{0}=0}}=p_{\Delta B_{0}\neq 0}.$$

If *λ_i_* is the median wavelength and that the flipper is optimally tuned for *π* flips at this wavelength i.e., $\omega_{p}=\pi h/m_{n}l_{B_{0}}\lambda_{i}$ (see Eq. (13)) and setting

(169) $$\xi =\frac{\left|B_{rf}^{eff}\right|}{\left|B_{rf}\right|}=\frac{\sqrt{\omega_{p}^{2}+{\left(\gamma_{n}\Delta B_{0}\right)}^{2}}}{\omega_{p}},$$

Eq. (168) simplifies to

(170) $$p_{\pi ,\Delta B_{0}\neq 0}=\frac{1}{\xi^{2}}\sin^{2}\left(\frac{\pi }{2}\xi \right)$$

for the median wavelength, which we assume is approximately true for the entire incident wavelength band. (Note that Eq. (170) is analogous to Eq. (49).) If we assume that *ΔB_0_* is sufficiently small that cose ε ≈ 1 and *〈*cos*Δφ_π_〉 ≈* 1-*〈Δφ_π_*^2^*〉*/2 = cos*〈Δφ_π_* (rms)*〉*, we have after one *π* coil due to the effect of *ΔB_0_*:

(171) $$\Delta \varphi_{\pi }\approx \cos^{-1}\left(p_{\pi ,\Delta B_{0}\neq 0}\right)=\cos^{-1}\left[\frac{1}{\xi^{2}}\sin^{2}\left(\frac{\pi }{2}\xi \right)\right].$$

For an *M*-coil unit spectrometer, we assume that *Δφ_π_ is* uncorrelated between coils and that the cumulative effect for *M* coils is obtained by summing in quadrature. Taking FWHM values, we have (by combining Eqs. (163) and (171)):

(172) $$\Delta \varphi_{NRSE}^{FWHM}\approx \sqrt{M}\cos^{-1}\left(\frac{1}{\xi^{2}}\sin^{2}\left(\frac{\pi }{2}\xi \right)\right)=4\sqrt{\ln\;2\;\ln\left(\frac{1}{P_{x}^{0}}\right)}$$

and hence

(173) $$P_{x}^{0}{|}_{\Delta l_{B_{0}}=0,\Delta \theta =0}=\exp(-\frac{M}{16\ln2}{\left[\cos^{-1}\{\frac{1}{\xi^{2}}\sin^{2}(\frac{\pi }{2}\xi )\}\right]}^{2}).$$

Specifically for a 4-*N* coil instrument we have:

(174) $$P_{x}^{0}{|}_{\Delta l_{B_{0}}=0,\Delta \theta =0}=\exp\left(-\frac{N}{4\ln2}{\left[\cos^{-1}\left\{\frac{1}{\xi^{2}}\sin^{2}\left(\frac{\pi }{2}\xi \right)\right\}\right]}^{2}\right).$$

In the range of operation of spectrometer configurations considered in this document, it can be shown that the value of *ξ* is typically no greater than about 1.3. In this range, it turns out that the awkward term ${\left[\cos^{-1}\left\{\frac{1}{\xi^{2}}\sin^{2}\left(\frac{\pi }{2}\xi \right)\right\}\right]}^{2}$ may be replaced very successfully by 4(*ξ*−1) so that

(175) $$P_{x}^{0}{|}_{\Delta l_{B_{0}}=0,\Delta \theta =0}=\exp\left(-\frac{M}{4\ln2}(\xi -1)\right),\;\text{for}\;\xi \leq 1.3,$$

or specifically for a 4-*N* coil instrument:

(176) $$P_{x}^{0}{|}_{\Delta l_{B_{0}}=0,\Delta \theta =0}=\exp\left(-\frac{N}{\ln2}(\xi -1)\right),\;\text{for}\;\xi \leq 1.3,$$

**Fig. 12:**
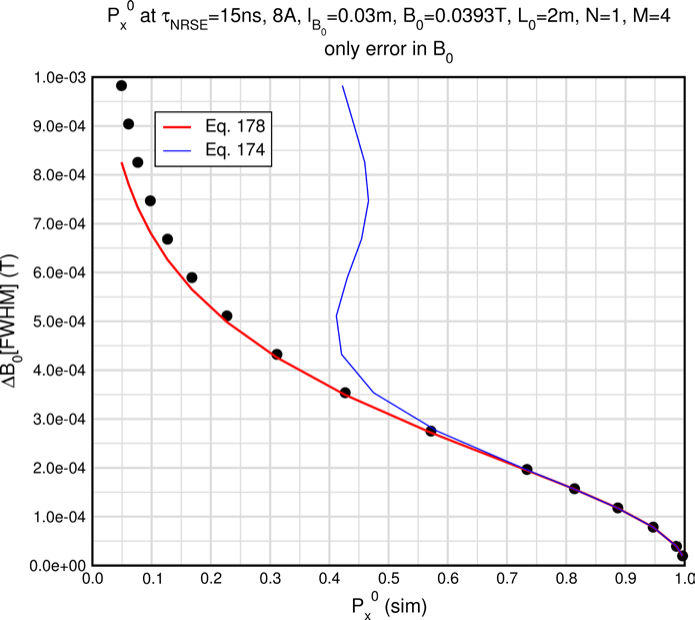
Simulated and analytical predictions of the effect of *ΔB_0_* on the elastic polarization *P_x_^0^* for *τ_NRSE_* = 15 ns at 8 Å, *N* = 1, with other spectrometer parameters given in the plot title. The blue curve represents Eq. (174) which is only valid for moderate to small *ΔB_0_.* The red curve represents the approximation obtained using 4(*ξ*-1) for the inverse cosine squared part of the exponential in Eq. (174) which just so happens to give a better approximation at larger *ΔB_0_* (Eqs. (176) and (178)).

**Fig. 13:**
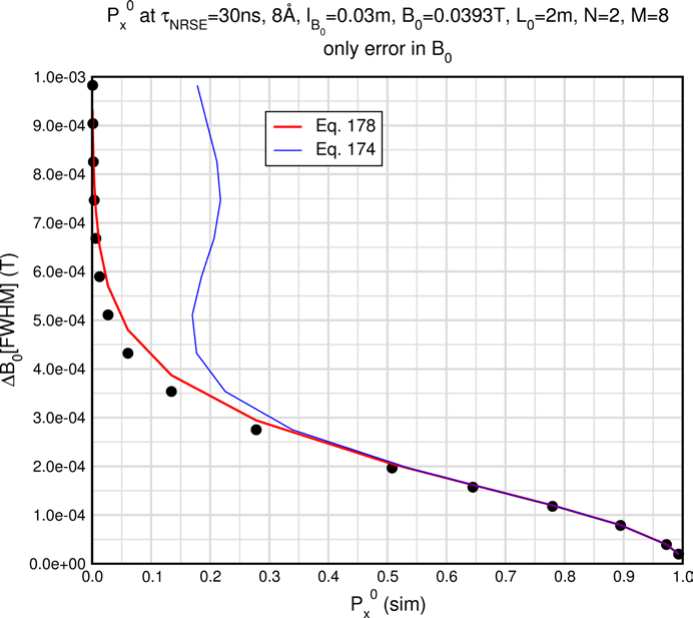
Simulated and analytical predictions of the effect of *ΔB_0_* on the elastic polarization *P_x_^0^* for *τ_NRSE_* = 30 ns at 8 Å, *N = 2*, with other spectrometer parameters given in the plot title. The blue curve represents Eq. (174) which is only valid for moderate to small *ΔB_0_.* The red curve represents the approximation obtained by 4(*ξ*-1) for the inverse cosine squared part of the exponential in Eq. (174) which just so happens to give a better approximation at larger *ΔB_0_* (Eqs. (176) and (178)).

It turns out, somewhat fortuitously, that Eqs. (175) and (176) produce a better approximation to *P_x_^0^* when *ΔB_0_* is too large to assume cos*ε* ≈ 1 (implicit in Eqs. (173) and (174)) or when the accumulated dephasing in the *x-y* plane approaches 2*π.* The approximations in Eqs. (175) and (176) make the inverse problem significantly more tractable (i.e., what tolerance on *ΔB_0_* is required to obtain a given value *of P_x_^0^* under a given set of conditions *λ_0_*, *B_0_* etc.?). The inverse expression, which is more useful in instrument design than the forward expression (Eq. (173)), then finally reduces to

(177) $$\begin{array}{l} \Delta B_{0}^{FWHM}|\Delta_{l_{B_{0}}=0,\Delta \theta =0}\approx \left|B_{rf}\right|\sqrt{{\left(1+\frac{4\ln\;2\;\ln\left(1/P_{x}^{0}\right)}{M}\right)}^{2}-1}\\ =|B_{rf}|\sqrt{\frac{4\ln\;2\;\ln\left(1/P_{x}^{0}\right)}{M}\left(\frac{4\ln\;2\;\ln\left(1/P_{x}^{0}\right)}{M}\right)+2} \end{array}$$

for *M* coils. Specifically for a 4-*N* coil instrument we have:

(178) $$\Delta B_{0}^{FWHM}|\Delta_{l_{B_{0}}=0,\Delta \theta =0}\approx \left|B_{rf}\right|\sqrt{\frac{\kappa }{N}\left(\frac{\kappa }{N}+2\right)},$$

where *κ* = ln 2 ln(1/*P_x_*^0^). The success of Eq. (178) is demonstrated in Fig. 12 and Fig. 13 for *N* = 1 and *N* = 2 respectively for a spectrometer setting with *B_0_* = 0.0393 T, *l_B0_* = 0.03 mm, *L_0_* = 2 m, *λ_0_* = 8 Å, which gives *τ_NRSE_* = 15 ns and 30 ns for *N* = 1 and *N* = 2 respectively.

### 6.2 Coil Flatness

In order to estimate tolerances on the flight path lengths, we return to the expanded equations representing *φ_0_* (and *φ_1_*) which contain the individual contributions (the flatness model used is that described in Sec. 3.6), and now we assume *ΔB_0_* = 0.

**Fig. 14:**
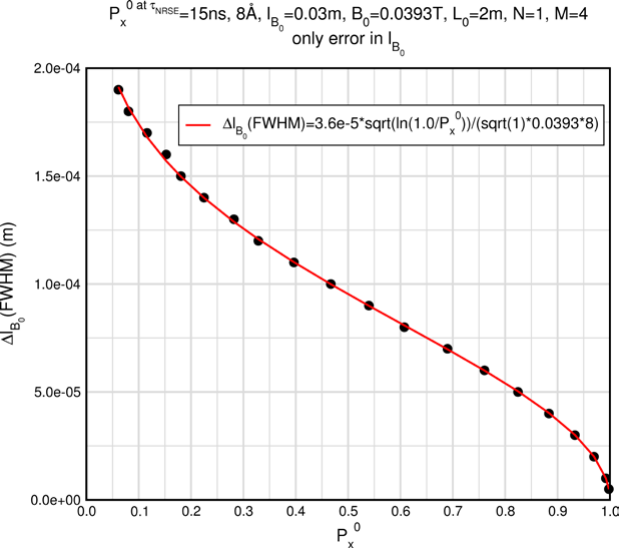
Simulated (black circles) and analytical predictions (Eq. (184) – red curve) of the effect of Gaussian fluctuations of the *π*-flipper length resulting from independent Gaussian fluctuations of the flatness of the windings on the entrance and exit sides of the coil. In this example, *N* = 1, *τ_NRSE_* = 15 ns and the other spectrometer parameters are given in the plot title.

**Fig. 15:**
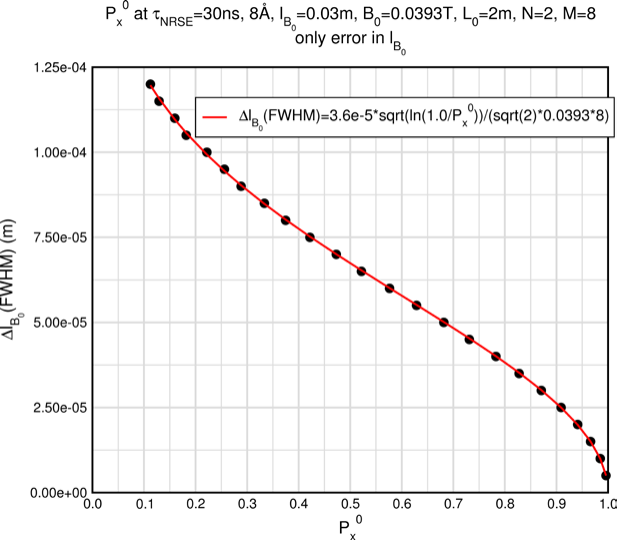
Simulated (black circles) and analytical predictions (Eq. (184) – red curve) of the effect of Gaussian fluctuations of the *π* -flipper length resulting from independent Gaussian fluctuations of the flatness of the windings on the entrance and exit sides of the coil. In this example *N* = 2, *τ_NRSE_* = 30 ns and the other spectrometer parameters are given in the plot title.

- For a 4 (*N* = 1)-coil NRSE, we have from Eq. (72) for a given neutron trajectory,
  $$\varphi_{0}=\frac{\omega_{rf}}{v_{n}}\left[2L_{0}+\Delta f_{R}(B)+\Delta f_{L}(B)-\Delta f_{L}(A)-\Delta f_{R}(A)\right]$$
  where we have set *φ_in_* = 0 (perfectly polarized incoming beam) and the terms *Δf_L_* and *Δf_R_* are the deviations of the coil surface from perfect flatness on the left and right hand sides of the coil respectively. Assuming, for similar coils, *Δf_L_* and *Δf_R_* have Gaussian distributions of equal FWHM *=Δf^FWHM^*, we can write
  (179) $$\frac{\Delta \varphi_{0}}{\varphi_{0}}=\frac{\sqrt{4N}\Delta f^{FWHM}}{2NL_{0}}=\frac{\Delta f^{FWHM}}{L_{0}}\;N=1$$
  whence
  (180) $$\Delta f_{FWHM}=\frac{L_{0}}{\sqrt{2}}\frac{\Delta \varphi_{NRSE}^{FWHM}}{\varphi_{0}}=\frac{h}{m_{n}\gamma_{n}}\frac{\sqrt{2\;\ln\;2\;\ln\left(\frac{1}{P_{x}^{0}}\right)}}{B_{0}\lambda_{i}}\approx \frac{2.54\times 10^{-5}\sqrt{\ln\left(\frac{1}{P_{x}^{0}}\right)}}{B_{0}[T]\lambda_{i}\left[Å\right]}\text{meters}.$$
  We can also write the FWHM fluctuation in the coil length, *Δl_B0_*, in terms of *Δf^FWHM^*, where
  (181) $$\begin{array}{l} \Delta l_{B_{0}}^{FWHM}|{}_{\Delta B_{0}=0,\Delta \theta =0}=\sqrt{2}\Delta f_{FWHM}=L_{0}\frac{\Delta \varphi_{NRSE}^{FWHM}}{\varphi_{0}}=\frac{2h}{m_{n}\gamma_{n}}\frac{\sqrt{\ln\;2\;\ln\left(\frac{1}{P_{x}^{0}}\right)}}{B_{0}\lambda_{i}}\\ \approx \frac{3.6\times 10^{-5}\sqrt{\ln\left(\frac{1}{P_{x}^{0}}\right)}}{B_{0}[T]\lambda_{i}\left[Å\right]}\text{meters}. \end{array}$$
- For *N*=2 bootstrap coils, for a given neutron trajectory, we have from Eq. (73):
  $$\begin{array}{l} {\varphi^{\prime }}_{B_{2}}=\frac{\omega_{rf}}{v_{n}}\left(\begin{array}{l} 4L_{AB}+4l_{g}+8l_{B_{0}}-\Delta f_{L}\left(A_{1}\right)-\Delta f_{R}\left(A_{1}\right)-\Delta f_{L}\left(A_{2}\right)-\Delta f_{R}\left(A_{2}\right)\\ +\Delta f_{L}\left(B_{1}\right)+\Delta f_{R}\left(B_{1}\right)+\Delta f_{L}\left(B_{2}\right)+\Delta f_{R}\left(B_{2}\right) \end{array}\right)\\ =\frac{\omega_{rf}}{v_{n}}\left(4L_{0}-\Delta f_{L}\left(A_{1}\right)-\Delta f_{R}\left(A_{1}\right)-\Delta f_{L}\left(A_{2}\right)-\Delta f_{R}\left(A_{2}\right)+\Delta f_{L}\left(B_{1}\right)+\Delta f_{R}\left(B_{1}\right)+\Delta f_{L}\left(B_{2}\right)+\Delta f_{R}\left(B_{2}\right)\;\right) \end{array}$$
  where we have set *φ_in_* = 0 (perfectly polarized incoming beam) and the terms *Δf_L_* and *Δf_R_* are the deviations of the coil surface from perfect flatness on the left and right hand sides of the coil respectively. Assuming, for similar coils, that *Δf_L_* and *Δf_R_* have Gaussian distributions of equal FWHM = *Δf^FWHM^*, we can write
  (182) $$\frac{\Delta \varphi_{0}}{\varphi_{0}}=\frac{\sqrt{4N}\Delta f_{FWHM}}{2NL_{0}}=\frac{\Delta f_{FWHM}}{\sqrt{N}L_{0}},\;N=2,$$
  whence
  (183) $$\Delta f_{FWHM}=L_{0}\frac{\Delta \varphi_{NRSE}^{FWHM}}{\varphi_{0}}=\frac{h}{m_{n}\gamma_{n}}\frac{\sqrt{2\ln2\ln\left(\frac{1}{P_{x}^{0}}\right)}}{\sqrt{N}B_{0}\lambda_{i}}\approx \frac{2.54\times 10^{-5}\sqrt{\ln\left(\frac{1}{P_{x}^{0}}\right)}}{\sqrt{N}B_{0}[T]\lambda_{i}\left[Å\right]}.$$
  We can also write the FWHM fluctuation in the coil length (length of the *B_0_* field) in terms of *Δf^FWHM^*, where
  (184) $$\Delta l_{B_{0}}^{FWHM}|{}_{\Delta B_{0}=0,\Delta \theta =0}=\sqrt{2}\Delta f_{FWHM}=\frac{2h}{m_{n}\gamma_{n}}\frac{\sqrt{\ln2\ln\left(\frac{1}{P_{x}^{0}}\right)}}{\sqrt{N}B_{0}\lambda_{i}}\approx \frac{3.6\times 10^{-5}\sqrt{\ln\left(\frac{1}{P_{x}^{0}}\right)}}{\sqrt{N}B_{0}[T]\lambda_{i}\left[Å\right]}\text{meters}.$$
  Inverting Eq. (184) we also have
  (185) $$P_{x}^{0}|{}_{\Delta B_{0}=0,\Delta \theta =0}\approx \exp\left[-\frac{N}{4\ln2}{\left(\frac{m_{n}\gamma_{n}}{h}B_{0}\lambda_{i}\Delta l_{B_{0}}^{FWHM}\right)}^{2}\right].$$
  Equation (184) seems to be generally valid, the “√*N*” not being apparent in the *N* = 1 case (Eq. (181)). The success of Eq. (184) is demonstrated in Fig. 14 and Fig. 15 for *N* = 1 and *N* = 2 respectively.

### 6.3 Coil Parallelism

Related to the coil flatness is the question of parallelism, which may actually impose the major engineering limitation. The tolerances on the coil length are the same as indicated in Sec. 6.2, however, a lack of parallelism leads to a predictable and continuous change of field paths over the beam area. If we assume that Eq. (184) defines approximately the maximum tolerance in the static field length, we can approximate the coil parallelism tolerance by

(186) $$\vartheta_{\max}^{surf}=\frac{1}{\max(a,l_{axial}\;)}\frac{2h}{m_{n}\gamma_{n}}\frac{\sqrt{\ln2\ln\left(\frac{1}{P_{x}^{0}}\right)}}{\sqrt{N}B_{0}\lambda_{i}}\approx \frac{1}{\max(a,l_{axial}\;)[m]}\frac{3.6\times 10^{-1}\sqrt{\ln\left(\frac{1}{P_{x}^{0}}\right)}}{\sqrt{N}B_{0}[T]\lambda_{i}\left[Å\right]}\text{rad},$$

where $\vartheta_{\max}^{surf}$ is the maximum tolerable angle between the entrance and exit surfaces of the static coil windings and *a* and *l_axial_* are the coil dimensions defined in Fig. 23.

### 6.4 Beam Divergence (Simplified Model)

**Fig. 16:**
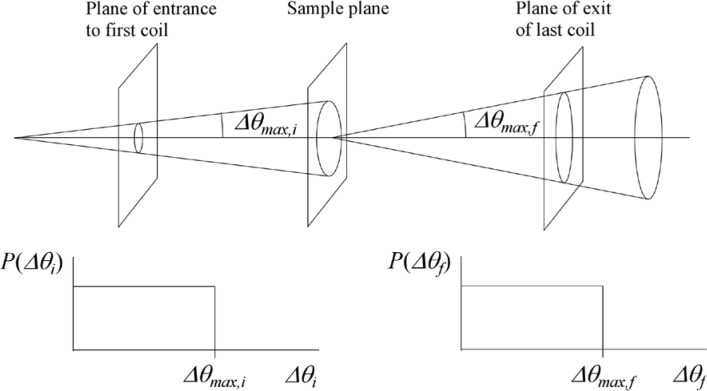
Essentials of the “simplified” divergence model.

We use a simplified model in order to estimate analytically the effects of beam divergence on the elastic resolution (polarization). More realistic beam divergence models, which are treated numerically, are described in Sec. 8.5. The simplified model assumes that the spectrometer components (coil boundaries, samples, etc.) are described by thin planes perpendicular to a nominal beam direction. A divergent incident or scattered beam is represented by selecting random trajectory polar angles, *Δθ_i_* or *Δθ_f_* up to specified maxima *Δθ_i,max_* and *Δθ_f,max_* respectively, where all *Δθ are* defined with respect to any axis parallel to the nominal beam axis. *Δθ_i_* and *Δθ_f_* are assumed to affect all path lengths upstream and downstream of the sample plane respectively. This situation is illustrated in Fig. 16. Therefore, the effect of beam divergence is to increase *all* distances between planes normal to the nominal beam axis by the factor 1/*cos*(*Δθ_i,f_*)*.*

In order to isolate the influence of the beam divergence on the elastic resolution one can consider a symmetrical spectrometer at the echo point with no field inhomogeneities such that *B_1_L_1_*=*B_0_L_0_*, 〈*φ_0_*〉=〈*φ_1_*〉, etc. The elastic resolution is still given by Eq. (113), i.e., *P_x_^0^*=〈cos*φ_NRSE_*〉=〈cos(*φ_0_−φ_1_*)〉. We also assume small divergence, which allows one to write $\varphi_{0}=\frac{2Nm_{n}\gamma_{n}B_{0}L_{0}\lambda_{i}}{h}$ etc. (see Sec. 5.1). With these assumptions the expression for *P_x_^0^* simplifies to

(187) $$P_{x}^{0}=\langle \cos\left(\varphi_{0}-\varphi_{1}\right)\rangle =\langle \cos\left(\langle \varphi_{0}\rangle +\Delta \varphi_{0}-\left[\langle \varphi_{1}\rangle +\Delta \varphi_{1}\right]\right)\rangle =\langle \cos\left(\Delta \varphi_{0}-\Delta \varphi_{1}\right)\rangle .$$

For a trajectory in the incident arm of the spectrometer, we have

(188) $$\frac{\Delta \varphi_{0}}{\langle \varphi_{0}\rangle }\approx \frac{\Delta L_{0}}{L_{0}}=\left[\frac{1}{\cos\Delta \theta_{i}}-1\right].$$

The distribution of *Δφ_0_* for random *Δθ* is by no means Gaussian or uniform. Because we assume small divergence (i.e., *Δθ_i,max_* and *Δθ_f,max_* are small – certainly within the range of angles encountered in the NRSE), we write for all incident arm trajectories:

(189) $$\Delta \varphi_{0}\approx \langle \varphi_{0}\rangle \left(1-\cos\Delta \theta_{i}\right)\approx \langle \varphi_{0}\rangle \frac{\Delta \theta_{i}^{2}}{2}$$

and likewise at the echo point:

(190) $$\Delta \varphi_{1}\approx \langle \varphi_{1}\rangle \left(1-\cos\Delta \theta_{f}\right)\approx \langle \varphi_{1}\rangle \frac{\Delta \theta_{f}^{2}}{2}=\langle \varphi_{0}\rangle \frac{\Delta \theta_{f}^{2}}{2}.$$

(Note *Δφ_0_* and *Δφ_1_* are not necessarily small numbers because 〈*φ_0_*〉 can be very large). Therefore, finally

(191) $$P_{x}^{0}|{}_{\Delta B_{0}=0,\Delta l_{\pi }=0}\approx \langle \cos\left(\frac{\langle \varphi_{0}\rangle }{2}\left[\Delta \theta_{i}^{2}-\Delta \theta_{f}^{2}\right]\right)\rangle \;\left(\text{small divergence},\;\text{at echo point},\;\text{only angular uncertainties}\right).$$

The average in Eq. (191) can be expressed in terms of the double integral over the range of *Δθ_i_* and *Δθ_f_* which are both assumed to be uniform in probability in the range (0, *Δθ_i,max_*), (0, *Δθ_f,max_*), permitting the average to be written simply as

(192) $$P_{x}^{0}|{}_{\Delta B_{0}=0,\Delta l_{\pi }=0}\approx \frac{\int_{0}^{\Delta \theta_{f,\max}}\int_{0}^{\Delta \theta_{i,\max}}\cos\left(\frac{\langle \varphi_{0}\rangle }{2}\left[\Delta \theta_{i}^{2}-\Delta \theta_{f}^{2}\right]\right)\;d\Delta \theta_{i}d\Delta \theta_{f}}{\Delta \theta_{i,\max}\Delta \theta_{f,\max}}.$$

It can be shown that Eq. (192) reduces to

(193) $$P_{x}^{0}|{}_{\Delta B_{0}=0,\Delta l_{\pi }=0}\approx \frac{\pi }{\langle \varphi_{0}\rangle }\frac{\left\{C_{1}\left(\sqrt{\frac{\langle \varphi_{0}\rangle }{\pi }}\Delta \theta_{i,\max}\right)C_{1}\left(\sqrt{\frac{\langle \varphi_{0}\rangle }{\pi }}\Delta \theta_{f,\max}\right)+S_{1}\left(\sqrt{\frac{\langle \varphi_{0}\rangle }{\pi }}\Delta \theta_{i,\max}\right)S_{1}\left(\sqrt{\frac{\langle \varphi_{0}\rangle }{\pi }}\Delta \theta_{f,\max}\right)\right\}}{\Delta \theta_{i,\max}\Delta \theta_{f,\max}},$$

where *C_1_* and *S_1_* are the Fresnel cosine and sine integrals respectively, defined by

(194) $$\begin{array}{l} C_{1}(x)=\int_{0}^{x}\cos\left(\frac{\pi }{2}t^{2}\right)dt\\ S_{1}(x)=\int_{0}^{x}\sin\left(\frac{\pi }{2}t^{2}\right)dt \end{array}$$

and

(195) $$\langle \varphi_{0}\rangle \approx \varphi_{0}=\frac{2Nm_{n}\gamma_{n}B_{0}L_{0}\lambda_{i}}{h}=9.26418\times 10^{4}NB_{0}[T]L_{0}[m]\lambda_{i}\left[Å\right].$$

Certain approximations for evaluating *C_1_* and *S_1_* have been discussed by Mielenz [19] (note that the *π*/6 term in Eq. 3b of this reference should be multiplied by *x*^3^) and Heald [20]. The integrals can also be evaluated numerically. For the particular case of |*Δθ_i,max_*| = |*Δθ_f,max_*| = |*Δθ_max_*|, Eq. (193) becomes:

(196) $$P_{x}^{0}|{}_{\Delta B_{0}=0,\Delta l_{\pi }=0}\approx \frac{\pi }{\langle \varphi_{0}\rangle }\frac{\left\{C_{1}^{2}\left(\sqrt{\frac{\langle \varphi_{0}\rangle }{\pi }}\Delta \theta_{\max}\right)+S_{1}^{2}\left(\sqrt{\frac{\langle \varphi_{0}\rangle }{\pi }}\Delta \theta_{\max}\right)\right\}}{\Delta \theta_{\max}^{2}},$$

or in terms of the instrument parameters:

(197) $$\begin{array}{l} P_{x}^{0}|{}_{\Delta B_{0}=0,\Delta l_{\pi }=0}\approx \frac{\pi h\Delta \theta_{\max}^{2}}{2Nm_{n}\gamma_{n}B_{0}L_{0}\lambda_{i}}\left\{C_{1}^{2}\left(\sqrt{\frac{2Nm_{n}\gamma_{n}B_{0}L_{0}\lambda_{i}}{\pi h}}\Delta \theta_{\max}\right)+S_{1}^{2}\left(\sqrt{\frac{2Nm_{n}\gamma_{n}B_{0}L_{0}\lambda_{i}}{\pi h}}\Delta \theta_{\max}\right)\right\}\\ (\text{case of}|\Delta \theta_{i,\max}|=|\Delta \theta_{f,\max}|=|\Delta \theta_{\max}|). \end{array}$$

**Fig. 17:**
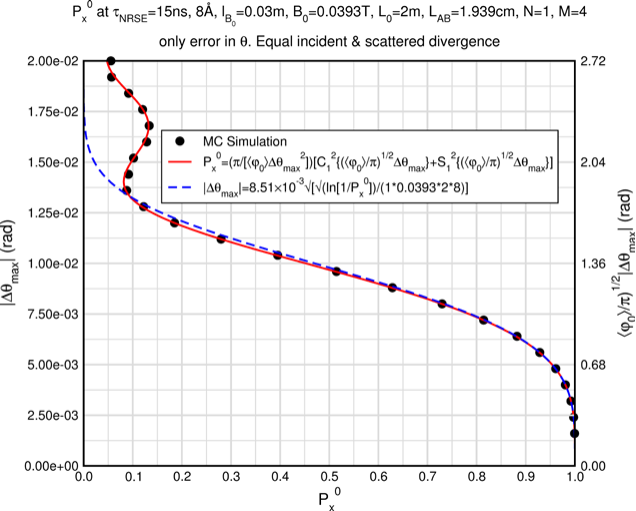
Monte Carlo simulated (black circles) and analytical predictions for the relationship between maximum divergence *Δθ*_max_ (*Δθ* uniformly distributed up to *Δθ*_max_) and *P_x_^0^* for the case *N* = 1, *τ_NRSE_* = 15 ns. The other spectrometer parameters are given in the plot title. The analytical approximation (as expressed by Eq. (193) [Eq. (196) for the particular case of |*Δθ_i,max_*| = |*Δθ_f,max_*| = |*Δθ_max_*|] describes the simulation very well and is represented by the red curve. The approximation (Eq. (202)) that should be valid for |*Δθ_max_| <~* 8.4×10^−3^ rad to within 1 % is shown by the blue dashed curve.

**Fig. 18:**
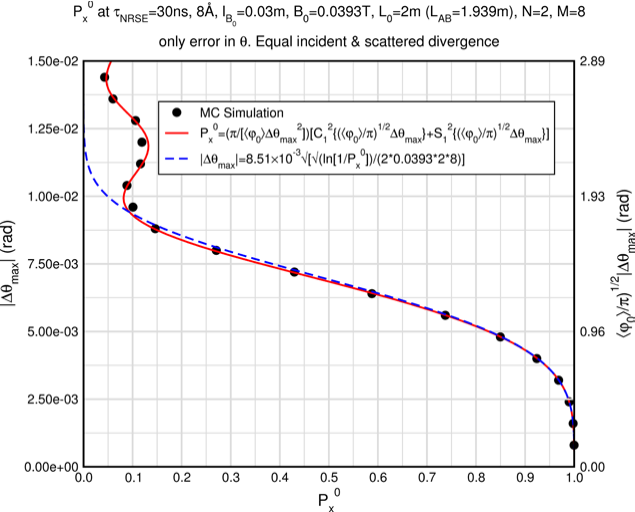
Monte Carlo simulated (black circles) and analytical predictions for the relationship between maximum divergence *Δθ*_max_ (*Δθ* uniformly distributed up to *Δθ*_max_) and *P_x_^0^* for the case *N* = *2, τ_NRSE_* = 30 ns. The other spectrometer parameters are given in the plot title. The analytical approximation (as expressed by Eq. (193) [Eq. (196) for the particular case of |*Δθ_i,max_*| = |*Δθ_f,max_*| = |*Δθ_max_*|]) describes the simulation very well and is represented by the red curve. The approximation (Eq. (202)) that should be valid for |*Δθ_max_| <~* 6.0×10^−3^ rad to within 1 % is shown by the blue dashed curve.

The success of Eq. (193) in describing the relationship between *Δθ_max_* and *P_x_^0^* is demonstrated in Fig. 17 and Fig. 18 for realistic examples. The examples with *τ_NRSE_* = 15 ns, *N* = 1, and *τ_NRSE_* = 30ns, *N = 2* have sufficiently large values of 〈*φ_0_*〉 that the arguments of *C_1_* and *S_1_* exceed unity in the plotted range (the values shown on the right hand side *y*-axes). They also have |*Δθ_i,max_*| = |*Δθ_f,max_*| = |*Δθ_max_*| (so that Eq. (196) is used).

In the present context it is useful to have *P_x_^0^* as the dependent variable and ask “what is the maximum permissible value of |*Δθ_max_*| to achieve a given value of *P_x_^0^*?” Unfortunately, inversion of Eq. (196) is not trivial. The traditional approximations for *C_1_* and *S_1_* discussed in Refs. [19,20] and others do not lend themselves to neat closed forms either, even for small arguments, since the numerator of Eq. (196) involves large powers of the argument for sufficient accuracy. However, we note that the expansion of *C_1_*^2^(*x*)*+S_1_*^2^(*x*) involves terms in *x*^4^*^n^*^+2^, *n* = 0, 1, 2,…, ∞ with alternating signs for the first few terms. Another function that has the same powers and signs as these first terms would be *x*^2^ exp(-*ax*^4^):

(198) $$x^{2}\exp\left(-ax^{4}\right)=\sum_{n=0}^{\infty }\frac{a^{n}x^{4n+2}}{n!}=x^{2}-\frac{ax^{6}}{1!}+\frac{a^{2}x^{10}}{2!}-\frac{a^{3}x^{14}}{3!}+\ldots .$$

The expansion of *C_1_*^2^(*x*)+*S_1_*^2^(*x*) for the first few terms is

(199) $$\begin{array}{l} C_{1}^{2}\left(x\right)+S_{1}^{2}\left(x\right)=x^{2}-0.21932x^{6}+0.020616x^{10}-0.0010163x^{14}-7.7553\times 10^{-5}x^{18}\\ +1.9623\times 10^{-5}x^{22}-1.9468\times 10^{-6}x^{26}+9.7417\times 10^{-8}x^{30}+\ldots , \end{array}$$

therefore we try setting the parameter *a* in Eq. (198) to the magnitude of the second term coefficient in Eq. (199) = *π*^2^/45 ≈ 0.21932 which makes the two leading terms in Eqs. (198) and (199) identical. This should certainly work well for *x <* 1 since the higher order terms decrease rapidly. With this substitution Eq. (198) becomes

(200) $$\begin{array}{l} x^{2}\exp\left(-\frac{\pi^{2}x^{4}}{45}\right)=x^{2}-0.21932x^{6}+0.024051x^{10}-0.0017583x^{14}+9.6405\times 10^{-5}x^{18}+\\ -4.2287\times 10^{-6}x^{22}+1.5457\times 10^{-7}x^{26}-4.843\times 10^{-9}x^{30}+\ldots , \end{array}$$

for which the first few terms are quite similar to those of Eq. (199). It turns out that this approximation can be applied with about 1 % accuracy up to *x ~ x_1%_* ~ 1.15, where the fan-out of the spins due to the divergence (= *Δφ_0_* (see Eq. (189)) ≈ *πx_1%_^2^*/2 ~ 0.7*π*) is still below 2*π* radians, i.e.,

(201) $$C_{1}^{2}\left(x\right)+S_{1}^{2}\left(x\right)\approx x^{2}\exp\left(-\frac{\pi^{2}x^{2}}{45}\right),x<\sim 1.15.$$

In fact the approximation is within 15 % for *x* up to about 1.8, at which point *Δφ_0_ ≈* 1.6*π* (as is seen from Fig. 17 and Fig. 18. Now identifying *x* with $\sqrt{\frac{2Nm_{n}\gamma_{n}B_{0}L_{0}\lambda_{i}}{\pi h}}\Delta \theta_{\max}$, Eq. (197) can be inverted using the approximation in Eq. (201) yielding:

(202) $$\begin{array}{l} \Delta \theta_{\max}{|}_{\Delta B_{0}=0,\Delta l_{\pi }=0}\approx \sqrt{\frac{h\sqrt{45\ln(\frac{1}{P_{x}^{0}})}}{2Nm_{n}\gamma_{n}B_{0}L_{0}\lambda_{i}}}≃8.51\times 10^{-3}\sqrt{\frac{\sqrt{\ln(\frac{1}{P_{x}^{0}})}}{NB_{0}[T]L_{0}[m]\lambda_{i}[\text{Å}]}}[\text{rad}]\\ \text{for}|\Delta \theta_{\max}|<∼1.15\sqrt{\frac{\pi h}{2Nm_{n}\gamma_{n}B_{0}L_{0}\lambda_{i}}}=\frac{6.7\times 10^{-3}}{\sqrt{NB_{0}[T]L_{0}[m]\lambda_{i}[\text{Å}]}}[\text{rad}]. \end{array}$$

The results of this latter approximation are plotted as the blue curves in Fig. 17 and Fig. 18. Although the suggested limits of applicability implied by Eq. (202) (for 1 % accuracy of Eq. (201)) are 8.4 mrad and 6.0 mrad for the *N* = 1 and *N* = 2 cases respectively shown in the figures, the approximation works quite well also for larger angles.

### 6.5 Approximation for Equal Contributions to Depolarization from *ΔB_0_, Δl_B0_*, and *Δθ_max_*

In the preceding sections, the contributions of *ΔB_0_*, *Δl_B0_*, or *Δθ to* the elastic polarization *P_x_^0^* were taken in isolation. Because all three parameters will have some uncertainty, their individual tolerances must be correspondingly tighter to compensate for the depolarization created by the other two. It is difficult to assess which parameter tolerance is easiest to achieve but some idea of the spectrometer requirements is obtained by setting the *ΔB_0_*, *Δl_B0_*, and *Δθ* contributions to the depolarization approximately equal. For *equal contributions*, we assume that the tolerances will be approximately 1/√3 times the values given by Eqs. (178), (184), and (202) respectively (for a 4-*N* coil instrument), i.e.,

(203) $$\Delta B_{0}^{FWHM}\approx \left|B_{rf}\right|\sqrt{\frac{\kappa }{3N}\left(\frac{\kappa }{N}+2\right)}\;(4\text{-}N\;\text{instrument, equal contribs to}\;P_{x}^{0}),$$

where $\kappa =\ln\;2\;\ln\left(1/P_{x}^{0}\right)$, as before. Note that *ΔB_0_* is defined by *N, l_B0_*, and *λ_i_* only and is independent of *B_0_* or zero field region parameters.

(204) $$\Delta l_{B_{0}}^{FWHM}=\frac{2h}{m_{n}\gamma_{n}}\frac{\sqrt{\ln\;2\;\ln\left(\frac{1}{P_{x}^{0}}\right)}}{\sqrt{3N}B_{0}\lambda_{i}}\approx \frac{2.08\times 10^{-5}\sqrt{\ln\left(\frac{1}{P_{x}^{0}}\right)}}{\sqrt{N}B_{0}[T]\lambda_{i}\left[Å\right]}\text{meters}\;(4\text{-}N\;\text{instrument, equal contribs to}\;P_{x}^{0}).$$

Note that *Δl_B0_* is defined by *N, B_0_* and *λ* only and is independent of *l_B0_* or zero field region parameters. Finally,

(205) $$\begin{array}{l} \Delta \theta_{\max}\approx \sqrt{\frac{h\sqrt{45\ln\left(\frac{1}{P_{x}^{0}}\right)}}{6Nm_{n}\gamma_{n}B_{0}L_{0}\lambda_{i}}}\approx 4.91\times 10^{-3}\sqrt{\frac{\sqrt{\ln\left(\frac{1}{P_{x}^{0}}\right)}}{NB_{0}[T]L_{0}[m]\lambda_{i}\left[Å\right]}}[\text{rad}]\\ (4\text{-}N\;\text{instrument, equal contribs to}\;P_{x}^{0}), \end{array}$$

for $\left|\Delta \theta_{\max}\right|<\sim \frac{6.7\times 10^{-3}}{\sqrt{NB_{0}[T]L_{0}[m]\lambda_{i}\left[Å\right]}}[\text{rad}]$.

Note that *Δθ_max_* depends on *λ* and on both the flipper coil and zero-field parameters (*N*, *B_0_*, *L_0_* (i.e., *L_AB_*, *l_B0_*, and *l_g_*)). Even though these parameters also appear in the expression for *τ_NRSE_*, the *λ*^3^-dependence of the latter means that *Δθ_max_* is not uniquely determined by the quantity *τ_NRSE_* (i.e., the same value of *τ_NRSE_* may require different values of *Δθ_max_* depending on the values of *N*, *B_0_*, *L_0_* and *λ*)*.*

### 6.6 Some Examples (Equal Contributions to Depolarization)

Consider requiring the elastic (resolution) polarization *P_x_^0^* to be greater than some specified minimum value at a reference point with equal contributions coming from *ΔB_0_, Δl_B0_*, and *Δθ_max_.* We consider the point *τ_NRSE_ ≈* 30 ns at *λ*= 8 Å with *N* = 2, for *M* = 8 *π* coils (*l_B0_*=0.03 m), with *B_0_* = 0.0393 T, *L_0_* = 2 m. Using Eqs. (203–205), several results are shown in Table 7.

**Fig. 19:**
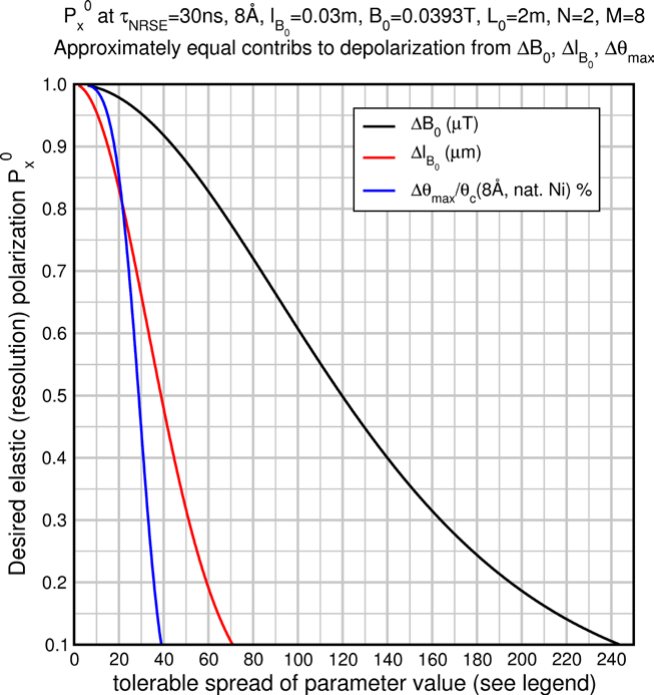
Parameter tolerances (see legend for units) required to achieve a specified minimum elastic (resolution) polarization, *P_x_^0^*, for *τ_NRSE_* = 30 ns at *λ* = 8 Å with the above spectrometer dimensions (*B_0_*=0.0393 T) and approximately equal contributions to the depolarization coming from *ΔB_0_*, *Δl_B0_*, and *Δθ_max_.*

The results in Table 7 are summarized in Fig. 19. Note the particular sensitivity of the instrumental resolution on the beam divergence once a certain threshold angle is reached.

## 7. NIST NRSE Project Goals

### 7.1 Desired Function

Desirable criteria for a NIST NRSE instrument are summarized as follows:

1. Emphasis on quasi-elastic scattering – coil tilting is not necessary.
2. Large solid angle coverage and multi-angle measurement capability.
3. If possible, the spectrometer should be able to access Fourier times of *τ_NRSE_* = 30 ns at *λ* = 8Å and be fabricated with sufficient precision to allow useful measurements to be performed at this measurement point.
4. Offer usable incident wavelengths at least down to 3 Å for high-*Q* capability.
5. Must have a short Fourier time measurement capability.

### 7.2 Spectrometer Dimensions and Field Magnitudes Required to Access *τ_NRSE_* = 30 ns at *λ* = 8 Å

From Eq. (120) we have

$$\tau_{NRSE}[\text{ns}]=0.37271N\;B_{0}[T]L_{0}[m]\lambda_{i}{\left[Å\right]}^{3},$$

where we assume that *B_0_* = *B_1_* so that *L_0_* = *L_1_* at the QENS echo point. In order to access *τ_NRSE_* = 30 ns at *λ* = 8 Å, we must satisfy the condition

(206) $$N{\left(B_{0}[T]L_{0}[m]\right)}_{\max}\geq 0.157\;\text{criterion for accessing}\;\tau_{NRSE}=30\;\text{ns at}\;\lambda =8\text{Å},$$

where (*B_0_L_0_*)*_max_* implies the maximum attainable value of the product *B_0_L_0_.* If we chose *N* = 2 as the most likely bootstrap factor, noting the advantages and disadvantages outlined in Sec. 3.4, this condition amounts to fulfilling:

(207) $${\left(B_{0}[T]L_{0}[m]\right)}_{\max}\geq 0.079\;\text{Tm}\;\text{criterion for accessing}\;\tau_{NRSE}=30\;\text{ns at}\;\lambda =8\text{Å with}\;N=2.$$

Obvious limitations on the maximum value of *B_0_* are imposed by the maximum current × winding density of the static field coils. This depends on the length cross-section, material, winding temperature, and the ability to remove heat. Increasing the zero-field drift path lengths increases proportionately the maximum achievable value of *τ_NRSE_*, however disadvantages include the rapid reduction in solid angle (∝ 1/*L*^2^) and possibly limitations imposed by available space. Owing to these constraints and the linear dependence of *τ_NRSE_* on *B_0_*, it seems reasonable to attempt to maximize the static magnetic field *B_0_* as far as possible. Evaluating *B_0_* and *L_0_* for *τ_NRSE_* =30 ns at *λ_i_* = 〈*λ_i_*〉 = 8Å, we have, for example,

$$\begin{array}{l} B_{0}\approx 0.08\;T,\;L_{0}=1\;m,\;N=2\\ B_{0}\approx 0.04\;T,\;L_{0}=2\;m,\;N=2. \end{array}$$

To date, the largest static fields produced in water-cooled NRSE coils using pure aluminum windings are about *B_0_* ≈ 0.025 T. With this field we require *L_0_* = 3.14 m for *N* = 2 (which is a little long for available floor space) or else *L_0_* = 1.57 m for *N*=4. Apart from the increased restrictions on the maximum incoming bandwidth, *Δλ*/*λ*, when using *N* = 4, doubling the number of *π* flipper coils has the obvious disadvantage of increasing the complexity and setup of the spectrometer and increasing the amount of material in the beam. Thus an *N* = 4 option is unattractive for a multi-angle instrument. Restricting *N* to 2 with *L_0_ ≤* 2 m and pursuing the goal of increasing *B_0_* towards 0.04 T presents itself as one of the more attractive options. Some consequences are explored in the following sections.

### 7.3 Bootstrap NRSE Coil Components and Specifications

#### 7.3.1 General Description

**Fig. 20:**
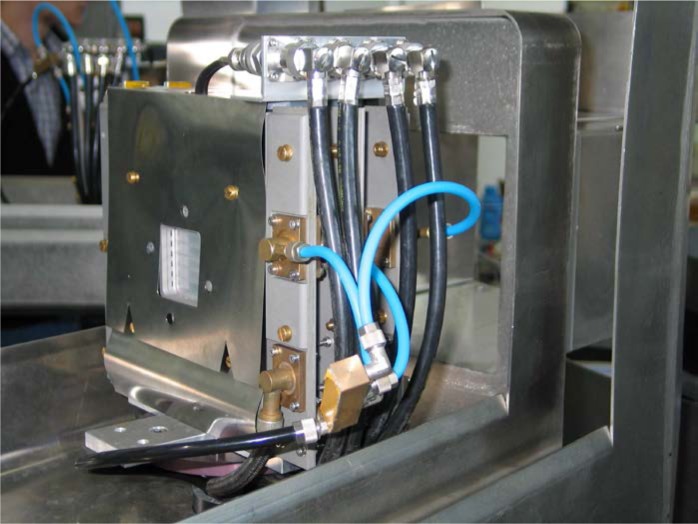
A *N*=2 bootstrap coil on the RESEDA spectrometer at FRM-II. The zero-field flight paths are magnetically shielded by a double-skinned *μ*-metal enclosure (removed). The *μ*-metal screen on the face of the coil, the r.f. coil air cooling connections (blue), and the static field coil water cooling connections (black) are visible (photo kindly allowed by T. Keller, FRM-II).

The *N*=2 bootstrap NRSE coil, a most recent example of which is shown in Fig. 20, is composed of back-to-back static field coils with equal but oppositely-opposed field directions. Each static field coil encloses an r.f coil (whose coil axis is perpendicular to that of the static coil). The r.f coil must be placed inside the static field coil in order to avoid significant r.f. attenuation that would otherwise occur in the metallic structures of the static field coil. *μ*-metal plates capping each end of the static field coils conduct magnetic flux lines between the two coils. An outer *μ*-metal shield enclosing the entire assembly, apart from the beam windows, helps reduce the stray field magnitude entering the zero field regions. For quasielastic applications, both the static and the r.f. coil axes are perpendicular to the beam direction. To profit from the advantages of the NRSE technique over conventional NSE, the NRSE coils must be moderately compact in the beam direction. Because the neutron beam traverses both the static and the r.f. coil windings, there are particular restrictions on the winding materials that may be used in the beam passage (see Sec. 7.3.2). High resolution requirements also impose restrictions on the shape of the windings themselves. These and other factors are discussed in the following sections.

#### 7.3.2 Aluminum Windings: Transmission and Small Angle Scattering

**Fig. 21:**
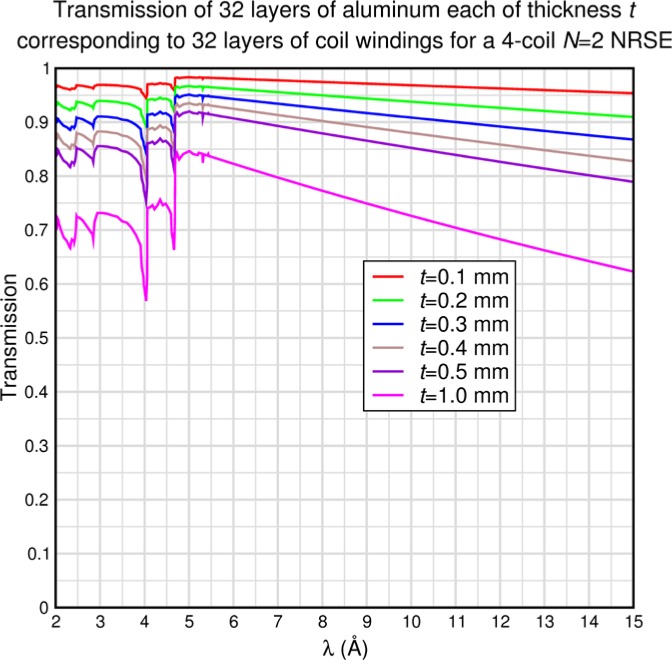
Estimated neutron transmission of the combined 32 layers of windings for a 4-*N* = 2 coil NRSE assuming that the static field and r.f. coil windings each have equal thickness *t*. These curves are based on the total cross-section of pure aluminum at 300 K.

Because the beam must traverse both the static field coil and the r.f. coil windings with this design, the neutronic properties of copper exclude it as a winding material within the beam region. For non-superconducting windings, the most obvious choice is aluminum. However, even pure aluminum has resistivity that is almost 60 % greater than pure copper at room temperature. For a 4-*N* = 2 coil NRSE instrument, the beam must traverse a total of 16*N* = 32 layers of static and r.f. coil windings. Assuming that each winding layer has the same thickness, *t*, we can estimate the anticipated maximum transmission of all the coils from the total cross-section of pure aluminum at room temperature. Some results for different winding thicknesses *t* are shown in Fig. 21. Note that the values in Fig. 21 are optimistic because (i) impurities (e.g. from anodization of the actual winding material) are not accounted for, and (ii) the transmission will be reduced by increased phonon scattering if the operational winding temperature exceeds 300 K (which it is likely to do significantly).

Very approximately, the macroscopic neutron cross-section of aluminum at all temperatures of interest is about 0.11 cm^−1^ for *λ* < 4.7Å. Therefore, we have

(208) $$T_{Al}\sim \exp\left(-0.11\;t[\text{cm}]\right),\;\lambda <4.7Å.$$

Estimating the equilibrium temperature and temperature gradients of the windings depends on the detailed coil design. In order to partially account for elevated winding temperatures at high-field operation of the coils, we approximate the macroscopic cross-section for *λ* > 4.7Å using the average of available data for pure aluminum [21] at *T* = 300 K and at *T* = 800 K. At 300 K data we have approximately

(209) $$\sum_{Al}\left(300K\right)\left[{\text{cm}}^{-1}\right]\approx \left(6.4+8.94\lambda \left[Å\right]\right)\times 10^{-3},\;\lambda \geq 4.7Å$$

and for the 800 K data we have approximately

(210) $$\sum_{Al}\left(800K\right)\left[{\text{cm}}^{-1}\right]\approx \left(1.91+1.175\lambda \left[Å\right]\right)\times 10^{-2},\;\lambda \geq 4.7Å.$$

Therefore, we use an effective aluminum macroscopic cross-section of

(211) $$\sum_{Al}^{eff}\left[{\text{cm}}^{-1}\right]\approx \left(1.28+1.03\lambda \left[Å\right]\right)\times 10^{-2},\;\lambda \geq 4.7Å$$

for the purposes of estimating the coil transmission.

We now assume that the static field coil windings (which usually have to carry higher maximum currents than the r.f windings) have thickness *t* and the r.f windings have thickness *t*/2, such that the total thickness of windings traversed by the beam in the spectrometer is 12*Nt* = 24*t* for *N* = 2. If we choose a transmission criterion such that *T_Al_* (*λ* = 8 Å) ≥ 80 %, then Eq. (211) requires that *t* must not exceed a maximum value, *t_max_*, of about 1.0 mm (i.e., the static field coil windings have thickness of about 1 mm, the r.f. windings have thickness of about 0.5 mm). For the r.f. coils the skin effect at ~1 MHz frequencies likely restricts the r.f. winding thickness to a smaller value (see Sec. 7.3.4.7).

Coils constructed at the Institut Laue-Langevin (ILL), Grenoble, France, Laboratoire Léon Brillouin (LLB), Saclay, France, and the *Forschungs*-Reaktor München-II (FRM-II), Munich, Germany, have used 0.4 mm-thick anodized aluminum band windings, with anodization depth of about 3*μ*m for insulation. The anodization layer can contain incorporated water, which gives rise to strong, anisotropic, small angle scattering. This small angle scattering is greatly reduced by boiling the wire in D_2_O under pressure at about 200 °C [11].

#### 7.3.3 Static Field Coils

**Fig. 22:**
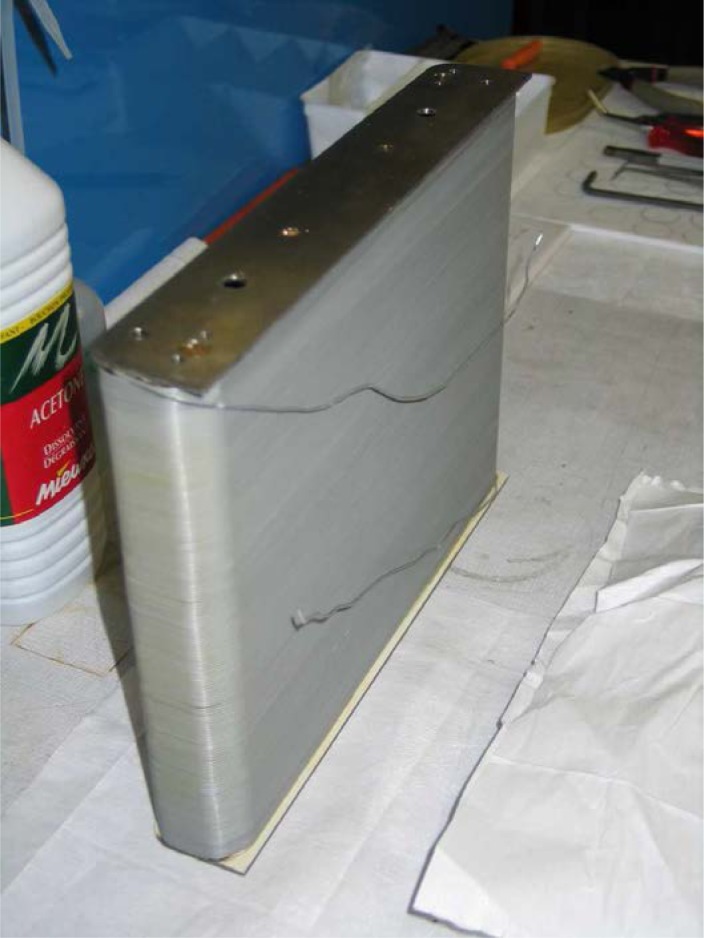
A static field coil developed for the Zeta spectrometer at the ILL, Grenoble using circular section Al wire. This type of coil is used for lower resolution applications. (Photos kindly allowed by R. Gähler, ILL).

An early static field coil using circular section aluminum wire developed for the Zeta spectrometer at the ILL, Grenoble, is shown in Fig. 22.

##### 7.3.3.1 Current in the static field coil

Sufficient static field homogeneity within the beam passage may be achieved by passing the beam through a suitably restricted area close to the axial center of a long solenoid. The field at the center of a long solenoid is

(212) $$B=\mu_{0}nI,$$

where *μ_0_* is the permeability of free space with *μ_0_ =4π ×* 10^−7^ NA^−2^. In SI units we have

(213) $$B_{0}[T]\approx 4\pi \times 10^{-7}n\left[m^{-1}\right]I\left[A\right]≃1.26\times 10^{-6}n\left[m^{-1}\right]I\left[A\right]\;\text{long solenoid approximation},$$

where *B_0_* is the static field in Tesla, *n* is the winding density in m^−1^, and *I* is the current in Amps. Equivalently, the current in the coil at field *B_0_* is

(214) $$I\left[A\right]=\frac{2.5\times 10^{6}}{\pi }\frac{B_{0}\left[T\right]}{n\left[m^{-1}\right]}\approx 8\times 10^{5}\frac{B_{0}\left[T\right]}{n\left[m^{-1}\right]}.$$

Thus the required current is inversely proportional to the winding density and is directly proportional to the required field *B_0_*.

##### 7.3.3.2 Resistance of the static field coil windings

**Fig. 23:**
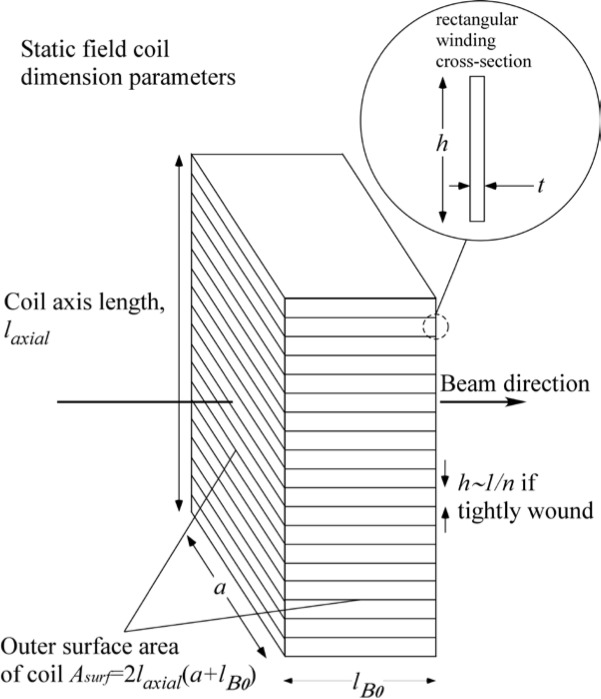
Dimension parameters of the static field coil using tightly-wound rectangular cross-section windings.

The resistance of the static field coil winding is

(215) $$R=\frac{\rho \left(T\right)l_{w}}{A_{w}},$$

where *l_w_* is the total length of the coil winding, *A_w_* is the wire cross-sectional area, and *ρ* (*T*) is the resistivity of the winding at its operating temperature, *T*. The winding length per turn (see Fig. 23) for the rectangular cross-section coil form is approximately 2(*a*+ *l_B0_*), assuming the winding thickness is negligible compared with *a* and *l_B0_*. For the particular case of single-layer windings, the total number of turns, *N_B0_*, is

(216) $$N_{B_{0}}=l_{axial}\;n\;\text{any single-layer winding},$$

so that the total length of any single-layer winding around the rectangular coil form shown in Fig. 23 is

(217) $$l_{w}\approx 2N_{B_{0}}\left(a+l_{B_{0}}\right)=2l_{axial}n\left(a+l_{B_{0}}\right).$$

The outer surface area of the rectangular coil form is

(218) $$A_{surf}=2l_{axial}\left(a+l_{B_{0}}\right),$$

so Eq. (217) may be rewritten as

(219) $$l_{w}=A_{surf}n\;\text{any thin single-layer winding around rectangular coil form}.$$

##### 7.3.3.3 Single-layer rectangular cross-section wire

The cross-sectional area, *A_w_*, of *rectangular* cross-section wire (see Fig. 23) is

(220) $$A_{w}=th\;\text{rectangular cross-section wire},\;\text{width}\;h,\;\text{thickness}\;t,$$

so that, using Eqs. (219) and (220), and noting that for a single winding *h ≤* 1/*n*, with the equality representing the tightly-wound limit, Eq. (215) becomes

(221) $$R=\frac{\rho \left(T\right)n\;A_{surf}}{th}\;\text{any single-layer rectangular cross-section wire},$$

with

(222) $$R=\frac{\rho \left(T\right)n^{2}\;A_{surf}}{t}\;tightly\text{-}wound\;\text{rectangular cross-section wire},\;\text{thickness}\;t\;$$

representing the tightly-wound limit with *h* = 1/*n*. Therefore, for a given *A_surf_*, the resistance of the *tightly-wound* coil increases as the *square* of the winding density and is inversely proportional to the winding thickness, *t*. Logically, the resistance is *minimized* for a given *n*, *A_surf_*, *t*, by ensuring that the windings are tightly-wound.

For a single-layer rectangular cross-section wire winding, the D.C. voltage required to maintain a static field *B_0_* is, from Eqs. (214) and (221)

(223) $$V=IR=\frac{2.5\times 10^{6}}{\pi }\frac{\rho \left(T\right)\left[\Omega m\right]A_{surf}\left[m^{2}\right]}{t[m]h[m]}B_{0}[T]\;\text{any single-layer rectang cross-section wire winding}$$

with

(224) $$V[V]=\frac{2.5\times 10^{6}}{\pi }\frac{\rho \left(T\right)\left[\Omega m\right]n\left[m^{-1}\right]A_{surf}\left[m^{2}\right]}{t[m]}B_{0}[T]\;\text{tightly-wound},\;\text{single-layer},\;\text{rectang cross-section wire windings}$$

representing the tightly-wound limit. Thus, for the tightly-wound case, the voltage required to maintain a field *B_0_* is proportional to *B_0_*, proportional to the winding density, and inversely proportional to the winding thickness in the beam direction for a given coil surface area. For a given *B_0_*, the voltage is *minimized* by tightly-winding the coil within the available surface area.

The power dissipated in the coil with single-layer, rectangular cross-section wire is (from Eqs. (214) and (221) or (223))

(225) $$\begin{array}{l} P[W]={\left(I[A]\right)}^{2}R[\Omega ]\\ \approx \frac{6.25\times 10^{12}}{\pi }\frac{\rho \left(T\right)\left[\Omega m\right]A_{surf}\left[m^{2}\right]}{n[m^{-1}]t[m]h[m]}{\left(B_{0}[T]\right)}^{2}\;\text{any single-layer rectang cross-section wire winding} \end{array}$$

Specifically, for the tightly-wound, rectangular cross-section wire winding it is (from Eqs. (214) and (222) or (224))

(226) $$P[W]\approx \frac{6.25\times 10^{12}}{\pi }\frac{\rho \left(T\right)\left[\Omega m\right]A_{surf}\left[m^{2}\right]}{t[m]}{\left(B_{0}[T]\right)}^{2}\;tightly\text{-}wound\;\text{rectang cross-section wire winding}.$$

Thus, for a given *A_surf_*, the power dissipated in the tightly-wound coil is inversely proportional to the winding thickness, *t*, and is independent *of n or h* (essentially a current sheet). We also note that the power increases as the square of the required field, *B_0_.* Like the voltage, the power dissipated is *minimized* for a given *B_0_* by tightly-winding the coil within the available surface area, since *h ≤* 1/*n.*

##### 7.3.3.4 Single-layer circular cross-section wire windings

The cross-sectional area of the *circular* cross-section wire, *A_w_*, is

(227) $$A_{w}=\pi r_{w}^{2}\;\text{circular cross-section wire of radius}\;r_{w}.$$

Using Eq. (219) and noting that for a single-layer circular winding we have the constraint *n ≤* 1/2*r_w_*, with the equality representing the tightly-wound case, Eq. (215) becomes

(228) $$R=\frac{\rho \left(T\right)n\;A_{surf}}{\pi r_{w}^{2}}\;\text{any circular cross-section wire winding},$$

with

(229) $$R=\frac{4\rho \left(T\right)n^{3}\;A_{surf}}{\pi }\;tightly\text{-}wound\;\text{circular cross-section wire},$$

representing the tightly-wound limit. Thus, for a given *A_surf_*, the *tight-winding* resistance increases as the *cube* of *n* (as opposed to *n*^2^ in the tightly-wound *rectangular* wire case with fixed *t*).

The D.C. voltage required to maintain a static field *B_0_* in the circular cross-section wire case is (from Eqs. (214) and (228))

(230) $$V[V]=2.5\times 10^{-6}\frac{\rho \left(T\right)\left[\Omega m\right]A_{surf}\left[m^{2}\right]}{{\left(\pi r_{w}\left[m\right]\right)}^{2}}B_{0}\left[T\right]\;\text{any single circular cross-section wire winding}.$$

Specifically, for the *tightly-wound* case it is (from Eqs. (214) and (229))

(231) $$\begin{array}{l} V[V]=\frac{10^{7}}{\pi^{2}}\rho \left(T\right)\left[\Omega m\right]{\left(n\left[m^{-1}\right]\right)}^{2}A_{surf}\left[m^{2}\right]B_{0}\left[T\right]\\ =2.5\times 10^{-6}\frac{\rho \left(T\right)\left[\Omega m\right]A_{surf}[m^{2}]}{{\left(\pi r_{w}\left[m\right]\right)}^{2}}B_{0}\left[T\right]\;\text{tightly-wound},\;\text{circular cross-section wire winding}. \end{array}$$

Thus, the voltage required to achieve a given *B_0_* in the circular cross-section wire case is independent of the winding density, other than *n* cannot exceed a value of 1/(2*r_w_*) for a single layer. Qualitatively, this is because decreasing *n* decreases *R* at the same rate that *I* (Eq. (214)) must increase to maintain *B_0_*.

The power dissipated in the coil with single-layer, circular cross-section wire windings is (from Eqs. (214) and (228) or (230))

(232) $$P[W]\approx \frac{6.25\times 10^{12}}{\pi^{3}}\frac{\rho \left(T\right)\left[\Omega m\right]A_{surf}\left[m^{2}\right]}{n\left[m^{-1}\right]{\left(r_{w}[m]\right)}^{2}}{\left(B_{0}[T]\right)}^{2}\;\text{any single circular cross-section wire winding},$$

where the tightly-wound case with *n* = 1/(2*r_w_*) is

(233) $$P[W]\approx \frac{1.25\times 10^{13}}{\pi^{3}}\frac{\rho \left(T\right)\left[\Omega m\right]A_{surf}\left[m^{2}\right]}{r_{w}[m]}{\left(B_{0}[T]\right)}^{2}\;tightly\text{-}wound\;\text{circular cross-section wire winding}.$$

Therefore, the tightly-wound coil represents the *minimum* power condition for circular cross-section wire. Furthermore, the circular wire should be as thick as is tolerable to minimize the power.

##### 7.3.3.5 Summary and static field coil power concerns

The coil flatness requirements for high resolution operation (see Sec. 6.2) favor *rectangular* cross-section wires for the static field coils. Two potential concerns are: (i) the magnitude of the currents supplied to the coils, (ii) excessive heat dissipation in the coils and the associated cooling difficulties. Item (i) is somewhat mitigated by choosing the largest value of *n* that is feasible. Item (ii) is mitigated by tightly-winding the coil as indicated in Sec.7.3.3.3. Beyond these measures Eq. (226) identifies the remaining constraints: Firstly, if *A_surf_* becomes small with respect to the beam area it is increasingly difficult to maintain adequate field homogeneity within this region at high *τ_NRSE_* (see e.g. Sec. 6.1 and Sec. 6.6). Secondly, the winding thickness in the beam direction, *t*, must be limited so as to maintain high neutron transmission (see Sec. 7.3.2). Finally, there are very limited choices of winding material that have both good cold neutron transmission combined with low electrical resistivity. Although maximum fields of a few 10’s of mT do not appear dauntingly high the heat production from the coil is potentially quite large. This is illustrated by the following examples:

The coils produced for the neutron research laboratories Laboratoire Léon Brillouin (LLB), Institut Laue-Langevin (ILL) (France), Forschungs-Neutronenquelle Heinz Maier-Leibnitz (FRM-II), and Helmholtz-Zentrum Berlin (HZB) (Germany) use tightly-wound 4 mm wide × 0.4 mm thick anodized aluminum band supplied by Wesselmann Umwelttechnik^3^, with *n* ≈ 250 m^−1^, *l_axial_* ≈ 0.2 m, *a* + *l_B0_* ≈ 0.25 m for a beam size of about 2.5 cm × 2.5 cm, so that *A_surf_* (see Eq. (218)) ≈ 0.1 m^2^. For these coils at maximum field (*B_0_ ≈* 0.025 T), we have (from Eq. (214)) *I* ≈ 80 A. For pure Al down to about liquid nitrogen temperature, we have

(234) $$\rho_{Al}\left(T\right)\left[\Omega m\right]\approx 1.14\times 10^{-10}T\left(K\right)-6.9\times 10^{-9}.$$

Therefore, specifically for aluminum, we have (from Eq. (226))

(235) $$P_{Al}\left[W\right]\approx {\left(B_{0}\left[T\right]\right)}^{2}\frac{A_{surf}\left[m^{2}\right]}{t\left[m\right]}\left(72.2T\left(K\right)-4.37\times 10^{3}\right)\;\text{tightly-wound rectangular wire windings}.$$

**Fig. 24:**
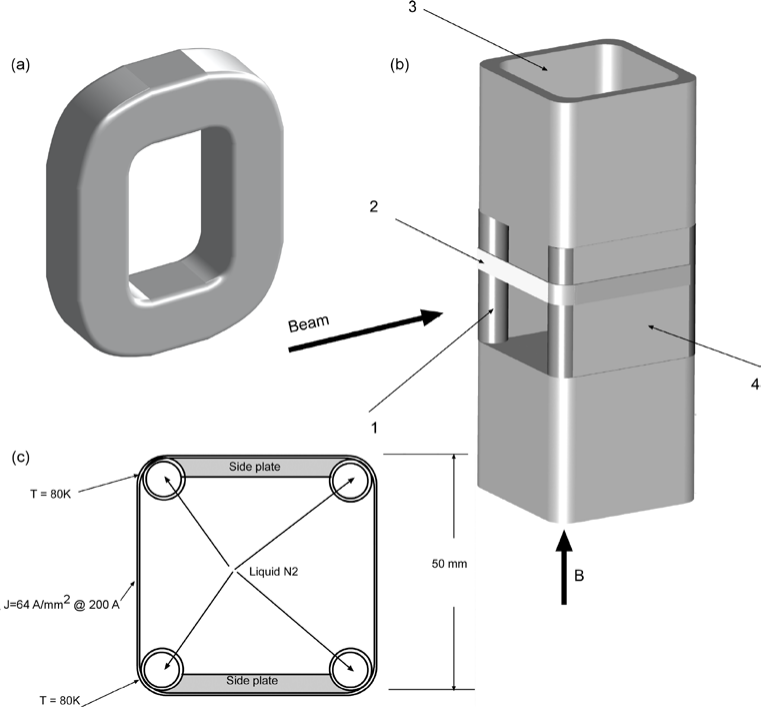
A concept for a liquid N_2_-cooled static field coil. (a) The overall shape of the coil. (b) A section through one side of the coil with beam passage showing: 1. Hollow tubes for passage of N_2_ running the full height of the coil form. 2. The 0.5 × 6.2 mm high purity aluminum conductor that is wound around the coil form with a close spacing of approximately 6.3 mm per turn and the cooling tubes. 3. Hollow interior of the coil form for the N_2_ 4. High purity aluminum side plates providing additional thermal contact and support for the conductor. (c) A cross-sectional view through of the beam passage shown in (b). (Concept and estimated parameters kindly provided by C. Goodzeit of M.J.B. Consulting, De Soto, TX, USA).

For *T ≈* 300 *K*, *P_Al_* (0.025 T) ≈ 2.7 kW. For *T ≈* 350 K, *P_Al_* (0.025 T) ≈ 3.3 kW. If similar coils are to achieve 0.04 Tesla, the current increases to *I* ≈ 128A with an increased power dissipation factor of approximately (0.04^2^/0.025^2^). The room-temperature power dissipation then increases to approximately 6.9 kW. If the coils are cooled to liquid nitrogen temperature ≈ 80 K, *P_Al_* is more than an order of magnitude smaller (≈ 220 W at *B_0_* = 0.025 T, ≈ 560 W at *B_0_* = 0.04 T). This is discussed by Gähler, Golub, and Keller in Ref [8]. One technical challenge is avoiding liquid coolant (water or liquid N_2_) in the beam passage since both scatter thermal neutrons strongly. A separate issue is the evidently undesirable increased beam divergence from small angle scattering that occurs in Aluminum. A concept for a liquid N_2_-cooled static field coil with the above requirements has been proposed by Carl Goodzeit of M.J.B. Consulting, De Soto, TX, USA (Fig. 24). The basic shape of this coil is a racetrack-shaped toroid (Fig. 24(a)). A section of one side of this hollow coil provides the beam passage (Fig. 24(b)) requiring high purity aluminum (99.999 %) conductor. The specific example shown has 0.5 mm thick and 6.2 mm wide conductor which implies *I* ≈ 198 A at *B_0_* = 0.04 T with a corresponding current density of about 64 A mm^−2^. The winding would be supported by and cooled by four hollow tubes for the passage of liquid N_2_ (Fig. 24(c)) running the full height of the coil. On the sides which do not transmit the beam, additional thermal contact and support is provided by heat-conducting side plates. Because the effective resistance of each turn is combined with the resistance of the turns in the remainder of the toroid, all turns, except at beam transit, can be of a lower resistivity material and are in thermal contact with the N_2_-filled coil form, thus they should remain close to 80 K. In general, the liquid N_2_ would be admitted at the bottom of the racetrack coil form and would vent from the top (these features and eventual feed-throughs for the r.f coil are not shown). The coils would be contained in an environment that prevents condensation of water vapor on the windings.

##### 7.3.3.6 Required static field coil current stability

The values in Table 7 imply that *ΔB_0_*/*B_0_* must be around 0.1 % in order to achieve *P_x_^0^*(8 Å, 30 ns) ≥ 0.5 for typical spectrometer dimensions. Even for perfect static field coil homogeneity (*ΔB_0_* = 0), this imposes a coil current stability of order of 0.1 % (*ΔI*/*I <~* 10^−3^). The current stability should certainly not become the limiting factor on *ΔB_0_.* Preferably it should be at least an order of magnitude better *(ΔI*/*I <* 10^−4^). Long-term current drift (e.g. in response to temperature changes) should also be in this range. Current supplies offering stabilities in the 10^−5^ range are commercially-available, so this is not expected to impose any technical limitation.

##### 7.3.3.7 Effect of coil dimensions on field homogeneity and field magnitude

**Fig. 25:**
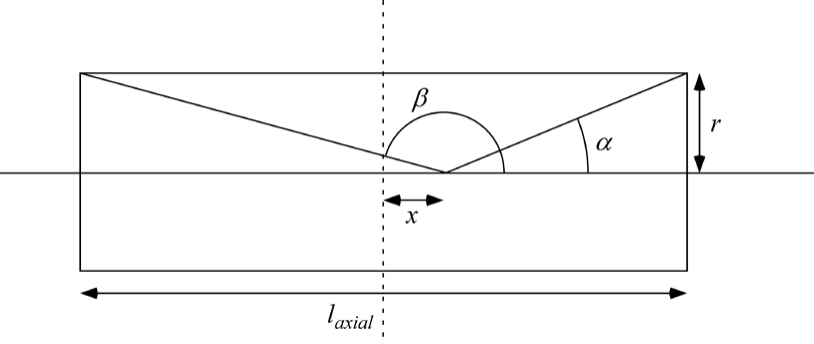
A short circular solenoid.

With respect to geometry, field homogeneity, field strength, and winding resistance, it is preferable that the static field coils be short in the beam direction, given that the coil width must be somewhat wider than the beam. Reducing the coil thickness in the beam direction tends to allow the perpendicular axial length of the coil to be reduced without loss in field homogeneity in the beam passage. This principle is illustrated by considering the axial field *of a cylindrical open-ended* solenoid (Fig. 25), where instead of the coil thickness in the beam direction we refer to the coil radius. The field at axial position *x* is

(236) $$B=\frac{\mu_{0}nI}{2}\left(\cos\alpha -\cos\beta \right)=\frac{\mu_{0}nI}{2}\left[\frac{l_{axial}/2-x}{\sqrt{\left(r^{2}+{\left(l_{axial}/2-x\right)}^{2}\right)}}+\frac{l_{axial}/2+x}{\sqrt{\left(r^{2}+{\left(l_{axial}/2+x\right)}^{2}\right)}}\right].$$

This can be re-expressed in terms of the dimensionless quantities

(237) $$\mu =\frac{2x}{l_{axial}},$$

which is the axial distance from the solenoid center expressed as a fraction of the half-length of the solenoid and

(238) $$\eta =\frac{2r}{l_{axial}},$$

which is the ratio of the diameter, *d*, of the coil to its axial length, so that

(239) $$\frac{B}{\mu_{0}nI}=\frac{1}{2}\left[\frac{1-\mu }{\sqrt{\left(\eta^{2}+{\left(1-\mu \right)}^{2}\right)}}+\frac{1+\mu }{\sqrt{\left(\eta^{2}+{\left(1+\mu \right)}^{2}\right)}}\right].$$

**Fig. 26:**
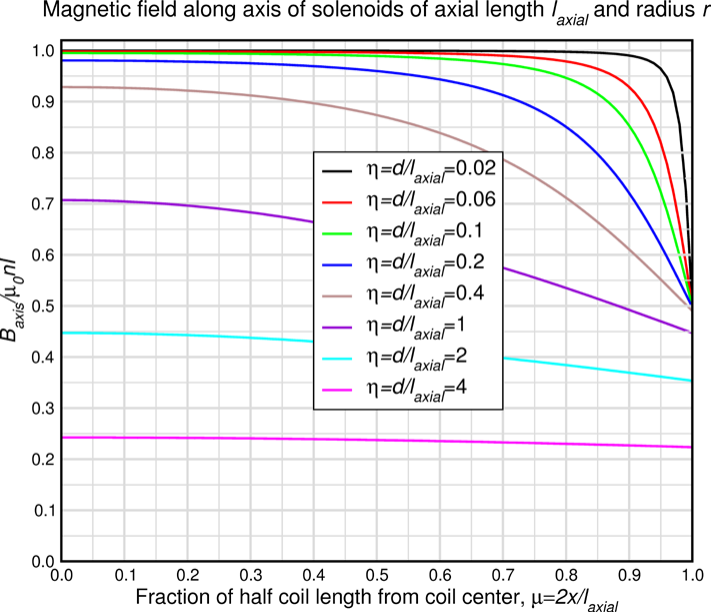
Variation of axial magnetic field of idealized solenoids with various diameter/axial length ratios, *η*.

In the “long” solenoid limit (*l_axial_* ≫ *r*), the field at the coil center is maximized (=*µ0nI*), whereas at its ends it is half this value (=*µ_0_nI*/2 – limit of Eq. (239) with *µ* = 1 and *η*^2^ ≪ (1+*µ*)^2^). This alone implies that the axial length of the coil must be substantially greater than the height of the neutron beam. Figure 26 shows the variation of the axial field normalized to the maximum attainable field (=*µ0nI*) for solenoids with various ratios *η* = *d*/*l_axial_*, calculated according to Eq. (236). Figure 26 reveals that as *η* increases:

- The axial range over which the field can be held close to *B*(*x* = 0) decreases.
- The maximum achievable field (at the center) decreases. This reduction becomes quite significant once *η* increases above about 0.4.

This latter consideration is particularly important in the present application where the goal of achieving the highest fields is already hampered by large currents. Usually, detailed field calculations are required to optimize the coil windings and dimensions. Using the example of the cylindrical solenoid, suppose the maximum axial length of the static field coil is 0.3 m, the beam height is 0.03 m, and the required *ΔB_0_*/*B_0_* is about 0.1 %. With reference to Fig. 26, this requires *B_0_*(*µ* = 0.1) ≥ 0.999*B_0_*(*µ* = 0). This occurs for *η* <~ 0.045, i.e., for coil diameters of 0.0135 m or less. Although this example just considers the axial field variation for a cylindrical solenoid, it suggests that careful control of the coil dimensions perpendicular to the coil axis are required to achieve sufficient field homogeneity within the beam passage of the NRSE coils.

##### 7.3.3.8 Coil flatness issues

As demonstrated in Sec. 6 the dimensional tolerances for high resolution coils are demanding. The winding support must be accurately machined and the windings themselves must be very flat. The use of anodized pure aluminum band not only creates a geometrically well-defined field region but also eliminates curved field lines that are generated in the vicinity of circular cross-section wires (Dubbers *et al.* [22]). The existence of a magnetic pressure (see Sec. 7.3.3.10) is also of concern for maintaining the shape of the windings. For high fields this usually requires clamping of the windings outside of the beam passage.

##### 7.3.3.9 Winding methods

Commercial coil winding tools are available, however, good experience has been obtained using machinist’s lathes. These machines offer the desirable combination of precise translational and rotational speeds, and adjustable torque settings.

##### 7.3.3.10 Magnetic pressure on the coil windings and their mechanical constraint

Magnetic pressure in a coil refers to the radial force exerted on the coil windings due to the difference in magnetic flux density inside and outside the coil. The magnetic pressure, *P_mag_*, exerted on the windings at the center of a long circular solenoid is

(240) $$P_{mag}=\frac{B^{2}}{2\mu_{0}}$$

from which we have

(241) $$P_{mag}[{\text{Nm}}^{-2}]=\frac{{\left(B[T]\right)}^{2}}{8\pi \times 10^{-7}}\approx 4\times 10^{5}{\left(B[T]\right)}^{2}.$$

For *B* = 0.04 T, *P_mag_* ≈ 637 Nm^−2^ (≈ 0.0063 Atm).

For the approximately rectangular section coils used in NRSE, Ampere’s law predicts that the magnitude of the field inside the coil is similar to that of a cylindrical coil carrying the same current, assuming that the field outside the coil is negligible with respect to that inside. Therefore, we assume that the magnetic pressure is also given by Eq. (241) near the center of a long rectangular section coil.

For a coil wound on a rectangular former with slight pre-tension, we can approximate the action of the magnetic pressure on the band-like windings by the mechanical problem of an evenly-loaded beam whose ends are constrained. According to Ref. [23], the maximum winding deflection at the center is

(242) $$y_{\max}=\frac{wl_{u}^{4}}{384EI},$$

where *w* is the load per unit length of the beam, *l_u_* is the unconstrained length of the beam, *E* is Young’s modulus for the winding material, and *I* is the moment of inertia. For the band-like (rectangular) windings of width *h* and (small) thickness *t*, the moment of inertia is

(243) $$I=\frac{ht^{3}}{12}.$$

The load per unit length is

(224) $$w=P_{mag}h,$$

so that Eq. (242) can be re-expressed as

(245) $$y_{\max}=\frac{P_{mag}l_{u}^{4}}{32Et^{3}},$$

i.e.,

(246) $$y_{\max}[m]\approx 1.25\times 10^{4}\frac{{\left(B[T]\right)}^{2}l_{u}{\left[m\right]}^{4}}{E[{\text{Nm}}^{-2}]t{\left[m\right]}^{3}}.$$

For pure Al windings (*E* = 7.1×10^10^ Nm^−2^) with *t* = 0.4 mm (as used in existing coils), *B* = 0.04 T, and a typical *l_u_* for the coil face traversed by the beam of about 0.25 m, we have *y_max_* ≈ 17 mm. This is clearly unacceptably large, therefore in order to maintain the coil dimensions within required tolerances, the windings must be clamped for high fields. Note that Eq. (245) contains the unconstrained length of the winding to the 4th power, therefore it is often feasible for the clamping plates to incorporate an open window allowing passage of the beam (see Fig. 20). For example, if this window is 0.03 m wide, then *l_u_* ≈ 0.03 m and Eq. (245) yields *y_max_* ≈ 3.5 *µ*m, which is well within the acceptable range (Table 7).

#### 7.3.4 r.f. Coils

##### 7.3.4.1 Existing r.f. coil designs

**Fig. 27:**
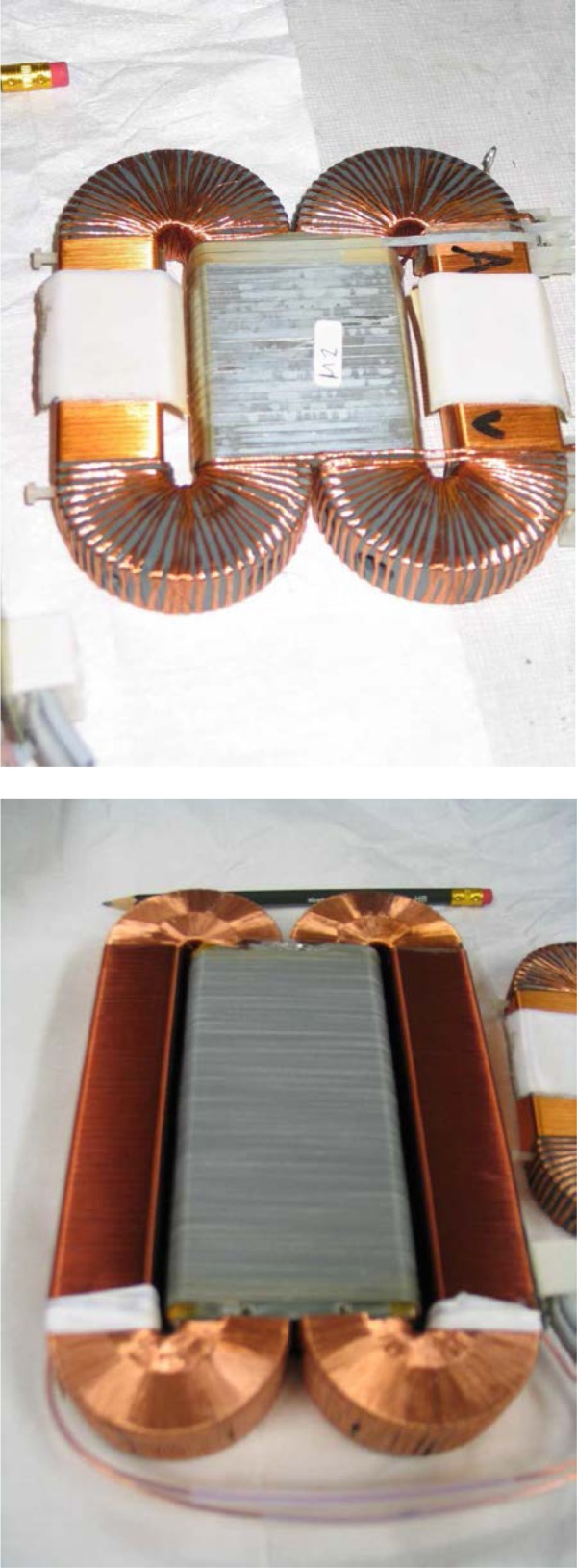
Two r.f. coils developed at the ILL. Top (smaller) uses anodized pure Al tape for the windings in the beam area. The larger model (bottom) uses circular section anodized pure Al wire. (Photos kindly allowed by R. Gähler, ILL, Grenoble).

The r.f. coils, two examples of which are shown in Fig. 27, are of similar overall design with the beam passing through the (gray) aluminum windings at the coil center. The fields are returned through the two arch-shaped coils which greatly reduce r.f. power loss from induced currents in (and consequent heating of) surrounding metallic structures, including the static field coils. This also prevents significant perturbations to the static field. The coils outside of the beam passage are wound with high-frequency (very thin stranded) copper wire to maximize electrical conductivity. The electrically-insulating return coil former material in the units shown is similar to the fiberglass/epoxy composite used in printed circuit boards. The method used at the ILL for maintaining tension on the aluminum windings is to stretch the windings over silicon-based rubber O-rings covered with Kapton tape (rubber containing carbon has been found to burn).

##### 7.3.4.2 r.f. circuit and impedance matching

The NRSE spectrometer operates at a single frequency for each scan point (or *τ_NRSE_*). Typically, an NRSE scan might consist of 10 or 20 points and therefore 10 or 20 different r.f. frequencies. Thus, even though the drive circuit is “narrow” band for each measurement point, it must be tunable through more than a decade of r.f. frequencies.

**Fig. 28:**
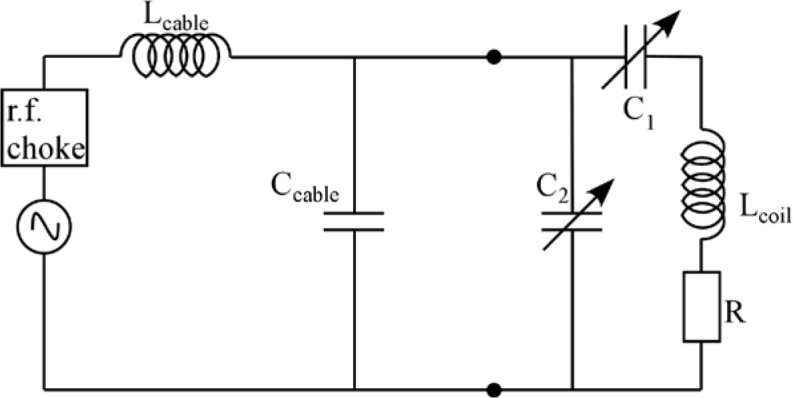
r.f. filter with lossless cables. *R* includes the resistance (likely mainly from the r.f. coil).

In this application at high frequencies, it is important to match the characteristic impedances of the transmission line with that of the load to prevent reflections of r.f. power from the load toward the source. Reactive elements in a circuit (inductance and capacitance) store and return energy to the source unless the circuit appears purely resistive (i.e., the voltage and the current are in phase). This is the condition for impedance matching. Equivalently-stated, the power factor (= cos*θ*), where *θ* is the phase angle between the current and the voltage must ideally equal 1or the net capacitative reactance of the circuit cancels the net inductive reactance. Impedance matching not only maximizes the efficiency of the circuit, but also prevents distortion of the r.f. signal caused by reflected, delayed signals. A lossless coaxial cable may be considered as an inductance in parallel with a capacitance as shown in Fig. 28. By “lossless”, we mean a perfectly insulating coaxial dielectric with negligible wire resistance. In this case, the characteristic impedance, *Z_0_*, of an *impedance-matched* cable at *any* frequency appears purely resistive with magnitude

(247) $$Z_{0}=\sqrt{\frac{L_{cable}}{C_{cable}}}=\sqrt{\frac{L^{\prime }}{C^{\prime }}},$$

where *L*′ and *C*′ are the characteristic inductance and capacitance per unit length of (uniform) cable. Typically, for coaxial r.f. cables, *Z_0_* is 50 Ω by design. The task is then to match the impedance of the rest of the circuit (including the r.f. coil) to emulate a resistive value of magnitude *Z_0._* Consider the r.f. filter circuit shown in Fig. 28. The power supply acts like a current source and the choke protects the source by giving it high output impedance at high frequency. The r.f. coil may be considered as the combination of the inductance and the series resistance, *R*. The parallel tunable capacitance *C_2_* allows maximization of the power factor by canceling the inductive reactance of the r.f. coil (which increases proportional to the frequency). In other words, the tunable capacitance *C_2_* is necessary to maintain the imaginary part of the impedance of the circuit at zero, because the capacitance *C_1_* must also change with frequency to maintain the *real* part of the circuit impedance at the value *Z_0_*; the impedance would otherwise decrease with increasing frequency causing signal reflection towards the source, and most of the voltage drop would occur across the cable. *C_1_* also A.C.-couples the r.f. coil to the power supply so that the undistorted r.f. voltage truly oscillates about 0 V. The reciprocal load impedance of the combined *C_1_*, *C_2_*, *L*, *R* part of the circuit is

(248) $$\frac{1}{Z_{load}}=j\omega C_{2}+\frac{1}{R+j\left(\omega L-\frac{1}{\omega C_{1}}\right)}=j\omega C_{2}+\frac{R-j\left(\omega L-\frac{1}{\omega C_{1}}\right)}{R^{2}+{\left(\omega L-\frac{1}{\omega c_{1}}\right)}^{2}},$$

where we have used *X_C_* = −*j/ωC*, *X_L_* = *jωL*, and the complex identity *y* = y y^*^/y^*^, etc. For exact impedance matching, we require Re(*Z_load_*) = *Z_0_* with a zero voltage-current phase difference which means that Im(*Z_load_*) (and consequently Im(1/*Z_load_*)) is zero. From Eq. (248), therefore, we have

(249) $$\frac{1}{Z_{0}}=\frac{R}{R^{2}+{\left(\omega L-\frac{1}{\omega C_{1}}\right)}^{2}}\;(\text{for exact impedance matching})$$

and

(250) $$\omega C_{2}-\frac{\left(\omega L-\frac{1}{\omega C_{1}}\right)}{R^{2}+{\left(\omega L-\frac{1}{\omega C_{1}}\right)}^{2}}=0\;(\text{for exact impedance matching}).$$

Note that we consider *R*, *L*, and *Z_0_* as fixed (neglecting a possible high frequency dependence of *R*(*ω*) due to the reduction of the conducting cross-sectional area of the wire caused by the skin effect (Sec. 7.3.4.7)). From the impedance matching conditions Eqs. (249) and (250), we obtain:

(251) $$C_{1}(\omega )=\frac{1}{\omega \left(\omega L-\sqrt{R(Z_{0}-R)}\right)}\;(\text{for exact impedance matching})$$

and

(252) $$C_{2}(\omega )=\frac{L-\frac{1}{\omega^{2}C_{1}(\omega )}}{R^{2}+{\left(\omega L-\frac{1}{\omega C_{1}(\omega )}\right)}^{2}}=\frac{1}{\omega Z_{0}}\sqrt{\frac{Z_{0}-R}{R}}\;(\text{for exact impedance matching}).$$

For these particular values of *C_1_* and *C_2_*, we construct a simplified table of voltages, currents, and impedances remembering that the cable acts like a pure resistance of *Z_0_* (as does the *C_1_*, *C_2_*, *L*, *R* part of the circuit), and these two sub-circuits reduce to a 2:1 voltage divider (Table 8).

**Fig. 29:**
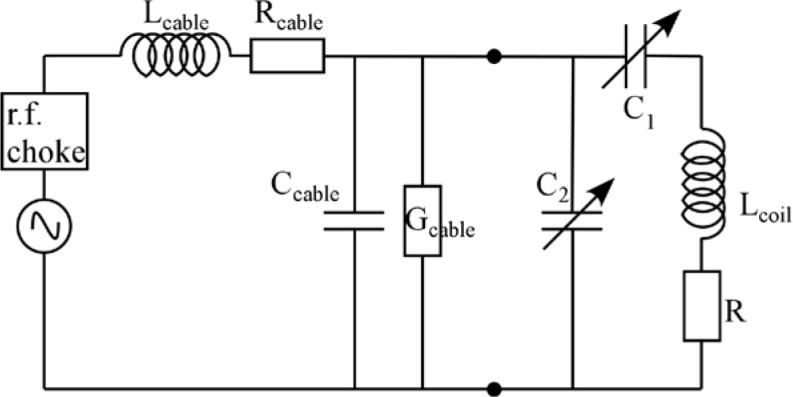
r.f. filter with losses in cables (cable resistance, represented by *R_cable_*, and conductance of dielectric, represented by *G_cable_*).

More realistically, the transmission line insulator has some conductance (represented by *G_cable_*) and nonzero resistance, represented by *R_cable_* (as shown in Fig. 29). Distortionless cables are fabricated such that

(253) $$\frac{G^{\prime }}{C^{\prime }}=\frac{R^{\prime }}{L^{\prime }},$$

where again the prime represents “per unit length”. Note that if such a circuit is used to drive *M* coils in parallel (for example the four coils of one arm of a 4 – *N* = 2 coil NRSE), the current through *C_1_* will be *M* times greater than for the single coil. Therefore, the capacitors must be rated to handle these currents.

##### 7.3.4.3 r.f. coil frequency, currents, and voltages

**Fig. 30:**
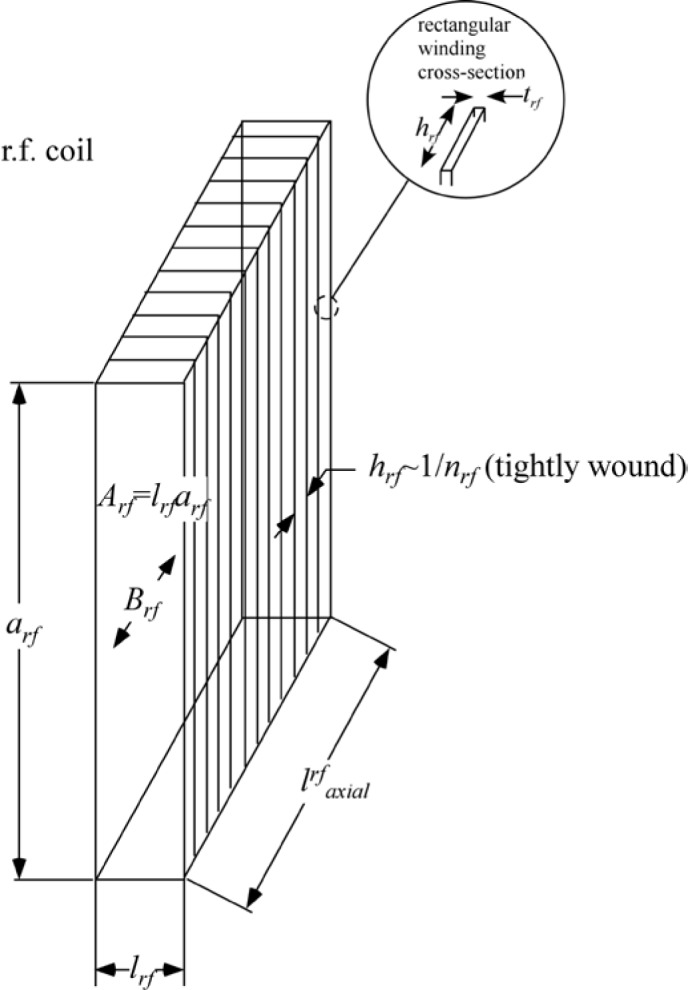
r.f. coil dimensions assuming rectangular cross-section windings.

The r.f. coil dimensions used in this and subsequent sections are shown in Fig. 30. From Table 8 at exact impedance matching we have (substituting the value of *C_2_*(*ω*) from Eq. (252)):

(254) $$V_{L}=\omega L\frac{V_{in}}{2Z_{0}}\left(j-\sqrt{\frac{Z_{0}-R}{R}}\right),$$

where *V_in_* is the *supply* voltage, with

(255) $$\left|V_{L}\right|=\omega L\frac{V_{in}}{2}\sqrt{\frac{1}{Z_{0}R}}$$

and

(256) $$I_{L}=\frac{V_{in}}{2Z_{0}}\left(1+j\sqrt{\frac{Z_{0}-R}{R}}\right)$$

with

(257) $$\left|I_{L}\right|=\frac{V_{in}}{2}\sqrt{\frac{1}{Z_{0}R}},$$

so that

(258) $$I_{L}=-j\frac{V_{L}}{\omega L}.$$

We see that *V_L_* is proportional to the frequency and *L* and that the current lags the voltage by 90°, as expected for a pure inductance. Because the r.f. frequency must match the Larmor precession frequency in the static field coils, reaching the maximum frequency of operation of the r.f. coil imposes the principal technical challenge. According to Eq. (10), we have *ν_rf_* ≈ 1.17 MHz for *B_0_* ≈ 0.04 T (729 KHz for *B_0_* = 0.025 T). Also, according to Eq. (15), the peak r.f. field is optimally-tuned according to the mean incident wavelength with

(259) $$B_{rf}^{pk}[T]≃\frac{1.35645\times 10^{-4}}{l_{rf}[m]\langle \lambda_{i}\rangle [Å]}$$

so that the largest r.f. field magnitude is defined by the *minimum* incident wavelength. Using the long solenoid approximation (Eq. (213)), we conclude that the peak current required in the r.f. coil is approximately

(260) $$\begin{array}{l} I_{rf}^{pk}[A]≃\frac{B_{rf}^{pk}[T]}{\mu \left[{\text{NA}}^{-2}\right]n_{rf}[m^{-1}]}≃\frac{1.356\times 10^{-4}}{\mu [{\text{NA}}^{-2}]n_{rf}[m^{-1}]l_{rf}[m]\langle \lambda_{i}\rangle \left[Å\right]}\\ ≃\frac{108}{n_{rf}[m^{-1}]l_{rf}[m]\langle \lambda_{i}\rangle \left[Å\right]}\;(\text{in air}/\text{vacuum}), \end{array}$$

where *n_rf_* is the winding density of the r.f. coil, so that the root mean square (rms) current is approximately

(261) $$\begin{array}{l} I_{rf}^{rms}[A]=\frac{I_{rf}^{pk}[A]}{\sqrt{2}}≃\frac{9.592\times 10^{-5}}{\mu [{\text{NA}}^{-2}]n_{rf}[m^{-1}]l_{rf}[m]\langle \lambda_{i}\rangle \left[Å\right]}\\ ≃\frac{76.3}{n_{rf}[m^{-1}]l_{rf}[m]\langle \lambda_{i}\rangle \left[Å\right]}\;(\text{in air}/\text{vacuum}). \end{array}$$

Applying Faraday’s law to a coil of inductance *L*,

(262) $$V=-L\frac{dI}{dt}$$

for which Eq. (258) is obviously a solution for sinusoidally-varying currents and voltages. For a sinusoidally-varying current $I_{rf}(t)=I_{rf}^{pk}$ sin *ω_rf_* (*t*) the maximum rate of change of the current is

(263) $${\left(dI/dt\right)}_{\max}[{\text{As}}^{-1}]=I_{rf}^{pk}[A]\omega_{rf}[s^{-1}]\approx 1.832\times 10^{8}I_{rf}^{pk}[A]B_{0}[T]$$

and for a long solenoid, we use the approximation

(264) $$L\approx \mu n_{rf}^{2}l_{axial}^{rf}A_{rf}$$

(*A_rf_* = *a_rf_* × *l_rf_* – see Fig. 30), whence

(265) $$\begin{array}{l} L[H]\approx 4\pi \times 10^{-7}{\left(n_{rf}[m^{-1}]\right)}^{2}l_{axial}^{rf}[m]A_{rf}[m^{2}]\\ \approx 1.26\times 10^{-6}{\left(n_{rf}[m^{-1}]\right)}^{2}l_{axial}^{rf}[m]A_{rf}[m^{2}]. \end{array}$$

Now Eq. (262) can be rewritten using Eqs. (260), (263), and (264), replacing *A_rf_* by *a_rf_* × *l_rf_*, where *a_rf_* is the r.f. coil dimension perpendicular to both the beam direction and to the r.f. field direction:

(266) $$V_{rf}^{pk}[V]\approx 2.5\times 10^{4}\frac{n_{rf}[m^{-1}]l_{axial}^{rf}[m]a_{rf}[m]}{\langle \lambda_{i}\rangle \left[Å\right]}B_{0}[T].$$

From Eq. (266) we see that the maximum voltage occurs at maximum *B_0_* and minimum *λ*. We will assume that the minimum useful wavelength is 2 Å. From Eq. (14) for a typical r.f. coil thickness *l_rf_* (in the beam direction) of 2.5 cm, we have *B_rf_^pk^*(*l_rf_* = 0.025 m, 〈*λ_i_*〉 = 2 Å) ≈ 2.71 mT. The peak current in the r.f. coil with winding density *n_rf_* = 250 m^−1^ is approximately *I_rf_^pk^*(*n_rf_* = 250 m^−1^, *l_rf_* = 0.025 m, 〈*λ_i_*〉 = 2 Å) ≈ 8.64 A. This is approximately one order of magnitude less than the maximum currents required in the static field coils with similar winding densities.

Typical r.f. coils such as those shown in Fig. 27, have self-inductances of order (40 to 50) *µ*H. For *L* = 50 *µ*H, *I_pk_* ≈ 8.64 A, *ω_0_* = 2*πν_0_*(max) ≈ 7.35×10^6^ rad s^−1^, we have *dI/dt* ≈ 6.3×10^7^ As^−1^ with a peak voltage across the r.f. coil of around *V_rf_^pk^*(*n_rf_* = 250 m^−1^, *l_rf_* = 0.025 m, 〈*λ_i_*〉 = 2 Å) ≈ 3.2 kV. This high peak voltage poses various challenges for electrical insulation, switching, and other circuit issues covered in Sec. 7.3.4.2. Typically Teflon-insulated high voltage cables are limited to about 1.5 kV. The peak voltage in this example may impose a limitation on the minimum operational wavelength.

The voltage between adjacent windings in a tightly-wound coil, *V_pk_^ww^*, must also be maintained below breakdown. For a simple voltage divider, this is simply the total voltage difference across the coil multiplied by the fractional length of one turn with respect to the total winding length on the coil. For tightly-wound rectangular windings this fraction is *h_rf_*/*l_axial_* = 1/(*n_rf_ l_axial_*) = 1/*N_rf_*, where *N_rf_* is the total number of turns on the r.f. coil, i.e.,

(267) $$V_{pk}^{ww}=\frac{V_{pk}^{\max}}{n_{rf}l_{axial}}=\frac{V_{pk}^{\max}}{N_{rf}}.$$

##### 7.3.4.4 r.f. power supply voltage at exact impedance matching

If *V_rms_^PS^* is the rms voltage of the *power supply*, we equate the magnitude of the rms current in the coil at exact impedance matching (from Table 8, with *V_in_* replaced by *V_rms_^PS^* and with the required value of *C_2_* from Eq. (252)) with the required rms current magnitude from Eq. (261), whence

(268) $$I_{rf}^{rms}[A]≃\frac{9.592\times 10^{-5}}{\mu [{\text{NA}}^{-2}]n_{rf}[m^{-1}]l_{rf}[m]\langle \lambda_{i}\rangle \left[Å\right]}=\frac{V_{rms}^{PS}}{2\sqrt{Z_{0}R}}\;\text{exact impedance matching}.$$

Therefore, if we assume *Z_0_* is 50 Ω, we require a power supply voltage of

(269) $$V_{rms}^{PS}[V]≃\frac{152.7\sqrt{Z_{0}[\Omega ]R[\Omega ]}}{n_{rf}[m^{-1}]l_{rf}[m]\langle \lambda_{i}\rangle \left[Å\right]}=\frac{1079.4\sqrt{R[\Omega ]}}{n_{rf}[m^{-1}]l_{rf}[m]\langle \lambda_{i}\rangle \left[Å\right]}\;\text{exact impedance matching},Z_{0}=50\Omega .$$

For *R* ≈ 1 Ω, *n_rf_* [m^−1^]*l_rf_* [m] ≈ 10, the supply voltage is approximately 100 V/*λ*[Å]. Note that this is typically much smaller than the high frequency voltages generated across the r.f. coil itself.

##### 7.3.4.5 Power dissipation in the r.f. circuit and in the r.f. coil

At exact impedance matching, Re(*Z_load_*) = *Z_0_*, Im(*Z_load_*) = 0 (see Sec. 7.3.4.2), and the resistance of the entire circuit is 2*Z_0_*, therefore the heat dissipated in the whole circuit is

(270) $$P_{rf\;circuit}=2{\left(I_{rms}^{PS}\right)}^{2}Z_{0}=\frac{{\left(V_{rms}^{PS}\right)}^{2}}{2Z_{0}}\;\text{exact impedance matching},$$

where $I_{rms}^{PS}$ is the total current delivered to the circuit from the power supply. Using Eq. (269) for *V_rms_^PS^* with *Z_0_* = 50 Ω, we have

(271) $$P_{rf\;circuit}[W]=1.165\times 10^{4}R[\Omega ]{\left(\frac{1}{n_{rf}[m^{-1}]l_{rf}[m]\langle \lambda_{i}\rangle \left[Å\right]}\right)}^{2}\;\text{exact impedance matching},Z_{0}=50\Omega .$$

Using the example from the previous section (i.e., *R* ≈ 1 Ω, *n_rf_* [m^−1^]*l_rf_* [m] ≈ 10) we have *P_rf circuit_* ≈ 117 W/(*λ*[Å])^2^ ≈ 30 W for a minimum wavelength of 2 Å.

The power dissipated as heat in the r.f. coil is only due to the coil resistance (the reactance alternately stores and releases energy back towards the source). From Table 8, at exact impedance matching, the voltage across the coil (assuming all the resistance *R* is due to the coil) is

(272) $$V_{coil}≃\frac{V_{rms}^{PS}}{2}\left(\frac{1}{Z_{0}}+j\omega C_{2}\right)R\;\text{at exact impedance matching}.$$

Therefore the power dissipated as heat in the coil at exact impedance matching is

(273) $$P_{coil}={\left|\frac{V_{rms}^{PS}}{2}\left(\frac{1}{Z_{0}}+j\omega C_{2}\right)\right|}^{2}R=\frac{{\left(V_{rms}^{PS}\right)}^{2}}{4Z_{0}}={\left(I_{rf}^{rms}\right)}^{2}R=\frac{P_{rf\;circuit}}{2}\;\text{at exact impedance matching},$$

where we have used the value of *C_2_* from Eq. (252), i.e., one half of the total power dissipated in the circuit (c.f. Eq. (270)) is dissipated in the r.f. coil at exact impedance matching.

##### 7.3.4.6 r.f. coil cooling

Because the maximum currents required in the r.f. coils are not so large (of order 10 A), experience has shown that compressed air cooling is usually adequate to maintain them below 100 °C for frequencies of up to about 750 kHz.

##### 7.3.4.7 The skin effect and the resistance of the r.f. coil windings

An unfortunate consequence of induced eddy currents and Lenz’s law at high frequencies is the concentration of current towards the outer surface of the conductor, commonly referred to as the “skin effect”. The skin depth (or thickness within which the current falls to 1/*e* of its outer surface value for a thick conductor), *δ*, is inversely proportional to the square root of the frequency:

(274) $$\delta =\sqrt{\frac{2}{\mu \sigma \omega }},$$

where *σ* is the conductivity and *µ* is the permeability of the wire. Two undesirables result. Firstly the resistance of the wire increases rapidly when *δ* is comparable to, or less than, the wire diameter. The skin depth for copper at the highest frequencies required (~1 MHz) is about 66 *µ*m (0.065 mm). Secondly, the increase in resistance with increasing frequency induces a frequency-dependence of the signal velocity causing dispersion even in a ‘distortionless’ cable. However, the latter effect is negligible for the NRSE due to the extremely narrow r.f. bandwidth for each spectrometer setting.

One solution for r.f. coils operating at about 1 MHz is to use multiple small diameter (preferably < *δ*), individually-insulated copper wires in parallel rather than fewer thicker conductors. This is the case for the coils shown in Fig. 27. Unfortunately, this is only feasible for the return coils outside the beam passage. For aluminum, the skin depth at 1 MHz is about 83 *µ*m (0.083 mm), i.e., for aluminum we assume that

(275) $$\delta_{Al}[\text{mm}]≃\frac{83}{\sqrt{\nu [\text{Hz}]}}.$$

For band-type windings, where *h* ≫ *t*, the ratio of the D.C. resistance, *R_0_*, to the winding resistance at frequency *ν*, *R*(*ν*), is given approximately by

(276) $$\frac{R(\nu )}{R_{0}}\approx \frac{\int_{0}^{t/2}dx}{\int_{0}^{t/2}\exp\left(-\frac{x}{\delta }\right)dx\;}=\frac{t}{2\delta \left[1-\exp\left(-\frac{t}{2\delta }\right)\right]}\;\text{band-like windings with}\;h\gg t.$$

Note that in the low-frequency limit *δ* ≫ *t*, therefore

(277) $$\frac{R(\nu )}{R_{0}}\approx \frac{t}{2\delta \left[1-\left(1-\frac{t}{2\delta }\right)\right]}=1\;\delta \gg t,\;$$

as expected, and in the high-frequency limit *t* ≫ *δ*, therefore

(278) $$\frac{R(\nu )}{R_{0}}\approx \frac{t}{2\delta }\;t\gg \delta ,$$

i.e., the band winding of thickness *t* in the high frequency limit has approximately the D.C. resistance of a conductor of thickness 2*δ* with the central core volume behaving like a perfect insulator. Applying Eqs. (275) and (276) to the *t* = 0.4 mm-thick aluminum band example from Sec. 7.3.2, we see that the resistance at 1 MHz is about 5 times greater than the D.C. resistance with a current at the center of the conductor of less than 9 % of the value near the surface. In this particular example, the 1 MHz conductor resistance would drop by less than 10 % even if the conductor were made arbitrarily thick. Consequently, increasing the aluminum winding thickness beyond a few 1/10ths of a millimeter (for MHz frequencies) results in only modest power reduction with unnecessary losses of neutron transmission.

##### 7.3.4.8 Allowable *ΔB_rf_*/*B_rf_* and *Δl_rf_*/*l_rf_*

In each *π*-flipper coil, the neutron spin ideally precesses through an angle *π* around *B_rf_* during neutron passage through the coil. The actual angle of precession of the spin about *B_rf_* is determined by the dispersion due to the spread of incident wavelengths, as discussed in Sec. 2.2. However, an additional loss of polarization/flipping efficiency by a similar mechanism results if there is a spread in the magnitude of *B_rf_* or of the length of the r.f. field, *l_rf_*. In order to maximize intensity, we wish to maximize the operational neutron wavelength bandwidth of the spectrometer. Therefore, it is reasonable to require that the effects of *Δ*(*B_rf_ l_rf_*) be small compared with the effects due to dispersion. In this way, acceptable operation of the instrument is sustained for the largest possible *Δλ*/*λ*. *Δ*(*B_rf_ l_rf_*) is reduced by appropriate coil engineering. Because of the similarities with dispersion, we use Eq. (43) (for triangular distributions for both *Δ*(*B_rf_ l_rf_*) and *Δλ*), or Eq. (44) (for Gaussian distributions), with *Λ_FWHM_* (see Eq. (36)) replaced by an effective value

(279) $$\Lambda_{FWHM}^{eff}\approx \sqrt{{\left(\frac{\Delta (B_{rf}l_{rf})}{B_{rf}l_{rf}}\right)}^{2}+{\left(\frac{\Delta \lambda_{FWHM}}{\langle \lambda_{i}\rangle }\right)}^{2}}.$$

We choose a reasonable criterion whereby (*Δ*(*B_rf_ l_rf_*)/*B_rf_ l_rf_*)^2^ is at most 10 % of (*Δλ_FWHM_*/〈*λ_i_*〉)^2^, i.e.,

(280) $$\Lambda_{FWHM}^{eff}\leq 1.05\Lambda_{FWHM}.$$

Estimated flipping efficiencies for triangular distributions (for *M* = 8 flipper coils) assuming (i) only dispersion and (ii) Eq. (280) for the combined effects of dispersion and *Δ*(*B_rf_ l_rf_*) are compared in Table 9. The results demonstrate that the latter creates measurable but tolerably small reductions in the flipping efficiency with respect to the effect of dispersion alone.

If we assume typical spectrometer operation at *ΛFWHM* <~ 10 %, we require

(281) $$\frac{\Delta (B_{rf}l_{rf})}{B_{rf}l_{rf}}<\sim 0.03.$$

Further assuming *equal* fractional error contributions due to *ΔB_rf_* and *Δl_rf_*, we can write

(282) $$\frac{\sqrt{2}\Delta B_{rf}}{B_{rf}}=\frac{\sqrt{2}\Delta l_{rf}}{l_{rf}}<\sim 0.03.$$

Using Eq. (282) for *l_rf_* = 0.025 m, we have *Δl_rf_* <~ 0.6 mm (which is not excessively demanding), with *ΔB_rf_*/*B_rf_* = *ΔB_rf_^pk^/B_rf_^pk^* <~ 2 % (which is more than an order of magnitude more relaxed than the required static field homogeneities (see Table 7)). Using Eq. (259) the above criterion may be re-expressed as

(283) $$\Delta B_{rf}^{pk}[T]≃\frac{2.9\times 10^{-6}}{l_{rf}[m]\langle \lambda_{i}\rangle \left[Å\right]}.$$

For the same *l_rf_* (= 0.025 m) and a wavelength range from 2 Å to 12 Å, (*B_rf_^pk^* varies from about 2.7 mT to about 0.45 mT respectively), we require *ΔB_rf_^pk^* <~ 60 *µ*T at 2 Å and *ΔB_rf_^pk^* <~ 10 *µ*T at 12 Å.

#### 7.3.5 Stray Fields in the “Zero Field” Regions

There are inevitably stray fields within the “zero-field” gaps that give rise to unwanted Larmor precession around the local stray field direction. In the worst cases, these can severely reduce or even destroy the echo signal. Sources of stray fields are leakage fields from the coils themselves, the Earth’s magnetic field, and other externally-produced magnetic fields. Even-*N* bootstrap coils greatly reduce the coil contribution by providing compact, closed return paths due to the oppositely-opposed field directions. Furthermore, the leakage field has opposite sign each side of the bootstrap coil, resulting in a first order cancellation of the Larmor precession upstream and downstream of the coil. Tight conduction of field lines between the coil pairs (and away from the zero field regions) is greatly improved by using high permeability *µ*-metal caps linking the coil ends. Leakage fields into the zero-field flight paths are further reduced by encapsulating the coil in a *µ*-metal screen with the exception of the beam path. External sources of stray field (such as the Earth’s field) are practically eliminated by surrounding the sensitive flight paths with multi-skinned *µ*-metal shielding [24] (see also Sec. 7.7). For a mean net stray field of magnitude *B_stray_*, integrated along the spectrometer arm of length *L*, the net additional precession angle is

(284) $$\langle \Delta \varphi_{stray}\rangle [\text{rad}]\approx \frac{\gamma_{n}m_{n}}{h}B_{stray}L\langle \lambda_{i}\rangle =4.63\times 10^{4}B_{stray}[T]L[m]\langle \lambda_{i}\rangle \left[Å\right].$$

*Example:* Unshielded Earth’s magnetic field (zip code 21737, Oct 8 2008) = 5.25 × 10^−5^ T, horizontal component = 2.06 × 10^−5^ T, vertical component = 4.83 × 10^−5^ T, *L* = 2 m, 〈*λ_i_*〉 = 8 Å, 〈*Δϕ_stray_*〉 = 35.8 rads = 2050.1° = 5.7 turns.

If the dominant stray field component is from coil leakage, the leakage field lines tend to align along the direction of the static fields. If we impose the constraint that the net precession angle in each arm of the spectrometer should not exceed about 10°, we require

(285) $$B_{stray}[T]L[m]\leq \frac{3.8\times 10^{-6}}{\langle \lambda_{i}\rangle \left[Å\right]}.$$

If *λ* = 8Å this corresponds to a stray field integral of about 5 × 10^−7^ Tm (for *L* = 2 m and *B_stray_* of about 0.2 *µ*T integrated over *L*). Gähler, Golub, and Keller [8] show measured stray fields obtained outside a typical *µ*-metal capped *N*=2 bootstrap coil. The stray field magnitude for such a coil with an internal field *B_0_* of 1.67 mT is about 3 *µ*T for about the first 0.05 m falling to less than 0.5 *µ*T at about 0.2 m from the coil. The estimated stray field integral on one side of the coil from this measurement is about 8 × 10^−7^ Tm. At *B_0_* = 0.04 T internal field, we might expect the stray field integral to be about (0.04 × 8 × 10^−7^/1.67 × 10^−3^) ≈ 1.9 × 10^−5^ Tm (38 times greater than the value given for *λ* = 8 Å above). In his Ph.D. thesis, T. Keller [25] shows that the field integral of such coils could be reduced by an additional factor of about 30 by adding the *µ*-metal screen around the *N* = 2 coil. In this case we anticipate a typical stray field integral magnitude on *one* side of the coil unit of about *Δ*_1_ ≈ 6.3 × 10^−7^ Tm at *B_0_* = 0.04 T, with a similar stray field integral magnitude *Δ*_2_ on the other side (of opposite sign). If the 4−*N* = 2 coils are arranged as shown in Fig. 6 and the typical residual net leakage field integral due to each *N* = 2 coil unit (=〈*Δ*_1_ − *Δ*_2_〉) is added in quadrature for the two coil units of each arm, using Eq. (285) we have a stray field integral cancellation criterion given by

(286) $$\langle \Delta_{1}-\Delta_{2}\rangle [\text{Tm}]<\sim \frac{3.8\times 10^{-6}}{\sqrt{2}\langle \lambda_{i}\rangle \left[Å\right]}\approx \frac{2.7\times 10^{-6}}{\langle \lambda_{i}\rangle \left[Å\right]}.$$

For the *λ* = 8Å example, this requires 〈*Δ*_1_ − *Δ*_2_〉 <~ 3.4×10^−7^ Tm for 10° net stray field precession in each arm. For *Δ*_1_ ≈ 6.3 × 10^−7^ Tm, this means that the typical stray field cancellation need only be about 50 % in this case. This appears entirely achievable.

#### 7.3.6 Measurement of Small τ_NRSE_

##### 7.3.6.1 The Bloch-Siegert shift

In the previous discussion, the resonant component of the r.f. field has been approximated as a pure rotating field and the influence of the counter-rotating component has been ignored. However, the applied r.f. field is an *oscillating* field, not a pure rotating field. Bloch and Siegert [5] treated the case that really exists in the resonance coil for a spin-1/2 particle traversing a static field with a superimposed, perpendicular oscillating field. This problem does not have an exact solution. However, they showed that for increasing *B_0_*/*B_rf_*, the solution increasingly approximates to that of a “static + circular” field with a similarly-shaped resonance curve, but with a resonance frequency that *deviates* from the classical Larmor frequency, *ω_0_*, by a fractional amount equal to

(287) $$\frac{\Delta \omega_{BS}}{\omega_{0}}=\frac{\left|\omega_{rf}-\omega_{0}\right|}{\omega_{0}}=\frac{B_{rf}^{2}}{16B_{0}^{2}}.$$

Typically for high-resolution operation of the NRSE flipper coils this fraction is small. For example, according to Eq. (15), for *l_B0_* = 0.03 m and short wavelength operation (*λ* = 2 Å), *B_rf_* = 2.26 mT. For *B_0_* = 0.04 T, *Δω_BS_*/*ω_0_* ≈ 2×10^−4^ corresponding to about 233 Hz. However, at low *τ_NRSE_*, *Δω_BS_* can be a significant fraction of the Larmor frequency.

##### 7.3.6.2 Solution using NSE mode operation of coils

When *B_0_* becomes comparable to *B_rf_*, the flippers do not perform well. Köppe *et al.* [11] provide some idea of when this is likely to occur. Their coils cease to operate satisfactorily for static fields *B_0_* < 2.7 mT for *λ* ≤ 6 Å. Assuming that these r.f. coils are no greater than about 0.025 m thick in the beam direction, we infer from Eq. (15) that the peak r.f. field at which problems occur is for *B_rf_^pk^* >~ 0.9 mT or *B_rf_* >~ 0.45 mT. Thus we assume that the *π*-flippers must be operated under the following condition:

(288) $$\frac{B_{rf}}{B_{0}}\leq 0.17\;\text{approximate codition for operating in NRSE mode}.$$

**Fig. 31:**
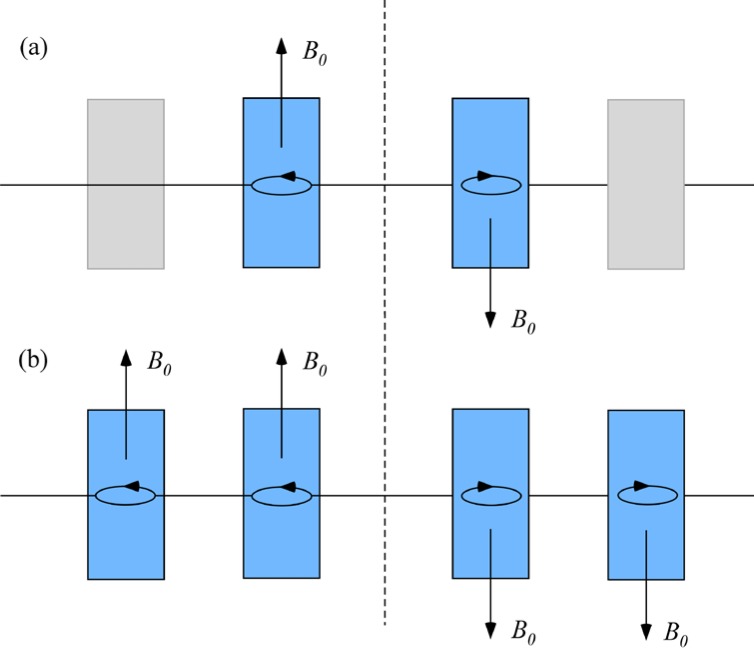
Using a 4-*N* = 1 coil NRSE spectrometer in NSE mode for measuring small Fourier times. The r.f. field is turned off in the coils. (a) Using the static field of two coils for the precession fields; (b) using the static field of all four coils to access higher *τ*.

This condition clearly limits the dynamic range of the NRSE instrument with respect to low *τ* (low *B_0_*) measurements. One possibility for measuring short Fourier times, proposed by Gähler, consists of turning off the r.f. field to all the coils and running in classic NSE mode. In a 4-*N* = 1 coil configuration one or both of the static coils in each arm can be operated. The fields are oriented in the correct sense in this case so that no *π*-flipper is required between the two arms of the spectrometer as in a conventional (longitudinal field) NSE instrument; consequently Larmor precession occurs within the coil length in opposite directions on each side of the sample. This is illustrated in Fig. 31. Several points at low *τ* can be measured by adjusting the field magnitude in the active coils.

**Fig. 32:**
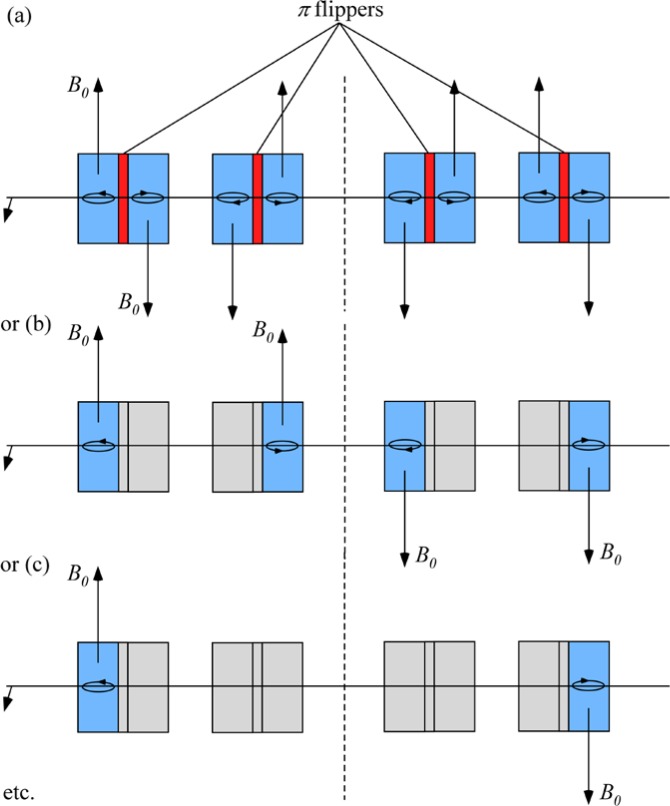
Using a 4-*N* = 2 coil NRSE spectrometer in NSE mode for measuring small Fourier times. The r.f. field is turned off in all coils: (a) all static fields on, using *π* flippers (b) (if feasible) switching off all wrong field directions (no *π* flipper) (c) switching off all but two of the fields.

The situation is more complicated in a bootstrap coil configuration (for example *N* = 2). In this case, all r.f. fields are switched off (as in the *N* = 1 case), however the static field directions are inappropriate for operation in NSE mode. Several solutions to this problem are illustrated in Fig. 32. In case (a), all static fields remain on but a *π* flipper is placed between the two opposing coils of a bootstrap pair, essentially reversing the field direction of the second coil. Conceivably, the field direction in the second coil of the pair could be reversed by reversing the current direction in the coil, but the two coils are often constructed from a single winding rendering this option impractical. In case (b), if the coils can be switched out of the circuit independently, only the fields that have the correct direction are energized. (c) is like (b) but only one coil of the correct field direction is energized in each arm. Note that if the static fields are provided by *permanent* magnets (see Sec. 9.2), only option (a) is feasible unless magnets of the wrong field direction are physically removed from the beam. For permanent magnets the NSE scan must be performed by rotating the magnets to change the field integrals in a way that does not introduce unwanted *Q*-dependence (see Sec. 9.3).

##### 7.3.6.3 An example of a combined NSE-NRSE mode scan

For NRSE operation we have *τ_NRSE_* given by Eq. (120), i.e., *τ_NRSE_*[ns] = 0.37271 *N B_0_*[T] *L_0_*[m] (*λ_i_*[Å])^3^, where (combining Eqs. (288) and (14)) for satisfactory NRSE mode operation we have

(289) $$B_{0}[T]l_{B_{0}}[m]\lambda_{i}\left[Å\right]>\sim 4\times 10^{-4}\text{Tm}Å\;\text{for satisfactory NRSE mode operation},$$

whence

(290) $$\tau_{NRSE}[\text{ns}]>\sim 1.5\times 10^{-4}\frac{NL_{0}[m]{\left(\lambda_{i}\left[Å\right]\right)}^{2}}{l_{B_{0}}[m]}\;\text{for satisfactory NRSE mode operation}.$$

**Fig. 33:**
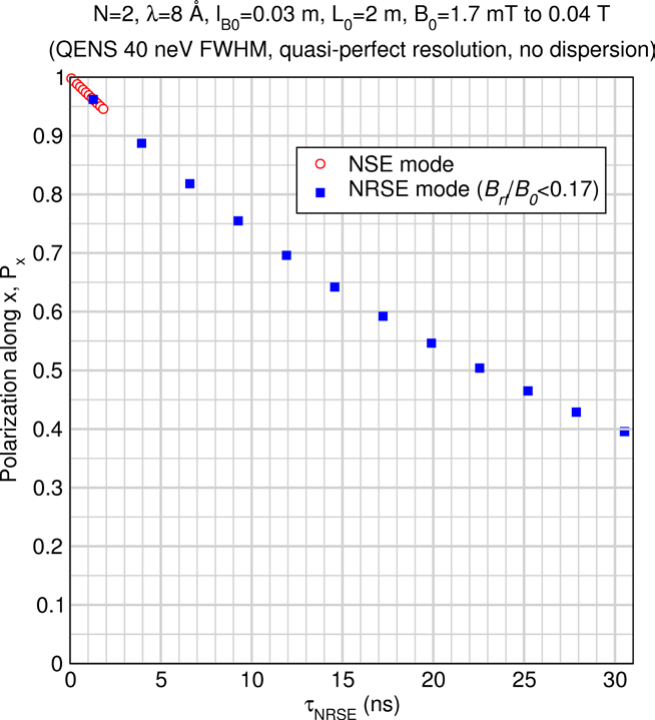
Fourier time (*τ_NRSE_*) ranges resulting from evenly-spaced values of *B_0_* in the range from about 1.7 mT (the minimum for NRSE operation in this example) to 0.04 T for both NSE mode (red circles) and NRSE mode (blue squares). This example is for *λ_i_* = 8 Å, *N* = 2, *L_0_* = 2 m, and *l_B0_* = 0.03 m. The curve happens to correspond to a 40 neV quasi-elastic scatterer in a highly-idealized spectrometer with quasi-perfect resolution (perfectly ideal construction, field uniformity etc.) and zero coil dispersion. Identical values of *B_0_* were used for each mode. Note that the NSE mode is highly compressed with respect to *τ_NRSE_* compared with the NRSE mode.

If we choose *N* = 2, *L_0_* = 2 m, *λ_i_* = 8 Å, and *l_B0_* ≈ 0.03 m for this scan we anticipate that it is possible to operate in NRSE mode for *τ_NRSE_* >~ 1.3 ns. This corresponds to *B_0_* >~ 1.7 mT in this case. For smaller values of *τ*, the spectrometer must be run in NSE mode. In NSE mode we have

(291) $$\tau_{NSE}[\text{ns}]=0.373MB_{0}[T]l_{B_{0}}[m]{\left(\lambda_{i}\left[Å\right]\right)}^{3},$$

where *M* is the total number of static field coils energized. If the maximum *B_0_* is 0.04 T, with *M* = 8 (i.e., the configuration shown in Fig. 32(a)), we can use NSE mode up to *τ_NSE_* ~ 1.8 ns for *λ* = 8 Å, i.e., there *is* a possible overlap between the upper NSE mode and the lower NRSE mode if the scheme in Fig. 32(a) is adopted. Some dummy data points are plotted in Fig. 33 showing the scan points that result from evenly-spaced values of *B_0_* in the range from about 1.7 mT (the minimum field for NRSE operation in this example) to 0.04 T for both NSE mode (red circles) and NRSE mode (blue squares).

### 7.4 Defining the Major Instrument Parameters for the NRSE Instrument Using Coils

We now explore some of the major constraints on the instrument parameters imposed by the proposed instrumental performance goals, when combined with some technical constraints for an NRSE instrument using resonance coils. This is by no means an exhaustive list and additional compromises may be necessary. Probably the major factors are as follows:

1. We wish to access *τ_NRSE_* = 30 ns at *λ* = 8Å. This has implications for the minimum achievable magnitude of the product *B_0_L_0_* expressed in Eq. (206).
2. Once 30 ns at *λ* = 8 Å is accessible, we wish to achieve a resolution function signal (polarization) greater than or equal to a stated minimum value, *P_x_^0^*. The static field homogeneity, coil flatness, and beam divergence required to achieve these conditions are given approximately by Eqs. (203–205) respectively.
3. The anticipated neutron transmission of the windings should be >~ 80 % at *λ* = 8 Å. This concerns the thickness of the windings in the beam direction, *t*. The transmission for aluminum windings may be estimated using the macroscopic cross-section given in Eq. (211).
4. Maximum limitations on the static field coil current/minimum coil winding density (see Eq. (214)).
5. Capacity to remove heat from the static field coils (see for example Eq. (235) for tightly-wound rectangular cross-section aluminum windings), given the estimated constraints on the coil surface area, the winding thickness, *t*, the maximum operating static field, *B_0_^max^*, (as constrained by condition 1 above), and the means available for cooling outside of the neutron beam passage (see Sec. 7.3.3.5).
6. Maximum limitations on the voltage across the r.f. coil at *B_0_^max^* (see Eq. (266)). These are dictated by cabling and insulation breakdown issues with a practical maximum of about 1.5 kV.

We assume, for the reasons given in Sec. 3.4, that the number of coils in the bootstrap is universally *N* = 2 and that the windings are made of aluminum.

Condition 1 for *N* = 2 imposes the constraint already given by Eq. (207), i.e.,

(292) $${\left(B_{0}L_{0}\right)}_{\max}\geq 0.079\text{Tm}\;\text{Condition}\;1:\text{Criterion for accessing}\;\tau_{NRSE}=30\;\text{ns at}\;\lambda =8\;Å\;\text{with}\;N=2.$$

Condition 2 imposes resolution requirements for *N* = 2, which may be stated as follows (assuming equal contributions from each term – see Eqs. (203–205)):

- $\Delta B_{0}^{FWHM}[T]\leq \frac{1.47\times 10^{-5}\sqrt{\ln\left(\frac{1}{P_{x}^{0}}\right)}}{l_{B_{0}}[m]\lambda_{i}\left[Å\right]}$
- $\Delta l_{B_{0}}^{FWHM}[m]\leq \frac{1.47\times 10^{-5}\sqrt{\ln\left(\frac{1}{P_{x}^{0}}\right)}}{B_{0}[T]\lambda_{i}\left[Å\right]}$
- $\Delta \theta_{\max}[\text{rad}]\leq 3.47\times 10^{-3}\sqrt{\frac{\sqrt{\ln\left(\frac{1}{P_{x}^{0}}\right)}}{B_{0}[T]L_{0}[m]\lambda_{i}\left[Å\right]},}\left|\Delta \theta_{\max}\right|[\text{rad}]<\sim \frac{4.7\times 10^{-3}}{\sqrt{B_{0}[T]L_{0}[m]\lambda_{i}\left[Å\right]}}.$

We also assume that these conditions must be satisfied at least for *λ* = 8 Å, thereby being automatically satisfied for *λ* < 8 Å, but not for *λ* > 8 Å. Choosing *P_x_^0^* at (30 ns, 8 Å) ≥ 0.5, the resolution conditions 2(a), (b), and (c) simplify to the approximate relations below, where in (c) we use the *equality* in condition 1 as representing the *minimum* product *B_0_L_0_* required to achieve 30 ns at 8 Å (i.e., the reference point at which condition 2 must apply):

(293) $$\begin{array}{l} (a)\;\Delta B_{0}^{FWHM}[T]\leq \frac{1.5\times 10^{-6}}{l_{B_{0}}[m]}\\ (b)\;\Delta l_{B_{0}}^{FWHM}[m]\leq \frac{1.5\times 10^{-6}}{B_{0}[T]}\;\text{Condition}\;2:\text{To achieve}\;P_{x}{}^{0}\geq 0.5\;\text{at}(30\;\text{ns},8\;Å).\\ (c)\;\Delta \theta_{\max}[\text{rad}]\leq \frac{1.12\times 10^{-3}}{\sqrt{B_{0}[T]L_{0}}[m]}≃4\times 10^{-3} \end{array}$$

Condition 3 amounts to having a total aluminum winding thickness traversed by the neutron beam of less than 24 mm. Using the 2:1 winding thickness ratio for the static: r.f. field coil windings used in the example in Sec. 7.3.2, we treat this condition as only influencing one of the critical parameters in the above list, namely the thickness *t* of the static field coil windings. The condition for the present purposes is therefore stated as *t* ≤ 1 mm on the understanding that the r.f. coil can work satisfactorily with winding thickness≤ 0.5 mm.

Condition 4 can be stated as

$$8\times 10^{5}\frac{B_{0}[T]}{n[m^{-1}]}\leq I_{\max},$$

where *I_max_* is a stated upper limit. If we choose a static field coil upper current limitation of *I_max_* = 100 A, condition 4 can be restated as:

(294) $$\frac{B_{0}[T]}{n[m^{-1}]}\leq 1.25\times 10^{-4}\;\text{Condition}\;4:\text{To limit}\;I_{\max}<100\;\text{A in the static field coil windings}.$$

Condition 5 for aluminum windings amounts to determining the maximum operational power dissipated as heat in the static field coil (i.e., at the maximum operating static field *B_0_^max^*) – see Eq. (235), *P*(*B_0_^max^*), and assessing whether it is reasonable:

$$P_{Al}(B_{0}^{\max})[W]={\left(B_{0}^{\max}[T]\right)}^{2}\frac{A_{surf}[m^{2}]}{t[m]}(72.2T(K)-4.37\times 10^{3}).$$

We will assume an equilibrium winding temperature of *T* ≈ 400 K. Minimizing *P*(*B_0_^max^*) in condition 5 is aided by choosing the *maximum* allowable value of *t* (i.e., 1 mm from condition 3), so that condition 5 becomes:

$$P_{Al}(B_{0}^{\max})[W]=2.45\times 10^{7}{\left(B_{0}^{\max}[T]\right)}^{2}A_{surf}[m^{2}],T=400K,t=t_{\max}=10^{-3}m.$$

We substitute the value of *A_surf_* for a rectangular form coil (Eq. (218)) so we have

$$P_{Al}(B_{0}^{\max})[W]=4.9\times 10^{7}{\left(B_{0}^{\max}[T]\right)}^{2}l_{axial}[m](a[m]+l_{B_{0}}[m]),T=400K,t=t_{\max}=10^{-3}m.$$

We assume from previously-developed coils that *l_axial_* and *a* must be at least about 7 times larger than the beam dimensions to achieve sufficient static field homogeneity within the beam passage. For a 3 cm × 3 cm beam, this equates to *a* ≈ *l_axial_* ≈ 0.2 m. Also *l_B0_* ≈ 0.03 m, therefore, substituting these values for a typical situation we have:

(295) $$\begin{array}{l} P_{Al}(B_{0}^{\max}){\left[W\right]}_{typ}\approx 2.25\times 10^{6}{\left(B_{0}^{\max}[T]\right)}^{2}\;\text{Condition}\;5:\text{Max power in typical static coil with}\;T=400\;K,\\ t=t_{\max}=10^{-3}m(1\;\text{mm}),\;a\approx l_{axial}=0.2m,\;l_{B0}=0.03m. \end{array}$$

Finally, condition 6 can be stated as (see Eq. (266)):

$$2.5\times 10^{4}\frac{n_{rf}[m^{-1}]l_{axial}^{rf}[m]a_{rf}[m]}{\langle \lambda_{i}\rangle \left[Å\right]}B_{0}^{\max}[T]\leq V_{rf}^{\max},$$

where for *V_rf_^max^* = 1500 V, we have:

$$\frac{n_{rf}[m^{-1}]l_{axial}^{rf}[m]a_{rf}[m]}{\langle \lambda_{i}\rangle \left[Å\right]}B_{0}^{\max}[T]\leq 0.06\;\text{for}\;V_{rf}^{\max}\leq 1500V.$$

This condition must be true for *all* operating conditions and consequently also for the minimum operating wavelength, which we choose as *λ* = 2 Å, where the voltage is maximized. Because the r.f. field homogeneity requirements are typically an order of magnitude more relaxed (see Sec. 7.3.4.8) than the required static field homogeneities (see Table 7) at high resolution, we assume that *l^rf^_axial_* and *a_rf_* need be only three times the beam size (i.e., we will make *l^rf^_axial_* = *a_rf_* = 0.09 m). Condition 6 then becomes:

(296) $$n_{rf}[m^{-1}]B_{0}^{\max}[T]\leq 15\;\text{Condition}\;6:\text{For}\;V_{rf}^{\max}\leq 1500\;\text{V for}\;l_{axial}^{rf}=a_{rf}=0.09\;\text{m and}\lambda =\lambda_{min}=2Å.$$

We make one further simplification to express condition 1 in terms of the maximum *B_0_* (*B_0_^max^*) only. The technical conditions 2(b), 4, 5, and 6 are all worst-case at maximum field (*B_0_^max^*). We might reduce the maximum necessary field *B_0_* by reasonably maximizing the inter-coil separation *L_0_* in condition 1. A value of *L_0_* = 2 m is about the longest practical value in terms of available floor space for the instrument. However the disadvantage of further increasing *L_0_* is that the instrumental solid angle of acceptance reduces proportional to 1/*L_0_^2^*. Fixing *L_0_* = 2 m, therefore, condition 1 (Eq. (292)) becomes:

$$B_{0}^{\max}=0.0395T\;\text{Condition}\;1\;\text{for}\;L_{0}=2m.$$

Therefore conditions 2(b), 4, 5, and 6 become respectively:

$$\Delta l_{B_{0}}^{FWHM}[m]\leq 3.8\times 10^{-5}\;(i.e.,\;\text{about}\;38\;\mu \text{m or less}-\text{see Eq}.(293)(b)).$$

$$n[m^{-1}]\geq 316\;(\text{minimum static field coil winding density for}\;I<100\;A-\text{see Eq}.(294)).$$

The minimum achievable power dissipated as heat at the maximum operating field, *B_0_^max^*, is (from Eq. (295)):

(297) $$P_{Al}\left(B_{0}^{\max}\right){\left[W\right]}_{typ}\geq 3510W\;T=400\;K,t=10^{-3}\;m,l_{axial}=0.2\;m,\;l_{B0}=0.03\;m,$$

i.e., the minimum required cooling power to maintain the windings at 400 K.

From Eq. (296) we have:

$$n_{rf}[m^{-1}]\leq 380\;(\text{limiting r.f. coil inductance}).$$

The parameter values 2(c), and 3 have already been determined in this example as *Δθ_max_* ≤ 4 × 10^−3^ rad and *t* = 10^−3^ m respectively.

The remaining parameter range to be determined is 2(a). This is somewhat driven by what is achievable in the coil design, but we have seen (Sec. 7.3.3.7) that small values of *l_B0_* aid in achieving the required static field homogeneity. Given that the static field coil must enclose both the r.f. coil and the necessary structures for heat removal, we anticipate *l_B0_* ≈ 0.03 m as imposing an approximate practical lower limit on the static field coil length (as has been assumed in many of the examples given above). Using this value, condition 2(a) amounts to designing a coil that can achieve

$$\Delta B_{0}^{FWHM}[T]\leq 5\times 10^{-5}.$$

For *B_0_^max^* = 0.0395 T, this corresponds to a static field homogeneity to about 0.305 % or better.

### 7.5 Coupling Coils

**Fig. 34:**
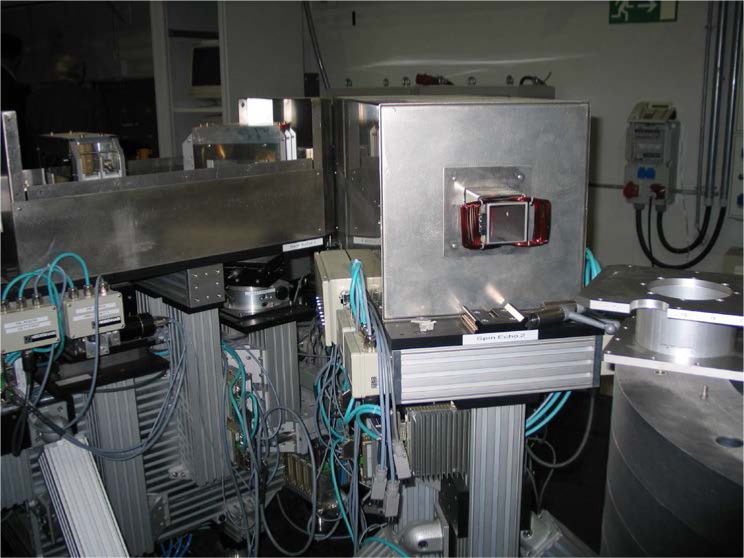
A coupling coil at the exit of a *µ*-metal housing on the NRSE-TAS spectrometer at the FRM-II, Garching, Germany (photo kindly allowed by T. Keller). The windings are bent outwards at the exit to avoid contact with the beam and to ensure an adiabatic transfer from the polarizer/analyzer field to the CC guide field.

The author is grateful to Roland Gähler of the ILL, Grenoble, for providing information about these coils:

The *µ*-metal shield surrounding the coils cannot be closed because a polarized neutron beam cannot be passed through *µ*-metal without significant depolarization. Thus the *µ*-metal tube must be open-ended. The open-ended tube by itself has field lines penetrating partially into the openings, thus in order to maintain control of the polarization direction at the entrance and exit of the *µ*-metal shield, coupling coils (CCs) are used. Gähler *et al.* use a *µ*-metal tube of about 0.1 m diameter into which is introduced a (0.15 to 0.2) m long (in the beam direction), rectangular cross-section CC. An example of a CC penetration into a *µ*-metal shield on the NRSE-TAS spectrometer at the FRM-II is shown in Fig. 34. The magnetic field axis of the CC is perpendicular to the beam. The residual field of the polarizer (and analyzer) at the entrance (exit) of these coils is usually a few hundred *µ*T. The field magnitude in the CCs is also typically a few hundred *µ*T. The windings on the polarizer side are bent outwards (this is visible in Fig. 34) in order to ensure an adiabatic transition from the polarizer field to the CC guide field, whilst eliminating CC windings from the neutron beam path. If the adiabatic condition is met, the neutron spins follow the direction of the CC guide field. On the inner side of the CCs, the neutrons pass abruptly through the windings and a non-adiabatic transition results, whereby the polarization direction immediately prior to passing through the windings is preserved. In order to ensure this, the CC return fields are conducted sharply into an additional *µ*-metal shield that surrounds the inner ends of the coil, thus avoiding a gradual stray field gradient downstream that could affect the polarization direction. Finally, the CCs (and hence the polarization direction) can be rotated through 90° without loss of polarization. For the ILL “Zeta” instrument, both the polarizer field and the initial polarization direction are vertical (parallel to *z*). The CCs are used to rotate the polarization to lie along *x* for normal instrument operation, or along *z* for individual tests of the flipper coils.

#### 7.5.1 Conditions for Adiabatic and Non-Adiabatic Field Transitions

“Adiabatic” and “non-adiabatic” spin transitions in spatially and/or temporally-varying magnetic fields refer to two extremes:

- Adiabatic: the spin direction follows the field direction at all times.
- Non-adiabatic: the neutron passage is sufficiently fast that the spin cannot follow the change of field direction and preserves its original direction.

For the CC it is convenient to consider a magnetic field, of constant magnitude *B_guide_*, initially parallel to the spin direction, which rotates uniformly through an angle *ψ* over a flight path length *d*. In its rest frame, a neutron of constant velocity *v_n_* sees a magnetic field rotating at frequency *Ω*, where

(298) $$\Omega =\psi \frac{v_{n}}{d}.$$

**Fig. 35:**
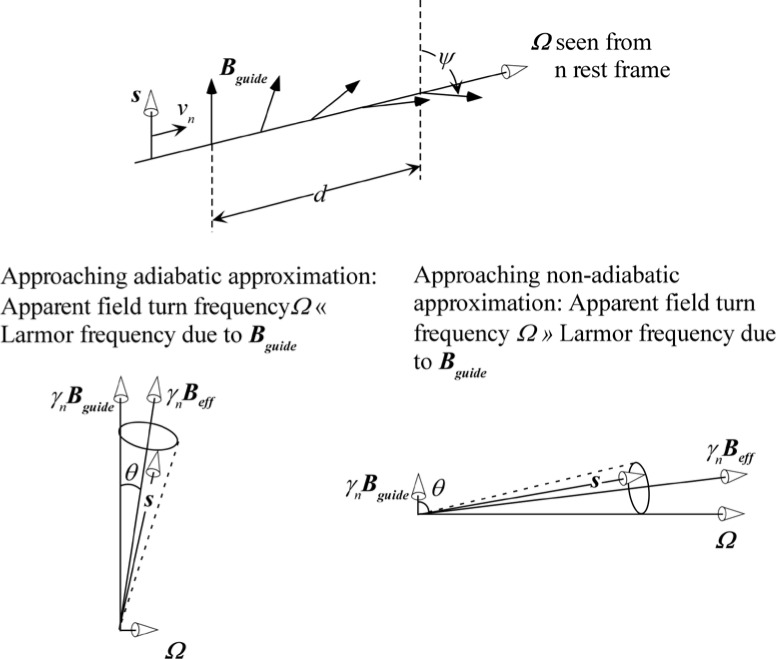
The effective magnetic field in a coordinate frame fixed to the rotating field. Two situations are shown tending towards the extreme adiabatic and non-adiabatic cases.

This situation has been represented by Ramsey [26] and other authors in terms of an effective field in a coordinate frame fixed to the rotating field (see Fig. 35). In the *adiabatic* case, ***B****_eff_* ≈ ***B****_guide_* (i.e., *θ* → 0), therefore ***s*** remains approximately parallel to ***B****_guide_* in the rotating frame and consequently the spin follows the change of direction of the guide field in the lab frame. In the *non-adiabatic* case, ***B_eff_* ≈ *Ω***/*γ_n_* (approximately independent of *B_guide_*, *θ* → *π*/2), and the spins precess at a rate *γ****B_ef__f_* ≈ *Ω***. In the lab frame, therefore, where *B_guide_* rotates with *Ω*, the spins stand still, i.e., they do not follow the change of direction of *B_guide_*. We note that the angle *θ* is given by

(299) $$\theta =\tan^{-1}\left(\frac{\Omega }{\gamma_{n}B_{guide}}\right)=\tan^{-1}\left(\frac{\psi v_{n}}{\gamma_{n}B_{guide}d}\right).$$

An order of magnitude for the required guide fields is obtained by considering several examples of an increasing approach to pure adiabatic rotation of spins (decreasing *θ*) through an angle *ψ* = *π*/2. The angle *ψ* is brought about by a uniform rotation of a guide field (of constant magnitude |*B_guide_*|) over a flight path of 0.5 m (a typical spacing between the polarizer and the coupling coil). The results are shown in Table 10.

### 7.6 Alignment of the *B_0_* Fields Using Coupling Coils

The coupling coils (Sec. 7.5) provide a convenient means of aligning the static fields of the coils in the spectrometer. This is performed by rotating the field axis of the coupling coil such that the neutron spins are aligned along the required *B_0_* field axis. The *B_0_* field of each coil is switched on one at a time and the static field coil is adjusted until the maximum signal is measured in a detector placed downstream of the analyzer.

### 7.7 Magnetic Shielding

It is essential to reduce net stray field integrals in the “zero-field” flight paths to the order of a few ×10^−7^ Tm (see Sec. 7.3.5). At high static fields *B_0_*, this involves magnetic screening of the individual coils units outside of the beam area. Significant sources of external magnetic fields must also be excluded. For example, the action of the unshielded Earth’s magnetic field may give rise to a precession of several turns over a typical 2 m drift path, which additionally is variable depending on the orientation of the spectrometer arm. Uncompensated neighboring magnetic environments may cause worse complications, especially if the field magnitude changes. Therefore, the neutron drift paths between the coils must also be magnetically shielded.

One of the best magnetic shielding materials is so-called “*μ*-metal”. *μ*-metal is an alloy with typical composition 75 % Ni, 2 % Cr, 5 % Cu, 18 % Fe and density of about 8.75 gcm^−3^. It has the property of being very soft magnetically, having a very small coercive field, and an extremely high permeability at low field strengths. With a single-skinned, 1 mm thick *μ*-metal tube, Dubbers *et al.* [22] were able to obtain a shielding factor for the Earth’s magnetic field of about 20, from about 40*μ*T to about 2*μ*T. However, the resulting several *μ*Tm field integral over 2 m drift paths is insufficient by nearly an order of magnitude for achieving the goals discussed in Sec. 7.3.5. The magnetic shielding factor is significantly improved by using multiple-skinned shields with intervening air gaps [27]. The case of triple-skinned, concentric cylindrical and spherical shields was first treated in an elementary way by Wills [24]. Dubbers [28] further simplified the cylindrical geometry, multi-skinned *μ*-metal case in the thin-shell approximation that agrees with the rigorous calculations to about 1 percent in most cases. He reiterates that the shielding is most effective when the shell diameters, *D_i_*, grow in geometric progression, i.e.,

(300) $$\frac{D_{i+1}}{D_{i}}=\kappa ,$$

where *κ* is a constant. Using this approximation, the total shielding factor, *S*, for *n* concentric shells with a constant diameter ratio *κ* is given approximately by

(301) $$S\approx {\left(\frac{\mu_{1}t_{1}}{D_{1}\kappa^{\frac{n+1}{2}}}\left(\kappa^{2}-1\right)+\frac{\left(n\kappa^{2}+n-2\right)}{n\kappa^{2}}\right)}^{n}\frac{\kappa^{2}}{\kappa^{2}-1}\;\text{valid for}\;\kappa <\frac{n+1}{n},$$

where *μ_1_ t_1_*/*D_1_* is the shielding factor of the innermost shell of diameter *D_1_*, thickness *t_1_*, and permeability *μ_1_*. Equation (301) demonstrates the value of using high permeability with *n* > 1, given that *t_1_* cannot be large for practical purposes and *D_1_* cannot be smaller than is allowed by the enclosed instrumentation. However, minimizing *D* not only increases shielding performance but also reduces the cost and weight of the shield. Magnetic shields should also be closed wherever possible since magnetic field lines can penetrate into openings by up to about five times the opening diameter. Closure maintains the reluctance path continuity, increasing shielding performance. Shield closures should also be rounded where possible because flux lines negotiate gentle radii better than sharp angles. One disadvantage with the high permeability of *μ*-metal is its low saturation field (the saturation field is inversely proportional to the permeability). If necessary, the magnetic shielding layer closest to the high field is fabricated from a lower permeability material to avoid saturation and successive shielding layers may be fabricated from increasingly high permeability material, as the field magnitude at each layer reduces.

After fabrication *μ*-metal shielding structures must be annealed in a dry hydrogen atmosphere at about 1200 °C for several hours. The hydrogen atmosphere helps remove carbon and other trace impurities. The high temperature relieves stresses from fabrication and allows the nickel crystallite grain boundaries to expand. The annealing can increase the permeability of the alloy significantly – typically by a factor of 40. However, careful handling of the *μ*-metal after annealing is required. Mechanical shocks readily disrupt the nickel grain structure, negating the permeability gain.

### 7.8 Beam Optics for High-Resolution Operation

In order to achieve the highest resolution goals of the instrument, the neutron flight path length distribution must be narrowed by corrective optics. Some evidence for this is presented in Sec. 8.5. A detailed study of the beam optics will be presented in a separately.

## 8. Monte Carlo Simulations of NRSE Instrument Performance

Some Monte Carlo simulations are presented that illustrate and validate some of the analytical models of the NRSE developed in the previous sections. Numerical techniques are invaluable for modeling complex cases where coupled variables are involved, whilst the analytical models are useful for making rapid predictions of the instrument parameters and performance.

### 8.1 General Description of the Monte Carlo Simulation Method

The time-dependence of *τ_NRSE_* is implicit in the simulations. The neutrons are treated as discrete particles, each having a particular spin vector and all spin coordinate transformations are performed exactly within the limitations of the following assumptions:

1. The r.f. field is rotating in a plane perpendicular to the static field *B_0_*.
2. The interaction of the r.f. field component that is rotating counter to the direction of Larmor precession in the static field can be ignored.
3. f. frequencies of successive coils are phase locked.
4. The magnitude of *B_0_* is assumed large with respect to *B_rf_*.

The neutron beam is assumed to be directed along the *y*-axis of a right-handed coordinate system with the neutron spin initially polarized along the *x*-axis. The applied static fields of the coils are applied parallel or antiparallel to the *z*-axis and the applied r.f. field is in the *x*-*y* plane. The simulation is built around a single r.f. flipper coil module which transforms the entry momentum-spin state of the neutron into an exit momentum-spin state with a main module which handles the spectrometer geometry, source distribution, sample setup, and gathers statistics. The signs of static field directions (and hence the sense of the resonant field rotations) are handled by explicitly applying sgn(*B_0_*) to the frequencies and angles in each coil as described below. For successive coils of a 4-*N* coil NRSE we choose

$$\mathrm{sgn}\;(B_{0})=\{\begin{array}{cc} \left[\begin{array}{cccc} 1 & 1 & -1 & -1 \end{array}\right] & N=1\\ \begin{array}{cccccccc} 1 & -1 & -1 & 1 & -1 & 1 & 1 & -1 \end{array} & N=2 \end{array}.$$

The program allows asymmetric scans to be performed in which the coil separations in the incident arm *L_AB_* are varied with respect to the second arm *L_CD_* in addition to variation of the static field, *B_0_*, for a specified range of discrete values. The coil separation ranges are specified symmetrically with respect to (*L_AB_* – *L_CD_*) = 0 in terms of the number of minimum periods $\pi /N\gamma_{n}B_{0}^{\max}$, where *N* is the bootstrap factor. The option to fix *L_AB_* = *L_CD_* for a “symmetric” scan is also available.

The incoming wavelength distribution may be selected from rectangular, triangular, or Gaussian distributions, or else a *δ* function (pure monochromatic) symmetrically with respect to a specified nominal (true mean) wavelength, *λ_0_*. Uncertainties in the coil lengths and the static field homogeneity are handled by randomly selecting values from Gaussian distributions which are centered on the nominal values for each coil. This means that for each Monte Carlo trajectory, the uncertainty is uncorrelated with the position in the coil; however, this allows for comparison with the simple “beam-average” formulations for the resolution contributions described in Sec.6. The beam divergence is considered to be uniform and symmetric up to specified limits of the incoming beam at the entrance to the first coil and may be specified independently for *x* and *z* for a beam traveling along *y*. Thereafter, the collimation is imposed by the dimensions of the coil windows or sample (if present) though which the beam is required to pass. An option to specify divergences according to the simplified model in Sec. 6.4 is also available.

The r.f. flipper module calculates the time spent in the each coil as *t_coil_* = *l_coil_*/(*v_n_* cos*θ*), where, $\cos\theta =k_{y}/\sqrt{k_{x}^{2}+k_{y}^{2}+k_{z}^{2}}$, where *k_x_*_,(_*_y,z_*_)_ are the components of the trajectory *k*-vector (or something proportional to it) and *y* is the nominal direction of the beam. To maintain generality, the r.f. phase on entry to the first coil is generated randomly between 0 and 2*π*, simulating a continuous neutron beam, even if it turns out that this phase cancels in the final result. The r.f. frequency, *ω_rf_*, is assumed to have *negligible uncertainty* with respect to the other frequencies in the problem (i.e., is fixed for a given spectrometer setting). The magnitude of *ω_rf_* is not necessarily constrained have the value of the nominal Larmor precession frequency in the static field, $\omega_{0}^{nom}$, i.e., $\omega_{rf}=\mathrm{sgn}\;(B_{0})\left|f\omega_{0}^{nom}\right|$ but *f* = 1 is the default. Also, as mentioned previously, *ω_0_* varies depending on a randomly selected value of *δB_0_*/*B_0_* from a Gaussian distribution, where $\omega_{0}=\mathrm{sgn}\;(B_{0})\left|\omega_{0}^{nom}(1+\delta B_{0}/B_{0}^{nom})\right|$. This value of *ω_0_* affects the entire trajectory passage through a given coil. In order to limit the number of variables in the problem, |*B_rf_*| is set to the nominal value which produces exact *π*-flips for the chosen nominal wavelength, *λ_0_*, and nominal coil length.

The coordinate transformations are handled as follows:

1. The initial polarization at the entrance to coil 1 is defined parallel to the *x*-axis, i.e.,
  $$P_{in}=\left[\begin{array}{l} 1\\ 0\\ 0 \end{array}\right]$$
2. The coordinate system is transformed around *z* so that *x*′ points along the direction of the applied resonant component of the r.f. field vector in the *xy* plane at the entrance to the coil, $B_{\mathrm{rf}}^{\mathrm{in}}$, i.e., along the direction defined by the phase angle $\psi_{rf}^{in}=\mathrm{sgn}\;(B_{0})\left|\psi_{rf}^{in}\right|$. If this is the first coil it is simply the randomly generated value between 0 and 2*π*. The transformation matrix (with the convention that a positive rotation about the positive *z*-axis is “positive *x* axis moving towards positive *y*-axis”) is:
  $$T_{1}=\left(\begin{array}{ccc} \cos\;\psi_{rf}^{in} & \sin\;\psi_{rf}^{in} & 0\\ -\sin\;\psi_{rf}^{in} & \cos\;\psi_{rf}^{in} & 0\\ 0 & 0 & 1 \end{array}\right).$$
  If *ω_rf_* ≠ *ω_0_* (which is the case in general if *δB_0_* ≠ 0, even if *f* is set to 1), the effective Larmor precession frequency, $\omega_{p}^{eff}$, around the effective field in the rotating coordinate system and the corresponding angular departure of the effective field from the *xy* plane, *α_eff_*, are calculated according to Ref. [15] as
  $$\omega_{p}^{eff}=\gamma_{n}\left|B_{rf}^{eff}\right|=\left|\sqrt{{\left(\omega_{0}-\omega_{rf}\right)}^{2}+\omega_{p}^{2}}\right|,$$
  where *ω_p_* =*γ_n_* |*B_rf_*|, as before, and
  $$\alpha_{eff}=\tan^{-1}\frac{\omega_{0}-\omega_{rf}}{\omega_{p}}.$$
  Note that $\omega_{p}^{eff}=2\omega_{A}$ in Ref. [13].
  The coordinate system is then rotated by -*α_eff_* about *y*′ (where the −sign ensures that a positive value of *α_eff_* rotates the +*x*′ axis toward the +*z*′ axis) such that *x*″ points along $B_{\mathrm{rf}}^{\mathrm{eff}}$, according to
  $$T_{2}=\left(\begin{array}{ccc} \cos\;\alpha_{eff} & 0 & \sin\;\alpha_{eff}\\ 0 & 1 & 0\\ -\sin\;\alpha_{eff} & 0 & \cos\;\alpha_{eff} \end{array}\right),$$
  so that the compound transformation from the lab frame to the rotating $B_{rf}^{eff}$ frame is
  $$T_{2}T_{1}=\left(\begin{array}{ccc} \cos\;\alpha_{eff}\cos\;\psi_{rf}^{in} & \cos\;\alpha_{eff}\sin\;\psi_{rf}^{in} & \sin\;\alpha_{eff}\\ -\sin\;\psi_{rf}^{in} & \cos\;\psi_{rf}^{in} & 0\\ -\sin\;\alpha_{eff}\cos\;\psi_{rf}^{in} & -\sin\;\alpha_{eff}\sin\;\psi_{rf}^{in} & \cos\;\alpha_{eff} \end{array}\right).$$
  Its inverse, (*T*_2_*T*_1_)^−1^, is also calculated to transform from the rotating frame back to the lab frame. (Note that for exact resonance (*ω_rf_* = *ω_0_*), *T_2_* is just the identity matrix and *T_2_T_1_* ≡ *T_1_*).
3. The total Larmor precession by an angle $\gamma_{n}\left|B_{rf}^{eff}\right|t_{coil}=\omega_{p}^{eff}t_{coil}$ is performed around $B_{rf}^{eff}$ (i.e., the *x*″ axis). Note that $\omega_{p}^{eff}$ is always positive but because we are now rotating the *object* (the neutron magnetic moment) rather than the *axes*, the transformation is like an axis rotation by $-\omega_{p}^{eff}t_{coil}$); the transformation matrix is
  $$T_{3}=\left(\begin{array}{ccc} 1 & 0 & 0\\ 0 & \cos\;\omega_{p}^{eff}t_{coil} & -\sin\;\omega_{p}^{eff}t_{coil}\\ 0 & \sin\;\omega_{p}^{eff}t_{coil} & \cos\;\omega_{p}^{eff}t_{coil} \end{array}\right),$$
  so that the compound transformation of the spin is
  $$T_{3}T_{2}T_{1}=\;\left(\begin{array}{ccc} \cos\;\alpha_{eff}\cos\;\psi_{rf}^{in} & \cos\;\alpha_{eff}\psi_{rf}^{in} & \sin\;\alpha_{eff}\\ \left(\begin{array}{l} \sin\;\alpha_{eff}\cos\;\psi_{rf}^{in}\sin\;\omega_{p}^{eff}t_{coil}\\ -\sin\;\psi_{rf}^{in}\cos\;\omega_{p}^{eff}t_{coil} \end{array}\right) & \left(\begin{array}{l} \sin\;\alpha_{eff}\sin\;\psi_{rf}^{in}\sin\;\omega_{p}^{eff}t_{coil}\\ +\cos\;\psi_{rf}^{in}\cos\;\omega_{rf}^{eff}t_{coil} \end{array}\right) & -\cos\;\alpha_{eff}\sin\;\omega_{p}^{eff}t_{coil}\\ -\left(\begin{array}{l} \sin\;\alpha_{eff}\cos\;\psi_{rf}^{in}\cos\;\omega_{p}^{eff}t_{coil}\\ +\sin\;\psi_{rf}^{in}\sin\;\omega_{p}^{eff}t_{coil} \end{array}\right) & \left(\begin{array}{l} \cos\;\psi_{rf}^{in}\sin\;\omega_{p}^{eff}t_{coil}\\ -\sin\;\alpha_{eff}\sin\;\psi_{rf}^{in}\cos\;\omega_{p}^{eff}t_{coil} \end{array}\right) & \cos\;\alpha_{eff}\cos\;\omega_{p}^{eff}t_{coil} \end{array}\right).$$
  The coil dispersion is accounted for at this stage.
4. Returning to the lab frame the spin transformation thus far is (*T*_2_*T*_1_)^−1^ *T*_3_*T*_2_*T*_1_.
5. Finally, we add on the applied r.f. field rotation (around the *z* axis) to the position of the magnetic moment during passage of the neutron through the coil via
  $$T_{4}=\left(\begin{array}{ccc} \cos\;\omega_{rf}t_{coil} & -\sin\;\omega_{rf}t_{coil} & 0\\ \sin\;\omega_{rf}t_{coil} & \cos\;\omega_{rf}t_{coil} & 0\\ 0 & 0 & 1 \end{array}\right),$$
  where *ω_rf_* = sgn (*B*_0_)|*ω_rf_*| which accounts for the sense of rotation of the resonant component in the particular coil. Note again that the *object* is rotated and the lab frame coordinate axes are left alone so that this matrix is equivalent to a coordinate rotation by −*ω_rf_ t_coil_*.
  The output of the flipper coil module is the output polarization vector:
  $$P_{out}=T_{4}{\left(T_{2}T_{1}\right)}^{-1}T_{3}T_{2}T_{1}P_{in}$$
  and the magnitude of the r.f. field phase at the exit of the coil:
  $$\left|\psi_{rf}^{out}\right|=\left|\psi_{rf}^{in}\right|+\left|\omega_{rf}\right|t_{coil}.$$

In the zero-field drift paths between the coils, no spin transformation is performed but the magnitude of the r.f. phase is advanced according to the time of flight in the drift path, *t_path_* = *L*/(*v_n_* cos*θ*), where *L* is measured along the *y*-axis, (i.e., $\left|\psi_{rf}^{f}\right|=\left|\psi_{rf}^{i}\right|+\left|\omega_{rf}\right|t_{path}$). $\left|\psi_{rf}^{f}\right|$ then becomes the input r.f. phase magnitude for the next coil. The polarization represented by Eq. (140) is (in this case) the mean value of the component *P_x_* at the exit of the final coil averaged over many trajectories. Stray field effects between coils are not accounted for. For magnetically-shielded symmetric Bootstrap configurations, this is not a bad approximation; for other configurations it assumes adiabatic passage of the neutron through these regions.

For quasielastic neutron scattering simulations, the specific case of self-diffusion at low *Q* is treated. In this approximation the quasielastic half energy width at half maximum given by

(302) $$\Gamma (Q)=\hbar DQ^{2},$$

where *D* is the (specified) diffusion coefficient in units of area per unit time (see Sec. 5.3). In order to simplify the Monte Carlo selection of a quasi-elastic energy transfer from a Lorentzian distribution, *Q* is calculated as the *elastic Q* value from the randomly selected incident wavelength and a specified fixed scattering angle, *θ_s_*, according to

(303) $$Q\approx Q_{el}=4\pi \sin\;\theta_{s}/\lambda_{i},$$

i.e., for the selection of *Q* only, the very small change in wavelength due to the scattering is ignored. For example, for an incident wavelength *λ_i_* = 8 Å (*E_i_* = 1.278 meV) with a typical NRSE energy transfer of 0.025 *μ*eV, *Δλ*/*λ_i_* ≈ ½ *ΔE*/*E_i_* ≈ 10^−5^, i.e., the *Q*-value is accurate to about 10^−3^ % which is very much smaller than the incident wavelength bandwidth, which is of order several %. Also the distribution of *θ_s_* in a real situation would broaden *Q* significantly more than 10^−3^ %. With the chosen value of *Γ*(*Q*), a Lorentzian-distributed energy transfer is randomly selected according to

(304) ℏω=−Γ(Q)tan[π(ran{−0.5,0.5})],

subject to the maximum sample energy gain restriction *ħω* ≤ *E_i_*, where *E_i_* is the incident neutron energy. Finally, the scattered neutron wavelength (velocity) is calculated from the incident wavelength and the randomly selected value of *ħω*. The resulting value is used for propagation of the neutron downstream of the sample position.

### 8.2 Numerical Verification of Analytical Approximations for Coil Dispersion

**Fig. 36:**
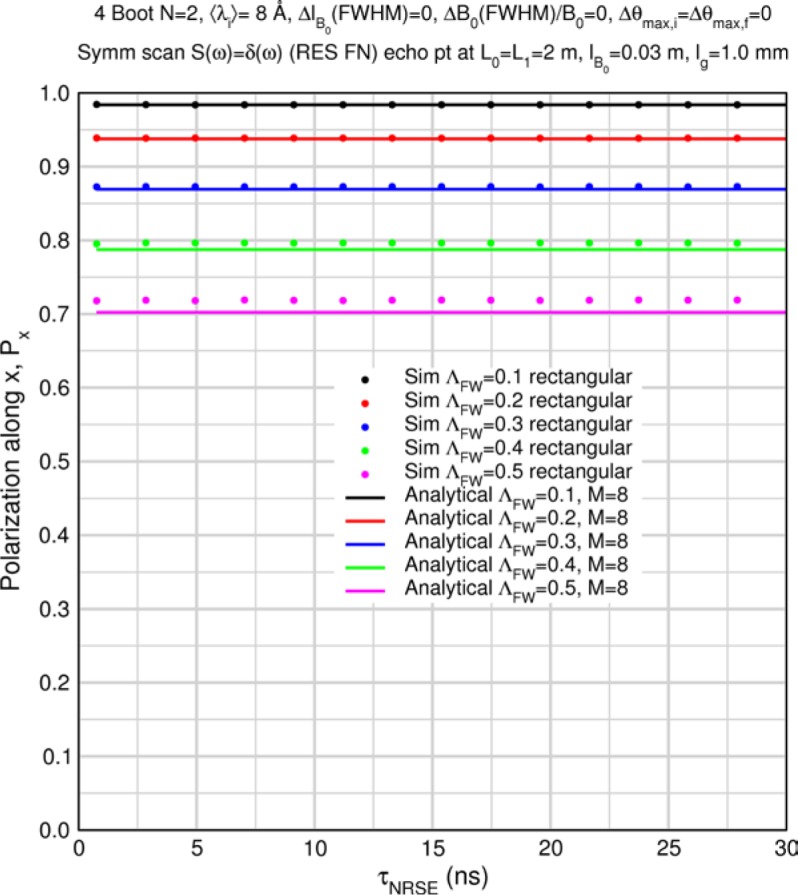
Simulated effects of flipper coil dispersion for rectangular incident wavelength spectra compared with analytical approximations using Eq. (42) (see Sec. 2.2.2) for various values of $\Lambda_{FW}=\Delta \lambda_{i}^{FW}/\langle \lambda_{i}\rangle$, for a “perfect” instrument (*ΔB_0_* = 0, *Δl_B0_* = 0, *Δθ_i,max_* = *Δθ_f,max_* = 0) and for elastic scattering (resolution function).

**Fig. 37:**
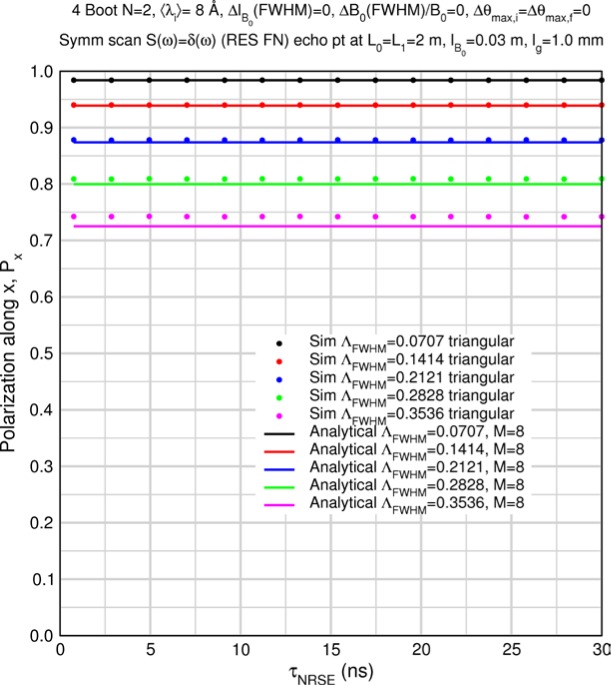
Simulated effects of flipper coil dispersion for triangular incident wavelength spectra compared with analytical approximations using Eq. (43) (see Sec. 2.2.2) for various values of $\Lambda_{FWHM}=\Delta \lambda_{i}^{FWHM}/\langle \lambda_{i}\rangle$, for a “perfect” instrument (*ΔB_0_* = 0, *Δl_B0_* = 0, *Δθ_i,max_* = *Δθ_f,max_* = 0) and for elastic scattering (resolution function). The values of *Λ_FWHM_* are chosen to give the same rms deviation with respect to 〈*λ_i_*〉 as the rectangular spectrum cases shown in Fig. 36 and are numerically equal to *Λ_FW_*(rect)/√2.

**Fig. 38:**
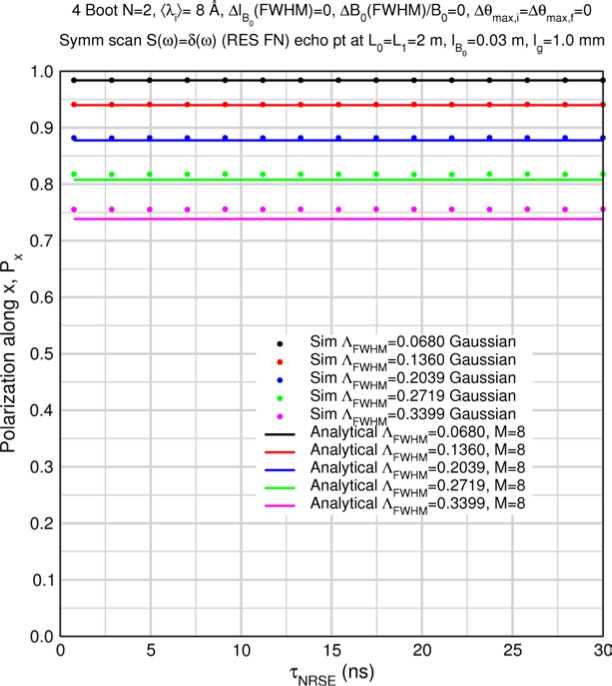
Simulated effects of flipper coil dispersion for Gaussian incident wavelength spectra compared with analytical approximations using Eq. (44) (see Sec. 2.2.2) for various values of $\Lambda_{FWHM}=\Delta \lambda_{i}^{FWHM}/\langle \lambda_{i}\rangle$, for a “perfect” instrument (*ΔB_0_* = 0, *Δl_B0_* = 0, *Δθ_i,max_* = *Δθ_f,max_* = 0) and for elastic scattering (resolution function). The values of *Λ_FWHM_* are chosen to give the same rms deviation with respect to 〈*λ_i_*〉 as the rectangular spectrum cases shown in Fig. 36 and are numerically equal to √((2/3)ln2)*Λ_FW_*(rect).

The approximations represented by Eqs. (42–44) (see Sec. 2.2.2) describe dispersion-induced depolarization after passage through *M* coils for rectangular, triangular, and Gaussian-shaped incident wavelength spectra respectively. In order to isolate dispersive effects in the Monte Carlo simulations, all other instrumental imperfections (coil dimension errors, field inhomogeneity, and beam divergence) are switched *off*, and the model has “perfect” polarizers and no sample. Results for rectangular wavelength spectra with $\Lambda_{FW}=\Delta \lambda_{i}^{FW}/\langle \lambda_{i}\rangle$ between 0.1 and 0.5 are shown in Fig. 36. For ease of comparison, results for triangular and Gaussian wavelength spectra are plotted with values of $\Lambda_{FWHM}=\Delta \lambda_{i}^{FWHM}/\langle \lambda_{i}\rangle$ which give equivalent *rms* wavelength deviations about the mean as the rectangular cases, i.e., the triangular spectra have *Λ_FWHM_* = *Λ_FW_*/√2, and the Gaussian spectra have *Λ_FWHM_* = √((2/3)ln2)*Λ_FW_* (see also Table 1). These are shown in Fig. 37 and Fig. 38 respectively. The results corresponding to *Λ_FW_* = 0.1 (black symbols and curves) show that the approximations made in Sec. 2.2.2 for extending the single coil case to the *M*-coil case agree with the simulations to within about 0.01 % for all spectral shapes. For the results corresponding to *Λ_FW_* = 0.2 (red symbols and curves), the agreement is at about the 0.1 % level, and for the *Λ_FW_* = 0.5 family (magenta symbols and curves), the approximations agree to about 2.3 % for all spectral shapes. For the perfect spectrometer, the loss of echo signal due to dispersion appears to be independent of *B_0_* (*τ_NRSE_*) for all practical cases.

### 8.3 Effects of Field Inhomogeneity, Coil Length Uncertainty, and Beam Divergence (Simplified Divergence Model) in the Absence of Flipper Dispersion

**Fig. 39:**
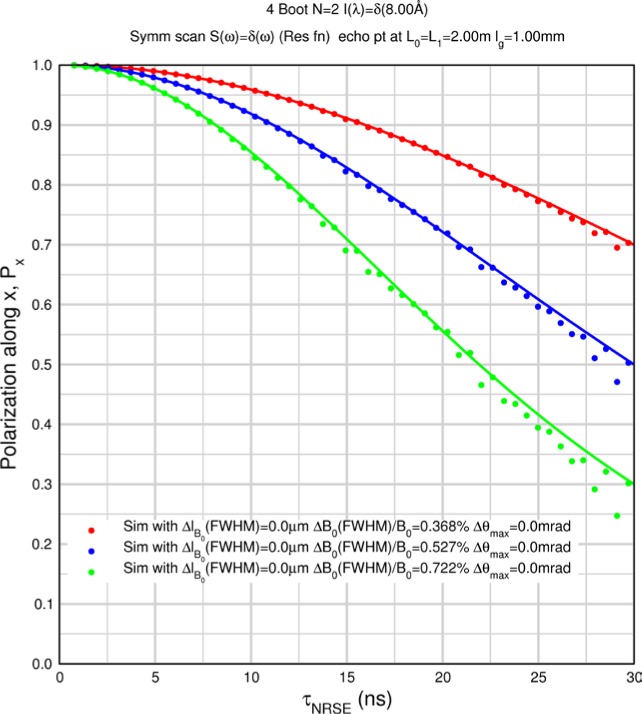
Simulations of the effect of *ΔB_0_* in isolation for a 4-*N=2* coil spectrometer with *L_0_* = *L_1_* = 2 m, *l_B0_* = 0.03 m, and incident spectrum *I*(*λ*) = *δ*(8Å). The plot shows simulations (circular symbols) for three fixed values of *ΔB_0_*/*B_0_* with Gaussian distributions. These were chosen such that the *P_x_*^0^ predicted by Eq. (178) are 0.7 (red), 0.5 (blue), and 0.3 (green) when *B_0_* =0.0393 T (corresponding to *τ_NRSE_* = 30 ns for these instrument parameters). The implied values of *ΔB_0_*/*B_0_*(FWHM) are 0.368 %, 0.527 %, and 0.722 % respectively. The solid curves are Eq. (176) which is the inverse representation of Eq. (178).

**Fig. 40:**
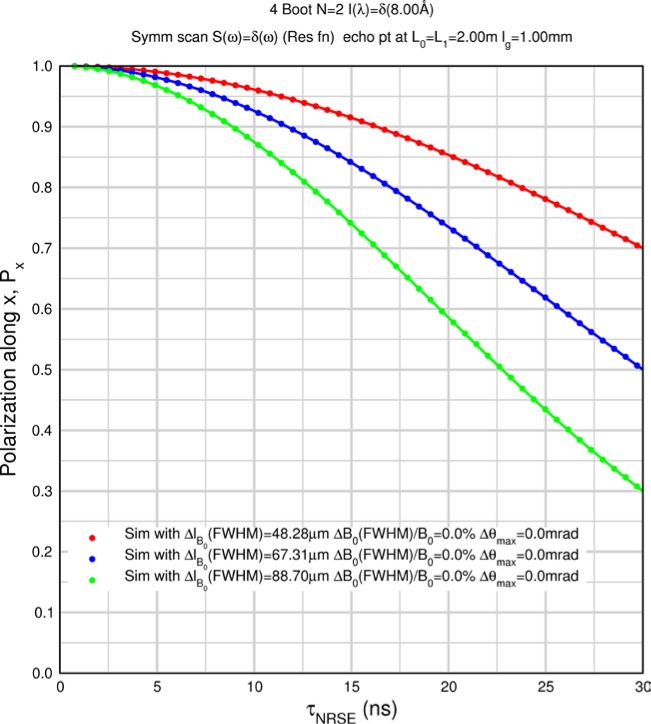
Simulations of the effect of *Δl_B0_* in isolation for a 4-*N=2* coil spectrometer with *L_0_* = *L_1_* = 2 m, *l_B0_* = 0.03 m, and incident spectrum *I*(*λ*) = *δ*(8Å). The plot shows simulations (circular symbols) for three fixed values of *Δl_B0_* (FWHM) with Gaussian distributions. These were chosen such that the *P_x_*^0^ predicted by Eq. (184) are 0.7 (red), 0.5 (blue), and 0.3 (green) when *B_0_* =0.0393 T (*τ_NRSE_* = 30 ns for these instrument parameters). The corresponding values of *Δl_B0_*(FWHM) are 48.3 *μ*m, 67.3 *μ*m, and 88.7 *μ*m respectively. The solid curves are Eq. (185), which is the inverse representation of Eq. (184).

**Fig. 41:**
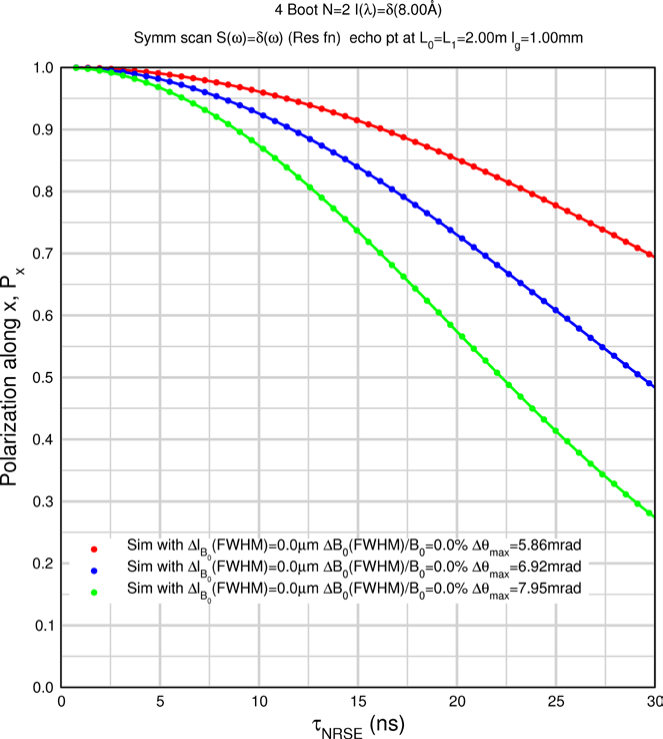
Simulations of the effect of *Δθ_max_* in isolation for a 4-*N=2* coil spectrometer with *L_0_* = *L_1_* = 2 m, *l_B0_* = 0.03 m, and incident spectrum *I*(*λ*) = *δ*(8Å). In this case *Δθ_i,max_* = *Δθ_f,max_* = *Δθ_max_*, such that Eq. (197) applies. The plot shows simulations (circular symbols) for three values of *Δθ_max_* with uniform distributions of *θ* up to these values. The *Δθ_max_* were chosen such that the *P_x_*^0^ predicted by the approximate inverse equation (202) are 0.7 (red), 0.5 (blue), and 0.3 (green) when *B_0_* =0.0393 T (*τ_NRSE_* = 30 ns for these instrument parameters). The corresponding values of *Δθ_max_* are 5.86 mrad, 6.92 mrad, and 7.95 mrad respectively. The solid curves are Eq. (197), with Fresnel integrals evaluated numerically.

**Fig. 42:**
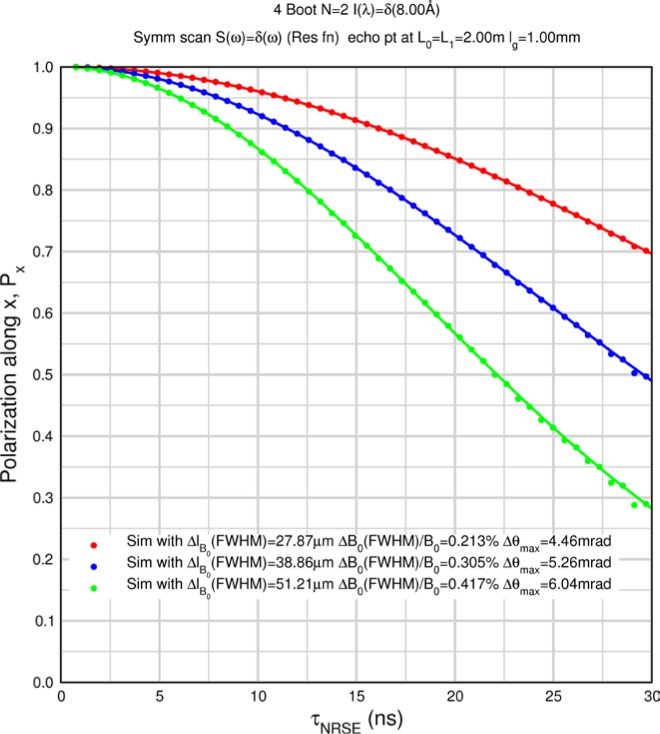
Simulations of the combined resolution effects of field inhomogeneity, coil length uncertainty, and beam divergence for a 4-*N=2* coil spectrometer with *L_0_* = *L_1_* = 2 m, *l_B0_* = 0.03 m, and incident spectrum *I*(*λ*) = *δ*(8Å). *ΔB_0_*(FWHM), *Δl_B0_* (FWHM), and *Δθ_max_* (simplified divergence model with *Δθ_i,max_* = *Δθ_f,max_*) were chosen according to Eqs. (203), (204), and (205) to yield *P_x_*^0^ values of 0.7 (red), 0.5 (blue), and 0.3 (green) when *B_0_* =0.0393 T (*τ_NRSE_* = 30 ns for these instrument parameters). The corresponding fixed *ΔB_0_*(FWHM)/*B_0_*, *Δl_B0_* (FWHM), and *Δθ_max_* values are shown in the legend. The simulation results are represented by the circular symbols. The solid lines are analytical approximations obtained by substituting *ΔB_0_*(FWHM), *Δl_B0_* (FWHM), and *Δθ_max_* into Eqs. (176), (185), and (197) respectively and taking their product.

**Fig. 43:**
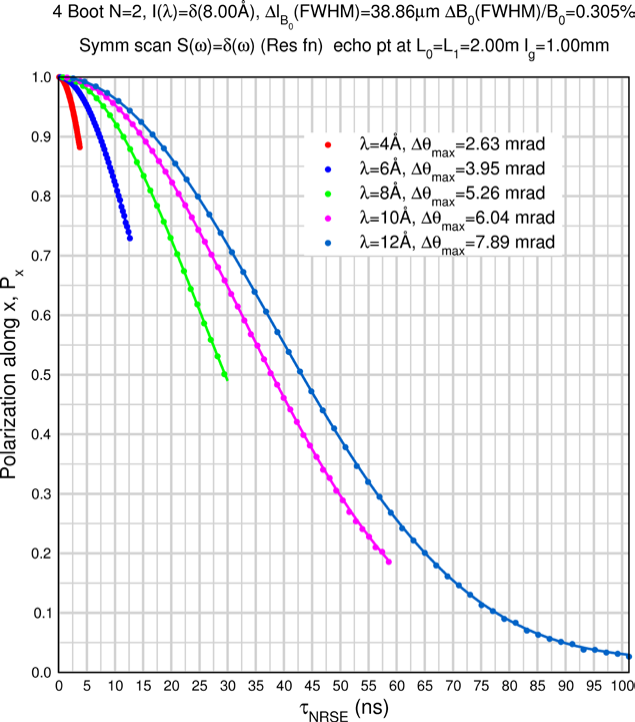
Simulated wavelength-dependence of the spectrometer resolution function. In this example *ΔB_0_* and *Δl_B0_* yield *P_x_*^0^ (*τ_NRSE_* = 30 ns) = 0.5 for *λ* = 8Å according to Eqs. (203) and (204). The divergence (equal in magnitude for the incident and scattered beams and proportional to wavelength) is calculated using a value of 0.658 mrad Å^−1^, which is the value inferred from Eq. (205) for *λ* = 8 Å when *P_x_*^0^ (*τ_NRSE_* = 30 ns) = 0.5. Thus the curves for *λ* = 8Å (green) are equivalent to the blue curves in Fig. 42. The situation is roughly equivalent to that of a beam from an uncoated polished glass neutron guide, with no scattering at the sample. The simulation results are represented by circular symbols. The analytical approximations, represented by the solid curves, are obtained by substituting *ΔB_0_*(FWHM), *Δl_B0_* (FWHM), and *Δθ_max_* into Eqs. (176), (185), and (197) respectively and taking their product. The maximum *τ_NRSE_* of each curve corresponds to *B_0_* =0.0393 T.

For *all* calculations in Sec. 8.3 the simplified beam divergence model described in Sec. 6.4 is adopted and incident and scattered beams are assumed to have uniform divergence of the same magnitude. Furthermore, all effects of flipper dispersion are effectively switched off by choosing a purely monochromatic incident beam with no subsequent energy changes. This means that the simulated polarizations tend to unity as *τ_NRSE_* → 0. The spectrometer configuration in each case is 4-*N*=2 bootstrap coils, with *L_0_* = *L_1_* = 2 m, *l_B0_* = 0.03 m, and an incident spectrum *I*(λ) = *δ* (8Å). Figure 39 to Fig. 43 show instrumental resolution functions. Figure 39 shows the effect of *ΔB_0_* in isolation. The Gaussian FWHM *ΔB_0_*/*B_0_* values for the simulations are estimated from Eq. (178) to give values of *P_x_*^0^ at *B_0_* = 0.0393 T (*τ_NRSE_* = 30 ns) of 0.7 (red curves), 0.5 (blue curves), and 0.3 (green curves), which fix the *ΔB_0_*/*B_0_* values at 0.368 %, 0.527 %, and 0.722 % respectively. The simulation results are represented by the circular symbols and the solid lines represent Eq. (176), with the chosen values of *P_x_*^0^ at *τ_NRSE_* = 30 ns. Note that Eq. (176) is the inverse representation of Eq. (178). Figure 40 shows the effect of *Δl_B0_* in isolation. In analogy with Fig. 39, values of *Δl_B0_* were chosen that yield *P_x_*^0^ = 0.7, 0.5, and 0.3 at *B_0_* = 0.0393 T (*τ_NRSE_* = 30 ns) according to Eq. (184). The simulations are represented by the circular symbols and the solid curves are Eq. (185), which is the inverse representation of Eq. (184). It is clear that Eq. (185) very accurately describes the simulation results to quite low polarizations. Figure 41 shows the effect of *Δθ* in isolation. In this example, the incoming and outgoing divergences are independent but identical in magnitude and generated according to the simplified model. In analogy with the previous figures, values of *Δθ_max_* were chosen such that *P_x_*^0^ = 0.7, 0.5, and 0.3 when *B_0_* = 0.0393 T (*τ_NRSE_* = 30 ns) according to Eq. (202). Note that Eq. (202) is an approximate inversion of Eq. (197), as described in Sec. 6.4. The simulations are represented by the circular symbols and the solid curves are Eq. (197), which very accurately describes the simulated points. In Fig. 42 the simulation combines resolution effects with approximately equal contributions from field inhomogeneity, coil length uncertainty, and beam divergence, calculated according to Eqs. (203–205), with *P_x_*^0^, (*B_0_* = 0.0393 T) = 0.7, 0.5, and 0.3, such that the simulated polarization is expected to reach these values at *τ_NRSE_* = 30 ns. The specific values of *ΔB_0_*(FWHM)/*B_0_*, *Δl_B0_* (FWHM), and half-width divergence (*Δθ_max_* = *Δθ_i,max_* = *Δθ_f,max_*) so obtained are shown in the legend. The simulated data are very well described by the products of Eqs. (176), (185), and (197) with the chosen values of *ΔB_0_*, *Δl_B0_*, and *Δθ_max_* (solid curves).

**Fig. 44:**
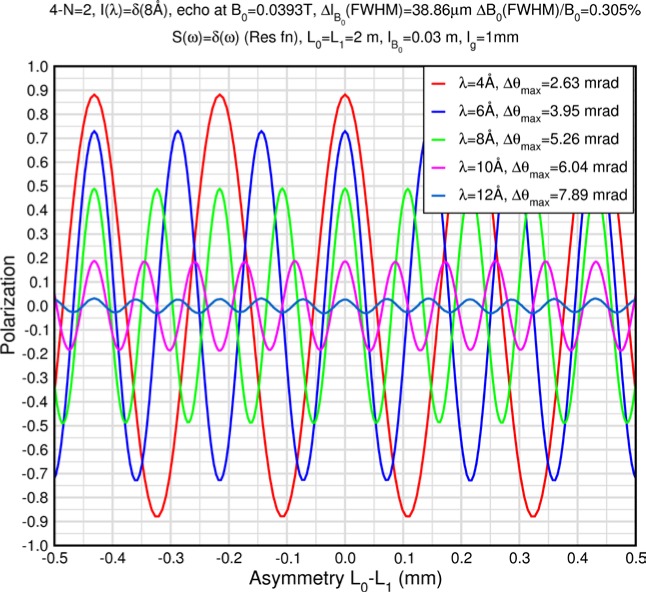
Simulated echo signals at maximum *B_0_*=0.0393 T (maximum *τ_NRSE_*) for the curves shown in Fig. 43. The simulations are fitted very precisely by the cosine form of Eq. (125) with a period inversely proportional to *λ* (Eq. (126)), apart from a pre-factor that describes the polarization loss due to the imposed instrumental imperfections.

**Fig. 45:**
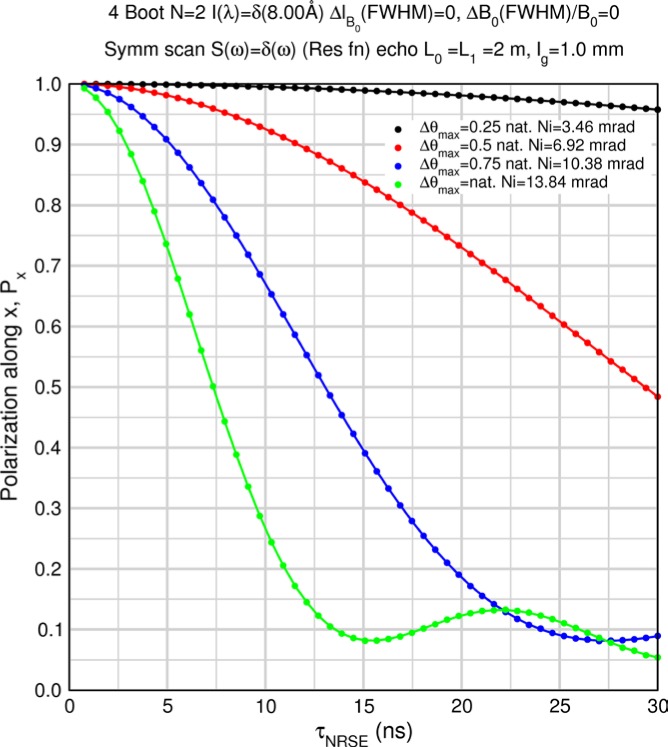
The isolated effect of incident and scattered beam divergence on the spin-echo signal for the reference spectrometer setup. The simulations (circular symbols) have *Δθ_i,max_* = *Δθ_f,max_* =*Δθ_max_* (simplified divergence model) with *Δθ_max_* = 0.25 (black), 0.5 (red), 0.75 (blue), and 1.0 (green) times the critical angle of natural Ni at $\lambda =8\;Å\;(\theta_{c}^{\text{Nat}\;\text{Ni}}(\lambda =8\;Å)=13.84\;mrad)$. No other spectrometer imperfections are included (i.e., *I*(*λ*) = *δ*(8 Å), *ΔB_0_* = *Δl_B0_* = 0). The solid curves are the corresponding results of Eq. (197), which provide excellent descriptions of the simulated data at all values of *Δθ_max_*.

Using the same *ΔB_0_* and *Δl_B0_* that yields the *P_x_*^0^ (8 Å, *τ_NRSE_* = 30 ns) = 0.5 case in Fig. 42 (*ΔB_0_*(FWHM)/*B_0_* = 0.305 %, *Δl_B0_*(FWHM) = 38.9 *μ*m), Fig. 43 shows the effect of changing wavelength. The half-width divergence (with *Δθ_max,i_* = *Δθ_max,f_* [see Fig. 16]) is determined by the same “half divergence angle per unit wavelength” (=0.658 mrad Å^−1^), so that the green curve in Fig. 43 is equivalent to the blue curve in Fig. 42. Note that 0.658 mrad Å^−1^ is a little less than is characteristic of polished glass (similar to a neutron guide with no metallic coating). The curves are plotted for identical ranges of *B_0_* from 0.001 T to 0.0393 T, but because of the *λ*^3^ –dependence of *τ_NRSE_*, the range of the abscissa is a sensitive function of wavelength. The echo signals corresponding to the cases shown in Fig. 43 at *B_0_* = 0.0393T (maximum *τ_NRSE_*) are shown in Fig. 44. Therefore, the polarizations at symmetry (*L_0_* = *L_1_*) are those of the curves in Fig. 43 at maximum *τ_NRSE_*. Note that there is no modulation of the peak magnitude when *L_0_* ≠ *L_1_* because a purely monochromatic incident beam is being simulated and that the periodicity is inversely proportional to the wavelength, as predicted by Eq. (126). Using the same reference spectrometer setup that reaches *τ_NRSE_* = 30 ns at *λ* = 8 Å, Fig. 45 demonstrates the significant suppression of the echo signal as the incident and scattered arm divergence approaches that of a natural Ni guide (≈ 1.73 mrad Å^−1^) at *λ* = 8Å. In this example *Δθ_i,max_* = *Δθ_f,max_* = *Δθ_max_* (simplified divergence model) in an otherwise perfect spectrometer (*ΔB_0_* =*Δl_B0_* =0). The simulation is for a purely monochromatic incident beam, *I*(*λ*) = *δ* (8 Å).

### 8.4 Simulations of Spectrometer Signal Revealing Flipper Coil Dispersion

All the simulations in this section adopt the same reference spectrometer configuration used previously, namely 4-*N* = 2 bootstrap coils, *L_0_* = *L_1_* = 2 m, *l_B0_* = 0.03 m, *l_g_*=1 mm, and 〈*λ_i_*〉 = 8 Å. Additionally, *ΔB_0_*(FWHM)/*B_0_* = 0.305 %, *Δl_B0_*(FWHM) = 38.9 *μ*m, and *Δθ_max,i_* = *Δθ_max,f_* = 5.26 mrad, which are the values from Eqs. (203–205) that yield *P_x_*^0^ (8 Å, *τ_NRSE_* = 30 ns) = 0.5 in the dispersionless case. However, this time flipper dispersion is included by having a non-zero incident wavelength bandwidth.

**Fig. 46:**
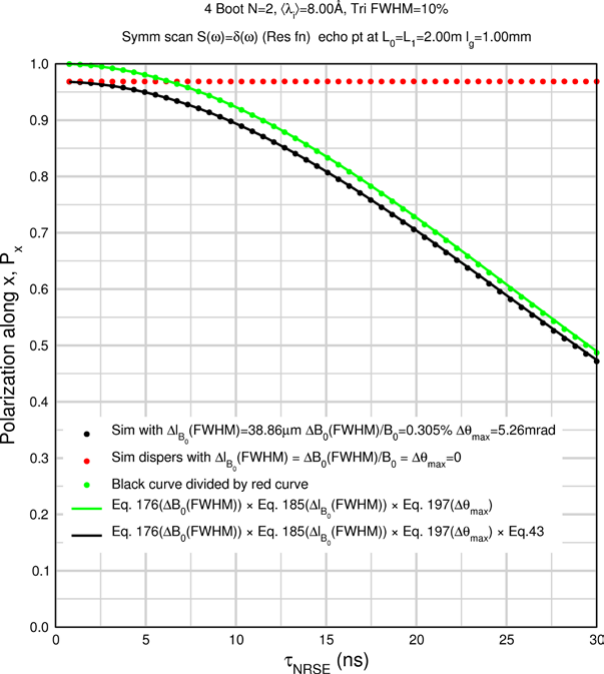
Comparison of simulated and analytical resolution functions (*S*(*ω*) = *δ*(*ω*)) revealing dispersive effects resulting from a triangular incident wavelength distribution with $\Delta \lambda_{i}^{FWHM}/\langle \lambda_{i}\rangle =10\%$ and 〈*λi*〉 = 8 Å. The simulations are performed for the reference spectrometer setup (4-*N* = 2 bootstrap coils, *L_0_* = *L_1_* = 2 m, *l_B0_* = 0.03 m, *l_g_*=1 mm): The black symbols represent the simulated dispersive resolution with *ΔB_0_*/*B_0_*, *Δl_B0_*, and *Δθ_i,max_* = *Δθ_f,max_* = *Δθ_max_*, calculated according to Eqs. (203), (204), and (205) to give a combined (dispersionless) *P_x_*^0^ (8 Å, *τ_NRSE_* = 30 ns) of 0.5. The red symbols are the simulated effect of dispersion in isolation, obtained by setting *ΔB_0_*, *Δl_B0_*, and *Δθ_max_* to zero. The green symbols represent the simulated “dispersionless” resolution, estimated by dividing the black symbols by the red symbols. This function is very well reproduced analytically by substituting the specified spectrometer imperfections into the product of Eqs. (176), (185), and (197) (solid green curve), as is the total effect (solid black curve) – obtained by multiplying the green curve by Eq. (43).

**Fig. 47:**
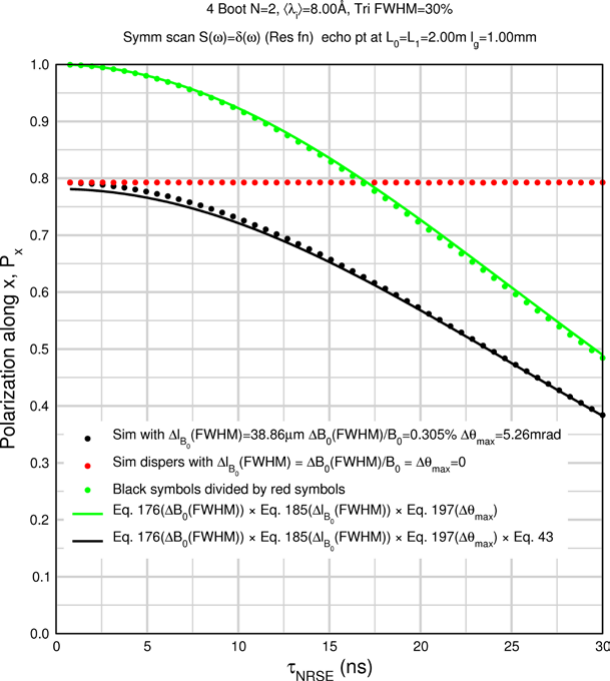
Comparison of simulated and analytical resolution functions (*S*(*ω*) = *δ*(*ω*)) revealing dispersive effects resulting from a triangular incident wavelength distribution with $\Delta \lambda_{i}^{FWHM}/\langle \lambda_{i}\rangle =30\%$ and 〈*λi*〉 = 8 Å. The simulations are performed for the reference spectrometer setup (4-*N* = 2 bootstrap coils, *L_0_* = *L_1_* = 2 m, *l_B0_* = 0.03 m, *l_g_*=1 mm): The black symbols represent the simulated dispersive resolution with *ΔB_0_*/*B_0_*, *Δl_B0_*, and *Δθ_i,max_* = *Δθ_f,max_* = *Δθ_max_*, calculated according to Eqs. (203), (204), and (205) to give a combined (dispersionless) *P_x_*^0^ (8 Å, *τ_NRSE_* = 30 ns) of 0.5. The red symbols are the simulated effect of dispersion in isolation, obtained by setting *ΔB_0_*, *Δl_B0_*, and *Δθ_max_* to zero. The green symbols represent the simulated “dispersionless” resolution, estimated by dividing the black symbols by the red symbols. This function is very well reproduced analytically by substituting the specified spectrometer imperfections into the product of Eqs. (176), (185), and (197) (solid green curve), as is the total effect (solid black curve) – obtained by multiplying the green curve by Eq. (43).

Figure 46 shows the elastic resolution function for $\Delta \lambda_{i}^{FWHM}/\langle \lambda_{i}\rangle =10\%$ (triangular) with the above instrumental uncertainties (black symbols). The red symbols represent the simulated resolution function for dispersion in isolation when $\Delta \lambda_{i}^{FWHM}/\langle \lambda_{i}\rangle =10\%$ (which is practically independent of *τ_NRSE_* – see Fig. 37). The green symbols are the result of dividing the dispersive resolution function (black symbols) by the effect of the dispersion in isolation (red symbols), which essentially reproduces the dispersionless resolution function (verified by plotting the analytical approximation (product of Eqs. (176), (185), and (197) – green solid curve). If this product is further multiplied by the *analytical* approximation for the effect of dispersion for a triangular spectral distribution (Eq. (43)), the simulation results are very well reproduced by the analytical approximation (solid black curve). Figure 47 is the exact analogue of Fig. 46 except that $\Delta \lambda_{i}^{FWHM}/\langle \lambda_{i}\rangle$ is increased from 10 % to 30 %, which exaggerates the effect of coil dispersion.

**Fig. 48:**
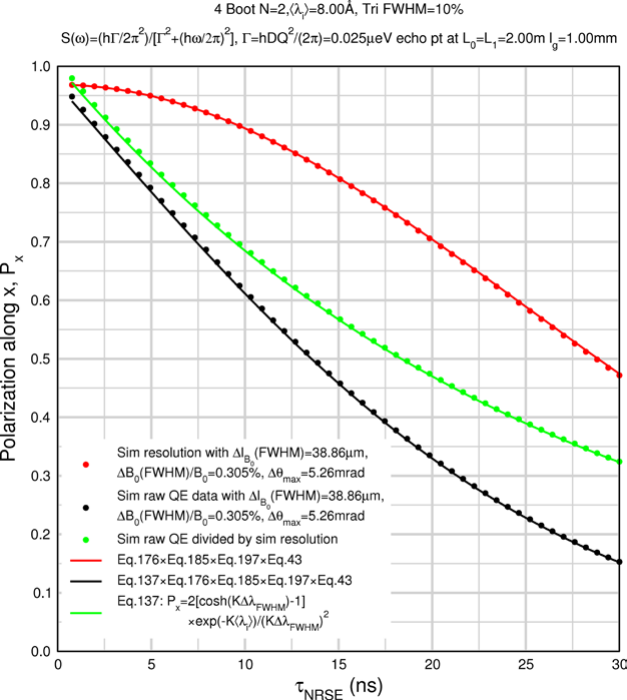
A simulated quasielastic experiment with *Γ* (HWHM) = 0.025 *μ*eV for a triangular incident wavelength band with $\Delta \lambda_{i}^{FWHM}/\langle \lambda_{i}\rangle =10\%$. The red symbols represent the simulated dispersive resolution function (equivalent to the black symbols of Fig. 46); The red solid curve is the analytical approximation to the dispersive resolution function (product of Eqs. (176), (185), (197), and Eq. (43)); the black symbols represent the simulated raw signal; the black solid curve is the analytical approximation (product of red solid curve and the theoretical intermediate scattering function for *Γ* (HWHM) = 0.025 *μ*eV [Eq. (137)]); the green symbols are the simulated resolution-corrected data (obtained by dividing the black symbols by the red symbols), which may be compared directly with Eq. (137) (solid green curve).

**Fig. 49:**
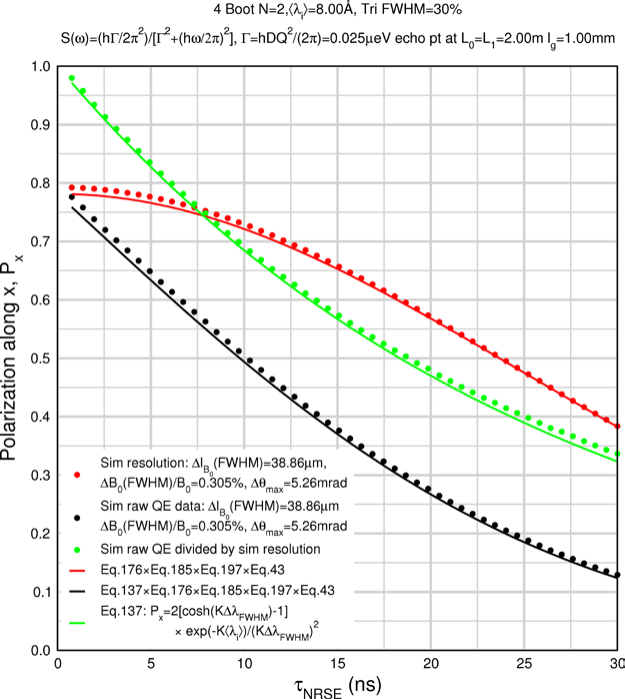
A simulated quasielastic experiment with *Γ* (HWHM) = 0.025 *μ*eV for a very coarse incident wavelength band (triangular distribution with $\Delta \lambda_{i}^{FWHM}/\langle \lambda_{i}\rangle =30\%$). The red symbols represent the simulated dispersive resolution function (equivalent to the black symbols of Fig. 46); The red solid curve is the analytical approximation to the dispersive resolution function (product of Eqs. (176), (185), (197), and Eq. (43)); the black symbols represent the simulated raw signal; the black solid curve is the analytical approximation (product of red solid curve and the theoretical intermediate scattering function for *Γ* (HWHM) = 0.025 *μ*eV [Eq. (137)]); the green symbols are the simulated resolution-corrected data (obtained by dividing the black symbols by the red symbols), which may be compared directly with Eq. (137) (solid green curve).

**Fig. 50:**
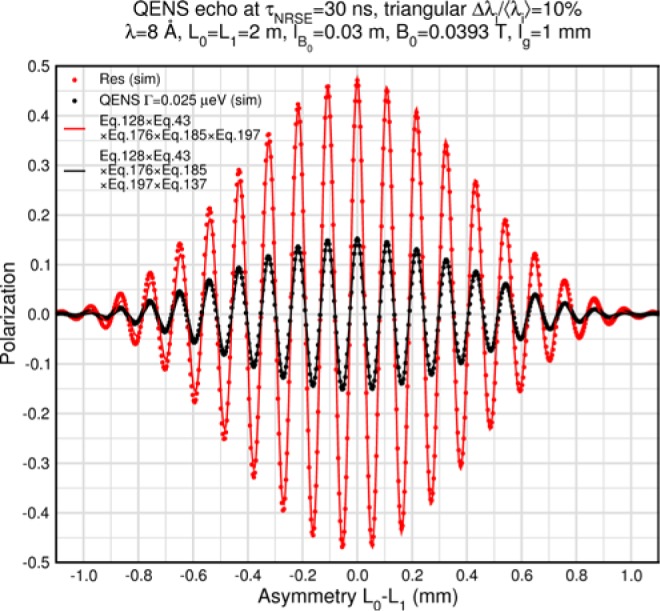
Simulated elastic resolution (red) and quasielastic (black) spin-echo signals at *τ_NRSE_* = 30ns – corresponding to the simulations shown in Fig. 48 with $\Delta \lambda_{i}^{FWHM}/\langle \lambda_{i}\rangle =10\%$ (triangular). The peak polarization at zero asymmetry should match the values at *τ_NRSE_* = 30 ns for the resolution (red symbols) and quasielastic (black symbols) in Fig. 48 within statistics. For comparison, the theoretical resolution function (product of Eqs. (128) [perfect instrument resolution echo, triangular incident spectrum], Eq. (43) [depolarization due to coil dispersion, triangular spectrum], Eq. (176), Eq. (185), and Eq. (197) for the depolarizing effects due to *ΔB_0_*, *Δl_B0_*, and *Δθ_max_* respectively) is shown (red solid curve). The black solid curve is the theoretical resolution function (red solid curve) multiplied by the theoretical depolarization due to the QENS at *δL*=0 (Eq. (137)), which describes quite well the simulated quasielastic data for this moderate value of $\Delta \lambda_{i}^{FWHM}/\langle \lambda_{i}\rangle$.

**Fig. 51:**
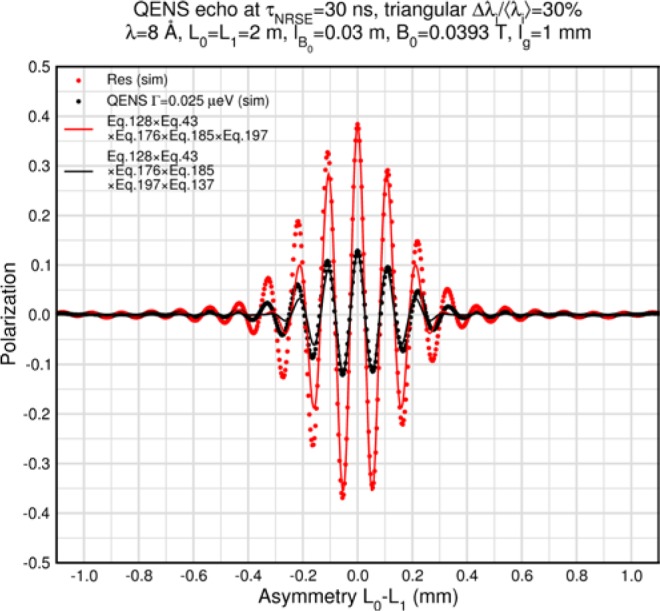
Simulated elastic resolution (red) and quasielastic (black) spin-echo signals at *τ_NRSE_* = 30ns – corresponding to the simulations shown in Fig. 49 with $\Delta \lambda_{i}^{FWHM}/\langle \lambda_{i}\rangle =30\%$ (triangular). The peak polarization at zero asymmetry should match the values at *τ_NRSE_* = 30 ns for the resolution (red symbols) and quasielastic (black symbols) in Fig. 49, within statistics. For comparison, the theoretical resolution function (product of Eqs. (128) [perfect instrument resolution echo, triangular incident spectrum], Eq. (43) [depolarization due to coil dispersion, triangular spectrum], Eq. (176), Eq. (185), and Eq. (197) for the depolarizing effects due to *ΔB_0_*, *Δl_B0_*, and *Δθ_max_* respectively) is shown (red solid curve). The black solid curve is the theoretical resolution function (red solid curve) multiplied by the theoretical depolarization due to the QENS at *δL*=0 (Eq. (137)), which approximately describes the data at small asymmetries.

Figure 48 and Fig. 49 extend the simulations shown in Fig. 46 and Fig. 47 respectively to include quasielastic scattering (with *Γ* (FWHM) = 0.025 *μ*eV). The simulated raw quasielastic signals (black symbols) divided by the resolution function (red symbols [also the black symbols in Fig. 46 and Fig. 47]) produce the green symbols. This function may be compared with the theoretical intermediate quasielastic scattering functions (Eq. (137) – continuous green curve). The main discrepancies between the theoretical functions and the simulations arise from the approximations concerning the effects of dispersion (Eq. (43)) and of *ΔB_0_* (Eq. (176)) when the cumulative out-of-*xy* plane excursions of the spin increase as the neutrons traverse multiple coils. However, even for very broad *Δλ* (30 % $\Delta \lambda_{i}^{FWHM}/\langle \lambda_{i}\rangle$ example) there remains quite reasonable agreement between the resolution-corrected simulation (green symbols) and the analytical approximation. The simulated elastic (resolution) spin-echo signal and quasi-elastic spin-echo signal (asymmetric scans) at *τ_NRSE_* = 30 ns (*B_0_* = 0.0393 T for this model - corresponding to the end points of the red and black symbols respectively in Fig. 48 and Fig. 49) are shown in Fig. 50 and Fig. 51 respectively.

### 8.5 Simulations with an Improved Divergence Model and Sample/Beam Size Effects (No Corrective Optics)

**Fig. 52:**
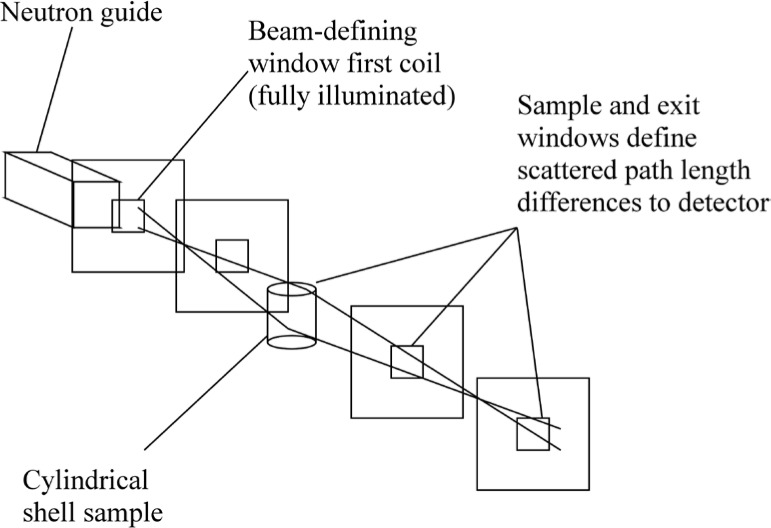
A more realistic situation for defining path length differences in the NRSE spectrometer.

In the preceding calculations (Sec. 8.3 and Sec. 8.4), the simplified beam divergence model (Sec. 6.4) was used to verify the validity of the analytical approximations given in Sec. 6. This model is useful for predicting order-of-magnitude divergence effects, however, the incident beam is usually provided by a neutron guide that gives rise to approximately random *x* and *z* components of the trajectory angle up to maxima of $\theta_{c}^{x}(\lambda_{i})$ and $\theta_{c}^{z}(\lambda_{i})$ respectively. Furthermore, the scattered beam divergence, in the absence of special optics, is usually defined by the sample size and the collimation between the sample and the detector. This more realistic situation is sketched in Fig. 52 and is the basis of the model used in the following calculations. For the polar angle *θ_i_* at the guide exit, we have

(305) $$\tan\theta_{i}=\sqrt{{\left(\tan\theta_{x}\right)}^{2}+{\left(\tan\theta_{z}\right)}^{2}},$$

or in the small angle approximation

(306) $$\theta_{i}=\sqrt{\theta_{x}^{2}+\theta_{z}^{2}}.$$

**Fig. 53:**
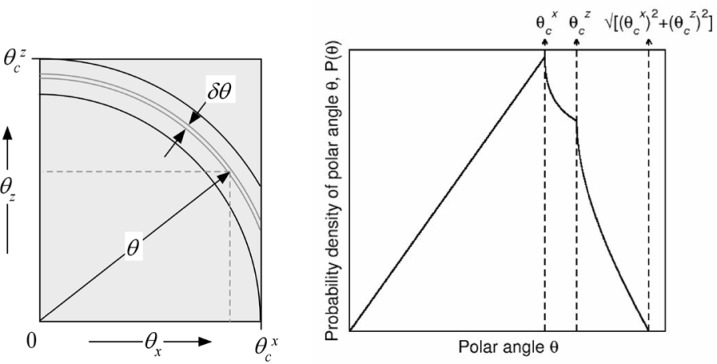
Calculation of the probability density for a polar angle *θ* from an idealized guide characterized by uniform horizontal and vertical divergence angles in the range $0\to \theta_{c}^{x}$ and $0\to \theta_{c}^{z}$ respectively.

If the spectrometer is designed to accept this angular range, the polar angle *θ_i_* also characterizes the beam divergence in the incident arm of the spectrometer. In the small angle approximation (Eq. (306)), we can readily calculate the probability density distribution of the polar angle *θ* produced by an idealized guide. This situation is illustrated in Fig. 53 for a general case where *θ_x_* ≠ *θz*. Equation (306) represents an arc of a circle of radius *θ* with origin (*θ_x_*, *θ_z_*) = (0, 0), confined within a box whose upper limits are $\theta_{x}=\theta_{c}^{x}$ and $\theta_{z}=\theta_{c}^{z}$. Because the *θ_x_* and *θ_z_* distributions are assumed uniform, the probability density for a polar angle *θ* is just proportional to the length of the arc segment of radius *θ*. Therefore, we have

(307) $$P(\theta )=\frac{\pi }{2}\frac{\theta }{\theta_{c}^{x}\theta_{c}^{z}}\;\text{for}\;\theta \leq \min\left(\theta_{c}^{x},\theta_{c}^{z}\right),$$

(308) $$P(\theta )=\left[\frac{\pi }{2}-\cos^{-1}\left(\frac{\min\left(\theta_{c}^{x},\theta_{c}^{z}\right)}{\theta }\right)\right]\frac{\theta }{\theta_{c}^{x},\theta_{c}^{z}}\;\text{for}\;\min\left(\theta_{c}^{x},\theta_{c}^{z}\right)<\theta \leq \max\left(\theta_{c}^{x},\theta_{c}^{z}\right),$$

and

(309) $$P(\theta )=\left[\frac{\pi }{2}-\cos^{-1}\left(\frac{\theta_{c}^{x}}{\theta }\right)-\cos^{-1}\left(\frac{\theta_{c}^{z}}{\theta }\right)\right]\frac{\theta }{\theta_{c}^{x},\theta_{c}^{z}}\;\text{for}\;\max\left(\theta_{c}^{x},\theta_{c}^{z}\right)<\theta \leq \sqrt{{\left(\theta_{c}^{x}\right)}^{2}+{\left(\theta_{c}^{z}\right)}^{2}},$$

where the denominator $\theta_{c}^{x}\theta_{c}^{z}$ is the area of the rectangle, which normalizes *P*(*θ*) to unit area for uniform *P*(*θ_x_*) and *P*(*θ_z_*).

The probability density distribution, *P*(*θ*), for the case illustrated on the left of Fig. 53 is shown on the right of the figure. It is immediately obvious that *P*(*θ*) is far from uniform, whence the principal weakness of the simplified divergence model (Sec. 6.4). For the simpler case of equal horizontal and vertical divergence $\theta_{c}^{x}=\theta_{c}^{z}=\theta_{c}$ and the above equations for *P*(*θ*) reduce to

(310) $$P(\theta )=\frac{\pi }{2}\frac{\theta }{\theta_{c}^{2}}\;\text{for}\;\theta \leq \theta_{c}$$

and

(311) $$P\left(\theta \right)=\left[\frac{\pi }{2}-2\cos^{-1}\left(\frac{\theta_{c}}{\theta }\right)\right]\frac{\theta }{\theta_{c}^{2}}\;\text{for}\;\theta_{c}<\theta \leq \sqrt{2}\theta_{c}.$$

**Fig. 54:**
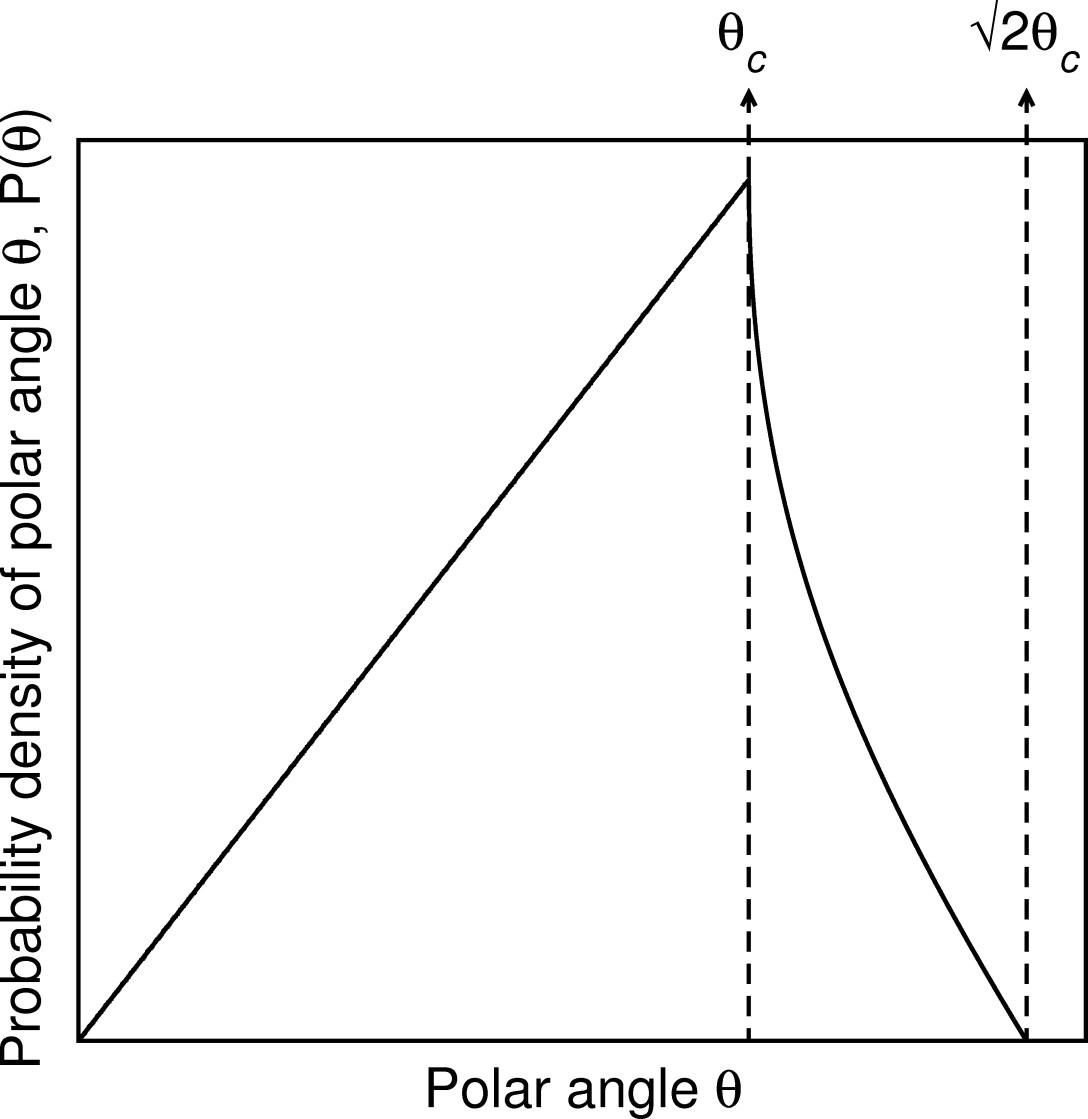
Probability density for a polar angle *θ* from an ideal neutron guide characterized by uniform and equal horizontal and vertical divergence angles in the range 0 → *θ_c_*.

The resulting function *P*(*θ*) is illustrated in Fig. 54. Therefore, clearly an improved model is necessary for more typical instrumental scenarios.

**Fig. 55:**
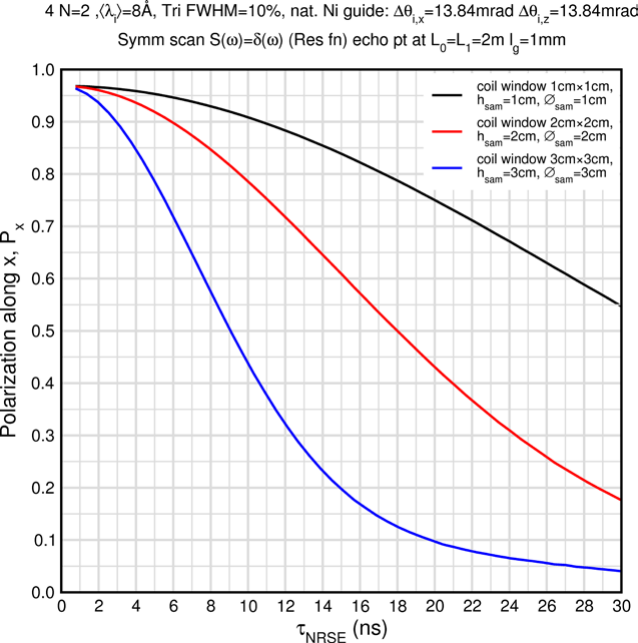
Resolution functions (*λ* = 8 Å) for the coil window and sample sizes given in the legend. For ease of comparison with previous results, the values of *ΔB_0_*/*B_0_* and *Δl_B0_*, *L_0_* and *l_B0_* are exactly those that give *P_x_*^0^ (*λ* = 8 Å, *τ_NRSE_* = 30 ns) = 0.5 in the simplified model case. The difference here is in the incoming and scattered beam divergence.

**Fig. 56:**
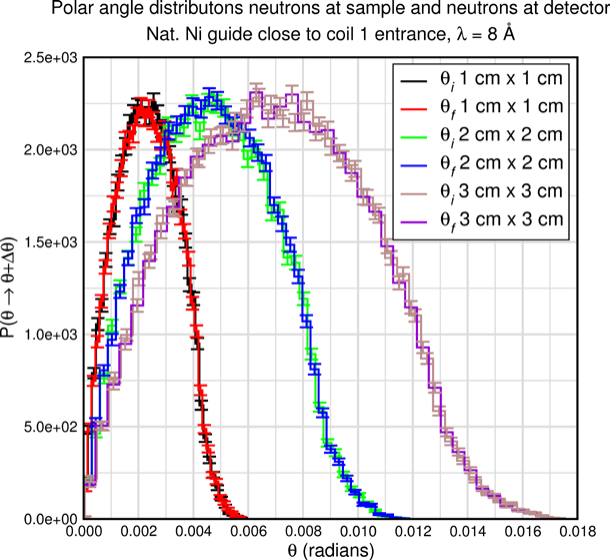
Simulated *P*(*θ*) of incident and scattered neutron trajectories that exit the final coil each side of the sample for the *λ* = 8 Å cases shown in Fig. 55. It is expected that the divergence is largely limited by the coil window sizes on both sides.

**Fig. 57:**
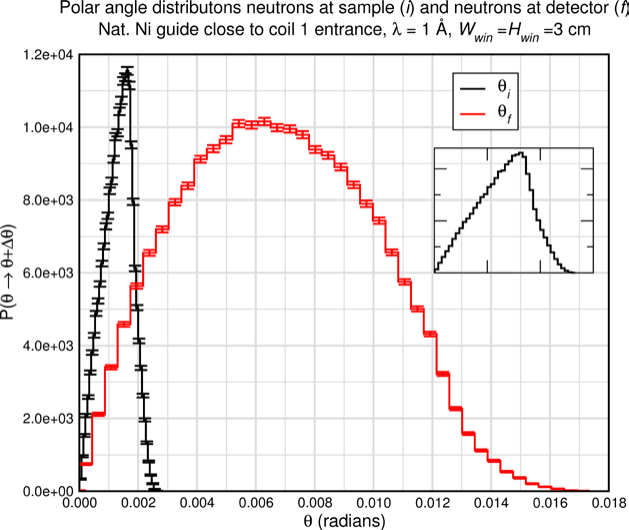
Simulated *P*(*θ*) of incident and scattered neutron trajectories that exit the final coil each side of the sample for *λ* = 1 Å with *W_win_* = *H_win_* = 3 cm. At this short wavelength it is expected that the divergence on the *incident* side is largely determined by the characteristic *P*(*θ*) of the neutron guide, whereas the *scattered* beam divergence is determined by the coil window size. The inset shows *P*(*θ_i_*) on a scale that is more easily compared with Fig. 54.

In the following all coils are assumed to have equally-sized beam-defining windows at their entrances and exits. The entrance of the first coil is assumed to be uniformly-illuminated with a beam that has uniform *x-y* and *z-y* plane angular distributions with |*θ* |up to $\theta_{c}^{x}(\lambda_{i})=\kappa_{x}\lambda_{i}$ and $\theta_{c}^{z}(\lambda_{i})=\kappa_{z}\lambda_{i}$ respectively, where *κ_x_* and *κ_z_* are independently-specified constants. The sample is assumed to be a thin cylindrical shell of radius *r* with its axis parallel to the *z*-axis. The neutron trajectories arriving at the sample are those that join random points on the first coil entrance window and random points on the sample without obstruction, subject to the maximum divergence constraints $\left|\theta_{x}|\leq \theta_{c}^{x}(\lambda_{i}\right)$ and $\left|\theta_{z}|\leq \theta_{c}^{z}(\lambda_{i}\right)$. The sample is assumed to scatter isotropically without self-shielding so that all unobstructed trajectories between the scattering point and the exit window of the final coil are equally probable and 100 % detected. Figure 55 shows example resolution functions using this model for *λ* = 8 Å with *Δλ*/*λ* = 10 % (triangular) for three coil window sizes (*w_win_* = *h_win_* = 1 cm, 2 cm, and 3 cm) assuming that a natural Ni guide (i.e., with *κ_x_* = *κ_z_* = 1.73 × 10^−3^ rad Å^−1^) is placed very close to the first coil entrance. The sample diameter, *D_sam_*, and height, *h_sam_*, in each case are chosen so that the projected sample cross-sectional area is equal to the window size (i.e., *D_sam_* = *w_win_*, *h_sam_* = *h_win_*). For ease of comparison with previous results, the spectrometer dimensions are identical to the reference (*L_0_* = 2 m, *l_B0_* = 0.03 m, *N* = 2) and *ΔB_0_*/*B_0_* and *Δl_B0_* are those that yield *P_x_*^0^ (*λ* = 8 Å, *τ_NRSE_* = 30 ns) = 0.5 with the simplified divergence model (non-dispersive case). The differences with respect to the blue curves in Fig. 42 are then attributable to the different incoming and outgoing beam divergence conditions. Note that the curve in Fig. 55 that most resembles the blue curves in Fig. 42 is for the smallest window/sample size (1 cm × 1 cm), where *w_win_*/*L_0_* ≈ 5 mrad (close to the value used [5.26 mrad] in the simplified model for *P_x_*^0^ = 0.5). For each of the three cases shown in Fig. 55, *w_win_*/*L_0_* <*θ_c_*(8Å) for *w_win_* (*h_win_*) = 1 cm, 2 cm and *w_win_*/*L_0_* ≈ *θ_c_*(8Å) for *w_win_* (*h_win_*) = 3 cm, so we expect that the coil windows/sample size more-or-less determine both the incoming and outgoing beam divergence in all three cases. The Monte-Carlo-generated *P*(*θ*) for the incoming (*i*) and scattered (*f*) trajectories of *detected* neutrons, corresponding to the cases in Fig. 55, are shown in Fig. 56. For comparison, Monte Carlo values of *P*(*θ*) for *w_win_* (*h_win_*) = 3 cm and *λ_i_* = 1 Å are shown in Fig. 57. In this case, *w_win_*/*L_0_* ≈ 9*θ_c_*(1 Å), therefore we expect that the *incoming* beam divergence is determined by the guide characteristics rather than the coil window size. Indeed, from Fig. 57 we see that *P*(*θ*) for the *incident* neutrons resembles that of the neutron guide (c.f. Fig. 54), whereas the scattered divergence is determined more by *w_win_* (*h_win_*) and resembles that of the maroon curve in Fig. 56, as expected. It is clear from Fig. 55 that, without corrective optics to narrow the flight path distribution, significant degradation of the resolution function is expected with typical neutron beam delivery systems and beam sizes, if high instrument resolution is required. Reducing *w_win_* (*h_win_*) and the incident beam divergence could significantly compromise data collection rates. However, corrective optics requirements will be considered elsewhere.

### 8.6 Resolution Effects for Asymmetrical Configurations of the Spectrometer

This section deals specifically with instrumental resolution effects in the general asymmetrical spectrometer case (*δ* (*BL*) ≠ 0). In NRSE spectrometers, it is customary to fix *B_0_* and vary *δL*, hence results are plotted in terms of *δL* = *L_0_* − *L_1_*. The resolution as a function of asymmetry relates to the range of frequencies in the scattering function that can usefully contribute to the signal, which ultimately limits the incident neutron wavelength bandwidth.

#### 8.6.1 Simulated Versus Theoretical Resolution Curves and Asymmetry-Dependence of Flipper Coil Dispersion

**Fig. 58:**
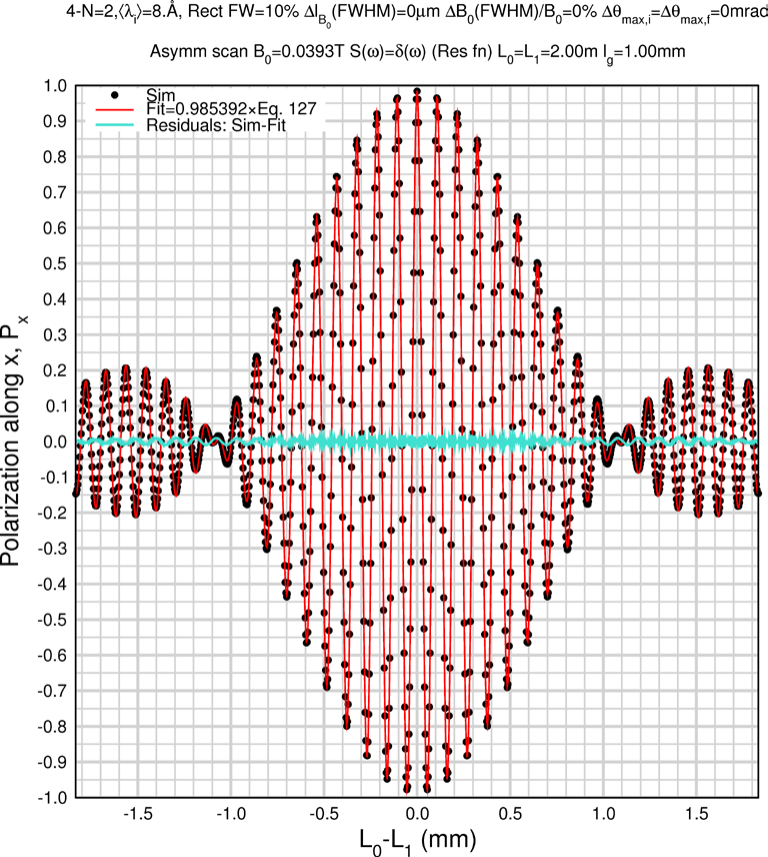
Resolution (S(*ω*)=*δ*(*ω*)) echo signal at *B_0_* = 0.0393 T for the reference spectrometer configuration (4-*N*=2, *L_1_*= 2 m etc.) with a rectangular incident wavelength distribution of full width (FW) *Δλ_i_*/〈*λ_i_*〉 = 10 % and 〈*λ_i_*〉 = 8.0 Å. The simulation is performed with *ΔB_0_* = *Δl_B0_* = *Δθ_max_* = 0, so that the only spectrometer imperfection is that due to flipper coil dispersion. The simulated signal is represented by the black circular symbols, the red curve is the least squares fit of a theoretical resolution function for a rectangular incident wavelength spectrum (Eq. (127)), multiplied by a single constant fit parameter to account for depolarization due to the combined effect of flipper coil dispersion through the eight coils. The fitted constant value (0.9854) is within 0.2 % of the value predicted by the approximate theory (Eq. (42)) of 0.9838 – see Table 1 and Fig. 36. The small residual fluctuations about zero (turquoise curve) imply that the effect of dispersion is approximately independent of the spectrometer asymmetry in this case.

**Fig. 59:**
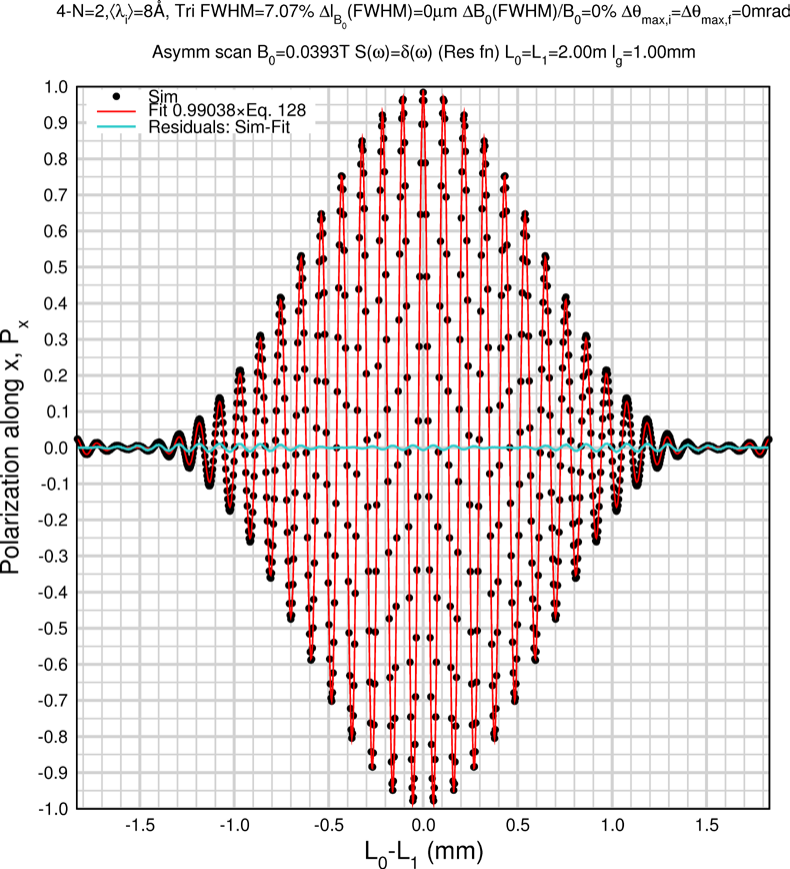
Resolution (S(*ω*)=*δ*(*ω*)) echo signal at *B_0_* = 0.0393 T for the reference spectrometer configuration (4-*N*=2, *L_1_*= 2 m etc.) with a triangular incident wavelength distribution of FWHM given by *Δλ_i_*/〈*λ_i_*〉 = 7.071 % and 〈*λ_i_*〉 = 8.0 Å. As in Fig. 58, *ΔB_0_* = *Δl_B0_* = *Δθ_max_* = 0, to isolate effects due to flipper coil dispersion. The simulated signal is represented by the black circular symbols, the red curve is the least squares fit of a theoretical resolution function for a triangular incident wavelength spectrum (Eq. (128)), multiplied by a single constant fit parameter. The fitted constant (=0.990) is within 0.7 % of the value predicted by the approximate theory (Eq. (43)) of 0.984 – see Table 1 and Fig. 37. The small residual fluctuations about zero (turquoise curve) imply that the effect of dispersion is approximately independent of the spectrometer asymmetry in this case.

**Fig. 60:**
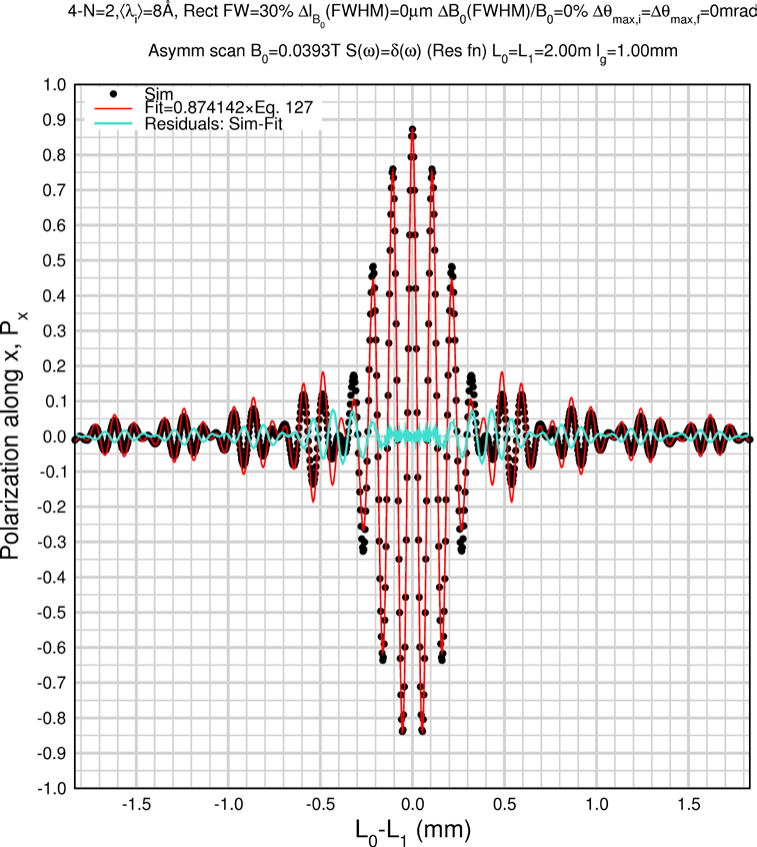
The exact analogue of Fig. 58 but with the full width of the rectangular incident wavelength spectrum increased to 30 %. Again, the black circular symbols represent the simulation, the red curve is the least squares fit of Eq. (127), multiplied by a single constant fit parameter. The fitted constant value (0.8741) is within 0.6 % of the value predicted by the approximate theory (Eq. (42)) of 0.8692 – see Table 1 and Fig. 36. The increased structure of the residuals (turquoise curve) is probably due to cumulative out-of-rotating plane excursions of the spin that is not accounted for in Eq. (127), nonetheless, there appears to be no strong asymmetry-dependence of the dispersion, even at these large values of *Δλ_i_*/〈*λ_i_*〉.

**Fig. 61:**
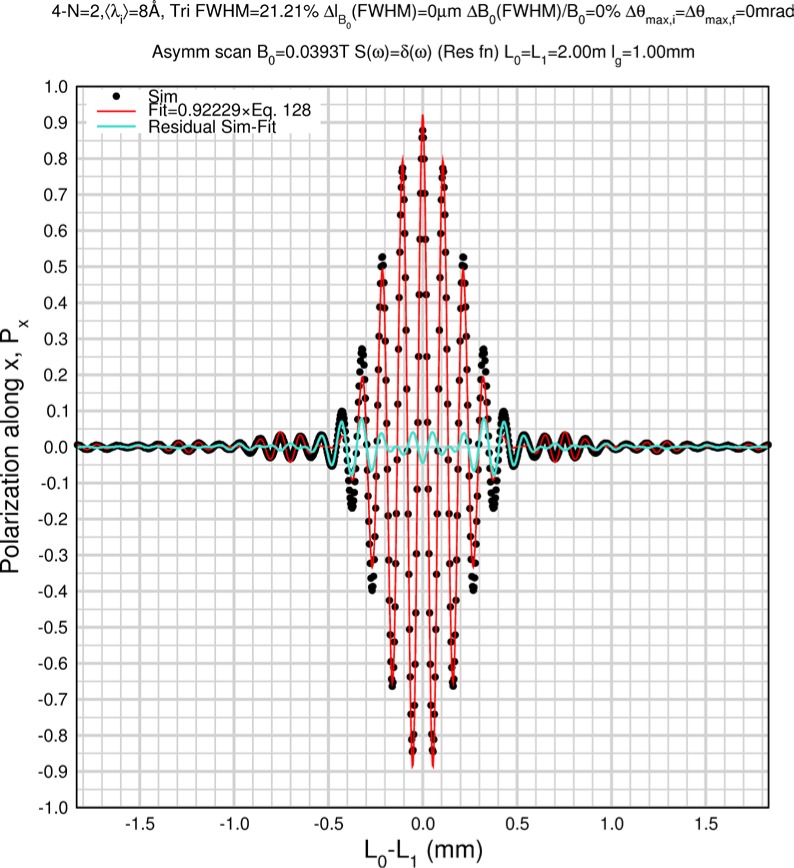
The exact analogue of Fig. 59 but with the FWHM of the triangular incident wavelength spectrum increased three times to 21.21 %. Again, the black circular symbols represent the simulation, the red curve is the least squares fit of Eq. (128)), multiplied by a single constant fit parameter. The fitted constant value (0.922) is within about 6 % of the value predicted by the approximate theory (Eq. (43)) of 0.874 – see Table 1 and Fig. 37, although clearly the data is much less well represented with only a constant fitting parameter. The increased structure of the residuals is probably due to cumulative out-of-rotating plane excursions of the spin that is not accounted for by Eq. (128), nonetheless, there appears to be no strong asymmetry-dependence of the dispersion, even at these large values of *Δλ_i_*/〈*λ_i_*〉.

In these simulations the effect of flipper coil dispersion is isolated from other forms of instrumental uncertainty by setting *ΔB_0_* = *Δl_B0_* = *Δθ_max_* = 0, i.e., flipper coil dispersion is the only instrumental imperfection. Again, the reference configuration (4-*N*=2, *L_1_* = 2 m etc.) is used for ease of comparison with other simulations and the examples fix *B_0_* = 0.0393 T and *λ_0_* = 8 Å (corresponding to *τ_NRSE_* = 30 ns). In Fig. 58 to Fig. 61, the simulated echo signals (black symbols) are compared with least-squares fits (red curves) of theoretical approximations to the resolution functions (Eq. (127) [rectangular] or Eq. (128) [triangular]) multiplied by a single, constant fit parameter. Figure 58 and Fig. 60 are for rectangular incident wavelength distributions with full width 10 % and 30 % *Δλ_i_*/〈*λ_i_*〉 respectively, whilst Fig. 59 and Fig. 61 are for triangular incident wavelength distributions with FWHM = 10/√2 % and 30/√2 % *Δλ_i_*/〈*λ_i_*〉, respectively (which give equivalent rms wavelength deviation with respect to the mean for both distributions). For the narrower band simulations, where Eq. (42) (rectangular) and Eq. (43) (triangular) describe well the effects of dispersion at *δ* (*BL*) = 0 (see Fig. 36 and Fig. 37), the fitted theoretical functions also describe well the simulated echo functions (as evidenced by the relatively small oscillations in the residuals). Therefore, the fitted constants are quite close to the values provided by these equations with *M* = 8 total coils (see also Table 1). The theoretical echo functions do not account specifically for the cumulative dispersive spin excursions out of the r.f. field plane as the neutron passes through multiple coils and the increased structure in the residuals at larger *Δλ_i_*/〈*λ_i_*〉 is likely due to this shortcoming rather than a real asymmetry-dependence of the dispersion. Nonetheless, the indications are that dispersion is only weakly dependent on the spectrometer asymmetry, if at all, under typical conditions.

#### 8.6.2 Asymmetry-Dependence of Static Field Inhomogeneity, Coil Length Uncertainty, and Beam Divergence in Typical Circumstances

**Fig. 62:**
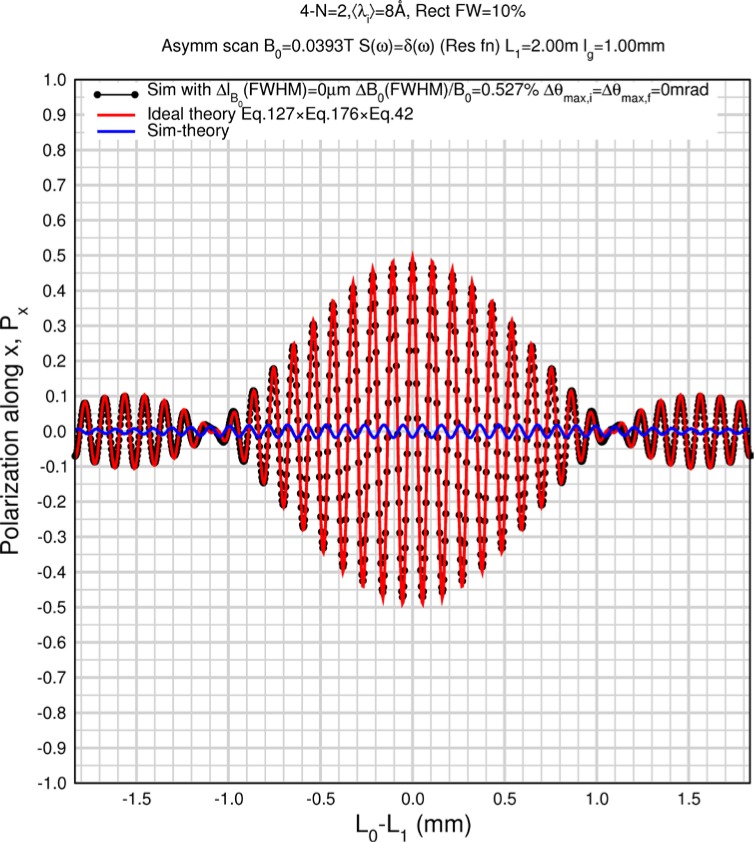
Simulated resolution echo at *B_0_* = 0.0393 T for the reference spectrometer configuration (4-*N*=2, *L_1_*= 2 m etc.) with a rectangular incident wavelength distribution of full width (FW) given by *Δλ_i_*/〈*λ_i_*〉 = 10 % and 〈*λ_i_*〉 = 8.0 Å. The simulation is performed with *Δl_B0_* = *Δθ_max_* = 0, but with *ΔB_0_* calculated according to Eq. (178) with *P_x_*^0^ = 0.5, such that the peak signal should be about 0.5 multiplied by the depolarization due to flipper coil dispersion. The red curve is the product of the theoretical “perfect instrument” resolution (Eq. (127)) multiplied by the estimated constant depolarization due to *ΔB_0_*, i.e., 0.5, (also obtained by substituting the chosen value of *ΔB_0_*(FWHM) back into Eq. (176)), multiplied by the estimated (constant) depolarization due to *Δλ* (Eq. (42)) with no fit parameters. The success of this analytical description of the simulation results in this example is revealed by the small residuals (blue curve). There is apparently no strong asymmetry-dependence of the effects of *ΔB_0_* in this case.

**Fig. 63:**
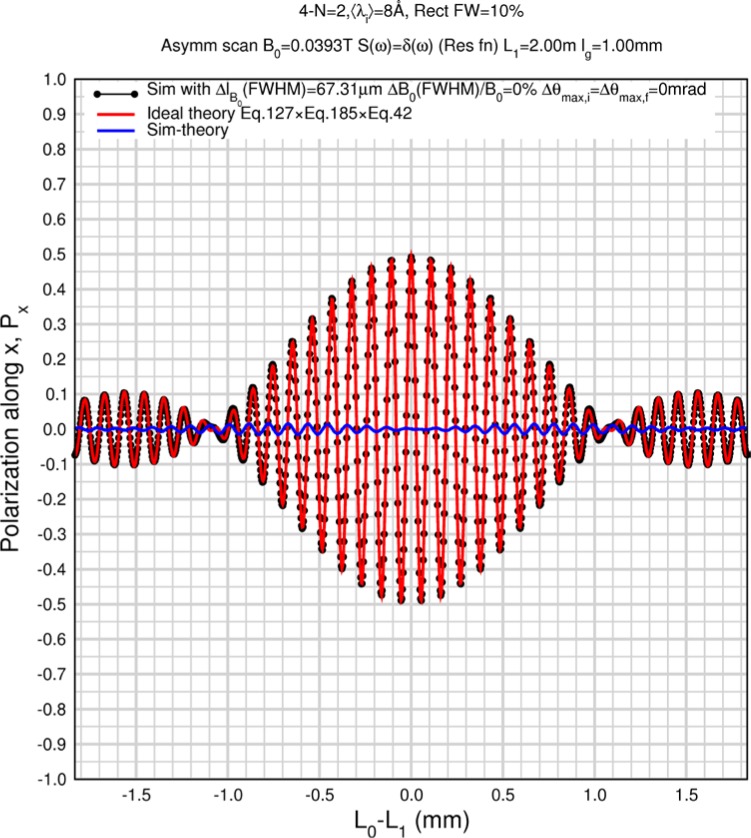
Simulated resolution echo at *B_0_* = 0.0393 T for the reference spectrometer configuration (4-*N*=2, *L_1_*= 2 m etc.) with a rectangular incident wavelength distribution of full width (FW) given by *Δλ_i_*/〈*λ_i_*〉 = 10 % and 〈*λ_i_*〉 = 8.0 Å. The simulation is performed with *ΔB_0_* = *Δθ_max_* = 0, but with *Δl_B0_* calculated according to Eq. (184) with *P_x_*^0^ = 0.5, such that the peak signal should be about 0.5 multiplied by the depolarization due to flipper coil dispersion. The red curve is the product of the theoretical “perfect instrument” resolution (Eq. (127)) multiplied by the estimated constant depolarization due to *Δl_B0_*, i.e., 0.5, (note: also obtained by substituting the chosen value of *Δl_B0_* (FWHM) back into Eq. (185)), multiplied by the estimated (constant) depolarization due to *Δλ* (Eq. (42)) with no fit parameters. The success of the analytical description of the simulation results in this example is revealed by the small residuals (blue curve). There is apparently no strong asymmetry-dependence of the effects of *Δl_B0_* in this case.

**Fig. 64:**
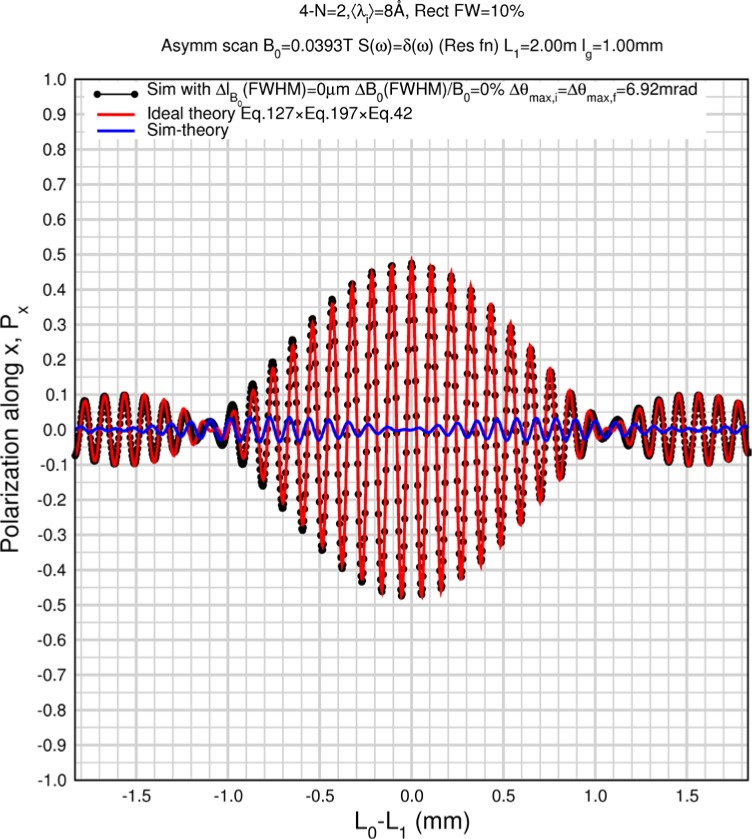
Simulated resolution echo at *B_0_* = 0.0393 T for the reference spectrometer configuration (4-*N*=2, *L_1_*= 2 m etc.) with a rectangular incident wavelength distribution of full width (FW) given by *Δλ_i_*/〈*λ_i_*〉 = 10 % and 〈*λ_i_*〉 = 8.0 Å. The simulation is performed with *ΔB_0_* =*Δl_B0_* = 0, but with *Δθ_i,max_* = *Δθ_f,max_* = *Δθ_max_* (simple divergence model) calculated according to Eq. (202) with *P_x_*^0^ = 0.5, such that the peak signal should be about 0.5 multiplied by the depolarization due to flipper coil dispersion. The red curve is the product of the theoretical “perfect instrument” resolution (Eq. (127)) multiplied by the estimated constant depolarization due to *Δθ_max_*, i.e., *approximately* 0.5, (“approximately” since Eq. (202) is only an approximate inversion of Eq. (197)), multiplied by the estimated (constant) depolarization due to *Δλ* (Eq. (42)) with no fit parameters. The success of the analytical description of the simulation results in this example is revealed by the relatively small residuals (blue curve). There is apparently no strong asymmetry-dependence of the effects of *Δθ_max_* in this case.

In the previous section it was shown that the depolarization due to flipper coil dispersion is roughly asymmetry-independent for moderate *Δλ_i_*/〈*λ_i_*〉. The resolution function for the case of a rectangular incident spectrum with *Δλ_i_*/〈*λ_i_*〉 = 10 % in an otherwise perfect spectrometer (one in which the only source of instrumental imperfection is flipper coil dispersion) has already been shown in Fig. 58 for the reference spectrometer configuration. Figure 62, Fig. 63, and Fig. 64 illustrate additionally the effects of applying, in turn, the effects of static field inhomogeneity, flipper coil length uncertainty, and beam divergence (simplified divergence model), respectively for the same basic spectrometer configuration. The magnitudes of *ΔB_0_*, *Δl_B0_*, and *Δθ_i,max_* = *Δθ_f,max_* = *Δθ_max_* are those obtained from Eqs. (176), (185), and (197) respectively that yield *P_x_*^0^ = 0.5 (in the absence of flipper coil dispersion). Instead of fitting the echo signal function, the simulations are compared directly (with no fit parameters) against the product of the theoretical “perfect (dispersionless) instrument” resolution function for the rectangular incident wavelength spectrum (Eq. (127)), the estimated dispersion depolarization (Eq. (42)), and the estimated effect of *ΔB_0_* (Eq. (176)), *Δl_B0_* (Eq. (185)), or *Δθ_max_* (Eq. (197)). The figures below demonstrate that the simulated resolution functions is predicted analytically to a good degree of accuracy for moderate beam monochromatization and that there are no strong asymmetry-dependent effects of *ΔB_0_*, *Δl_B0_*, or *Δθ_max_*.

## 9. Towards the Definition of the NIST NRSE Instrument

### 9.1 Possibilities Using MIEZE-II Configuration

#### 9.1.1 General Instrument Features

**Fig. 65:**
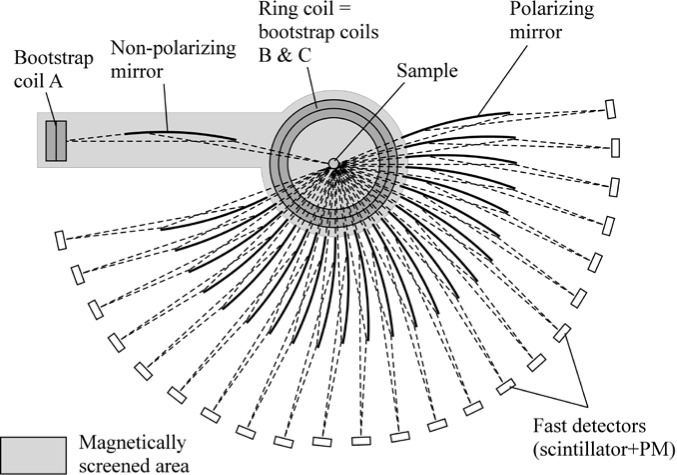
Schematic of a possible MIEZE-II NRSE instrument configuration (as proposed by Gähler). In the annular coil, the static field is perpendicular to the plane of the drawing and the r.f. field is in the plane of the drawing.

The general features of a so-called MIEZE-II spectrometer [29] are shown in Fig. 65. The configuration shown is equivalent to a multi-arm conventional arrangement of flipper coils, but with the fourth coil unit replaced by a “thin” detector at exactly the same location. The second and third bootstrap coils are created from the annular coil surrounding the sample area. In contrast to the similarly-named MIEZE spectrometer (see Ref. [16]), the r.f. frequency of all coil units is identical. The discussion in Sec. 4.2.1.3 illustrates the effect of eliminating the fourth coil: When the r.f. angular frequency is tuned to the Larmor frequency, *ω_0_*, the neutron spin-up and spin-down states retain their kinetic energy splitting after leaving the third coil unit, corresponding to a Larmor precession of angular frequency 2*ω_0_* (for *N* = 1 coils), or 4*ω_0_* (for *N* = 2 coils). Because the quasielastic echo point occurs at *L_0_* = *L_1_* for the 4 identical coil unit arrangement, the polarization at *L_1_* = *L_0_* (the detector plane in the MIEZE-II) is modulated at angular frequency 2*ω_0_* (*N* = 1), or 4*ω_0_* (*N* = 2), with maximum amplitude.

#### 9.1.2 Toroidal r.f. Solenoid

An annular *π*-flipper illustrated in Fig. 65 would be a new development for NRSE. The design of the r.f. coil depends on maintaining voltages within reasonable limits.

##### 9.1.2.1 Self-inductance of the toroidal r.f. solenoid

The self-inductance of a toroidal r.f. solenoid, radius *r_toroid_*, is approximately

(312) $$L=\frac{\mu_{0}N_{rf}^{2}A_{rf}}{l_{axial}^{rf}}=\frac{\mu_{0}N_{rf}^{2}A_{rf}}{2\pi r_{toroid}},$$

where *N_rf_* is the total number of turns, *l^rf^_axial_* is the axial length of the solenoid (in this case the mean circumference - *l^rf^_axial_* = 2*πr_toroid_*), and *A_rf_* is the cross-sectional area of the r.f. solenoid (the area enclosed by a single r.f. winding – i.e., the equivalent of *a_rf_* × *l_rf_* in Fig. 30). Substituting the winding density *n_rf_* (= *N_rf_*/*l^rf^_axial_*) into Eq. (312), we have

(313) $$\begin{array}{l} L_{toroid}[H]=2\pi r_{toroid}\mu_{0}n_{rf}^{2}A_{rf}=8\pi^{2}\times 10^{-7}{\left(n_{rf}\left[m^{-1}\right]\right)}^{2}r_{toroid}[m]A_{rf}[m^{2}]\\ =7.9\times 10^{-6}{\left(n_{rf}\left[m^{-1}\right]\right)}^{2}r_{toroid}[m]A_{rf}[m^{2}]. \end{array}$$

We now consider the dimensions of the toroidal coil. The tolerable uncertainty on *B_rf_* × *l_rf_* is somewhat relaxed for the r.f. coils when compared with the static field coil requirements at the highest values of *B_0_*, because only a *π* rotation of the spin is required around *B_rf_*. The flipping efficiency is naturally limited by coil dispersion so that relaxing the tolerance on *Δ*(*B_rf_ l_rf_*) is usually accompanied by a restriction of the bandwidth, *Δλ* (see Sec. 7.3.4.8). Nonetheless, it is likely that the r.f. coil height does not have to greatly exceed the beam height. For a toroidal coil, we substitute 2*πr_toroid_* for *l_axial_* in Eq. (266). If we impose a high voltage restriction, *V_rf_^pk^* ≤ 1500 V, we end up with

(314) $$\frac{n_{rf}\left[m^{-1}\right]r_{toroid}[m]a_{rf}[m]}{\langle \lambda_{i}\rangle \left[Å\right]}B_{0}[T]\leq 9.5\times 10^{-3}.$$

As an example, we assume that a coil height (side of the rectangle perpendicular to the beam direction) *a_rf_* ≈ 0.1 m provides sufficient r.f. field homogeneity within the beam area. The toroid radius, *r_toroid_*, must be sufficiently large to accommodate typical scattering sample environments. A reasonable value is *r_toroid_* ≈ 0.3 m. The choice of *r_toroid_* does not affect the instrumental resolution significantly, but it affects the usable solid angle. In order to estimate a worst case, we use the maximum value of *B_0_* and the minimum value of 〈*λ_i_*〉 from the previous discussions (about 0.04 T and 2 Å, respectively). It follows, from Eq. (314), that satisfying *n_rf_* (*B_0_* = 0.04 *T*, 〈*λ_i_*〉 = 2 Å, *r_toroid_* = 0.3 m, *a_rf_* = 0.1 m) ≤ 16 m^−1^ (1 turn every 6.3 cm) maintains the r.f. voltage below 1.5 kV in this case.

##### 9.1.2.2 Resistance and inductive reactance of the toroidal r.f. solenoid

The resistance of the r.f. coil winding is

(315) $$R_{toroid}=\rho (T)\frac{l_{w}^{rf}}{A_{w}^{rf}},$$

where *l_w_^rf^* is the total length of the winding and *A_w_^rf^* is the cross-sectional area of the r.f. wire, i.e.,

(316) $$l_{w}^{rf}\approx 4\pi r_{toroid}\;n_{rf}\left(a_{rf}+l_{rf}\right)$$

and

(317) $$A_{w}^{rf}=t_{rf}h_{rf},$$

so that Eq. (315) may be rewritten as

(318) $$R_{toroid}=\rho (T)\frac{4\pi r_{toroid}n_{rf}\left(a_{rf}+l_{rf}\right)}{t_{rf}h_{rf}}.$$

Using the above example with *l_rf_* = 0.025 m and *r_toroid_* = 0.3 m, the perimeter of one winding is 2(*a_rf_*+*l_rf_*) = 0.25 m and the total length of the winding, for *n_rf_* =16 m^−1^, is *l_w_^rf^* (*n_rf_* = 16 m^−1^, *a_rf_* = 0.1 m, *l_rf_* = 0.025 m) ≈ 25*r_toroid_* ≈ 7.5 m. Tightly-wound, rectangular cross-section wire has width perpendicular to the beam, *h_rf_* = *h_rf_^max^* ≈ 1/*n_rf_* ≈ 0.063 m. Choosing the winding thickness parallel to the beam direction, *t_rf_* = *t_max_* = 0.4 mm (as given in Sec. 7.3.2 for aluminum), the cross-sectional area is *A_w_^rf^* = 4 × 10^−4^/16 = 2.5 × 10^−5^ m^2^ (0.25 cm^2^). The minimum resistance of the winding is therefore

(319) $$R_{toroid}^{{}^{min}}[\Omega ]\approx 3\times 10^{5}\rho (T)[\Omega m].$$

Specifically for pure aluminum windings (using Eq. (234)), we have

(320) $$R_{Al}^{toroid}(T)[\Omega ]=\left(1.43\times 10^{-9}T[K]-8.7\times 10^{-8}\right)\frac{r_{toroid}[m]n_{rf}\left[m^{-1}\right](a_{rf}+l_{rf})[m]}{t_{rf}[m]h_{rf}[m]},$$

and for this specific geometry

(321) $$R_{toroid}^{min}\{\text{Al}\}(T)[\Omega ]\approx 3.44\times 10^{-5}T(K)-2.08\times 10^{-3}.$$

Around room temperature, *ρ_Al_* ≈ 2.73 × 10^−8^ Ωm and the resistance of the winding is approximately *R_toroid_^min^*{Al}(*T* = 300 K, *r_toroid_* = 0.3 m, *t_rf_* = 4 × 10^−4^ m, *n_rf_* =16 m^−1^) ≈ 8 mΩ.

The inductive reactance of the r.f. coil is

(322) $$X_{L}[\Omega ]=\omega_{rf}\left[s^{-1}\right]L[H].$$

On resonance, with *B_0_* = 0.04 T, we have *ω_rf_* ≈ 7.3×10^6^ rad s^−1^ (*ν_rf_* = 1.17 MHz) (Eq. (9)). For *n_rf_* = 16 m^−1^, *r_toroid_* = 0.3 m, and choosing a typical *A_rf_* = 0.1 × 0.025 = 2.5 × 10^−3^ m^2^, we have (from Eq. (313)) *L* ≈ 1.5 *µ*H, from which we obtain *X_L_* ≈ 11 Ω. Therefore, *X_L_* ≫ *R_toroid_* at the highest frequencies (in this example, by more than three orders of magnitude).

##### 9.1.2.3 Current and power dissipated in r.f. coil

With a maximum required r.f. field magnitude of about 2.7 mT (see Sec. 7.3.4.3) and a winding density *n_rf_* = *n_rf_*(*r_toroid_* = 0.3 m) = 16 m^−1^, Eq. (213) implies that a peak current *I_pk_* ≈ 2.7 × 10^−3^/(1.26 × 10^−6^ × 16) = 134 A is required. Therefore, having eliminated the high voltage problem we appear to run into problems with peak current. This is mitigated by increasing the minimum operational wavelength. Nonetheless, because the r.f. coil load is almost entirely inductive at high frequencies (see previous example), the high frequency current in the coil lags the voltage by approximately 90°. The heat dissipated in the r.f. coil is only that due to the resistance. In the example given in Sec. 9.1.2.2, for room temperature aluminum windings of enclosed area 0.25 cm^2^, *R_toroid_* ≈ 8 mΩ, therefore the maximum rms power dissipated in the coil is *P_RMS_^rf^*{Al}(*T* = 300 K, *r_toroid_* = 0.3 m, *n_rf_* = 16 m^−1^, *A_w_^rf^* = 2.5 × 10^−5^ m^2^) ≈ (134/√2)2 × 8 × 10^−3^ ≈ 72 W. This is not excessive.

#### 9.1.3 Requirements for the MIEZE Detection System

As demonstrated in Sec. 6.6, accessing high-resolution requires flight path length uncertainties in the several tens of microns range, with frequencies in the 1 MHz range. Therefore, the active part of the detector must be flat and capture neutrons within tens of micron thicknesses with reasonable efficiency. The most suitable detector type appears to be a scintillator-photomultiplier combination. The charged particles for activation of the scintillation originate from a nuclear reaction produced by the absorption of thermal neutrons. The very small absorption depth probably requires a ^6^Li-containing compound such as ^6^LiF, which produces negligible gamma radiation. Scintillator material such as ZnS:Ag, ZnS:Cu,Al,Au have the advantage of rapid decay times (no afterglow). The data acquisition response time should be preferably within the 1 ns to 10 ns range with signal handling up to about 4 MHz, if *N* = 2 bootstrap coils are used. (Note that the signal frequency is 4*ω_0_* for *N* = 2 − see Sec. 9.1.1).

### 9.2 Criteria for Permanent Magnet NRSE Options

**Fig. 66:**
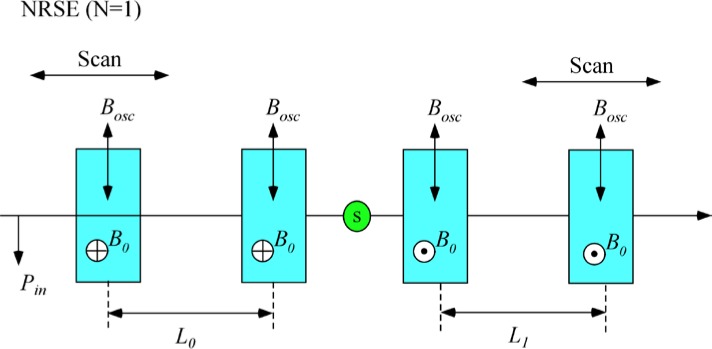
Schematic of a quasi-elastic NRSE instrument using electrically-insulating permanent magnets to provide the static field with a superimposed r.f. field.

An important limitation on the static field coil is the restriction on the winding thickness parallel to the beam, imposed by neutron absorption and scattering. In Sec. 7.3.3.1 we saw that this may lead to significant heat generation at high fields, unless the coils are cryogenically cooled. We now consider the feasibility of replacing the static field coils by a ferromagnetic or anti-ferromagnetic material that transmits neutrons. The static field magnitude is fixed in this scenario, therefore, a scan of *τNRSE* might involve a scan of the r.f. unit separation in each arm of the spectrometer, such that that *δ*(*BL*) is maintained at zero (the echo point). This contrasts with varying the static field magnitude at fixed *L* in the coil case. A quasi-elastic NRSE spectrometer using permanent magnets to provide the static field (*N* = 1) is shown in schematically in Fig. 66.

#### 9.2.1 Comparison of Static Field Coil and Permanent Magnet NRSE

**(a) Coil**

- *τ_NRSE_* scan usually fixes *L_0_*, *L_1_* and varies *B_0_*.
- Requires anodized aluminum windings.
- High resolution applications typically require very flat and parallel windings.
- Production of (0.03 to 0.04) Tesla fields with thin (in the beam direction) Al windings is challenging. Heat dissipation is proportional to the coil surface area and can reach several kW with typical windings, unless cryogenically cooled.
- Maintaining adequate coil cooling without interfering with the beam path is challenging.

**(b) Permanent magnet**

- *τ_NRSE_* scan has fixed *B_0_*, *B_1_*, vary *both L_0_* and *L_1_*.
- High resolution applications require similar dimensional tolerances to the coils (but probably easier to achieve).
- Field homogeneity is very good inside the magnet and field boundaries are abrupt.
- Requires a neutron-transparent magnetic material.
- Magnet must reside *inside* the r.f. coil – requires magnetic material to be electrically-insulating.
- Magnet likely requires an externally-applied saturation field.
- Fixed r.f. frequency – (no r.f. circuit tuning, fixed impedance).
- Resonance width requires field magnitudes in each unit to be similar to within a few tens of *µ*T.
- Significantly reduced heat removal problem.
- Compact, with no electrical coil circuitry.

#### 9.2.2 Definition of the Required Instrument Parameters Using Permanent Magnets

In the following, we develop a set of inequalities defining the major parameters required to achieve the desired spectrometer performance using permanent magnets.

##### 9.2.2.1 Coil unit geometry

**Fig. 67:**
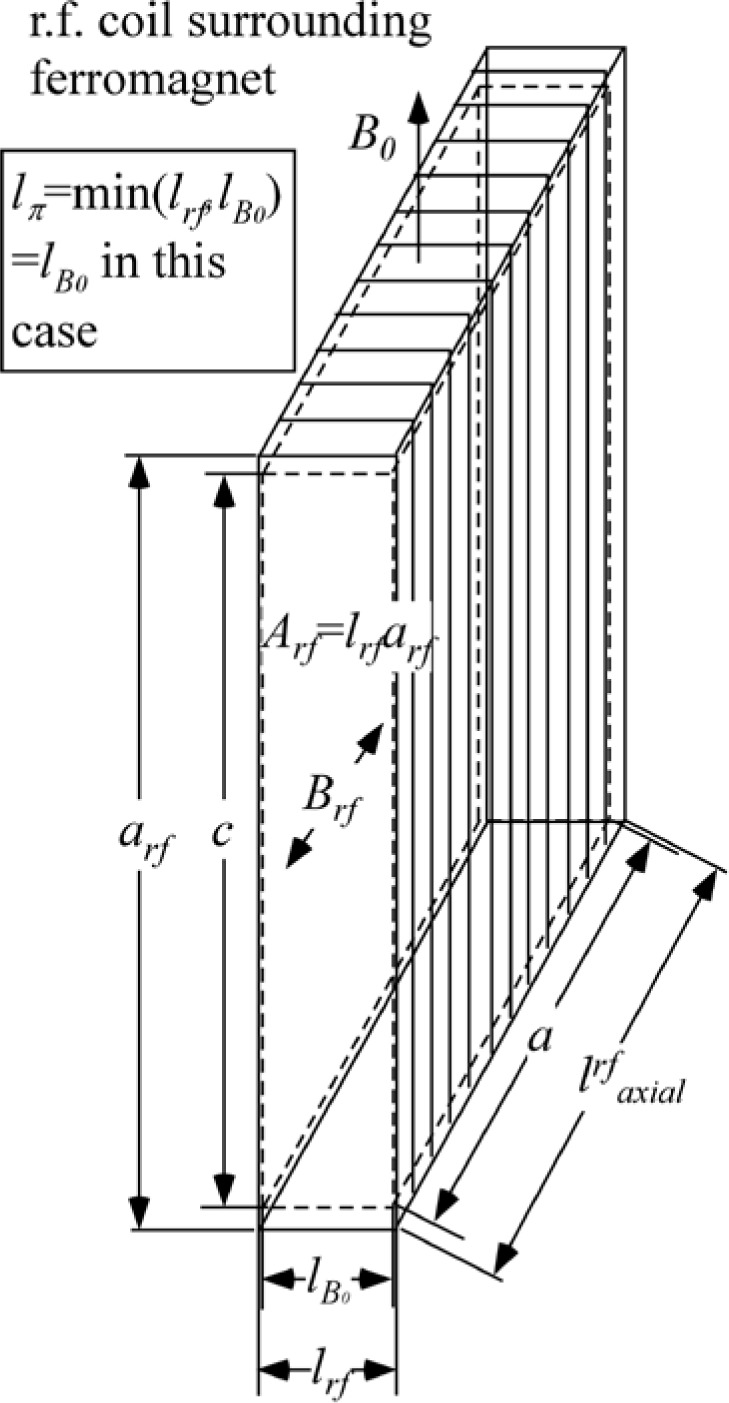
Schematic of a flipper coil unit using a permanent magnetic material to provide the static field.

The coil unit consists of a permanent magnetic material enclosed by an r.f. coil (with a perpendicular field axis) as shown in Fig. 67. Henceforth, we refer to the dimensions defined in Fig. 67.

##### 9.2.2.2 *B_0_L_0_* magnitude criterion for accessing *τ_NRSE_* = 30 ns at *λ* = 8 Å

The criterion for accessing *τ_NRSE_* = 30 ns at *λ* = 8 Å is expressed by Eq. (206). Assuming that there is a practical upper limit on *L_0_* imposed by spatial constraints (represented by *L_0_^max^*), we define a minimum *required* value of *B_0_* criterion according to:

(323) $$B_{0}^{\max}[T]\geq \frac{0.157}{NL_{0}^{\max}[m]}\;\text{minimum}\;B_{0}\;\text{magnitude to reach}\;\tau_{NRSE}=30\;\text{ns at}\;\lambda =8Å.$$

##### 9.2.2.3 Minimum wavelength (*λ* = 2 Å) r.f. voltage criterion

An approximate expression for the peak r.f. voltage in terms of the r.f. coil parameters and the neutron wavelength is given by Eq. (266), where *a_rf_* and *l^rf^_axial_* are shown in Fig. 67:

(324) $$V_{rf}^{pk}[V]\approx 2.5\times 10^{4}\frac{n_{rf}\left[m^{-1}\right]l_{axial}^{rf}[m]a_{rf}[m]}{\langle \lambda_{i}\rangle \left[Å\right]}B_{0}[T].$$

We know that *a_rf_* cannot be smaller than the beam height, say *a_rf_^min^* ≈ 0.03 m. For compatibility with typical high voltage cable ratings (see Sec. 7.3.4.3), we have

(325) $$V_{rf}^{\max}<1500V,$$

which, combined with Eq. (324), equates to

(326) $$\frac{n_{rf}[m^{-1}]l_{axial}^{rf}[m]a_{rf}[m]}{\langle \lambda_{i}\rangle \left[Å\right]}B_{0}^{\max}[T]\leq 0.06,$$

as it was in Sec. 7.4. This is most demanding at the minimum operating wavelength, which we assume to be *λ* = 2 Å, therefore

(327) $$n_{rf}\left[m^{-1}\right]l_{axial}^{rf}[m]a_{rf}[m]B_{0}^{\max}[T]\leq 0.12.$$

We remember that *B_0_* is also subject to the constraint expressed in Eq. (323), therefore we require

(328) $$L_{0}^{\max}[m]\geq 1.3\frac{n_{rf}[m^{-1}]l_{axial}^{rf}[m]a_{rf}[m]}{N}\;\text{maximum r}.f.\;\text{voltage at min}\;\lambda (=2\;Å)\;\text{criterion}.$$

##### 9.2.2.4 Required Resolution: Tolerance criteria for *B_0_*, *l_B0_*, and beam divergence

If the elastic signal magnitude must equal or exceed *P_x_*^0^ at *τ_NRSE_*(*λ* = 8 Å) = 30 ns, this imposes maximum tolerances on the values of *B_0_*, *l_B0_*, and the beam divergence, *Δθ* which are expressed in Eqs. (203–205) (for equal contributions). We assume that the field variation inside the permanent magnet, *ΔB_mat_*, is a fixed property of the material and that the minimum dimensional uncertainty in the beam direction is *Δl_mat_*. If we choose *P_x_^0^*(*τ_NRSE_* = 30 ns, *λ* = 8 Å) ≥ 0.5, Eq. (203) can be rewritten in terms of a condition on the variable *l_B0_*, i.e.,

(329) $$l_{B_{0}}[m]\leq \frac{2.2\times 10^{-6}}{\sqrt{N}\Delta B_{mat}[T]}\;\text{originating from}\;\Delta B_{0}\;\text{criterion},$$

where we have set 〈*λ*〉 = 8 Å. Note that this condition is not especially demanding for any realistically attainable *ΔB_mat_*. Likewise, for *P_x_^0^*(*τ_NRSE_* = 30 ns, *λ* = 8 Å) ≥ 0.5, Eq. (204) imposes a condition on the magnitude of *B_0_*, i.e.,

(330) $$B_{0}[T]\leq \frac{2.2\times 10^{-6}}{\sqrt{N}\Delta l_{mat}[m]},$$

subject to the minimum required *B_0_* condition (Eq. (323)), from which we have:

(331) $$L_{0}^{\max}[m]\geq 7.1\times 10^{4}\frac{\Delta l_{mat}[m]}{\sqrt{N}}\;\text{from}\;\Delta l_{B0}\;\text{criterion and minimum required}\;B_{0}\;\text{criterion}.$$

Applying the *P_x_^0^*(*τ_NRSE_* = 30 ns, *λ* = 8 Å) ≥ 0.5 to Eq. (205), we have

(332) $${\left(\Delta \theta_{\max}\left[\text{rad}\right]\right)}^{2}\left(\lambda =8Å\right)\leq \frac{4.3\times 10^{-6}}{NB_{0}[T]L_{0}[m]},$$

subject to the condition (323), which leads to

(333) $$\Delta \theta_{\max}\left(\lambda =8Å\right)\leq 5.2\times 10^{-3}\;\text{rad maximum divergence criterion for}\;P_{x}{}^{0}\left(\tau_{NRSE}=30\;\text{ns},\;\lambda =8\;Å\right),$$

(For reference, this is about 37.5 % of the critical angle of natural Ni at *λ* = 8Å).

##### 9.2.2.5 Tolerance criterion for *B_rf_* – r.f. penetration of the permanent magnet and absorbed r.f. power

Variations of the magnitude of *B_rf_* within the static field region lead to reduced flipping efficiencies and, consequently, reduced signal magnitudes. One source of attenuation of *B_rf_* is absorption of the r.f. field by the magnetic material with the associated heating. In the medium-wave (MW) to short-wave (SW) band that is relevant to the NRSE (far from molecular vibrations that reside in the > 100 GHz microwave range), the average magnitude of the Poynting vector (which, for a plane wave, is the energy density × the phase velocity) in a material of conductivity, *σ*, permeability, *µ*, and permittivity, *ε*, may be expressed as

(334) $$\begin{array}{l} S_{av}\frac{H_{rf}^{2}}{4}\left(\frac{\omega \varepsilon +\sqrt{\omega^{2}\varepsilon^{2}+\sigma^{2}}}{\sqrt{\omega^{2}\varepsilon^{2}+\sigma^{2}}}\right){\left[\frac{2\omega \mu }{\left(\sqrt{\omega^{2}\varepsilon^{2}+\sigma^{2}}+\omega \varepsilon \right)}\right]}^{1/2}\exp\left(-{\left[2\omega \mu \left(\sqrt{\omega^{2}\varepsilon^{2}+\sigma^{2}}-\omega \varepsilon \right)\right]}^{1/2}y\right)\\ =\frac{H_{rf}^{2}}{4}\left(\frac{\omega \varepsilon +\sqrt{\omega^{2}\varepsilon^{2}+\sigma^{2}}}{\sqrt{\omega^{2}\varepsilon^{2}+\sigma^{2}}}\right){\left[\frac{2\omega \mu }{\left(\sqrt{\omega^{2}\varepsilon^{2}+\sigma^{2}}+\omega \varepsilon \right)}\right]}^{1/2}\exp\left(-2\frac{y}{\delta }\right), \end{array}$$

where *δ* is the attenuation length and *ω* is the angular frequency of the electromagnetic radiation. There are two obvious limiting cases:

- Perfect insulator: *σ* → 0 and *δ* → ∞ (no electromagnetic radiation is absorbed).
- Good conductor: *σ* ≫ *ωε* and *δ* → (2/*µσω*)^1/2^ (otherwise called the skin depth [see Eq. (274)]). For the good conductor,

(335) $$S_{av}=H_{rf}^{2}{\left[\frac{\mu \omega }{8\sigma }\right]}^{1/2}\exp\left(-2\frac{y}{\delta }\right).$$

**Fig. 68:**
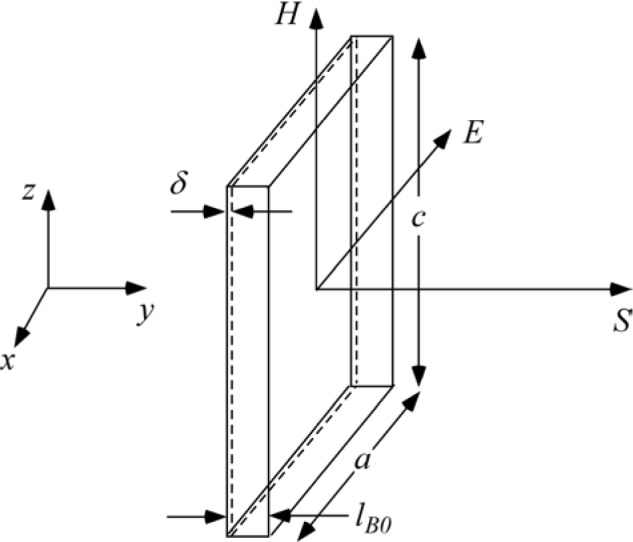
With the r.f. *H*-field oriented as shown in a good conductor, most of the r.f. power is absorbed perpendicular to the plane containing *E* and *H* (in the direction of the Poynting vector, *S*) within the skin depth, *δ*, if *l_B0_* > *δ*.

For an applied r.f. *H*-field as shown in Fig. 68, with a slab thickness *l_B0_* ≫ *δ* in the direction of *S_av_*, and slab area *ac*, the Poynting vector is interpreted as follows:

(336) $$S_{av}=\frac{energy}{vol}.phase\;velocity=\frac{energy}{ac\;dy}.\frac{dy}{dt}=\frac{power}{ac},$$

which is the power per unit slab area, *ac*. From Eq. (335) we note that almost 90 % of the r.f. energy is absorbed in the initial thickness *δ*, as indicated in Fig. 68. In fact, after a thickness *l_B0_*, the power absorbed per unit slab area is

(337) $$\Delta S_{av}=H_{rf}^{2}{\left[\frac{\mu \omega }{8\sigma }\right]}^{1/2}\left[1-\exp\left(-2\frac{l_{B_{0}}}{\delta }\right)\right]\approx H_{rf}^{2}{\left[\frac{\mu \omega }{8\sigma }\right]}^{1/2}\;\text{for}\;l_{B_{0}}\gg \delta .$$

The power absorbed per unit area increases as the square root of the frequency.

The required electromagnetic properties of the magnetic material are estimated by assuming that the r.f. field attenuation corresponds to a value *ΔB_rf_* which produces a precession angle *π*−*β* around ***B****_rf_* (see Sec. 2.2), in which *β* must not exceed ± 2.5°. This ensures that *ΔB_rf_* does not significantly compromise the usable bandwidth *Δλ*/*λ*, (± 2.5° corresponds to the equivalent effect produced by *Δλ*/*λ* ≈ 3 % FWHM). Thus, by setting

(338) $$\frac{\Delta B_{rf}}{B_{rf}}\leq 0.03,$$

we require that the exponential in Eq. (334) is greater than (1−0.03) = 0.97 over the thickness of the crystal, *l_B0_*, i.e.,

(339) $$\exp\left(-2\frac{l_{B_{0}}}{\delta }\right)\geq 0.97,$$

Which, in turn, implies

(340) $$\delta \geq 65.7l_{B_{0}}=65.7l_{\pi }\approx 65.7l_{rf},$$

where

(341) $$\delta ={\left(\frac{2}{\omega_{rf}\mu \left(\sqrt{\omega_{rf}^{2}\varepsilon^{2}+\sigma^{2}}-\omega_{rf}\varepsilon \right)}\right)}^{1/2}.$$

Combining Eqs. (340) and (341) with the resonance tuning condition for the r.f. frequency (Eq. (9)), we obtain:

(342) $$l_{B_{0}}[m]=l_{\pi }[m]\leq \frac{1.59\times 10^{-6}}{{\left[B_{0}[T]\mu \left[{\text{NA}}^{-2}\right]\left(\sqrt{{\left(1.83\times 10^{8}B_{0}[T]\right)}^{2}\varepsilon {\left[{\text{Fm}}^{-1}\right]}^{2}+\sigma {\left[m^{-1}\Omega^{-1}\right]}^{2}}-1.83\times 10^{8}B_{0}[T]\varepsilon \left[{\text{Fm}}^{-1}\right]\right)\right]}^{1/2}},$$

subject to the minimum *B_0_* criterion (Eq. (323)). If the criterion in Eq. (342) is satisfied, the r.f. power absorption is almost uniformly distributed over the slab thickness and the power absorbed in the slab volume is

(343) $$P_{rf}^{abs}\leq 0.03\;ac\frac{H_{pk}^{2}}{4}\left(\frac{\omega \varepsilon +\sqrt{\omega^{2}\varepsilon^{2}+\sigma^{2}}}{\sqrt{\omega^{2}\varepsilon^{2}+\sigma^{2}}}\right){\left[\frac{2\omega \mu }{\left(\sqrt{\omega^{2}\varepsilon^{2}+\sigma^{2}}+\omega \varepsilon \right)}\right]}^{\frac{1}{2}}.$$

The remaining issues are those of providing adequate cooling to the magnetic material.

##### 9.2.2.6 Examples

**Some simplifications**

The instrument parameter envelope is defined by the solution of Eqs. (323), (328), (329), (331), (333), and (342), whilst ensuring that the r.f. power absorption (Eq. (343)) and neutron absorption/scattering remain under control. In these examples, we make the following simplifications:

- We fix *N* = 2 − the preferred bootstrap factor for the spectrometer (see Sec. 3.4.2).
- Because there are two conditions involving *L_0_^max^*, one concerning materials length tolerances (Eq. (331)) and the other involving parameters influencing the r.f. coil inductance (Eq. (328)), we impose limits on *L_0_^max^* using Eq. (331), then examine the consequences for the r.f. coil parameters in Eq. (328) − specifically the coil winding density, given limitations on the r.f. coil dimensions.
- The permanent magnet material is one that satisfies Eqs. (342) and (323) simultaneously, has low r.f. power absorption (Eq. (343)), and is transparent to neutrons. The conditions associated with Eqs. (342) and (343) amount to finding a sufficiently electrically-insulating material. Satisfying Eq. (323) requires the material to have a suitably large *B* field at saturation.
- Satisfying Eq. (333) for high-resolution operation likely requires placement of neutron optical elements that reduce flight path length differences between coil units to a factor not grossly exceeding *Δθ_max_*^2^/2.

The above simplifications lead to the following set of conditions that must be satisfied simultaneously:

(344) $$l_{B_{0}}[m]\leq \frac{1.56\times 10^{-6}}{\Delta B_{mat}[T]}\;(\text{from Eq}.\;(329)).$$

(345) $$L_{0}^{\max}[m]\geq 5.0\times 10^{4}\Delta l_{mat}[m]\;(\text{from Eq}.\;(331)).$$

Combining Eqs. (345) and (328), we have

(346) $$n_{rf}\left[m^{-1}\right]l_{axial}^{rf}[m]a_{rf}[m]\leq 7.7\times 10^{4}\Delta l_{mat}[m].$$

(347) $$\Delta \theta_{\max}\left(\lambda =8Å\right)\leq 5.2\times 10^{-3}\;\text{rad}\;(\text{from Eq}.\;(333)).$$

(348) $$l_{B_{0}}\approx l_{\mu }[m]\leq \frac{1.59\times 10^{-6}}{{\left[B_{0}[T]\mu \left[{\text{NA}}^{-2}\right]\left(\sqrt{{\left(1.83\times 10^{8}B_{0}[T]\right)}^{2}\varepsilon {\left[{\text{Fm}}^{-1}\right]}^{2}+\sigma {\left[m^{-1}\Omega^{-1}\right]}^{2}}-1.83\times 10^{8}B_{0}[T]\varepsilon \left[{\text{Fm}}^{-1}\right]\right)\right]}^{\frac{1}{2}}}\;(\text{from Eq}.(342)),$$

subject to

(349) $$B_{0}^{\max}[T]\geq \frac{7.85\times 10^{-2}}{L_{0}^{\max}[m]}\;(\text{from Eq}.\;(323)).$$

**Example with *N* = 2, 0.01º slab parallelism, *a* = *c* = 0.1 m, and *ΔB****_mat_* **= 50 *μ*T**

The slab parallelism of 0.01° is based on what is considered reasonably achievable. Over a slab dimension of 0.1 m, this corresponds to *Δl_mat_* ≈ 20 *µ*m. Using these values in Eqs. (344–349), we obtain:

$$l_{\pi }[m]\leq \frac{1.56\times 10^{-6}}{\Delta B_{mat}[T]}\Rightarrow l_{\pi }\leq 0.031\;m.$$

$$L_{0}^{\max}[m]\geq 5.0\times 10^{4}\Delta l_{mat}[m]\Rightarrow L_{0}^{\max}\geq 1\;m.$$

For this range of *L_0_^max^* we have, from Eq. (349):

$$B_{0}^{\max}[T]\geq \frac{7.85\times 10^{-2}}{L_{0}^{\max}[m]}\Rightarrow \{{}_{B_{0}\left(L_{0}^{\max}=2\;m\right)\geq 0.0393\;T}^{B_{0}\left(L_{0}^{\max}=1\;m\right)\geq 0.0785\;T}$$

and, from Eq. (346):

$$n_{rf}\left[m^{-1}\right]l_{axial}^{rf}[m]a_{rf}[m]\leq 1.54.$$

Assuming *a_rf_* ≈ *c* = 0.1 m and *l^rf^_axial_* ≈ *a* = 0.1 m (see Fig. 67), this condition is expressed in terms of a maximum winding density of the r.f. coil by

$$n_{rf}^{\max}\left[m^{-1}\right]\leq 154\;m^{-1}.$$

Using the above range of *l_B0_* (*l_π_*), Eq. (348) becomes:

$$\begin{array}{l} B_{0}[T]\mu \left[{\text{NA}}^{-2}\right]\left(\sqrt{{\left(1.83\times 10^{8}B_{0}[T]\right)}^{2}\varepsilon {\left[{\text{Fm}}^{-1}\right]}^{2}\sigma {\left[m^{-1}\Omega^{-1}\right]}^{2}}-1.83\times 10^{8}B_{0}[T]\varepsilon [{\text{Fm}}^{-1}]\right)\leq 2.6\times 10^{-9}\\ \text{subject to}\;B_{0}^{\max}[T]\geq \frac{7.85\times 10^{-2}}{L_{0}^{\max}[m]}\;(\text{Eq}.\;(349)). \end{array}$$

As an example, room temperature FeO has *µ* =(1 + *χ_M_*)*µ_0_* = 1.27 × 10^−6^ NA^−2^ and *ε_r_* = 14.2 (*ε* = 1.26 × 10^−10^ Fm^−1^). The conductivity of FeO [30] is *σ* ≈ 2000 m^−1^Ω^−1^. In this case, the left hand side of the inequality is approximately *B_0_* [T] *µ* [NA^−2^] *σ*[m^−1^ Ω^−1^] ≈ 10^−4^, making it impossible to satisfy. However, at liquid nitrogen temperature the band conduction is such that *σ* drops below about 0.3 m^−1^Ω^−1^ (see also Ref. [30]). The left hand side in this case is about 1.5 × 10^−8^, which is much closer to the requirement. Alternatively, the *ΔB_rf_*/*B_rf_* condition must be relaxed. Nonetheless, the materials issues for a permanent magnet option appear to be the principal challenge, especially in view of the neutron transmission constraints.

#### 9.2.3 Potential Problems with the Multi-Angle Permanent Magnet Configuration

**Fig. 69:**
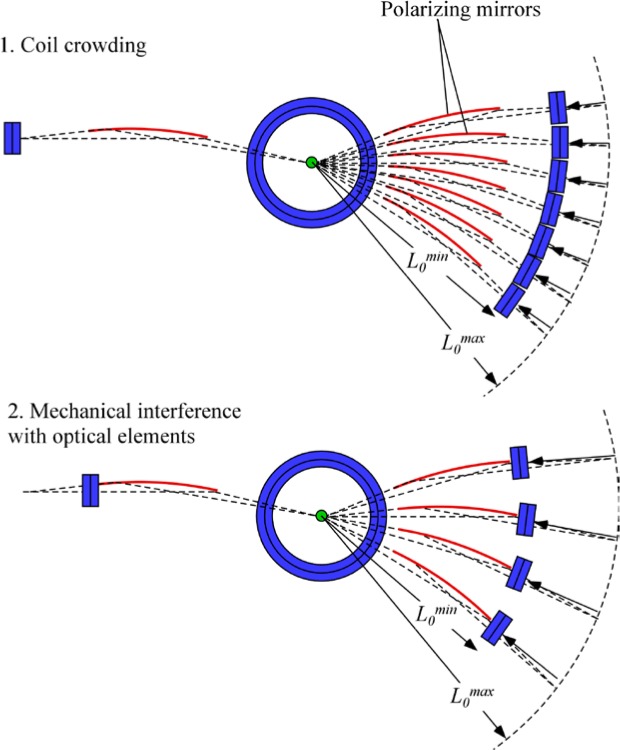
Possible mechanical interferences when using permanent magnets in a multi-angle NRSE arrangement.

In a permanent magnet, multi-angle NRSE arrangement, a potential geometrical issue is either that of crowding of the fourth flipper coil units (or detectors in a MIEZE-II configuration) as *τ_NRSE_* (and hence *L_0_*) is reduced, or that of mechanical interference of the coil units with the high-resolution optical elements (Fig. 69). Both problems reduce the Fourier time range. If corrective mirrors are installed, they are optimized for the highest resolution (i.e., for *L_0_* = *L_0_^max^*) and may not be required when measuring shorter Fourier times. An NSE mode of operation could also take over at short Fourier times. Because the magnetic field magnitude is fixed, one cannot adopt the method described in Sec. 7.3.6.2 for the NSE mode. A possible solution consists of rotating the magnets to change the field integral (see following section).

### 9.3 An NSE Permanent Magnet Configuration?

**Fig. 70:**
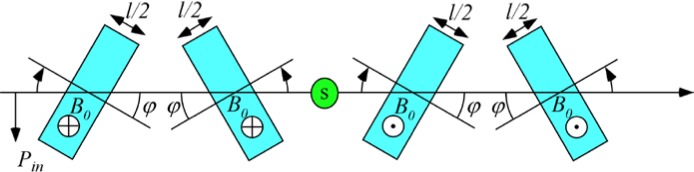
A possible NSE configuration for quasielastic scattering using permanent magnets. The total field integral in each arm = *B_0_l*/cos*φ*. The symmetrically opposing tilt scheme allows the net locus of constant *φ_NSE_* to remain parallel to the *Q*-axis in *Q*-*ω* space.

One may compare the Fourier time ranges of a NRSE spectrometer with that of the NSE configuration using permanent magnets. As there is no oscillating field in the NSE, the precession field integral is varied by changing the magnet tilt, as opposed to the magnet separation. However, the spin-up and spin-down neutron *k*-vector components that are *normal* to the field boundaries are split in magnitude inside the field, whereas the parallel components are not. The result is that the locus of constant spin-echo phase is *Q*-dependent. Whilst this property is exploited for measuring widths of dispersive excitations [29], it is problematic for quasielastic scattering, where a given spin-echo phase is obtained for a range of energy transfer-*Q* magnitude combinations allowed by the broad incident wavelength band and the beam divergence. A possible mitigating solution uses opposing symmetric tilts (as opposed to tilts in the same sense), as shown schematically in Fig. 70. The upper Fourier time limits for the NSE and NRSE configurations are obtained by comparing the effective field integrals 2*NB_0_^NRSE^L_0_* for the NRSE with *B_0_^NSE^ l*/cos *φ_max_* for the NSE, where *φ_max_* is the maximum tilt angle of the magnet. For an *NRSE* instrument capable of reaching *τ_NRSE_* = 30 ns at *λ* = 8 Å, we have

(350) $$2NB_{0}^{NRSE}L_{0}\approx 0.32\;\text{Tm}.$$

Ferromagnetic materials might have *B_0_* in the range (1 to 2) T. Therefore, reaching a field integral of 0.32 Tm with the NSE configuration requires

(351) $$\frac{l}{\cos\;\varphi_{\max}}\approx (0.32\;\text{to}\;0.16)m.$$

For any *φ_max_* this is unacceptably thick for thermal neutron transmission, therefore a permanent magnet NSE configuration is feasible only for measuring the lower Fourier time range (as was the case with the coils – see e.g. Fig. 33).

### 9.4 Neutron Guide Requirements

**Fig. 71:**
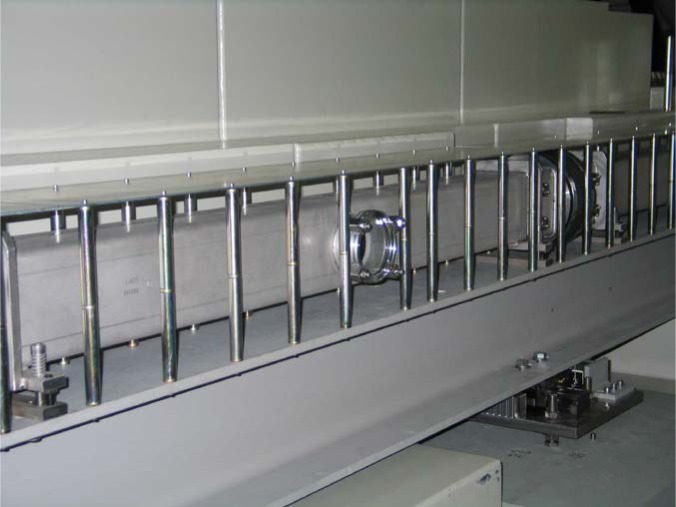
A section of the polarizing neutron guide feeding the 3-axis NRSE at the FRM-II. The vertical bars are permanent magnets providing a guide-field. The neutron guide is contained inside a vacuum casing in this example (photo kindly allowed by T. Keller, FRM-II).

The static field homogeneity and corrective optics requirements demand a small area, low divergence, cold neutron beam. This may be provided by a curved or curved-straight polarizing neutron guide or a conventional neutron guide followed by a polarizer. If the beam monochromatization is provided by a velocity selector, the polarizing elements are placed downstream. A polarizing neutron guide at FRM-II is shown in Fig. 71.

A curved-straight neutron guide arrangement, designed according to the prescription for “Phase-Space Tailoring” (PST) [31,32], is particularly suitable for this application. Despite the curved section, a PST guide is capable of delivering a beam with optimal intensity and uniform spatial and angular distributions, for all wavelengths exceeding a threshold, *λ′*, determined by the guide geometry and reflective coatings. From the estimates given in Table 7, it is likely that the incident beam divergence tolerances are stringent for high-resolution operation. Even if beam divergence dominates the instrumental depolarization, the tolerances in Table 7 are relaxed by only a factor of √3. This requires a critical angle of reflection of about 50 % of natural Ni – about that of polished glass – to obtain *P_x_^0^* = 0.5 at (*τ_NRSE_* = 30 ns, *λ* = 8 Å) in this example. This degree of beam collimation is not required for lower resolution measurements, therefore a likely design would introduce additional collimation, as necessary, into a more divergent beam.

To illustrate the implications of the high-resolution limits for a PST guide design, we choose a beam size *W* × *H* = 3 cm × 3 cm with a total length of the curved-straight guide combination, *L_tot_* = *L_c_* + *L_str_* = 50 m, where *L_c_* is the length of the curved section and *L_str_* is the length of the straight section. The short neutron wavelength filtering ability of the curved section (assuming no direct line of sight) is expressed in terms of the “characteristic wavelength”, *λ_c_*. In the small angle approximation, *λ_c_* is given by

(352) $$\lambda_{c}\approx \sqrt{\frac{2W}{\rho }}\frac{1}{\gamma_{Ni}m_{out}},$$

where *W* is the guide width, *ρ* is the radius of curvature, *m_out_* is the factor by which the critical angle of the reflective coating on the outer radius of the curved section exceeds that of natural Ni, and *γ_Ni_* is the critical angle per unit wavelength of natural Ni (*γ_Ni_* ≈ 1.73 × 10^−3^ rad Å^−1^). For an ideal (perfect reflectivity and circular curvature), long (no line-of-sight) curved guide, *λ_c_* defines:

- The wavelength below which the transmitted beam consists *only* of neutrons that have had no contact with the inner radius.
- The wavelength at which the 2-D transmission is 2/3 that of the ideal long straight guide with side coatings *m_out_*.
- The wavelength below which the transmitted 2-D phase space area decreases ∝ *λ*^3^ (c.f. ∝ *λ* for the 2-D straight guide).

A compromise between good epithermal neutron suppression and reasonable transmission at *λ_min_* = 2 Å is achieved by setting *λ_c_* ≈ 4 Å. If the required conditions for PST operation are met (see Ref. [32]), *λ′* is given by

(353) $$\lambda^{\prime }=\frac{m_{out}}{\sqrt{\left(m_{out}^{2}-m_{in}^{2}\right)}}\lambda_{c},$$

where *m_in_* characterizes the inner radius critical angle for the curved section. Note that *λ*′ exists within the range between *λ_c_*(when *m_out_* ≫ *m_in_*) and ∞ (when *m_in_* ≥ *m_out_*). Consequently, ideal PST conditions are not obtainable for *λ* < *λ_c_*, however, curved-straight configurations can significantly reduce spatial-angular asymmetries introduced by the curved section [33]. A curved guide is considered “long” if it has no direct line-of-sight [LOS]. For this to be true, *L_c_* must satisfy:

(354) $$L_{c}\geq L_{LOS}\approx \sqrt{8W\rho }.$$

Particularly favorable gamma-ray filtering occurs when *L_c_* ≥ 2*L_LOS_*, since neither the direct nor the once-scattered gamma rays from the source are viewable from the guide exit. Thus, in the following examples we set a desirable (but not necessary) constraint *L_c_* = 2*L_LOS_*, i.e., *L_c_* = 4√(2*Wρ*).

The lateral displacement of the curved-straight guide exit with respect to the projected axis at the guide entrance (a useful quantity when considering instrument placement) is given by

(355) $$d_{tot}=d_{c}+d_{str}=\rho \left(1-\cos\left(\frac{L_{c}}{\rho }\right)\right)+L_{str}\sin\left(\frac{L_{c}}{\rho }\right).$$

Parameters for several guides that satisfy the PST guide conditions with the above constraints are summarized in Table 11.

**Fig. 72:**
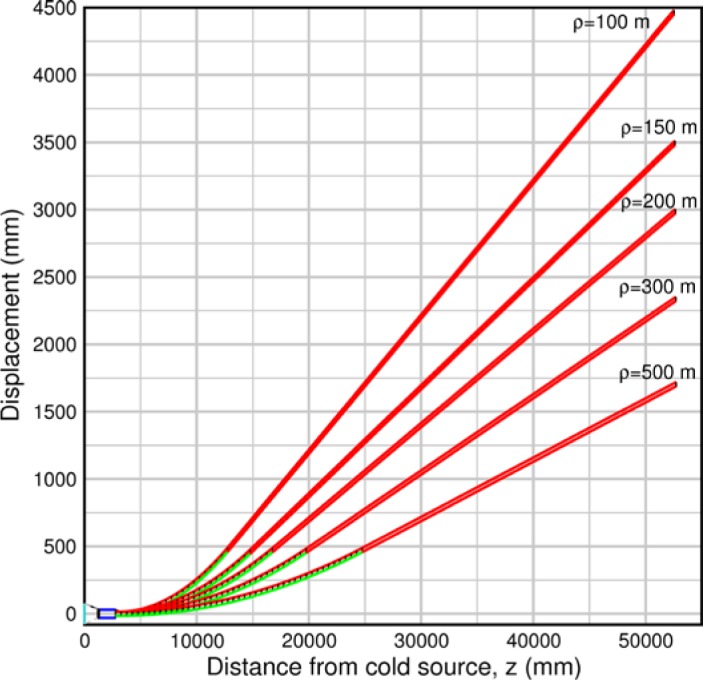
Modeled curved-straight guide geometries for simulation of the PST guide systems described in Table 11.

**Fig. 73:**
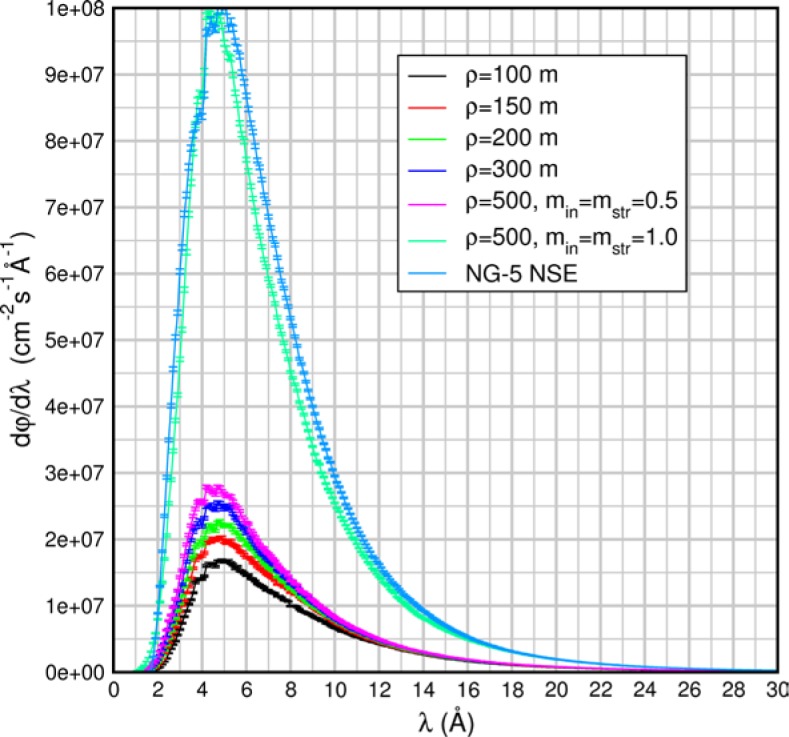
Simulated differential flux spectra (*dφ*/*dλ*) at the exits of the PST guide systems described in Table 11 and for the NCNR guide NG-5 (a ^58^Ni-coated optical filter), all assuming no velocity selector and no polarizer. The source model is the NCNR Unit 2 liquid hydrogen cold source.

**Fig. 74:**
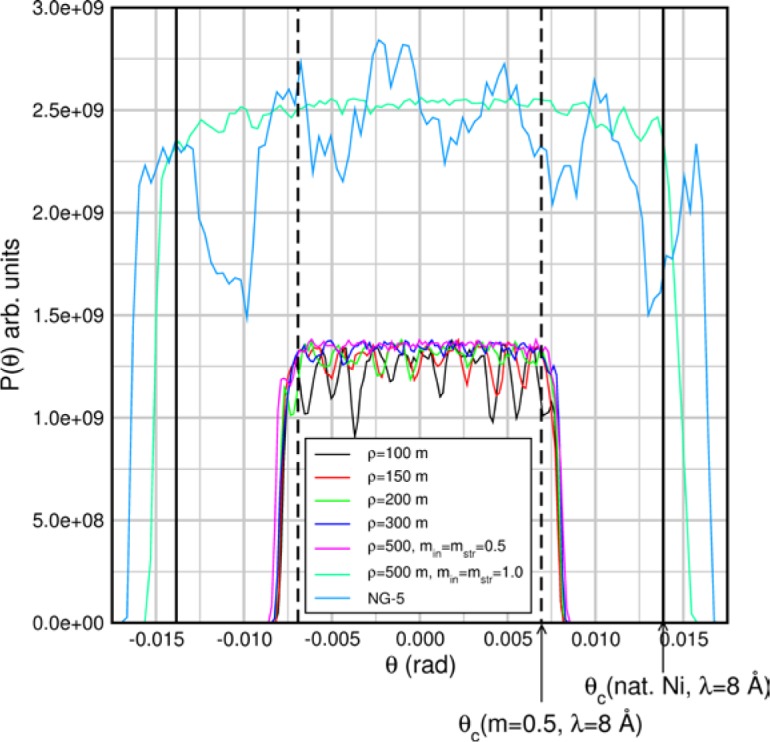
Horizontal angular distributions for the PST guide systems described in Table 11 and for the NCNR guide NG-5 (a ^58^Ni-coated optical filter) at *λ* = 8 Å. Note that *λ* = 8 Å is greater than *λ′* for the PST guides. The vertical solid lines show the idealized critical angles of natural Ni (solid lines) and for *m*=0.5 (dashed lines).

**Fig. 75:**
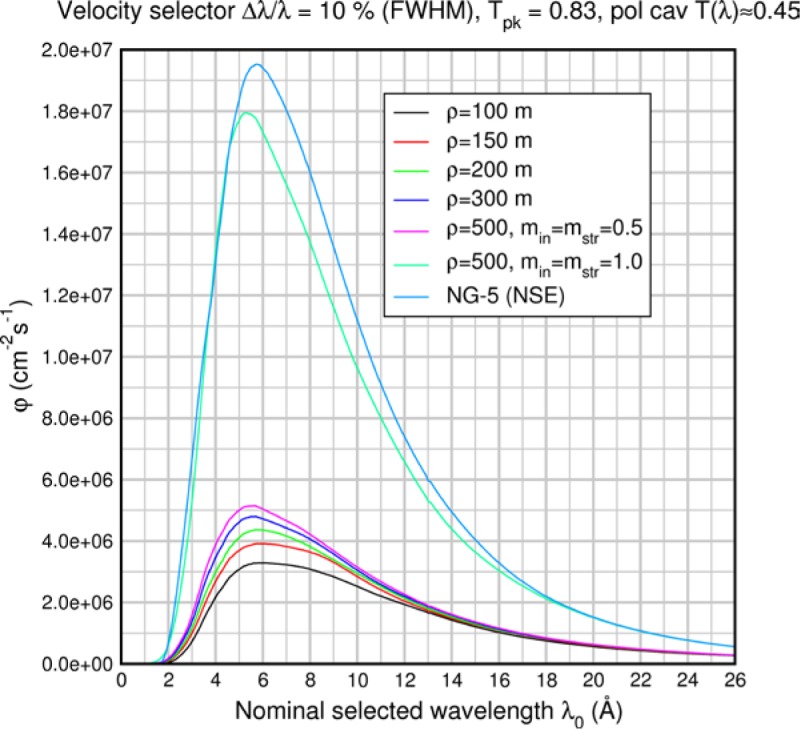
Simulated integral fluxes at the exits of the PST guide systems described in Table 11 and the NCNR guide NG-5 (a ^58^Ni-coated optical filter), assuming a Dornier-type velocity selector with *Δλ*/*λ* (FWHM) = 10 % and a polarizing cavity of wavelength-independent transmission 0.45 are placed in the beam.

The guide systems described in Table 11 are illustrated schematically in Fig. 72. Their simulated performance (with no velocity selector or polarizer) at the NCNR Unit 2 liquid hydrogen cold source is shown in Fig. 73. The simulated intensity of the NG-5 guide (a ^58^Ni-coated optical filter) at the NSE instrument is also shown under the same conditions of no velocity selector and no polarizing cavity. Figure 74 shows the horizontal angular distributions at *λ* = 8 Å (greater than *λ′* for all models in Table 11). The horizontal angular distributions show the expected uniformity within the critical angle limits of the straight sections (indicated by the vertical lines). Figure 75 shows simulated integral fluxes that could be expected at the guide exits when using a typical Dornier-type velocity selector operating at 10 % *Δλ*/*λ* (FWHM), with a polarizing cavity of wavelength-independent transmission 0.45. The predicted flux of the *m_str_* = 1 guide is comparable to that of the NG-5 under similar conditions, with a slightly reduced beam divergence (see Fig. 74).

## Figures and Tables

**Table 1:** Flipping efficiency versus full width (FW) (rectangular distribution) or full width at half maximum (FWHM) (triangular, Gaussian distributions) for *M* = 8 *π* coils. The pairs of values in parentheses for the triangular and Gaussian distributions are values of *Λ_FWHM_* and corresponding *P_disp_*/*P_ideal_* that give equivalent rms wavelength deviation with respect to the mean as the rectangular distribution with the value of *Λ_FW_* given in the first column. For the triangular distribution this implies *Λ_FWHM,triang_* = *Λ_FW,rect_*/√2. For the Gaussian distribution this implies *Λ_FWHM,gauss_* = √((2/3)ln2)*Λ_FW,rect_*.

*Λ_FW/FWHM_*	*P_disp_/P_ideal_* (*M=8*)		
	Rectangular (Eq. (42))	Triangular (Eq. (43))	Gaussian (Eq. (44))
0.1	0.98378	0.96831 (0.0707,0.98386)	0.96613 (0.0680,0.98393)
0.2	0.93778	0.88600 (0.1414,0.93888)	0.88155 (0.1360,0.93986)
0.3	0.86916	0.78127 (0.2121,0.87390)	0.77880 (0.2039,0.87765)
0.4	0.78750	0.67898 (0.2828,0.79961)	0.68064 (0.2719,0.80788)
0.5	0.70237	0.59115 (0.3536,0.72511)	0.59587 (0.3399,0.73834)

**Table 2:** Flipping efficiency versus full width (FW) (rectangular distribution) or full width at half maximum (FWHM) (triangular, Gaussian distributions) for *M* = 6 *π* coils. The pairs of values in parentheses for the triangular and Gaussian distributions are values of *Λ_FWHM_* and corresponding *P_disp_*/*P_ideal_* that give equivalent rms wavelength deviation with respect to the mean as the rectangular distribution with the value of *Λ_FW_* given in the first column. For the triangular distribution this implies *Λ_FWHM,triang_* = *Λ_FW,rect_*/√2. For the Gaussian distribution this implies *Λ_FWHM,gauss_* = √((2/3)ln2)*Λ_FW,rect_*.

*Λ_FW/FWHM_*	*P_disp_/P_ideal_* (*M=6*)		
	Rectangular (Eq. (42))	Triangular (Eq. (43))	Gaussian (Eq. (44))
0.1	0.98779	0.97600 (0.0707,0.98783)	0.97427 (0.0680,0.98787)
0.2	0.95266	0.91138 (0.1414,0.95329)	0.90715 (0.1360,0.95387)
0.3	0.89874	0.82378 (0.2121,0.90158)	0.81999 (0.2039,0.90396)
0.4	0.83194	0.73158 (0.2828,0.83959)	0.73102 (0.2719,0.84529)
0.5	0.75870	0.64689 (0.3536,0.77402)	0.64987 (0.3399,0.78399)

**Special Note**

Gähler and Golub [2] ignore the (usually small) angle *χ* and give an expression for 〈cos *ε*〉 in terms of the root mean square (rms) value of *ε*, *ε_rms_*, which is valid for small *ε*. For these reasons, their expression does not predict exactly the quantum mechanical flipping efficiency. The reader should beware of substituting, for example, FWHM values for *δv* into Eq. (33) of Ref. [2]. The latter equation is valid for discrete ±*δv* with respect to the mean, therefore their expression must be averaged over the appropriate velocity distribution, *F*(*v_n_*). For sufficiently small *ε*, the approximation is
$P\approx \langle \cos\varepsilon \rangle ≃\langle 1-\varepsilon^{2}/2\rangle =1-\langle \varepsilon^{2}\rangle /2\approx \cos\;\varepsilon_{rms}.$ with the combined effect of *M* similar *π* coils approximated by summing the *ε*’s in quadrature [ii], whence
$$P(M)\approx \langle \cos\sqrt{M}\varepsilon \rangle \approx \cos\sqrt{M}\varepsilon_{rms}.$$

**Table 3:** Phases for “perfect” single *π* coils with zero stray fields between coils showing how the field direction signs for the resonant component of the r.f. field apply (*B_rf_* is chosen to oscillate along the *x* axis).

Location	*y*	Neutron spin phase angle *φ*	r.f. field phase *ψ*
Entrance *A*	0	0	*ψ_A_*
Exit coil *A*	*l_A_*	*2ψ_A_*+sgn(*B_0_^A^*)(1/*v_i_*)*γ_n_*|*Β_0_|l_A_*	*ψ_A_*+sgn(*B_0_^A^*)(1/*v_i_*)*γ_n_*|*Β_0_|l_A_*
Entrance *B*	*l_A_+L_AB_*	*2ψ_A_*+sgn(*B_0_^A^*)(1/*v_i_*)*γ_n_*|*Β_0_|l_A_*	sgn(*B_0_^A^*)sgn(*B_0_^B^*)[*ψ_A_*+sgn(*B_0_^A^*)(1/*v_i_*)*γ_n_|B_0_*|(*l_A_+L_A_*)]
Exit coil *B*	*l_A_+L_AB_+l_B_*	2sgn(*B_0_^A^*)sgn(*B_0_^B^*)[*ψ_A_*+sgn(*B_0_^A^*)(1/*v_i_*)*γ_n_*|*Β_0_*|(*L_AB_+l_A_*)]+sgn(*B_0_^B^*)(1/*v_i_*)*γ_n_*|*Β_0_*|*l_B_*−*2ψ_A_*sgn(*B_0_^A^*)(1/*v_i_*)*γ_n_|Β_0_*|*l_A_^A^*	sgn(*B_0_^A^*)sgn(*B_0_^B^*)[*ψ_A_+*sgn(*B_0_^A^*)(1/*v_i_*)*γ_n_*|*Β_0_*|(*l_A_+L_AB_*)]+sgn(*B_0_^B^*)(1/*v_i_*)*γ_n_*|*Β_0_*|*l_B_*
Sample (non-spin flip or coherent scatterer)	*l_A_*+*L_AB_*+*l_B_*+*L_BS_*	2sgn(*B_0_^A^*)sgn(*B_0_^B^*)[*ψ_A_*+sgn(*B_0_^A^*)(1/*v_i_*)*γ_n_*|*Β_0_*|(*L_AB_*)]+sgn(*B_0_^B^+l_A_*)(1/*v_i_*)*γ_n_*|*Β_0_*|*l_B_*−*2ψ_A_*−sgn(*B_0_^A^*)(1/*v_i_*)*γ_n_*|*Β_0_|l_A_*	sgn(*B_0_^A^*)sgn(*B_0_^B^*)[*ψ_A_*+sgn(*B_0_^A^*)(1/*v_i_*)*γ_n_*|*Β_0_*|(*L_AB_+l_A_*)]+sgn(*B_0_^B^*)(1/*v_i_*)*γ_n_*|*Β_0_*|(*l_B_*+*L_BS_*)
Entrance *C*	*l_A_*+*L_AB_*+*l_B_*+*L_BS_*+*L_SC_*	2sgn(*B_0_^A^*)sgn(*B_0_^B^*)[*ψ_A_*+sgn(*B_0_^A^*)(1/*v_i_*)*γ_n_*|*Β_0_*|(*L_AB_+l_A_*)]+sgn(*B_0_^B^*)(1/*v_i_*)*γ_n_*|*Β_0_*|*l_B_*−*2ψ_A_*−sgn(*B_0_^A^*)(1/*v_i_*)*γ_n_*|*Β_0_|l_A_*	*ψ_C_*
Exit coil *C*	*l_A_*+*L_AB_*+*l_B_*+*L_BS_*+*L_SC_*+*l_C_*	2*ψ_C_*+sgn(*B_0_^C^*)(1/*v_f_*)*γ_n_*|*Β_1_|l_C_*−(2sgn(*B_0_^A^*)sgn(*B_0_^B^*)[*ψA*+sgn(*B_0_^A^*)(1/*v_i_*)*γ_n_*|*Β_0_*|(*L_AB_+l_A_*)]+sgn(*B_0_^B^*)(1/*v_i_*)*γ_n_*|*Β_0_*|*l_B_*−*2ψ_A_*)sgn(*B_0_^A^*)(1/*v_i_*)*γ_n_*|*Β_0_|l_A_*)	*ψ_C_*+sgn(*B_0_^C^*)(1/*v_f_*)*γ_n_*|*Β_1_|l_C_*
Entrance *D*	*l_A_*+*L_AB_*+*l_B_+L_BS_+L_SC_+l_C_*+*L_CD_*	2*ψ_C_*+sgn(*B_0_^C^*)(1/*v_f_*)*γ_n_*|*Β_1_|l_C_*−(2sgn(*B_0_^A^*)sgn(*B_0_^B^*)[*ψA*+sgn(*B_0_^A^*)(1/*v_i_*)*γ_n_*|*Β_0_*|(*L_AB_+l_A_*)]+sgn(*B_0_^B^*)(1/*v_i_*)*γ_n_*|*Β_0_*|*l_B_*−*2ψ_A_*)sgn(*B_0_^A^*)(1/*v_i_*)*γ_n_*|*Β_0_|l_A_*)	sgn(*B_0_^C^*)sgn(*B_0_^D^*)[*ψ_C_*+sgn(*B_0_^C^*)(1/*v_f_*)*γ_n_*|*Β_1_*|(*l_C_+L_CD_*)]
Exit coil *D*	*l_A_*+*L_AB_+l_B_+L_BS_*+*L_SC_*+*l_C_*+*L_CD_*+*l_D_*	2(sgn(*B_0_^C^*)sgn(*B_0_^D^*)[*ψ_C_*+sgn(*B_0_^C^*)(1/*v_f_*)*γ_n_*|*Β_1_*|(*l_C_+L_CD_*)])+sgn(*B_0_^D^*)(1/*v_f_*)*γ_n_*|*Β_1_|l_D_*−{2*ψ_C_*+sgn(*B_0_^C^*)(1/*v_f_*)*γ_n_*|*Β_1_|l_C_*−(2sgn(*B_0_^A^*)sgn(*B_0_^B^*)[*ψ_A_*+sgn(*B_0_^A^*)(1/*v_i_*)*γ_n_*|*Β_0_*|(*L_AB_+l_A_*)]+sgn(*B_0_^B^*)(1/*v_i_*)*γ_n_*|*Β_0_*|*l_B_*−*2ψ_A_*−sgn(*B_0_^A^*)(1/*v_i_*)*γ_n_*|*Β_0_|l_A_*)}	2(sgn(*B_0_^C^*)sgn(*B_0_^D^*)[*ψ_C_*+sgn(*B_0_^C^*)(1/*v_f_*)*γ_n_*|*Β_1_*|(*l_C_+L_CD_*)])+sgn(*B_0_^D^*)(1/*v_f_*)*γ_n_*|*Β_1_|l_D_*

**Table 4:** Phases for “perfect” single *π* coils with zero stray fields between coils applying the field direction signs indicated in Fig. 5.

Location	*y*	Neutron spin phase angle *φ*	r.f. field phase *ψ*
Entrance *A*	0	0	*ψ_A_*
Exit coil *A*	*l_A_*	*2ψ_A_*+(1/*v_i_*)*γ_n_*|*Β_0_|l_A_*	*ψ_A_*+(1/*v_i_*)*γ_n_*|*Β_0_|l_A_*
Entrance *B*	*l_A_*+*L_AB_*	*2ψ_A_*+(1/*v_i_*)*γ_n_*|*Β_0_|l_A_*	*ψ_A_*+(1/*v_i_*)*γ_n_*|*Β_0_*|(*L_AB_+l_A_*)
Exit coil *B*	*l_A_*+*L_AB_*+*l_B_*	(1/*v_i_*)*γ_n_*|*Β_0_*|(2*L_AB_+l_A_+l_B_*)	*ψ_A_*+(1/*v_i_*)*γ_n_*|*Β_0_*|(*L_AB_+l_A_+l_B_*)
Sample (non-spin flip or coherent scatterer)	*l_A_*+*L_AB_*+*l_B_*+*L_BS_*	(1/*v_i_*)*γ_n_*|*Β_0_*|(2*L_AB_+l_A_+l_B_*)	*ψ_A_*+(1/*v_i_*)*γ_n_*|*Β_0_*|(*L_AB_+l_A_+l_B_*+*L_BS_*)
Entrance *C*	*l_A_*+*L_AB_*+*l_B_*+*L_BS_*+*L_SC_*	(1/*v_i_*)*γ_n_*|*Β_0_*|(2*L_AB_+l_A_+l_B_*)	*ψ_C_*
Exit coil *C*	*l_A_*+*L_AB_*+*l_B_*+*L_BS_*+*L_SC_*+*l_C_*	2*ψ_C_*−(1/*v_f_*)*γ_n_*|*Β_1_|l_C_*−(1/*v_i_*)*γ_n_*|*Β_0_*|(2*L_AB_+l_A_+l_B_*)	*ψ_C_*−(1/*v_f_*)*γ_n_*|*Β_1_|l_C_*
Entrance *D*	*l_A_*+*L_AB_*+*l_B_*+*L_BS_*+*L_SC_*+*l_C_*+*L_CD_*	2*ψ_C_*−(1/*v_f_*)*γ_n_*|*Β_1_|l_C_*−(1/*v_i_*)*γ_n_*|*Β_0_*|(2*L_AB_+l_A_+l_B_*)	*ψ_C_*−(1/*v_f_*)*γ_n_*|*Β_1_*|(*l_C_+L_CD_*)
Exit coil *D*	*l_A_*+*L_AB_*+*l_B_*+*L_BS_*+*L_SC_*+*l_C_*+*L_CD_+l_D_*	(1/*v_i_*)*γ_n_*|*Β_0_*|(2*L_AB_+l_A_+l_B_*)−(1/*v_f_*)*γ_n_*|*Β_1_*|(*2L_CD_+l_C_*)*+l_D_*	*ψ_C_*−(1/*v_f_*)*γ_n_*|*Β_1_*|(*l_C_+L_CD_+l_D_*)

**Table 5:** Phase angles for a “perfect” 4−*N* = 2 bootstrap coil NRSE applying the static field signs as indicated in Fig. 6 for *N* = 2.

Location	*y*	Neutron spin phase angle *φ*	r.f. field phase *ψ*
Entrance *A_1_*	0	0	*ψ_A1_*
Exit *A_1_*	*l_A1_*	*2ψ_A1_*+(1/*v_i_*)*γ_n_*|*Β_0_|l_A1_*	*ψ_A1_*+(1/*v_i_*)*γ_n_*|*Β_0_|l_A1_*
Entrance *A_2_*	*l_A1_*+*l_g_*	*2ψ_A1_*+(1/*v_i_*)*γ_n_*|*Β_0_|l_A1_*	−*ψ_A1_*−(1/*v_i_*)*γ_n_*|*Β_0_*|(*l_A1_+l_g_*)
Exit *A_2_*	*l_A1_*+*l_g_+l_A2_*	−4*ψ_A1_*−(1/*v_i_*)*γ_n_*|*Β_0_*|(3*l_A1_+2l_g_+l_A_*)	−*ψ_A1_*−(1/*v_i_*)*γ_n_*|*Β_0_*|(*l_A1_+l_g_+l_A2_*)
Entrance *B_1_*	*l_A1_+l_g_+l_A2_+L_AB_*	−4*ψ_A1_*−(1/*v_i_*)*γ_n_*|*Β_0_*|(3*l_A1_+2l_g_+l_A_*)	−*ψ_A1_*−(1/*v_i_*)*γ_n_*|*Β_0_*|(*l_A1_+l_g_*+*l_A2_+L_AB_*)
Exit *B_1_*	*l_A1_+l_g_+l_A2_+L_AB_+l_B1_*	*2ψ_A1_*+(1/*v_i_*)*γ_n_*|*Β_0_*|(*l_A1_*−*l_A2_*−*2L_AB_*−*l_B1_*)	−*ψ_A1_*−(1/*v_i_*)*γ_n_*|*Β_0_*|(*l_A1_+l_g_+l_A2_+L_AB_+l_B1_*)
Entrance *B_2_*	*l_A1_+2l_g_+l_A2_+L_AB_+l_B1_*	*2ψ_A1_*+(1/*v_i_*)*γ_n_*|*Β_0_*|(*l_A1_*−*l_A2_*−*2L_AB_*−*l_B1_*)	*ψ_A1_*+(1/*v_i_*)*γ_n_*|*Β_0_*|(*l_A1_+2l_g_+l_A2_+L_AB_+l_B1_*)
Exit *B_2_*	*l_A1_+2l_g_+l_A2_+L_AB_+l_B1_+l_B2_*	(1/*v_i_*)*γ_n_*|*Β_0_*|(*l_A1_+4l_g_+3l_A2_+4L_AB_+3l_B1_+l_B2_*) *= φ_B2_’*	*ψ_A1_*+(1/*v_i_*)*γ_n_*|*Β_0_*|(*l_A1_+2l_g_+l_A2_+L_AB_+l_B1_+l_B2_*)
Sample (non-spin flip or coherent scatterer)	*l_A1_+2l_g_+l_A2_+L_AB_+l_B1_+l_B2_+L_BS_*	*φ_B2_*’	*ψ_A1_*+(1/*v_i_*)*γ_n_*|*Β_0_*|(*l_A1_+2l_g_*+*l_A2_+L_AB_*+*l_B1_*+*l_B2_+L_BS_*)
Entrance *C_1_*	*l_A1_+2l_g_+l_A2_+L_AB_+l_B1_+l_B2_+L_BS_+ L_SC_*	*φ_B2_’*	*ψ_C1_*
Exit *C_1_*	*l_A1_+2l_g_+l_A2_+L_AB_+l_B1_+l_B2_+L_BS_+L_SC_+l_C1_*	*2ψ_C1_*−(1/*v_f_*)*γ_n_*|*Β_1_|l_C1_*−*φ_B2_’*	*ψ_C1_*−(1/*v_f_*)*γ_n_*|*Β_1_|l_C1_*
Entrance *C_2_*	*l_A1_+3l_g_+l_A2_+L_AB_+l_B1_+l_B2_+L_BS_+L_SC_+l_C1_*	*2ψ_C1_*−(1/*v_f_*)*γ_n_*|*Β_1_|l_C1_*−*φ_B2_’*	−*ψ_C1_*+(1/*v_f_*)*γ_n_*|*Β_1_*|(*l_C1_+l_g_*)
Exit *C_2_*	*l_A1_+3l_g_+l_A2_+L_AB_+l_B1_+l_B2_+L_BS_+L_SC_+l_C1_+l_C2_*	−4*ψ_C1_*+1(1/*v_f_*)*γ_n_*|*B_1_*|(*3l_C1_+2l_g_*+*l_C2_*)*+φ_B2_’*	−*ψ_C1_*+(1/*v_f_*)*γ_n_*|*Β_1_*|(*l_C1_+l_g_*+*l_C2_*)
Entrance *D_1_*	*l_A1_+3l_g_+l_A2_+L_AB_+l_B1_+l_B2_+L_BS_+L_SC_+l_C1_+L_C2_+l_CD_*	−4*ψ_C1_*+(1/*v_f_*)*γ_n_*|*Β_1_*|(*3l_C1_+2l_g_*+*l_C2_*)*+φ_B2_’*	−*ψ_C1_*+(1/*v_f_*)*γ_n_*|*Β_1_*|(*l_C1_+l_g_*+*l_C2_+L_CD_*)
Exit *D_1_*	*l_A1_+3l_g_+l_A2_+L_AB_+l_B1_+l_B2_+L_BS_+L_SC_+l_C1_+l_C2_+L_CD_ +l_D1_*	*2ψ_C1_*+(1/*v_f_*)*γ_n_*|*Β_1_*|(−*l_C1_*+*l_C2_+2L_CD_+l_D1_)*−*φ_B2_’*	−*ψ_C1_*+(1/*v_f_*)*γ_n_*|*Β_1_*|(*l_C1_+l_g_*+*l_C2_+L_CD_+l_D1_*)
Entrance *D_2_*	*l_A1_+4l_g_+l_A2_+L_AB_+l_B1_+l_B2_+L_BS_+L_SC_+l_C1_+L_C2_+l_CD_+l_D1_*	2*ψ_C1_*+(1/*v_f_*)*γ_n_*|*Β_1_*|(−*l_C1_*+*l_C2_+2L_CD_+l_D1_)*−*φ_B2_’*	*ψ_C1_*−(1/*v_f_*)*γ_n_*|*Β_1_*|(*l_C1_+2l_g_*+*l_C2_+L_CD_+l_D1_*)
Exit *D_2_*	*l_A1_+4l_g_+l_A2_+L_AB_+l_B1_+l_B2_+L_BS_+L_SC_+l_C1_+l_C2_+L_CD_+l_D1_+l_D2_*	*φ_B2_’*−(1/*v_f_*)*γn*|*Β_1_*|(*l_C1_+4l_g_*+3*l_C2_+4L_CD_+3l_D1_+l_D2_*)=(1/*v_i_*)*γ_n_*|*Β_0_*|(*l_A1_+4l_g_+3l_A2_+4L_AB_+3l_B1_+l_B2_*)−(1/*v_f_*)*γn*|*Β1*|(*l_C1_+4l_g_*+3*l_C2_+4L_CD_+3l_D1_+l_D2_)*	*ψ_C1_*−(1/*v_f_*)*γ_n_*|*Β_1_*|(*l_C1_+2l_g_*+*l_C2_*+*L_CD_+l_D1_+l_D2_*)

**Table 6:** Spin flip probabilities for non-magnetic samples.

Scatter	Coherent	Incoherent	
		Spin	Isotope
Non-spin flip	1	1/3	1
Spin flip	0	2/3	0

**Table 7:** Parameter tolerances required to achieve a specified minimum elastic (resolution) polarization *P_x_^0^* for *τ_NRSE_* = 30 ns at *λ*= 8 Å with the above spectrometer dimensions (*B_0_*=0.0393 T) and approximately equal contributions to the depolarization coming from *ΔB_0_*, *Δl_B0_*, and *Δθ_max_.* The final column normalizes *Δθ_max_* to the critical angle of natural Ni at the same wavelength.

For *P_x_^0^* >	*ΔB_0_* (FWHM)[(μT] <	*Δl_B0_* (FWHM)[(μm] <	*Δθ_max_* [mrad] <	*Δθ_max_/θ_c_*(8Å, nat. Ni) %
0.1	244	71	5.4	39.0
0.2	195	59	4.9	35.7
0.3	164	51	4.6	33.2
0.4	140	45	4.3	31.0
0.5	120	39	4.0	28.9
0.6	101	33	3.7	26.8
0.7	84	28	3.4	24.5
0.8	65	22	3.0	21.8
0.9	45	15	2.5	18.0
0.95	31	11	2.1	15.1
0.99	14	5	1.4	10.0

**Table 8:** Circuit values at exact impedance matching (*C_1_* and *C_2_* are given by Eqs. (251) and (252)).

	*C_1_*	*L*	*R*	*C_2_*	*C_1_LR*	*C_2_//C_1_LR*	*Z_0_*, *C_2_//C_1_LR*
*V*	$\frac{v_{in}}{2C_{1}}\left(C_{2}-\frac{j}{Z_{0}\omega }\right)$	$\frac{v_{in}}{2}\left(j\frac{1}{Z_{0}}-\omega C_{2}\right)\omega L$	$\frac{v_{in}}{2}\left(\frac{1}{Z_{0}}+j\omega C_{2}\right)R$	$\frac{V_{in}}{2}$	$\frac{V_{in}}{2}$	$\frac{V_{in}}{2}$	*V_in_*
*I*	$\frac{v_{in}}{2}\left(\frac{1}{Z_{0}}+j\omega C_{2}\right)$	$\frac{v_{in}}{2}\left(\frac{1}{Z_{0}\;}+j\omega C_{2}\right)$	$\frac{v_{in}}{2}\left(\frac{1}{Z_{0}}+j\omega C_{2}\right)$	$\frac{jV_{in}\omega C_{2}}{2}$	$\frac{v_{in}}{2}\left(\frac{1}{Z_{0}}+j\omega C_{2}\right)$	$\frac{V_{in}}{2Z_{0}}$	$\frac{V_{in}}{2Z_{0}}$
*Z*	$-\frac{j}{\omega C_{1}}$	*jωL*	*R*	$-\frac{j}{\omega C_{2}}$	$R+j\left(\omega L-\frac{1}{\omega C_{1}}\right)$	*Z_0_*	*2Z_0_*

**Table 9:** Flipping efficiency for triangular distributions of (i) *Δλ* (dispersion only) and (ii) *Δλ* combined with effects of *Δ*(*B_rf_ l_rf_*) using the criterion expressed in Eq. (280) for *M* = 8 *π* coils.

*Λ_FWHM_* (= *Δλ_FWHM_*/〈*λ_i_*〉) (%)	*P_disp_/P_ideal_* (*M* =8)	
	*Λ_FWHM_* (only)	with *Λ_FWHM_^eff^* = 1.05 *Λ_FWHM_*
10	0.96831	0.96519
20	0.88600	0.87604
30	0.78127	0.76533
40	0.67898	0.66009
50	0.59115	0.57179

**Table 10:** Estimated minimum (fixed) guide field magnitudes, |*B_guide_*|, required to produce a *π*/2 rotation of a neutron spin that follows a guide field rotation of *π*/2 radians over a distance of 0.5 m. The actual conditions for adiabatic rotation are usually determined experimentally.

*v_n_* (ms^−1^)	*λ* (Å)	*Ω* (*ψ* =*π*/2,*d* = 0.5m) = *πv_n_*	|*B_guide_*| for *θ* =		
			*π*/18 (10°)	*π*/180 (1°)	*π*/1800 (0.1°)
1000	≈ 4	1000*π*	≈ 100*μ*T	≈ 1 mT	≈ 10 mT
500	≈ 8	500*π*	≈ 50 *μ*T	≈ 0.5 mT	≈ 5 mT

**Table 11:** Parameters for guides satisfying idealized PST guide conditions for *λ* > *λ′*, given the constraints *λ_c_* = 4 Å, *L_tot_* = 50 m, *W* = 3 cm, *L_c_* = 2*L_LOS_* with *m_in_* ≥ 0.5 and *m_str_* ≥ 0.5, for several radii of curvature of the curved sections.

*ρ* (m)	*L_LOS_* (m)	*L_c_* (m)	*L_str_* (m)	*L_str_*(min) [minimum for ideal PST (*λ* > *λ′*)] (m)	*m_out_* (*λ_c_* = 4Å)	*m_in_*	*m_str_*	*λ′* (Å)	Displacement of guide exit *d_tot_* (m)
100	4.90	9.80	40.20	8.58	3.54	0.5	0.5	4.04	4.41
150	6.00	12.00	38.00	8.54	2.89	0.5	0.5	4.06	3.52
200	6.93	13.86	36.14	8.50	2.50	0.5	0.5	4.08	2.98
300	8.49	16.97	33.03	8.41	2.04	0.5	0.5	4.13	2.35
500	10.95	21.91	28.09	8.23	1.58	1.0	0.5	4.22	1.71
500	10.95	21.91	28.09	3.36	1.58	1.0	1.0	5.16	1.71

## Footnotes

1 It has been proposed to ramp |*B_rf_*| for situations where the neutron energy is strongly correlated with its time of emission, for example at pulsed neutron sources. However for continuous sources this is not feasible.

2 Note that we use the symbol “*Δ*” to imply a *statistical spread*, not to be confused with the “***δ*”** used to imply a *difference*, when referring to the asymmetry *B_0_L_0_ - B_1_L_1_*.

3 Certain commercial equipment, instruments, or materials are identified in this paper to foster understanding. Such identification does not imply recommendation or endorsement by the National Institute of Standards and Technology, nor does it imply that the materials or equipment identified are necessarily the best available for the purpose.

## 10. Appendix A: Summary of Useful NRSE Formulas

(*L_0_* = length between coil centers, *l*=total coil length, *l_Β0_* = length of one *π*-flipper coil assuming the static field region encloses the r.f. field region, *l_π_*=length of combined r.f. and static field region (usually=*l_rf_* for *π* flipper coils), *N*=number of *π* flipper coils in Bootstrap, *M*=total number of *π*-coils traversed by the beam in the instrument).

**Larmor angular frequency (=*ω_rf_* at resonance)**

*ω*_0_ = *γ_n_B*_0_   Eq. (5)   with $\gamma_{n}=\frac{2|\mu_{n}|}{\hbar }=1.832472\times 10^{8}\;{\text{rad s}}^{-1}T^{-1}$   Eq. (6).

**Larmor frequency (=r.f. frequency at resonance)**

$v_{0}\left[\text{MHz}\right]=v_{rf}^{reson}\left[\text{MHz}\right]=\frac{\gamma_{n}B_{0}}{2\pi }=29.1647B_{0}[T]$   Eq. (8).

**Larmor period=r.f. period at resonance**

$\tau_{0}[\mu s]=\frac{1}{v_{0}[\text{MHz}]}=\frac{0.03429}{B_{0}[T]}$ from Eq. (8).

**“Effective” 4-coil NRSE precession frequency with bootstrap factor *N***

$v_{eff}[\text{MHz}]=2Nv_{0}[\text{MHz}]=58.3294NB_{0}[T]$.

**Actual number of precessions in one *π* flipper coil length *l_B0_*, static field *B_0_***

$N_{prec}^{\pi }=\frac{v_{0}l_{B_{0}}}{v_{n}}=\frac{\varphi_{\pi }}{2\pi }=\frac{\gamma_{n}}{2\pi }\frac{m_{n}}{h}B_{0}l_{B_{0}}\lambda =7.3722\times 10^{3}B_{0}[T]l_{B_{0}}[m]\lambda \left[Å\right]$.

**Approximate full width at half maximum of resonance curve (i.e., a 50 % drop in polarizing efficiency) for a general value of *l_π_* is very well fitted by**

$\Delta v_{FWHM}[\text{Hz}]=\frac{3.16\times 10^{3}}{l_{\pi }[m]\lambda_{n}\left[Å\right]}$   Eq. (50).

**Frequency shift for 1 % drop in the polarizing efficiency is well fitted by**

$\Delta v_{99\%}[\text{Hz}]=\frac{198}{l_{\pi }[m]\lambda_{n}\left[Å\right]}$   Eq. (51).

**Effects of Dispersion: Flipping efficiencies**

Flipping efficiency for a wavelength *λ_i_* for single flipper tuned for resonance and for exact *π* flips for the mean neutron wavelength 〈*λ_i_*〉.

$\frac{P_{disp}}{P_{ideal}}=\sin^{2}\left(\frac{\pi }{2}\frac{\lambda_{i}}{\langle \lambda_{i}\rangle }\right)$   Eq. (30).

Averaged over various incident wavelength distributions:

- Rectangular
  ${\langle \frac{P_{disp}}{P_{ideal}}\rangle }_{1\;coil}=\frac{1}{2}+\frac{1}{\Lambda_{FW}\pi }\sin(\frac{\pi }{2}\Lambda_{FW})$   Eq. (34), $\text{with}\;\Lambda_{FW}=\Delta \lambda_{FW}/\langle \lambda_{i}\rangle$   Eq. (33).
- Triangular
  ${\langle \frac{P_{disp}}{P_{ideal}}\rangle }_{1\;coil}=\frac{1}{2}+\frac{1}{\pi^{2}\Lambda_{FWHM}^{2}}[1-\cos(\pi \Lambda_{FWHM})]$   Eq. (37), $\text{with}\;\Lambda_{FWHM}=\Delta \lambda_{FWHM}/\langle \lambda_{i}\rangle$   Eq. (36).
- Gaussian
  ${\langle \frac{P_{disp}}{P_{ideal}}\rangle }_{1coil}=\frac{1}{2}\left(1+\exp\left[-{\left(\frac{\pi }{4}\right)}^{2}\frac{\Lambda_{FWHM}^{2}}{\ln2}\right]\right)$   Eq. (40), $\text{with}\;\Lambda_{FWHM}=\Delta \lambda_{FWHM}/\langle \lambda_{i}\rangle$   Eq. (36).

**Approximate instrumental tolerances to achieve resolution goal *P_x_^0^* (equal contributions from *ΔB_0_*, *Δl_B0_*, and *Δθ_max_*) − see Sec. 6**

- Tolerance on *B_0_* field in each *π*-flipper for a 4-*N* coil instrument (Gaussian -equal contributions)
  $\Delta B_{0}^{FWHM}\approx \left|B_{rf}\right|\sqrt{\frac{\kappa }{3N}\left(\frac{\kappa }{N}+2\right)}$   Eq. (203)   with $\kappa =\ln2\ln\left(1/P_{x}^{0}\right)$.
- Tolerance on coil flatness in each *π*-flipper for a 4-*N* coil instrument (Gaussian – equal contributions)
  $\Delta l_{B_{0}}^{FWHM}=\frac{2h}{m_{n}\gamma_{n}}\frac{\sqrt{\ln2\ln\left(\frac{1}{P_{x}^{0}}\right)}}{\sqrt{3N}B_{0}\lambda_{i}}\approx \frac{2.08\times 10^{-5}\sqrt{\ln\left(\frac{1}{P_{x}^{0}}\right)}}{\sqrt{N}B_{0}[T]\lambda_{i}\left[Å\right]}\;\text{meters}$   Eq. (204).
- Tolerance on beam divergence in each arm of a 4-*N* coil instrument (uniform incident and scattered polar angles up to *Δθ*_max_ − equal contributions)
  $\Delta \theta_{\max}\approx \sqrt{\frac{h\sqrt{45\ln\left(\frac{1}{P_{x}^{0}}\right)}}{6Nm_{n}\gamma_{n}B_{0}L_{0}\lambda_{i}}}\approx 4.91\times 10^{-3}\sqrt{\frac{\sqrt{\ln\left(\frac{1}{P_{x}^{0}}\right)}}{NB_{0}[T]L_{0}[m]\lambda_{i}\left[Å\right]}}[\text{rad}]$   Eq. (205), for $\left|\Delta \theta_{\max}\right|<∼\frac{6.7\times 10^{-3}}{\sqrt{NB_{0}[T]L_{0}[m]\lambda_{i}\left[Å\right]}}[\text{rad}]$.

**Net phase change in 4 equal coils, midpoints of coils in first arm separated by *L_0_*, midpoints of coils in second arm separated by *L_1_***

$\varphi_{NRSE}=2N\gamma_{n}\left[\frac{B_{0}L_{0}}{v_{i}}-\frac{B_{1}L_{1}}{v_{f}}\right]=\frac{2Nm_{n}\gamma_{n}}{h}[B_{0}L_{0}\lambda_{i}-B_{1}L_{1}\lambda_{f}]$   Eq. (69).

When tuned for QENS with *B_0_L_0_* = *B_1_L_1_*:

$\varphi_{NRSE}[\text{rads}]=92641.8N\left[B_{0}[T]L_{0}[m]\lambda_{i}\left[Å\right]-B_{1}[T]L_{1}[m]\lambda_{f}\left[Å\right]\right]$.

**NRSE Fourier time (*v_f_* = *v_i_* + *δv, δv* «*v_i_* approximation)**

$\tau_{NRSE}=\frac{2N\hbar \gamma_{n}B_{0}L_{0}}{m_{n}v_{i}^{3}}=\frac{\gamma_{n}}{\pi }{\left(\frac{m_{n}}{h}\right)}^{2}NB_{0}L_{0}\lambda_{i}^{3}=2N\nu_{0}L_{0}{\left(\frac{m_{n}}{h}\right)}^{2}\lambda_{i}^{3}$   Eq. (119),

where

$\begin{array}{r} \tau_{NRSE}[\text{ns}]=0.37271N\;B_{0}[T]L_{0}[m]{\left(\lambda_{i}\left[Å\right]\right)}^{3}\\ \equiv 1.27794\times 10^{-2}N\nu_{0}[\text{MHz}]L_{0}[m]{\left(\lambda_{i}\left[Å\right]\right)}^{3} \end{array}$   Eq. (120).

**Resolution function approximations for no sample, isotope incoherent elastic scattering, or small *ω*, small divergence (apart from depolarizations resulting from instrumental imperfections and flipper coil dispersion)**

Defining $A=\frac{2N\gamma_{n}m_{n}B_{0}}{h}\delta L$   (Eq. (124)):

- Purely monochromatic (“perfect instrument”)
  $P_{x}(\omega \to 0)\approx S(Q)\cos(A\lambda_{0})$   (Eq. (125)),
  with a periodicity given by
  $\delta {\left(BL\right)}_{2\pi }=\frac{h}{m_{n}}\frac{\pi }{\gamma_{n}N}\frac{1}{\lambda_{0}}=\frac{\pi \nu_{0}}{\gamma_{n}N}$   (Eq. (126)).
- Rectangular incident wavelength spectrum (“perfect instrument”)
  $P_{x}(\omega \to 0)\approx \frac{2S(Q)}{A\Delta \lambda_{FW}}\sin\left(A\frac{\Delta \lambda_{FW}}{2}\right)\cos\left(A\langle \lambda_{i}\rangle \right)$   (Eq. (127)).
- Triangular incident wavelength spectrum (“perfect instrument”)
  $P_{x}(\omega \to 0)\approx \frac{S(Q)}{\Delta \lambda_{FWHM}^{2}A^{2}}\left\{2\cos\left(A\langle \lambda_{i}\rangle \right)\left[1-\cos\left(A\Delta \lambda_{FWHM}\right)\right]\right\}$   (Eq. (128)).

**Quasielastic signal approximations, symmetric *S*(*Q, ω), Γ(Q)* ≈ *ħDQ_el_*^2^, *δL* → 0, small divergence (apart from depolarizations resulting from instrumental imperfections and flipper coil dispersion)**

Defining $K=16\pi \gamma_{n}{\left(\frac{m_{n}}{h}\right)}^{2}NB_{0}L_{0}D\sin^{2}\theta =\frac{16\pi^{2}D\sin^{2}\theta }{\lambda_{i}^{3}}\tau_{NRSE}$   (Eq. (134)):

- Purely monochromatic (“perfect instrument”)
  $P_{x}(\delta L\to 0)\approx \exp(-K\lambda_{0})$   (Eq. (135)).
- Rectangular incident wavelength spectrum (“perfect instrument”)
  $P_{x}(\delta L\to 0)\approx \frac{\sinh(K\Delta \lambda_{FW}/2)}{\left(K\Delta \lambda_{FW}/2\right)}\exp\left(-K\langle \lambda_{i}\rangle \right)$   Eq. (136)).
- Triangular incident wavelength spectrum (“perfect instrument”)
  $P_{x}(\delta L\to 0)\approx \frac{2\left(\cosh\left(K\Delta \lambda_{FWHM}\right)-1\right)}{K^{2}\Delta \lambda_{FWHM}^{2}}\exp\left(-K\langle \lambda_{i}\rangle \right)$   (Eq. (137)).
- Gaussian incident wavelength spectrum (“perfect instrument”)
  $\begin{array}{l} P_{x}(\delta L\to 0)\approx \frac{1}{2}\exp\left(-K\langle \lambda_{i}\rangle \right)\exp\left(\frac{\sigma^{2}K^{2}}{2}\right)\left(\text{erf}\left(\frac{\langle \lambda_{i}\rangle -\sigma^{2}K}{\sqrt{2}\sigma }\right)+1\right)\\ \sigma =\frac{\Delta \lambda_{FWHM}}{\sqrt{8\ln2}} \end{array}$   (Eq. (138)).

**Approximate maximum achievable static field for *n* turns cm^−1^ (long solenoid approximation)**

$B_{0}[T]\approx 4\pi \times 10^{-7}n[m^{-1}]I[A]≃1.26\times 10^{-6}n[m^{-1}]I[A]$   Eq. (213).

**Required current in static field coil to produce a static field *B_0_***

$I[A]=\frac{2.5\times 10^{6}}{\pi }\frac{B_{0}[T]}{n[m^{-1}]}\approx 8\times 10^{5}\frac{B_{0}[T]}{n[m^{-1}]}$   Eq. (214).

**Magnetic pressure acting outwards on *B_0_* coil windings**

$P_{mag}[{\text{Nm}}^{-2}]=\frac{{\left(B[T]\right)}^{2}}{8\pi \times 10^{-7}}\approx 4\times 10^{5}{\left(B[T]\right)}^{2}$   Eq. (241).

**Approximate deflection of unconstrained rectangular wire (thickness *t*) winding, length *l_u_* due to magnetic pressure**

$y_{\max}[m]\approx 1.25\times 10^{4}\frac{{\left(B[T]\right)}^{2}l_{u}{\left[m\right]}^{4}}{E[{\text{Nm}}^{-2}]t{\left[m\right]}^{3}}$   Eq. (246).

**Resistance of static field coil of external surface area *A_surf_* with tightly-wound, rectangular cross-section wire windings of thickness *t***

$R=\frac{\rho (T)n^{2}A_{surf}}{t}$   Eq. (222).

**Required voltage across static field coil of external surface area *A_surf_* with tightly-wound, rectangular cross-section wire windings of thickness *t***

$V[V]=I[A]R[\Omega ]=\frac{2.5\times 10^{6}}{\pi }\frac{\rho (T)[\Omega m]n[m^{-1}]A_{surf}[m^{2}]}{t[m]}B_{0}[T]$   Eq. (224).

**Power dissipated in static field coil of external surface area *A_surf_* with tightly-wound, rectangular cross-section wire windings of thickness *t***

$\begin{array}{l} P[W]=I[A]V[V]={\left(I[A]\right)}^{2}R[\Omega ]\\ \approx \frac{6.25\times 10^{12}}{\pi^{2}}\frac{\rho (T)[\Omega m]A_{surf}[m^{2}]}{t[m]}{\left(B_{0}[T]\right)}^{2} \end{array}$   Eq. (226).

Specifically for Al windings down to *T* ~ 80 K

$P_{Al}[W]\approx {\left(B_{0}[T]\right)}^{2}\frac{A_{surf}[m^{2}]}{t[m]}\left(72.2T(K)-4.37\times 10^{3}\right)$   Eq. (235).

***B_rf_* required for *π* flip (amplitude of r.f. field=2*B_rf_*)**

$B_{rf}[T]≃\frac{6.782232\times 10^{-5}}{l_{rf}[m]\lambda_{n}\left[Å\right]}=\frac{6.782232\times 10^{-5}}{l_{\pi }[m]\lambda_{n}\left[Å\right]}$   Eq. (14).

**Required peak amplitude of r.f. field**

$B_{rf}^{pk}[T]=2B_{rf}[T]≃\frac{1.35645\times 10^{-4}}{l_{rf}[m]\lambda_{n}\left[Å\right]}=\frac{1.35645\times 10^{-4}}{l_{\pi }[m]\lambda_{n}\left[Å\right]}$   Eq. (15).

**Approximate skin-depth in aluminum**

$\delta_{Al}[\text{mm}]≃\frac{83}{\sqrt{\nu [\text{Hz}]}}$   Eq. (275).

**Approximate ratio of resistance at frequency *ν* to D.C. resistance for rectangular cross-section wire windings with *h* » *t***

$\frac{R(\nu )}{R_{0}}\approx \frac{t}{2\delta \left[1-\exp\left(-\frac{t}{2\delta }\right)\right]}$   Eq. (276).

**Approximate required peak current in r.f. coil**

$\begin{array}{l} I_{rf}^{pk}[A]≃\frac{B_{rf}^{pk}[T]}{\mu [{\text{NA}}^{-2}]n_{rf}[m^{-1}]}≃\frac{1.356\times 10^{-4}}{\mu [{\text{NA}}^{-2}]n_{rf}[m^{-1}]l_{rf}[m]\langle \lambda_{i}\rangle \left[Å\right]}\\ ≃\frac{108}{n_{rf}[m^{-1}]l_{rf}[m]\langle \lambda_{i}\rangle \left[Å\right]}(\text{in air}/\text{vacuum}) \end{array}$   Eq. (260).

**rms current in r.f. coil at exact impedance matching (Z_0_ usually 50Ω**

$\begin{array}{l} I_{rf}^{rms}[A]=\frac{I_{rf}^{pk}[A]}{\sqrt{2}}≃\frac{9.592\times 10^{-5}}{\mu [{\text{NA}}^{-2}]n_{rf}[m^{-1}]l_{rf}[m]\langle \lambda_{i}\rangle \left[Å\right]}\\ ≃\frac{76.3}{n_{rf}[m^{-1}]l_{rf}[m]\langle \lambda_{i}\rangle \left[Å\right]}(\text{in air}/\text{vacuum}) \end{array}$   Eq. (261).

**Approximate maximum voltage in r.f. coil (long solenoid approx, length *l^rf^_axial_*, thickness in beam direction *l_rf_*, width perpendicular to *l^rf^_axial_* and *l_rf_* = *a_rf_*)**

$V_{rf}^{pk}[V]\approx 2.5\times 10^{4}\frac{n_{rf}[m^{-1}]l_{axial}^{rf}[m]a_{rf}[m]}{\langle \lambda_{i}\rangle \left[Å\right]}B_{0}[T]$   Eq. (266).

using *L* ≈ *µ_0_n_rf_*^2^*l_axial_A_rf_* (Eq. (264)) which can be restated as:

**Approximate self-inductance of long solenoid**

$\begin{array}{l} L[H]\approx 4\pi \times 10^{-7}{\left(n_{rf}\left[m^{-1}\right]\right)}^{2}l_{axial}^{rf}[m]A_{rf}[m^{2}]\\ \approx 1.26\times 10^{-6}{\left(n_{rf}\left[m^{-1}\right]\right)}^{2}l_{axial}^{rf}[m]A_{rf}[m^{2}] \end{array}$   Eq. (265).

**Required rms power supply voltage at exact impedance matching**

$V_{rms}^{PS}[V]≃\frac{152.7\sqrt{Z_{0}[\Omega ]R[\Omega ]}}{n_{rf}[m^{-1}]l_{rf}[m]\langle \lambda_{i}\rangle \left[Å\right]}=\frac{1079.4\sqrt{R\left[\Omega \right]}}{n_{rf}[m^{-1}]l_{rf}[m]\langle \lambda_{i}\rangle \left[Å\right]}$   Eq. (269).

**Heat dissipated in the whole r.f. circuit at exact impedance matching**

$P_{rf\;circuit}=2{\left(I_{rms}^{PS}\right)}^{2}Z_{0}=\frac{{\left(V_{rms}^{PS}\right)}^{2}}{2Z_{0}}$   Eq. (270).

**Power dissipated as heat in the r.f. coil at exact impedance matching**

$P_{coil}={\left|\frac{V_{rms}^{PS}}{2}\left(\frac{1}{Z_{0}}+j\omega C_{2}\right)\right|}^{2}R=\frac{{\left(V_{rms}^{PS}\right)}^{2}}{4Z_{0}}={\left(I_{rf}^{rms}\right)}^{2}R=\frac{P_{rf\;circuit}}{2}$   Eq. (273).

**Approximate maximum rate of change of current in r.f. coil**

${\left(dI/dt\right)}_{\max}[{\text{As}}^{-1}]=I_{rf}^{pk}[A]\omega_{rf}[s^{-1}]\approx 1.832\times 10^{8}I_{rf}^{pk}[A]B_{0}[T]$   Eq. (263).

**Approximate tolerances on r.f. coil**

Using the criteria $\Lambda_{FWHM}^{eff}\approx \sqrt{{\left(\frac{\Delta \left(B_{rf}l_{rf}\right)}{B_{rf}l_{rf}}\right)}^{2}+{\left(\frac{\Delta \lambda_{FWHM}}{\langle \lambda_{i}\rangle }\right)}^{2}}\leq 1.05\frac{\Delta \lambda_{FWHM}}{\langle \lambda_{i}\rangle }$and $\frac{\Delta \lambda_{FWHM}}{\langle \lambda_{i}\rangle }\leq 10\%$,

with $\frac{\Delta \left(B_{rf}l_{rf}\right)}{B_{rf}l_{rf}}=\frac{\sqrt{2}\Delta B_{rf}}{B_{rf}}=\frac{\sqrt{2}\Delta l_{rf}}{l_{rf}}<∼0.03$

for equal contributions to resolution function (Eq. (282)), we have

$\Delta B_{rf}^{pk}[T]≃\frac{2.9\times 10^{-6}}{l_{rf}[m]\langle \lambda_{i}\rangle \left[Å\right]}\;\text{Eq}.\;(283)\;\text{and}\frac{\Delta l_{rf}}{l_{rf}}<∼0.02$.

**Length of toroidal r.f. coil winding, toroid radius *r*, cross-section *a_rf_* × *b_rf_***

$l_{w}^{rf}\approx 4\pi r_{toroid}n_{rf}\left(a_{rf}+l_{rf}\right)$   Eq. (316).

**Self-inductance of toroidal solenoid of mean radius *r* and cross-sectional area *A_rf_***

$\begin{array}{l} L_{toroid}[H]=2\pi r_{toroid}\mu_{0}n_{rf}^{2}A_{rf}=8\pi^{2}\times 10^{-7}{\left(n_{rf}\left[m^{-1}\right]\right)}^{2}r_{toroid}[m]A_{rf}[m^{2}]\\ =7.9\times 10^{-6}{\left(n_{rf}\left[m^{-1}\right]\right)}^{2}r_{toroid}[m]A_{rf}[m^{2}] \end{array}$   Eq. (313).

**Resistance of toroidal solenoid of mean radius *r***

$R_{toroid}=\rho (T)\frac{4\pi r_{toroid}n_{rf}(a_{rf}+l_{rf})}{t_{rf}h_{rf}}$   Eq. (318).

Specifically for Al rectangular windings *t_rf_* × *h_rf_*:

$R_{Al}^{toroid}(T)[\Omega ]=\left(1.43\times 10^{-9}T[K]-8.7\times 10^{-8}\right)\frac{r_{toroid}[m]n_{rf}[m^{-1}]\left(a_{rf}+l_{rf}\right)[m]}{t_{rf}[m]h_{rf}[m]}$   Eq. (320).

**Stray field for total precession in arm of length *L* <10°**

$B_{stray}[T]L[m]\leq \frac{3.8\times 10^{-6}}{\langle \lambda_{i}\rangle \left[Å\right]}$   Eq. (285).

**Unshielded “zero field path” precession due to average stray field *B_stray_* along path length *L***

$\langle \Delta \varphi_{stray}\rangle [\text{rad}]\approx \frac{\gamma_{n}m_{n}}{h}B_{stray}L\langle \lambda_{i}\rangle =4.63\times 10^{4}B_{stray}[T]L[m]\langle \lambda_{i}\rangle \left[Å\right]$   Eq. (284).
